# Property-Driven Design of Thermally Robust Organophosphorus Ionic Liquids for High-Temperature Applications

**DOI:** 10.1021/acsaenm.5c00221

**Published:** 2025-05-05

**Authors:** Muhammadiqboli Musozoda, Andrew L. Bishuk, Blake J. Britton, Marija Scheuren, Charles H. Laber, Gary A. Baker, Matthew S. Baker, Matthias Zeller, Daniel H. Paull, Patrick C. Hillesheim, Arsalan Mirjafari

**Affiliations:** a Department of Chemistry, 14828 State University of New York at Oswego, Oswego, New York 13126, United States; b Department of Chemistry and Physics, 53713 Ave Maria University, Ave Maria, Florida 34142, United States; c Department of Chemistry, 14716 University of Missouri, Columbia, Missouri 65211, United States; d U.S. Army Engineer Research and Development Center, Vicksburg, Mississippi 39180, United States; e Department of Chemistry, Purdue University, West Lafayette, Indiana 47907, United States; f Department of Chemistry and Physics, 3391 Florida Gulf Coast University, Fort Myers, Florida 33965, United States; g Department of Chemistry, Illinois State University, Normal, Illinois 61761, United States

**Keywords:** functional organic materials, thermally robust materials, photoluminescent materials, organophosphorus π-conjugated salts, ionic liquids, molecular engineering, crystal engineering, structure−property−function relationships

## Abstract

We have developed a class of organophosphorus ionic materials featuring tetraarylphosphonium cations with extended π-conjugated systems via a facile and modular approach. These mesothermal ionic liquids demonstrate exceptional thermal stability, maintaining their structural integrity when heated at 300 °C for 96 h under aerobic conditions without decomposition. Their negligible volatility and strategic exclusion of aliphatic C­(sp^3^)–H bonds from our molecular architecture yields materials with outstanding resistance to thermo-oxidative degradation. Our rigorous investigation using comprehensive single-crystal X-ray diffraction and thermodynamic studies validates the design principles while providing detailed insights into the structure–property relationships governing their thermal stability, melting behavior, and photophysical properties. Our studies reveal a systematic correlation between the nature of the cations and the resulting phase transitions. Additionally, detailed photophysical characterization demonstrates that select derivatives exhibit strong fluorescence with quantum yields up to 42%, suggesting potential applications in optoelectronic devices. These thermally robust organic-ion materials with tunable properties have potential applications ranging from thermally demanding environments (thermoresponsive materials, advanced nuclear reactor coolants, and thermal energy storage) to optoelectronic devices that capitalize on their unique photoluminescent characters.

## Graphical abstract

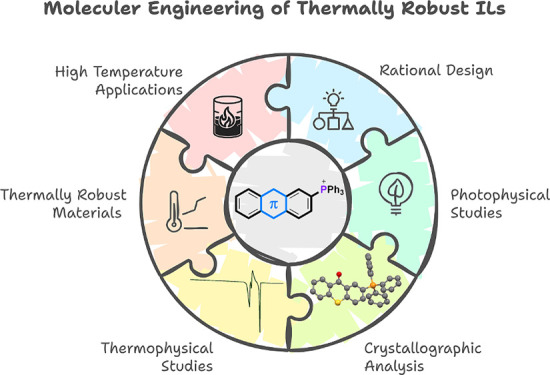

## Introduction

Advanced heat transfer fluids play a crucial role in modern thermal management systems. Recent advances in the field have resulted in the development of a new generation of heat transfer fluids with intended application in thermoresponsive materials,^1^ organic coolants for nuclear reactors,^2^ and thermal energy storage systems.^3–6^ An ideal heat transfer fluid must have several essential characteristics: high thermal stability, negligible vapor pressure, minimal corrosiveness, nonflammability, low toxicity, and low viscosity—all while maintaining cost-effectiveness. Among these properties, thermal stability is particularly vital as it determines the fluid’s maximum operating temperature.^7^

Conventional heat transfer fluids are primarily limited to low-to-medium temperature applications, although some polymers are suitable for high-temperature use.^8^ This limitation is particularly relevant for advanced nuclear reactors, where organic fluids as coolants could provide advantages over traditional pressurized and boiling water systems.^2^ However, current organic coolants based on peraryl compounds (Figure 1) have major drawbacks, including flammability, moderate-to-high toxicity, poor heat conductivity, and thermal instability that leads to decomposition or polymerization.^2^ In contrast to the currently employed technology, ionic liquids offer promising characteristics for advanced thermal applications, combining high long-term thermal stability, negligible vapor pressure, and low-melting points. These properties, coupled with the fact that many high-valent organophosphorous compounds serve as effective flame retardants,^9^ suggest that phosphonium-based ILs could excel as high-performance materials for thermally demanding conditions.

**Figure 1:**
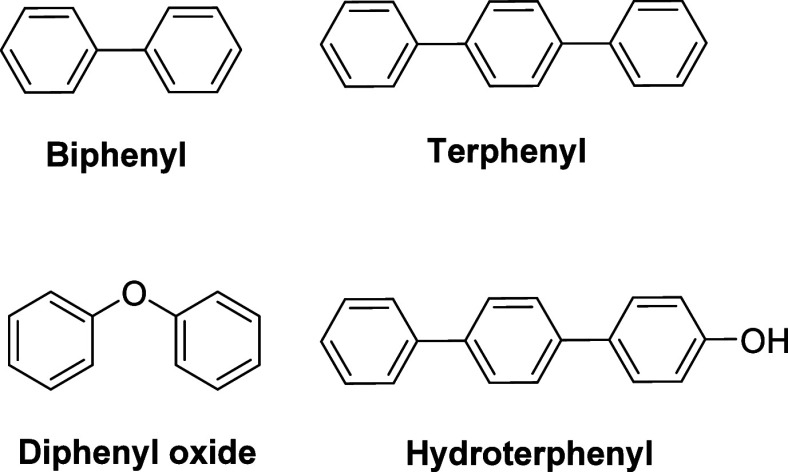
Chemical structures of organic coolants for nuclear reactor applications, illustrating the *para* isomer of biphenyl and terphenyl. In practice, mixtures of the *ortho*, *meta*, and *para* isomers are used.

Ionic liquids (ILs) have garnered considerable interest as versatile functional materials due to their distinctive physicochemical properties,^10^ enabling diverse applications that address key societal challenges in energy, sustainability, and healthcare.^11–19^ However, many new IL designs merely iterate on concepts from the 1990s.^20^ This stagnation in fundamental innovation suggests that the field maybe reaching a plateau in terms of novel molecular architectures, hindering our ability to both overcome current challenges while limiting new applications. Consequently, there is a pressing need to incorporate new design criteria and synthetic strategies into IL development, moving beyond the conventional cation designs that have dominated the literature for more than two decades.^21^

A major issue in IL research is that conventional ILs are significantly less thermostable than previously believed. This discrepancy primarily stems from differences between short-term and long-term thermal stability assessments.^22^ Traditional thermal stability testing involves rapid temperature ramping studies under an inert atmosphere, with researchers typically reporting the temperature of major decomposition steps as the measure of thermal stability.^23^ However, inconsistencies arise in how these decomposition temperatures are reported, with some studies using onset temperatures, others using 1–5% mass loss temperatures, and still others using various ratios of weight loss versus temperature.^24^ More importantly, these short-term decomposition temperatures often substantially overestimate the ILs’ actual thermal stability. Numerous studies have demonstrated that when ILs are subjected to extended thermal exposure, their long-term stability differs markedly from what short-term testing would suggest.^25,26^

Traditional IL cations (imidazolium, quaternary ammonium, and tetraalkylphosphonium) undergo decomposition at elevated temperature through anion-mediated retro-Menshutkin reaction or Hofmann elimination.^22^ To address thermal stability limitation in ILs, Davis and colleagues developed peraryl mesothermal ILs, bridging the gap between classical all-inorganic molten salts and conventional ILs.^27–31^ While molecular simulation can theoretically predict melting points with reasonable accuracy,^32^ such *a priori* predictions require knowledge of the crystal structure—an aspect that remains challenging to determine solely from molecular structure due to limitations in current crystal structure prediction methodologies.^33^ Despite these ILs being crystalline solids at room temperature, single-crystal X-ray diffraction (SCXRD) and solid-state structure–property relationship studies were notably absent from the literature, likely due to challenges in growing single-crystalline forms of most ILs.^34^

Motivated by our initial findings,^35^ and building on our longstanding interest in designing low-melting ILs with specific properties and molecular behavior/functions,^36^ we set out to develop triphenylphosphonium-based ILs with π-conjugated scaffolds, allowing for the targeting of a wide liquidus range that balances low-melting points with high thermal stability. This approach aligns with the growing interest in organophosphorus π-conjugated materials, which have emerged as an attractive research area due to the unique structural and electronic properties of their P-cationic centers, creating intriguing opportunities across a wide range of applications, including electronics, optics, and spintronics.^37–40^

Pursuing this strategy, we developed a homologous series of 15 ILs based on triphenylphosphonium cations with heteroatom-doped π-conjugated backbones, all exhibiting exceptionally high long-term thermal stability—i.e., 300 °C for 96 h under air (Figure 2). Under controlled conditions, these ILs remarkably formed single crystals, allowing for a deep understanding of how structural changes in the IL cation impact the supramolecular interactions. Within this work, we rigorously evaluated the structure–property relationships of these ILs via thermophysical methods (differential scanning calorimetry and thermogravimetric analysis), SCXRD, and photophysical techniques (UV–vis absorption and fluorescence spectroscopy). Several key structural principles influence the properties of these ionic materials. The strategic incorporation of hydrogen bond donors/acceptors and electron-donating/withdrawing groups allows for systematic tuning of melting points and thermal stability.

**Figure 2:**
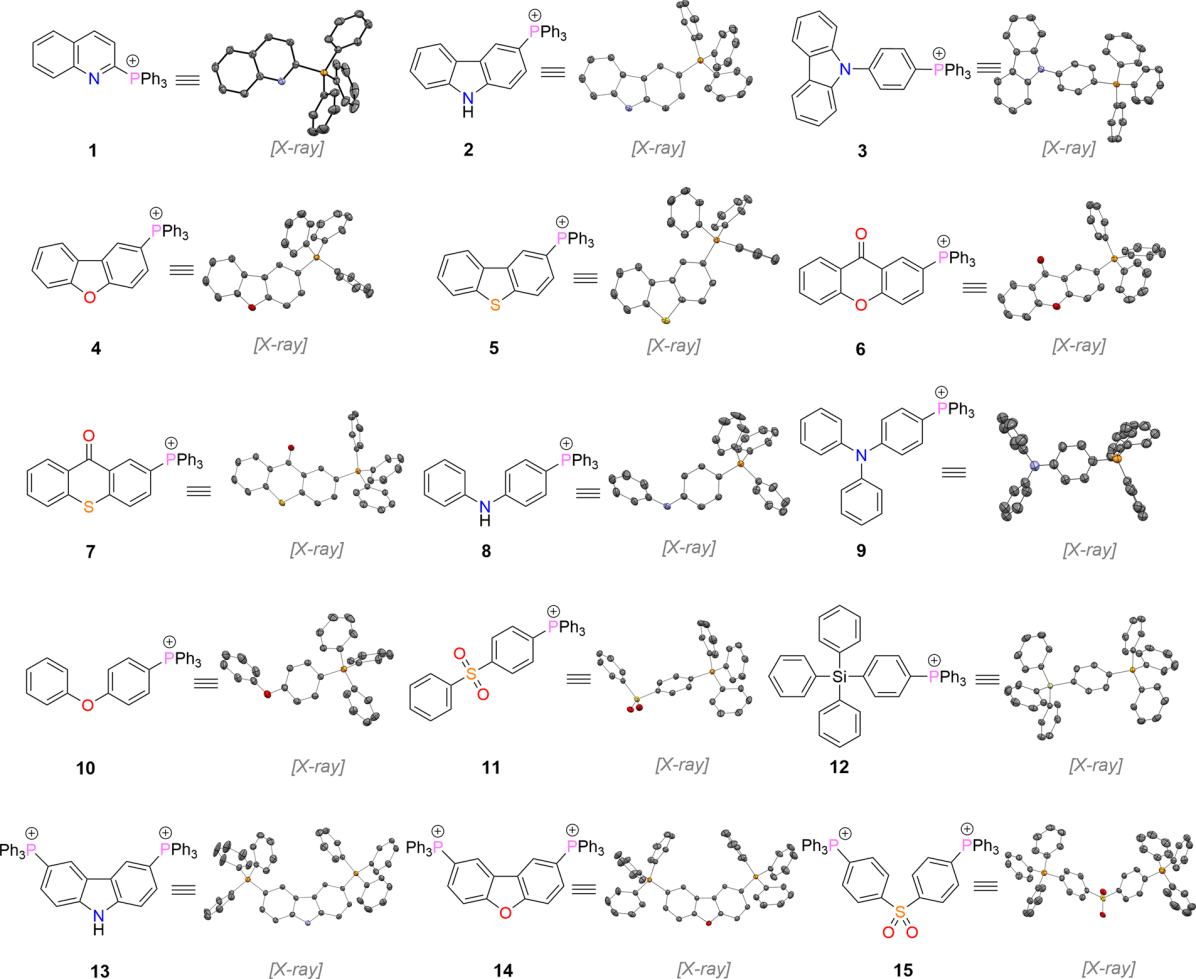
Chemical and crystal structures of tetraarylphosphonium ILs with diverse π-conjugated heterocyclic scaffolds. The [NTf_2_]^−^ counteranion is omitted for clarity. The numbering corresponds to the entries in Table 1 and throughout the text.

## Experimental Section

### Materials and Instrumentation

All commercial chemicals are used as received unless otherwise noted. ^1^H, ^13^C, and ^31^P NMR analyses were performed on a Bruker 500 MHz NMR instrument at 295 K with the chemical shifts (δ) notated as parts per million (ppm) and referenced to the residual ^1^H signal of CDCl_3_ as a solvent at room temperature.

The mass spectrometry (MS) data were obtained by using a Thermo Scientific Altis TSQ triple-quadrupole mass spectrometer. Samples were prepared at 10 ppm in LCMS-grade acetonitrile (Fisher Optima) and introduced into the MS directly by a syringe pump set to mix 5 uL/min of this solution into a 0.2 mL/min flow from the HPLC consisting of acetonitrile with 0.1% formic acid (Fisher Optima). Data were collected using the automated optimization for “selected reaction monitoring” (SRM) analysis in the Chromeleon software, using argon (1.5 mTorr) in the collision chamber and a capillary temperature of 325 °C. Each type of gas flow was optimized for each molecular ion and was within 20% of the default values.

The reported optimal isolation mass for the molecular *m*/*z* is the peak of this band; this is a low-resolution mass spectrometer; therefore, the mass agreement is uniformly excellent. This was optimized for the source voltage (CID), which was 0 V, except where otherwise noted. The capillary voltage (VCAP) was also optimized and is reported. The top 5 SRM reactions are reported with their max intensity at optimized collision energy/voltage (CV) for that product. These are reported with their relative maximum intensity as well as the intensity of the SRM ion relative to the molecular ion maximum intensity at CV = 0.

Melting points and glass transition temperatures were measured using a TA Discovery 250 DSC Differential Scanning Calorimeter calibrated using indium (melting point) and sapphire (heat capacity) references.

Thermogravimetric analyses were performed on a TA Instruments TGA 550 under nitrogen flow using a platinum pan. The samples were heated from room temperature at a rate of 25 °C/min to a maximum temperature of 500 °C under air.

Single-crystal XRD experiments were carried out with a Bruker AXS D8 Quest diffractometer with a PhotonII or PhotonIII charge-integrating pixel array detector (CPAD) and either a Mo Kα sealed X-ray tube or Cu Kα radiation microsource X-ray tube. Absorption was corrected by multiscan methods using *SADABS*. Additional details are provided in the Crystallographic Data section (Supporting Information).

### General Synthetic Procedure

The synthetic procedure follows our previously reported protocol with minor modifications.^41^ The synthesis proceeds in two steps: a nickel-catalyzed coupling reaction, followed by anion metathesis (Scheme 1). In the first step, a heavy-walled round-bottom flask equipped with a PTFE bushing pressure seal and egg-shaped magnetic stir bar is charged with equimolar amounts of aryl bromides (**a**), triphenylphosphine (**b**, ∼0.5 g), and 5 mol % anhydrous NiBr_2_ in degassed ethylene glycol (∼5 mL). Expectedly, the corresponding aryl chlorides were also attempted but proved unreactive under these conditions. After flushing the flask with N_2_ gas, it is immersed in a preheated oil bath at 190 °C, ensuring complete submersion of the reaction mixture. The reaction proceeds for 30 min (see the Supporting Information for kinetic studies), after which the flask is removed from the oil bath and cooled to room temperature. The reaction mixture is then worked up by diluting with dichloromethane (20 mL), deionized water (10 mL), and brine (10 mL). During the workup, the catalysts and ethylene glycol are partitioned into the aqueous phase while the desired tetraphenylphosphonium salts remain in the organic phase. Following separation of the layers, the aqueous phase is extracted with dichloromethane (2 × 20 mL). The combined organic layers are dried over anhydrous Na_2_SO_4_ and concentrated under reduced pressure. Washing the resulting solid with diethyl ether (3 × 15 mL) affords the bromide salt as a crystalline white solid, which is characterized by NMR (^1^H, ^13^C, and ^31^P) spectroscopy. The second step involves anion exchange, where the bromide salt is suspended in hot deionized water (∼50 °C and 30 mL). KNTf_2_ (1.2 equiv) is added in one portion to the hot suspension with stirring. White precipitates form within 30 min, after which the mixture is cooled to room temperature with continued stirring. The precipitated product is collected by vacuum filtration, yielding the desired final phosphonium NTf_2_ product as a white crystalline solid, except 7, which is a yellowish solid. The yields of both intermediate bromide salts and the final products are excellent (82–97%). The **1**–**15** compounds are characterized by NMR (^1^H, ^13^C, and ^31^P) spectroscopy and ESI-MS analysis.

**Scheme 1:**
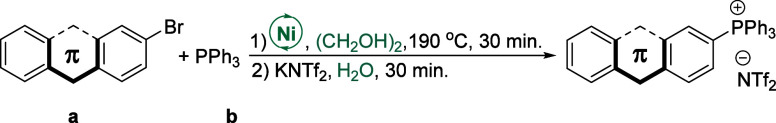
Two-Step Synthesis of Targeted Tetraarylphosphonium ILs Using Various Aryl Bromides (**a**) and Triphenylphosphine (**b**).

For X-ray crystallographic studies, the IL products were recrystallized by using vapor-diffusion methods with two different solvent systems: ILs **1**–**6**, **11**, **12**, **14**, and **15** were recrystallized using methanol as a solvent and Et_2_O as an antisolvent, while ILs **7**–**9** and **13** were recrystallized using dichloromethane as a solvent and cyclohexane as an antisolvent.

#### IL **1**

*T*_m_ = 124.7 °C; ^1^H NMR (500 MHz, CDCl_3_) δ 8.61 (m, 1H), 8.21 (d, *J =* 8.6 Hz, 1H), 8.06 (d, *J =* 8.4 Hz, 1H), 7.91 (m, 4H), 7.81 (m, 2H), 7.75 (m, 12H); ^13^C NMR (126 MHz, CDCl_3_) δ 149.2, 149.0, 145.4, 144.4, 139.2, 139.1, 135.8, 135.7, 134.8, 134.7, 132.1, 130.7, 130.6, 130.5, 130.0, 129.0, 129.0, 128.6, 125.2, 125.0, 121.2, 118.7, 117.8, 117.1, 116.1; ^31^P NMR (202 MHz, CDCl_3_) δ 14.7; MS (ESI): *m*/*z* 390.126 (M = C_27_H_21_NP^+^, calcd 390.141).

#### IL **2**

*T*_m_ = 153.0 °C; ^1^H NMR (500 MHz, CDCl_3_) δ 10.34 (s, 1H), 8.10 (dd, *J =* 13.5, 1.8 Hz, 1H), 8.00 (dd, *J =* 8.6, 2.9 Hz, 1H), 7.87 (m, 4H), 7.73 (td, *J* = 7.9, 3.6 Hz, 6H), 7.69 (d, *J* = 8.2 Hz, 1H), 7.64 (ddd, *J* = 12.8, 8.4, 1.3 Hz, 6H), 7.45 (m, 2H), 7.22 (m, 1H); ^13^C NMR (126 MHz, CDCl_3_) δ 143.9, 143.9, 140.8, 135.4, 135.4, 134.4, 134.3, 130.7, 130.5, 130.4, 130.0, 129.9, 127.9, 126.9, 126.8, 124.6, 124.5, 123.9, 121.4, 121.3, 120.7, 120.1, 119.6, 118.9, 118.7, 116.2, 114.2, 114.1, 112.6, 102.9, 102.1; ^31^P NMR (202 MHz, CDCl_3_) δ 24.3; MS (ESI): *m*/*z* 428.189 (M = C_30_H_23_NP^+^, calcd 428.156).

#### IL **3**

*T*_m_ = 171.0 °C; ^1^H NMR (500 MHz, CDCl_3_) δ 8.11 (d, *J =* 7.5 Hz, 4H), 7.99 (dd, *J =* 9.0, 2.6 Hz, 2H), 7.91 (dd, *J =* 9.4, 1.6 Hz, 2H), 7.87 (m, 1H), 7.80 (t, *J =* 7.7 Hz, 6H), 7.70 (m, 6H), 7.61 (d, *J =* 8.2 Hz, 2H), 7.44 (ddd, *J =* 8.4, 7.1, 1.2 Hz, 2H), 7.33 (m, 2H); ^13^C NMR (126 MHz, CDCl_3_) δ 144.9, 139.5, 136.3, 136.2, 136.2, 135.9, 134.5, 134.4, 134.3, 130.9, 130.8, 127.6, 127.5, 126.7, 124.4, 123.8, 121.6, 121.2, 120.6, 118.7, 117.8, 117.1, 115.0, 114.2, 109.8; ^31^P NMR (202 MHz, CDCl_3_) δ 22.9; MS (ESI): *m*/*z* 504.165 (M = C_36_H_27_NP^+^, calcd 504.188).

#### IL **4**

*T*_m_ = 104.3 °C; ^1^H NMR (500 MHz, CDCl_3_) δ 8.16 (dd, *J =* 12.8, 1.8 Hz, 1H), 7.97 (dt, *J =* 7.8, 1.0 Hz, 1H), 7.91 (m, 4H), 7.77 (m, 6H), 7.68 (m, 8H), 7.58 (ddd, *J =* 8.5, 7.3, 1.3 Hz, 1H), 7.42 (td, *J =* 7.5, 1.0 Hz, 1H); ^13^C NMR (126 MHz, CDCl_3_) δ 159.8, 157.0, 135.8, 135.8, 134.5, 134.4, 132.9, 132.8, 130.9, 130.8, 129.6, 127.7, 127.6, 127.0, 126.9, 124.3, 121.9, 121.7, 121.2, 118.6, 118.3, 117.6, 114.6, 114.4, 112.2, 111.1, 110.4; ^31^P NMR (202 MHz, CDCl_3_) δ 24.2; MS (ESI): *m*/*z* 429.140 (M = C_30_H_22_OP^+^, calcd 429.140).

#### IL **5**

*T*_m_ = 134.2 °C; ^1^H NMR (500 MHz, CDCl_3_) δ 8.25 (m, 2H), 8.02 (d, *J =* 7.8 Hz, 1H), 7.92 (m, 4H), 7.78 (td, *J =* 7.9, 3.7 Hz, 6H), 7.63 (m, 8H), 7.51 (m, 1H); ^13^C NMR (126 MHz, CDCl_3_) δ 147.5, 147.5, 139.8, 137.0, 136.9, 135.9, 135.8, 134.5, 134.4, 133.5, 130.9, 130.8, 130.4, 130.3, 128.8, 127.5, 127.4, 125.7, 125.4, 125.3, 123.8, 123.1, 122.3, 121.2, 118.7, 118.2, 117.5, 116.1, 112.8, 112.1; ^31^P NMR (202 MHz, CDCl_3_) δ 24.2; MS (ESI): *m*/*z* 445.141 (M = C_30_H_22_PS^+^, calcd 445.117).

#### IL **6**

*T*_m_ = 153.9 °C; ^1^H NMR (500 MHz, CDCl_3_) δ 8.53 (m, 1H), 8.26 (d, *J =* 8.0 Hz, 1H), 7.95 (m, 5H), 7.79 (m, 7H), 7.64 (dd, *J =* 13.4, 8.1 Hz, 7H), 7.46 (t, *J =* 7.6 Hz, 1H); ^13^C NMR (126 MHz, CDCl_3_) δ 175.4, 159.9, 159.9, 156.0, 138.9, 138.8, 136.4, 136.0, 136.0, 134.8, 134.7, 134.4, 134.4, 131.0, 130.9, 130.2, 126.8, 125.5, 122.7, 122.6, 122.1, 122.0, 121.7, 121.2, 118.6, 118.6, 117.5, 116.8, 113.4, 112.7; ^31^P NMR (202 MHz, CDCl_3_) δ 23.1; MS (ESI): *m*/*z* 457.115 (M = C_31_H_22_O_2_P^+^, calcd 457.135).

#### IL **7**

*T*_m_ = 202.6 °C; ^1^H NMR (500 MHz, CDCl_3_) δ 8.77 (dd, *J* = 14.1, 2.0 Hz, 1H), 8.53 (dd, *J* = 8.1, 1.5 Hz, 1H), 8.04 (dd, *J* = 8.5, 2.9 Hz, 1H), 7.90 (m, 3H), 7.86 (m, 1H), 7.77 (td, *J* = 7.9, 3.7 Hz, 6H), 7.73 (m, 1H), 7.65 (m, 7H), 7.56 (ddd, *J* = 8.1, 7.0, 1.2 Hz, 1H); ^13^C NMR (126 MHz, CDCl_3_) δ 178.4, 146.0, 146.0, 136.4, 136.3, 136.2, 136.0, 135.8, 135.3, 135.2, 134.5, 134.4, 134.3, 133.6, 130.9, 130.8, 130.7, 130.6, 129.9, 129.7, 129.4, 128.9, 127.7, 126.6, 123.8, 121.2, 118.6, 117.5, 116.8, 116.1, 115.7, 114.9; ^31^P NMR (202 MHz, CDCl_3_) δ 23.2; MS (ESI): *m*/*z* 473.139 (M = C_31_H_22_OPS^+^, calcd 473.112).

#### IL **8**

*T*_m_ = 164.4 °C; ^1^H NMR (500 MHz, CDCl_3_) δ 7.84 (d, *J* = 1.9 Hz, 3H), 7.70 (td, *J* = 8.0, 3.6 Hz, 7H), 7.59 (m, 7H), 7.32 (m, 2H), 7.26 (d, *J* = 4.4 Hz, 4H), 7.21 (s, 1H), 7.08 (s, 1H); ^13^C NMR (126 MHz, CDCl_3_) δ 151.5, 151.5, 139.3, 135.9, 135.8, 135.3, 135.2, 134.4, 134.3, 134.2, 134.1, 130.7, 130.5, 130.4, 129.5, 124.6, 123.8, 122.3, 121.2, 119.5, 118.7, 116.1, 115.8, 115.7, 101.2, 100.4; ^31^P NMR (202 MHz, CDCl_3_) δ 22.0; MS (ESI): *m*/*z* 430.191 (M = C_30_H_25_NP^+^, calcd 430.172).

#### IL **9**

*T*_g_ = 164.4 °C; ^1^H NMR (500 MHz, CDCl_3_) δ 7.75 (m, 4H), 7.64 (td, *J =* 7.9, 3.5 Hz, 9H), 7.51 (ddd, *J =* 13.0, 8.5, 1.3 Hz, 9H), 7.29 (m, 6H), 7.16 (dt, *J =* 9.2, 1.4 Hz, 11H), 7.11 (t, *J =* 1.2 Hz, 1H), 6.96 (dd, *J =* 9.0, 2.9 Hz, 3H); ^13^C NMR (126 MHz, CDCl_3_) δ 153.9, 153.9, 145.0, 144.7, 144.5, 135.5, 135.4, 135.4, 134.7, 134.2, 134.1, 130.6, 130.5, 130.2, 127.0, 126.5, 121.3, 119.1, 118.7, 118.5, 118.4, 118.4, 103.1, 102.3; ^31^P NMR (202 MHz, CDCl_3_) δ 22.0; MS (ESI): *m*/*z* 506.250 (M = C_36_H_29_NP^+^, calcd 506.203).

#### IL **10**

*T*_m_ = 103.6 °C; ^1^H NMR (500 MHz, CDCl_3_) δ 7.87 (m, 3H), 7.74 (td, *J* = 7.9, 3.6 Hz, 6H), 7.60 (ddd, *J* = 13.0, 8.4, 1.3 Hz, 6H), 7.46 (m, 4H), 7.26 (m, 1H), 7.21 (m, 1H), 7.19 (s, 1H), 7.13 (dd, *J* = 8.6, 1.1 Hz, 2H); ^13^C NMR (126 MHz, CDCl_3_) δ 164.5, 164.5, 153.8, 136.6, 136.5, 135.7, 135.6, 134.3, 134.2, 130.7, 130.6, 130.5, 126.0, 123.8, 121.2, 120.9, 118.8, 118.6, 118.3, 117.6, 109.2, 108.5; ^31^P NMR (202 MHz, CDCl_3_) δ 22.6; MS (ESI): *m*/*z* 431.210 (M = C_30_H_24_OP^+^, calcd 431.156).

#### IL **11**

*T*_m_ = 151.7 °C; ^1^H NMR (500 MHz, CDCl_3_) δ 8. 8.21 (dd, *J* = 8.6, 2.8 Hz, 2H), 7.95 (dd, *J* = 8.5, 1.3 Hz, 2H), 7.87 (m, 3H), 7.81 (m, 2H), 7.73 (td, *J* = 7.9, 3.7 Hz, 6H), 7.63 (t, *J* = 7.4 Hz, 1H), 7.57 (m, 8H); ^13^C NMR (126 MHz, CDCl_3_) δ 148.4, 148.4, 139.3, 136.1, 136.1, 135.6, 135.5, 134.5, 134.5, 134.4, 131.0, 130.9, 129.9, 129.2, 129.1, 128.3, 124.2, 123.7, 123.5, 121.1, 118.6, 116.7, 116.0; ^31^P NMR (202 MHz, CDCl_3_) δ 23.2; MS (ESI): *m*/*z* 479.109 (M = C_30_H_24_O_2_PS^+^, calcd 479.123).

#### IL **12**

*T*_m_ = 211.9 °C; ^1^H NMR (500 MHz, CDCl_3_) δ 7.89 (m, 4H), 7.76 (dt, *J =* 7.5, 4.3 Hz, 6H), 7.58 (m, 14H), 7.45 (m, 6H), 7.39 (d, *J =* 6.7 Hz, 4H); ^13^C NMR (126 MHz, CDCl_3_) δ 145.8, 145.8, 137.9, 137.8, 136.3, 135.9, 135.9, 134.4, 134.3, 133.1, 133.0, 132.1, 130.9, 130.8, 130.4, 128.3, 119.2, 118.5, 117.6, 116.9; ^31^P NMR (202 MHz, CDCl_3_) δ 23.1; MS (ESI): *m*/*z* 597.22 (M = C_42_H_34_PSi^+^, calcd 597.216).

#### IL **13**

*T*_m_ = 240.3 °C; ^1^H NMR (500 MHz, CDCl_3_) δ 11.08 (s, 1H), 8.12 (m, 4H), 7.87 (m, 7H), 7.72 (dt, *J* = 7.7, 4.1 Hz, 12H), 7.62 (m, 13H); ^13^C NMR (126 MHz, CDCl_3_) δ 144.6, 135.4, 135.4, 134.5, 134.4, 131.7, 130.6, 130.5, 128.5, 128.4, 121.1, 119.0, 118.6, 118.3, 115.3, 115.2; ^31^P NMR (202 MHz, CDCl_3_) δ 24.3.; MS (ESI): *m*/*z* 344.581 (M = C_48_H_37_NP_2_^2+^, calcd 344.620).

#### IL **14**

*T*_m_ = 182.6 °C; ^1^H NMR (500 MHz, CDCl_3_) δ 8.21 (dd, *J* = 13.1, 1.8 Hz, 2H), 7.95 (dd, *J* = 8.7, 2.4 Hz, 2H), 7.84 (m, 6H), 7.69 (m, 26H); ^13^C NMR (126 MHz, CDCl_3_) δ 160.4, 160.4, 135.5, 135.5, 134.7, 134.6, 134.1, 134.0, 130.7, 130.6, 130.3, 130.2, 125.1, 125.0, 121.0, 118.4, 118.2, 117.5, 115.9, 114.7, 114.5, 112.8, 112.0; ^31^P NMR (202 MHz, CDCl_3_) δ 24.2; MS (ESI): *m*/*z* 345.085 (M = C_48_H_36_OP_2_^2+^, calcd 345.112).

#### IL **15**

*T*_m_ = 198.7 °C; ^1^H NMR (500 MHz, CDCl_3_) δ 8.33 (dd, *J* = 8.5, 2.8 Hz, 3H), 7.86 (m, 10H), 7.73 (td, *J* = 7.9, 3.7 Hz, 13H), 7.61 (ddd, *J* = 13.2, 8.5, 1.2 Hz, 12H); ^13^C NMR (126 MHz, CDCl_3_) δ 146.1, 146.1, 135.9, 135.9, 135.8, 134.8, 134.7, 134.6, 134.5, 131.0, 130.9, 130.9, 130.8, 130.8, 130.0, 129.9, 124.9, 124.2, 123.6, 121.0, 118.5, 116.8, 116.1, 115.9; ^31^P NMR (202 MHz, CDCl_3_) δ 23.2; MS (ESI): *m*/*z* 370.049 (M = C_48_H_38_O_2_P_2_S^2+^, calcd 370.103).

### UV–vis Spectroscopy

UV–vis for the bromide salt of IL **2** was carried out on a Thermo Scientific Evolution 220 Spectrophotometer from 190 to 400 nm with a resolution of 1 nm (Figure S1). The spectrum was taken using acetonitrile as the solvent and a quartz cuvette with a 4 mm path length. The maximum absorption for this compound was determined to be at approximately 200 nm.

### HPLC Method and Instrument

High-performance liquid chromatography (HPLC) analyses were performed using a Dionex UltiMate 3000 series system with an RS Variable Wavelength Detector and analyzed at a wavelength of 200 nm. The column used was a Restek (250 × 4.6 mm) reverse-phase C18 column. The gradient for this experiment was a 9:1 acetonitrile/water mixture for 2 min, followed by a ramp to pure acetonitrile for over 6 min. Pure acetonitrile was run for an additional 2 min, resulting in a 10 min run with a flow rate of 1.0 mL/min.

### Reaction Procedure for the Kinetic Study

To a oven-dried round-bottom flask equipped with a stir bar and reflux condenser were added 3-bromocarbazole (0.20 g, 0.81 mmol), triphenylphosphine (0.21 g, 0.81 mmol), and NiBr_2_ (0.02 g, 0.08 mmol). The reaction vessel was purged with N_2_ three times. Ethylene glycol (15 mL) was degassed with N_2_ and then added to the reaction vessel. The reaction mixture was heated at 190 °C until complete. Aliquots (200 μL) were removed at 15 min intervals and diluted in 800 μL of acetonitrile. Subsequently, 100 μL of the stock solution were removed and added to 900 μL of acetonitrile. The final solution was analyzed via HPLC (Figures S2 and S3).

### Differential Scanning Calorimetry

In this work, melting points are reported as the transition from the crystalline solid state to the isotropic liquid state, distinguished by the magnitude of enthalpy for the transition and shape of the DSC curve. For each experiment, 5–10 mg of the sample were loaded into an open aluminum pan and heated to 120 °C for 20 min to remove any water absorbed from the environment, residual solvents, or volatile contaminants from synthesis. The samples were then cooled to −50 °C, equilibrated for 2 min, and then heated at a ramp rate of 5 °C/min to 200 °C. Determined by the TRIOS analysis software, melting points are reported as the melting onset temperature and glass transition temperatures are reported as the midpoints of the phase transitions. The samples underwent 8–10 heating and subsequent cooling processes at a rate of 5 °C/min, alternating with 10 min isothermal periods to identify the correct phase transitions by observing three overlapping cycles and reported values are the average of three measurements. All measurements were carried out under a nitrogen atmosphere (50 mL/min) and were reproducible to ± 1 °C.

At the melting point, equilibrium exists between the solid and liquid phases, and the change in Gibbs free energy is zero:

$$\Delta G_{\mathrm{fus}}=0=\Delta H_{\mathrm{fus}}-T_{m}\Delta S_{\mathrm{fus}}\;\Rightarrow \;T_{m}=\Delta H_{\mathrm{fus}}/\Delta S_{\mathrm{fus}}$$

where Δ*H*_fus_ is the enthalpy of fusion, *T*_m_ is the melting point, and Δ*S*_fus_ is the enthalpy of fusion. The *T*_m_ is determined as a delicate balance between the enthalpy and entropy of fusion. Note that decreases in the enthalpy and increases in entropy result in a melting point reduction.

### Long-Term Thermal Stability Analysis

Samples (∼0.3 g) of each IL were placed in new, uncovered 15 mL porcelain crucibles and heated at 300 °C in air using a muffle furnace for 96 h. After thermal exposure, samples were cooled to room temperature and weighed to determine the mass loss. The structural integrity of the thermally stressed samples was evaluated using NMR spectroscopy (^1^H, ^13^C, and ^31^P) and ESI-MS analysis. This methodology follows established protocols for evaluating extended thermal stability of ILs under aerobic conditions.^27^

### Photophysical Experiments

To perform photophysical measurements, triphenylphosphonium-based salt (**1**–**15**) stocks were prepared at a millimolar concentration in ethanol. Stocks were diluted to ∼10 μM in ethanol to perform optical measurements. UV–vis spectra were recorded on an Agilent Cary 60 spectrophotometer in dual beam mode equipped with a circulating constant-temperature bath for the sample chamber held at 25 °C. The scan rate was typically 600 nm/min, and all spectra were blank corrected. Steady-state fluorescence experiments were performed with a Shimadzu RF-6000 spectrofluorometer using a scan speed of 600 nm/min and excitation and emission bandpasses of 3.0 nm. All emission spectra were blank corrected. Fluorescence quantum yield (QY) values were determined using the Parker–Rees method using the relation:^42^

$${\mathrm{QY}}_{u}={\mathrm{QY}}_{r}\left(\frac{A_{r}}{A_{u}}\right)\left(\frac{F_{u}}{F_{r}}\right)\left(\frac{{n_{u}}^{2}}{{n_{r}}^{2}}\right)$$

In this expression, *A*_u_ and *A*_r_ denote the absorbance of the unknown and reference sample at the chosen excitation wavelength (λ_ex_ = 280 or 350 nm), and *F*_u_ and *F*_r_ represent the total, integrated fluorescence intensity for the unknown and references sample when excited at the same excitation wavelength, respectively. The refractive indices of the solvents in which the unknown and reference samples are prepared are given by *n*_u_ and *n*_r_, respectively. The quantum yield standards used in this study were quinine sulfate in 0.1 M H_2_SO_4_ (QY = 0.577, λ_ex_ = 350 nm) and l-tryptophan in water (QY = 0.13, λ_ex_ = 280 nm).

## Results and Discussion

Following the established nickel-catalyzed coupling reaction,^41^ we synthesized 15 triphenylphosphonium-based ionic fluids incorporating a [NTf_2_]^−^ counteranion via a modular strategySupporting Information (Figure 2). Our decision to employ the [NTf_2_]^−^ anion was guided by its well-established thermal stability with a decomposition temperature of ≥420 °C^43^ and its demonstrated chemical inertness toward organophosphorus cations compared to other anions.^30^ Except ILs **10** and **11**,^29^ all compounds are new and were synthesized using commercially available aryl bromide building blocks to create diverse cation backbones. This expanded structural scope allowed us to examine both electronic and steric effects on the thermal and optical properties of these ILs, providing valuable structure–property insights while broadening the range of functional groups used in IL development.

We optimized the synthesis of tetraarylphosphonium salts by conducting a systematic kinetic study to resolve the literature discrepancies. By monitoring the Ni-catalyzed coupling between 3-bromocarbazole and Ph_3_P (selected as our model substrate) via HPLC, we demonstrated that the reaction achieves high yields in just 30 min under optimized conditions (Supporting Information, Figures S2 and S3). This represents a notable improvement in synthetic efficiency while maintaining the high product purity essential for establishing reliable structure–property correlations for the IL products.

### Short-Term and Long-Term Thermochemical Evaluation

The thermal stability of these compounds was rigorously evaluated using two complementary approaches. For a short-term stability assessment, we conducted gradient thermogravimetric analysis (TGA) under dynamic heating conditions in air. While long-term isothermal studies are more representative of realistic thermal stability (*vide infra*), 5% mass loss (*T*_onset5%_) still provides useful comparisons about the relative stability of the species for studying causes of thermal decomposition. Remarkably, the data revealed excellent thermal robustness, with onset temperatures for *T*_onset5%_ ranging from 404.2 to 471.0 °C (Table 1 and Figure 3). The notable exception was **12** (triphenylsilyl), which not only showed poor thermal stability (*T*_onset5%_ < 400 °C) but also failed extended thermal stability testing. This reduced thermal stability can be attributed to silicon’s electronic effects. Presumably, silicon can stabilize aromatic radicals through σ-hyperconjugation,^44^ which facilitates the cleavage of the C–P bond under thermal stress. We confirmed this degradation pathway through a sublimation experiment, where the collected white solid was identified as Ph_3_P through ^1^H and ^31^PNMR spectroscopy.

**Table 1:** Thermal Data of the Triphenylphosphonium-type ILs^b^

IL	*T*_onset5%_ (°C) (±0.2–0.9%)	% mass loss (±1%)	*T*_m_ (°C) (±0.1–0.8%)	*T*_g_ (°C) (±0.2)	Δ*H* ^fus^ (kJ mol^–1^) (±0.1–0.7%)	Δ*S* ^fus^ (J mol^–1^K^–1^) (±0.3–1.1%)
**1**	431.1	70	124.7		52.7	88.9
**2**	430.9	0	153.0		41.1	68.3
**3** ^a^	444.5	3	133.7, 171.1	47.1	34.1	63.5
**4**	404.2	4	104.3		42.7	80.4
**5**	434.2	0	134.2		31.9	56.9
**6**	450.9	0	153.9		22.0	38.0
**7** ^a^	443.5	0	142.9, 202.6		56.5	104.0
**8**	437.8	3	164.3		36.5	24.9
**9**	454.2	2		38.7		
**10**	446.4	3	103.6	2.0	47.2	89.1
**11**	453.7	4	151.7	31.9	53.1	95.0
**12**	332.5	37	211.9			63.9
**13** ^a^	471.0	0	219.7, 240.3		48.9	43.2
**14**	461.5	0	182.6		30.0	82.3
**15**	424.5	0	198.7		43.8	120.8

a Two melting temperatures correspond to the distinct polymorphic phase transition.

b Long-term thermal stability tests were conducted at 300 °C for 96 h in a muffle furnace in the air.

**Figure 3:**
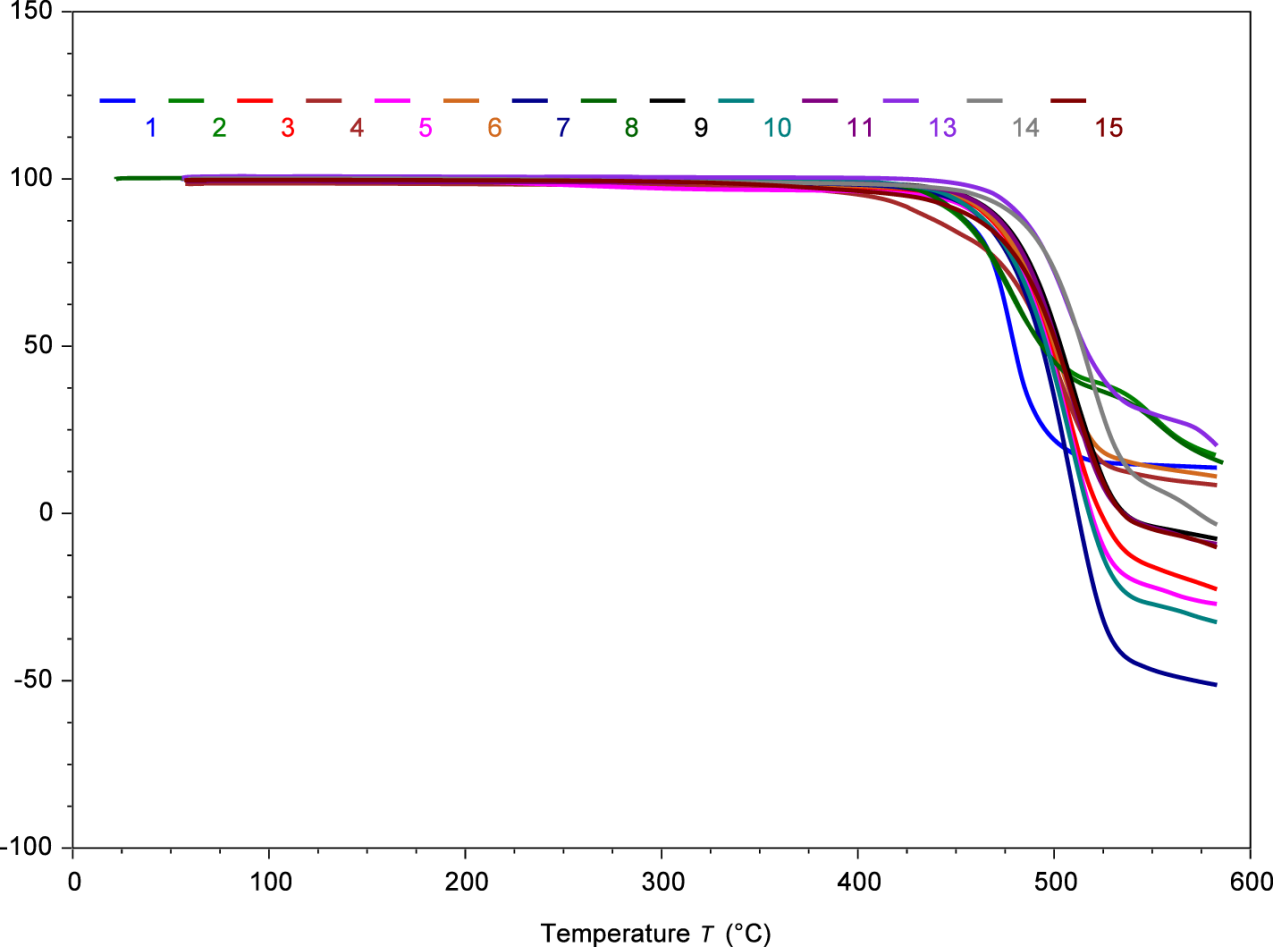
TG analysis demonstrating the short-term thermal stability of the tetraarylphosphonium ILs. These salts exhibit exceptional stability above 400 °C under aerobic conditions. IL **12** is excluded due to its lower stability. The number at the top corresponds to the respective IL.

To assess the long-term stability under function-relevant conditions, we conducted extended isothermal studies at elevated temperatures. Per our standard approach for evaluating long-term thermal stability,^35^ the salts were charred in porcelain crucibles and heated in air at 200, 250, and 300 °C in a muffle furnace for 96 h (Table 1). Decomposition was monitored via mass loss and spectroscopic changes. NMR analyses (^1^H, ^13^C, and ^31^P) of the compounds after thermal exposure were identical to those prior to heating (see the Supporting Information, Figure S5). A low mass loss of was noted in most of the samples, which is attributed to water loss, with samples appearing slightly darker than unheated materials.^31^ The presence of the basic nitrogen in IL **1** (quinoline) likely facilitates β-hydrogen elimination, leading to thermal decomposition at elevated temperatures.

The exceptional long- and short-term thermal stabilities demonstrated by these compounds can be attributed to the rigid aromatic structure of the cations and the absence of aliphatic C­(sp^3^)–H, which prevents common decomposition pathways typically seen in conventional ILs.^32^

### Thermal and Thermodynamics Analysis

The thermal characterization of these ILs using differential scanning calorimetry (DSC) yielded comprehensive phase behavior data. Table 1 shows the melting points (*T*_m_), glass transition temperatures (*T*_g_), enthalpy of fusion (Δ*H* ^fus^), and entropies of fusion (Δ*S* ^fus^). The results reveal distinct *T*_m_ values ranging from 103.6 to 211.9 °C for monocationic (**1**–**12**) and from 181.5 to 240.3 °C for dicationic ILs (**13**–**15**). With the exception of IL **9** (triphenylamine), all compounds exhibited sharp endothermic peaks, indicating well-defined melting transitions (Figure 4). These peraryl-based compounds form crystalline solids rather than isotropic liquids, classifying them as mesothermal ILs with relatively high-melting points attributed to both supramolecular interactions from π-conjugated moieties and the rigid tetraarylphosphonium structure (*vide infra*).

**Figure 4:**
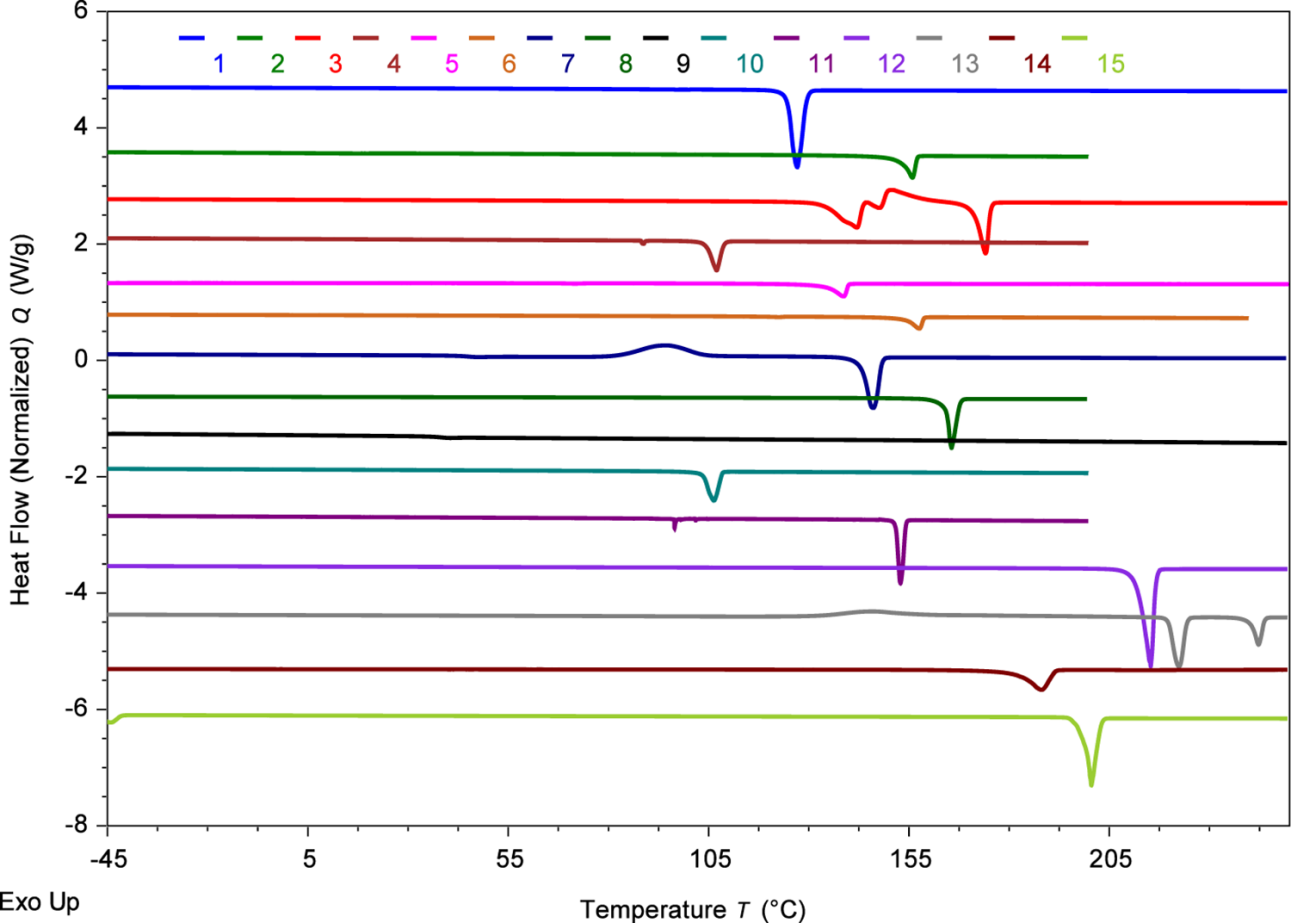
DSC thermogram of tetraarylphosphonium ILs **1**–**15** with thermograms, displaying key phase transitions, offset along the heat flow (*y*) axis for clarity but not rescaled. The number at the top corresponds to the respective IL.

To understand how structural modifications impact thermal behaviors, we analyzed the relationship between molecular structure and phase transitions using thermodynamic parameters, determining whether changes in the ILs’ fluidity are driven by enthalpic effects, entropic effects, or a combination of both (Table 2). IL melting points can be modified via two approaches: reducing the surface charge density of the ions or by decreasing molecular symmetry—both of which impact Δ*H* ^fus^ and Δ*S* ^fus^.^32^ In our perarylphosphonium systems, the structural elements providing thermal stability, particularly the aryl motifs, inherently lead to a higher *T*_m_.

**Table 2:** Comparison of Thermal and Thermodynamic Properties between the Monocationic ILs (**1**–**12**) and the [Ph_4_P]­[NTf_2_] Reference Compound (*T*_m_ = 135.0 °C, Δ*H* ^fus^ = 32.3 kJ mol^–1^, Δ*S* ^fus^ = 79.2 J mol^–1^ K^–1^),^29^ Analyzing How Structural Modifications Impact These Properties^a^

IL	Δ*T*_ref→*i*_(°C)	*T*_m,i_/*T*_m,ref._ × 100%	Δ*H* ^fus,i^/Δ*H* ^fus,ref.^ × 100%	Δ*S* ^fus,i^/Δ*S* ^fus,ref.^ × 100%	driving force
**1**	–10.3	92%	163%	112%	entropy-dominated *T*_m_ decrease
**2**	+18.0	113%	127%	86%	enthalpy–entropy cooperative *T*_m_ increase
**3**	+36.1	127%	105%	80%	enthalpy–entropy cooperative *T*_m_ increase
**4**	–30.7	77%	132%	101%	entropy-dominated *T*_m_ increase
**5**	–0.8	99%	99%	71%	enthalpy-dominated *T*_m_ decrease
**6**	+18.9	114%	68%	48%	entropy-dominated *T*_m_ increase
**7**	+67.6	150%	175%	131%	enthalpy-dominated *T*_m_ increase
**8**	+29.4	121%	113%	31%	enthalpy–entropy cooperative *T*_m_ increase
**9**					
**10**	–31.4	76%	146%	112%	entropy-dominated *T*_m_ decrease
**11**	+16.7	112%	164%	120%	enthalpy-dominated *T*_m_ increase
**12**	+76.9	157%	198%	151%	enthalpy-dominated *T*_m_ increase

a The data reveal that while we can identify whether entropy or enthalpy primarily drives the changes in thermal behavior, both factors change simultaneously with similar magnitudes but in opposite directions, which are nearly offsetting.

Using tetraphenylphosphonium bistriflimide ([Ph_4_P]­[NTf_2_]) as a reference compound, we systematically evaluated how replacing a single phenyl group with various heteroatom-based π-conjugated scaffolds affects the thermal behavior. Table 2 lists relative changes in Δ*T*_m_ alongside the ratios of Δ*H* ^fus^ and Δ*S* ^fus^, comparing monocationic salts to [Ph_4_P]­[NTf_2_]. Despite significant variations in both Δ*H* ^fus^ and Δ*S* ^fus^, these parameters often changed in parallel directions, nearly offsetting each other’s influence on *T*_m_. This complexity demonstrates the challenge of establishing precise structure–property relationships for IL materials. Five key patterns emerged from our analysis:

1. **Nitrogen-containing heterocycles** showed diverse behaviors. Despite quinoline providing an extended π-electron system, the *T*_m_ of IL **1** is lower than [Ph_4_P]­[NTf_2_], which was primarily driven by entropic effects (Table 2), attributable to increased molecular asymmetry introduced by the quinoline moiety. In contrast, ILs **2** (carbazole), **3** (*N*-phenylcarbazole), and **8** (diphenylamine) exhibited increased *T*_m_ through combined enthalpic and entropic effects, manifested by concurrent decreases in Δ*H* ^fus^ and increases in Δ*S* ^fus^. This can be attributed to highly polar N–H moieties that form hydrogen bonds, creating crystal structures with increased anion–cation interactions (*vide infra*). Specifically, these N–H moieties allow for the formation of multiple H bonds with an [NTf_2_]^−^ anion in each cation–anion pair, contributing significantly to the higher melting points observed in these compounds.
2. **Oxygen-containing linkers** in ILs **4** (dibenzofuran) and **10** (diphenyl ether) decreased *T*_m_ values, driven by entropic factors. Thermodynamic analysis revealed increases in Δ*H* ^fus^ and Δ*S* ^fus^ in both ILs **4** and **10**. These changes can be attributed to two phenomena: (i) the *p*-phenoxy substituents weaken cation–anion interactions, leading to a reduction in lattice energy and corresponding increase in Δ*H* ^fus^; (ii) the decreased molecular symmetry imparted by the phenoxy groups disrupts solid-phase packing, leading to a larger entropy gain upon melting. Conversely, the highly polar C^+^–O^–^ motif in IL **6** (xanthone) substantially increased the melting point (Δ*T*_m_ = 49.6 °C), concomitant with decreases in Δ*H* ^fus^ and Δ*S* ^fus^.
3. **Sulfur-containing linkers** in ILs **5** (dibenzothiophene) and **7** (thioxanthone) consistently show elevated *T*_m_ values. The **4**/**5** comparison (oxygen vs sulfur linker) revealed an entropically driven increase, while the **6**/**7** difference was enthalpy-dominated. Sulfur’s larger size and greater polarizability promotes stronger intermolecular interactions through enhanced van der Waals forces, π-π stacking, and sulfur–hydrogen interactions. The different C–S and C–O bond lengths further break molecular symmetry, influencing thermal behavior—a phenomenon we previously exploited to reduce the *T*_m_ of lipid-like ILs.^36,45^ IL **7** exhibited dramatic increases in both Δ*H* ^fus^ (177%) and Δ*S* ^fus^ (183%) relative to IL **5**, with the larger enthalpy increase resulting in a substantial melting point elevation (Δ*T*_m_ = 68.4 °C).
4. **Electronic effects** of the linkers become apparent when comparing ILs **10** (diphenyl ether) and **11** (diphenyl sulfone). IL **11** exhibits a significantly higher melting point than IL **10** (Δ*T*_m_ = 48.1 °C), with this increase being predominantly enthalpy-driven. In IL **10**, the electron-donating *p*-phenoxy groups enable charge delocalization across the molecular framework, reducing surface charge density and leading to a lower *T*_m_. Conversely, the electron-withdrawing sulfonyl group in IL **11** creates a more localized charge distribution, resulting in stronger intermolecular interactions and a higher *T*_m_.
5. **Dicationic systems** exhibited distinct patterns compared to their monocationic counterparts, with substantially elevated melting points. ILs **14** (dibenzofuran) and **15** (diphenyl sulfone) showed a modest Δ*T*_m_ of 16.2 °C, with IL **15** showing a higher melting point accompanied by increases in both Δ*H* ^fus^ and Δ*S* ^fus^ (Table 2). This behavior stems primarily from the sulfone group’s higher polarity compared to the ether group, creating stronger intermolecular interactions—similar to the trend observed between ILs **10** and **11** (vide supra). More dramatic changes appear when comparing IL **13** with its monocationic analogue (**2**): Δ*H* ^fus^ increases substantially (119%) due to stronger Coulombic forces, while Δ*S* ^fus^ decreases (63%) from increased cation symmetry, collectively raising the *T*_m_ by 87.2 °C.

In short, these structure–property relationships reveal how molecular architecture governs the thermal behavior in these ILs, with melting behavior influenced by a complex interplay of molecular symmetry, charge distribution, H-bonding capability, and electronic effects. Our systematic analysis reveals significant variations in both enthalpic and entropic contributions across different structural motifs, highlighting the complexity of *T*_m_ prediction. To elucidate the underlying intermolecular interactions responsible for these trends, we next conducted a crystallographic analysis of these materials.

### Crystallographic Characterization and Structural Studies

Our crystallographic analysis provides molecular-level explanations for the thermophysical trends observed in our DSC studies. While Coulombic forces dominate lattice enthalpy,^46,47^ the supramolecular interactions significantly influence melting behavior. A more detailed discussion of these interactions, visualized with interaction fingerprints derived from Hirshfeld surface analysis, is provided in the Supporting Information.

**Figure 5:**
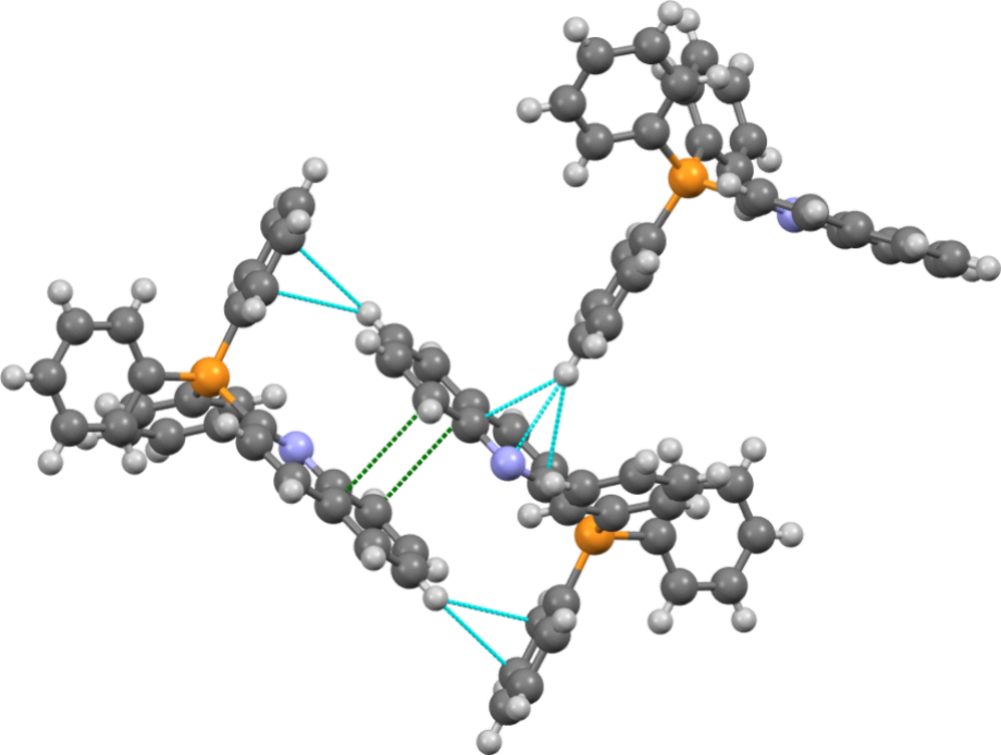
Illustration of cation–cation interactions in IL **1**: π–π stacking (green) and π–H interactions (blue) are depicted, occurring at distances shorter than the sum of the van der Waals radii of the respective atoms. Anions are omitted for clarity.

**Figure 6:**
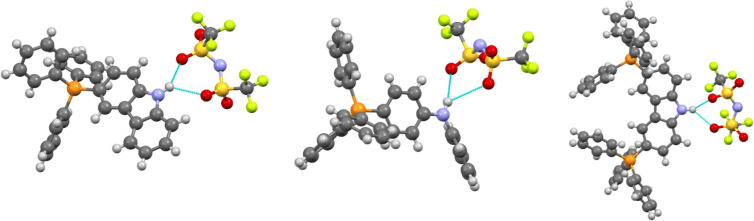
Depiction of the cyclical hydrogen bonding interactions present in molecules containing N–H groups in ILs **2**, **8**, and **13**. Both the *cis* and TS1 conformers of the anions are observed to make cyclical R_1_^2^(6) H-bonding motifs.

**Figure 7:**
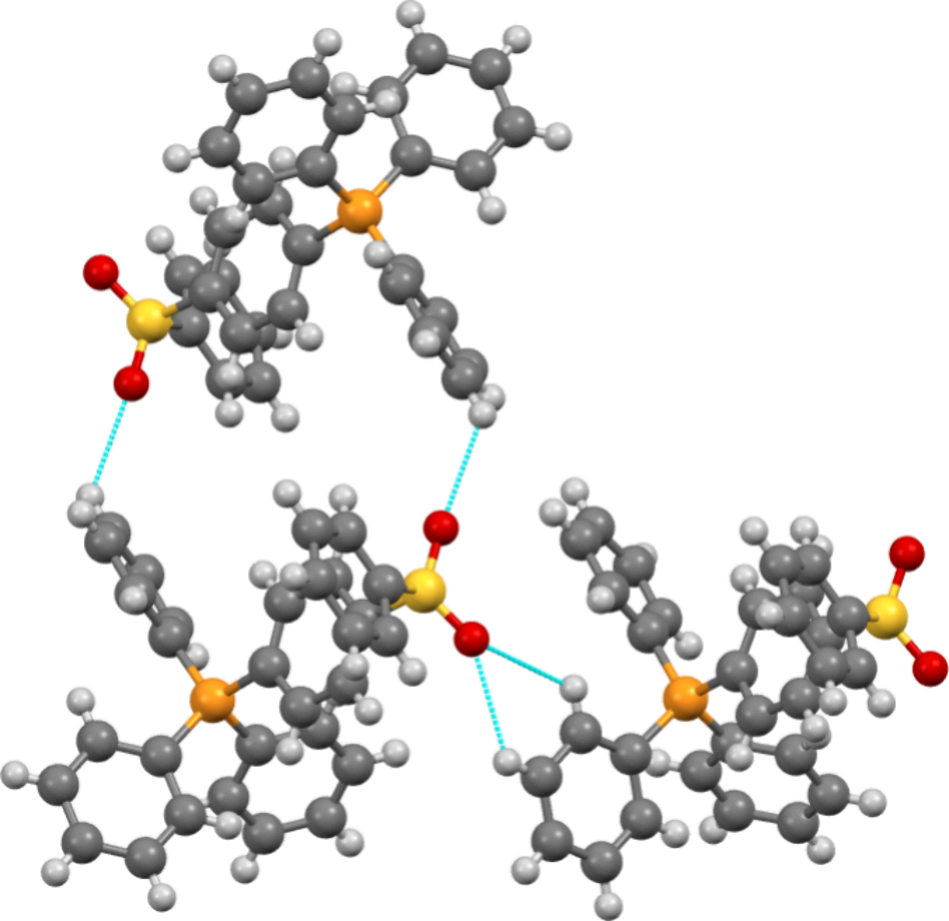
Illustration of sulfonyl group interactions with aromatic hydrogen atoms in IL **11**. The sulfonyl group’s tetrahedral geometry creates a network where multiple cations connect through O···H interactions. These interactions occur exclusively with the aromatic hydrogens on the triphenylphosphonium-based cation. Anions are not shown for the sake of clarity.

**Figure 8:**
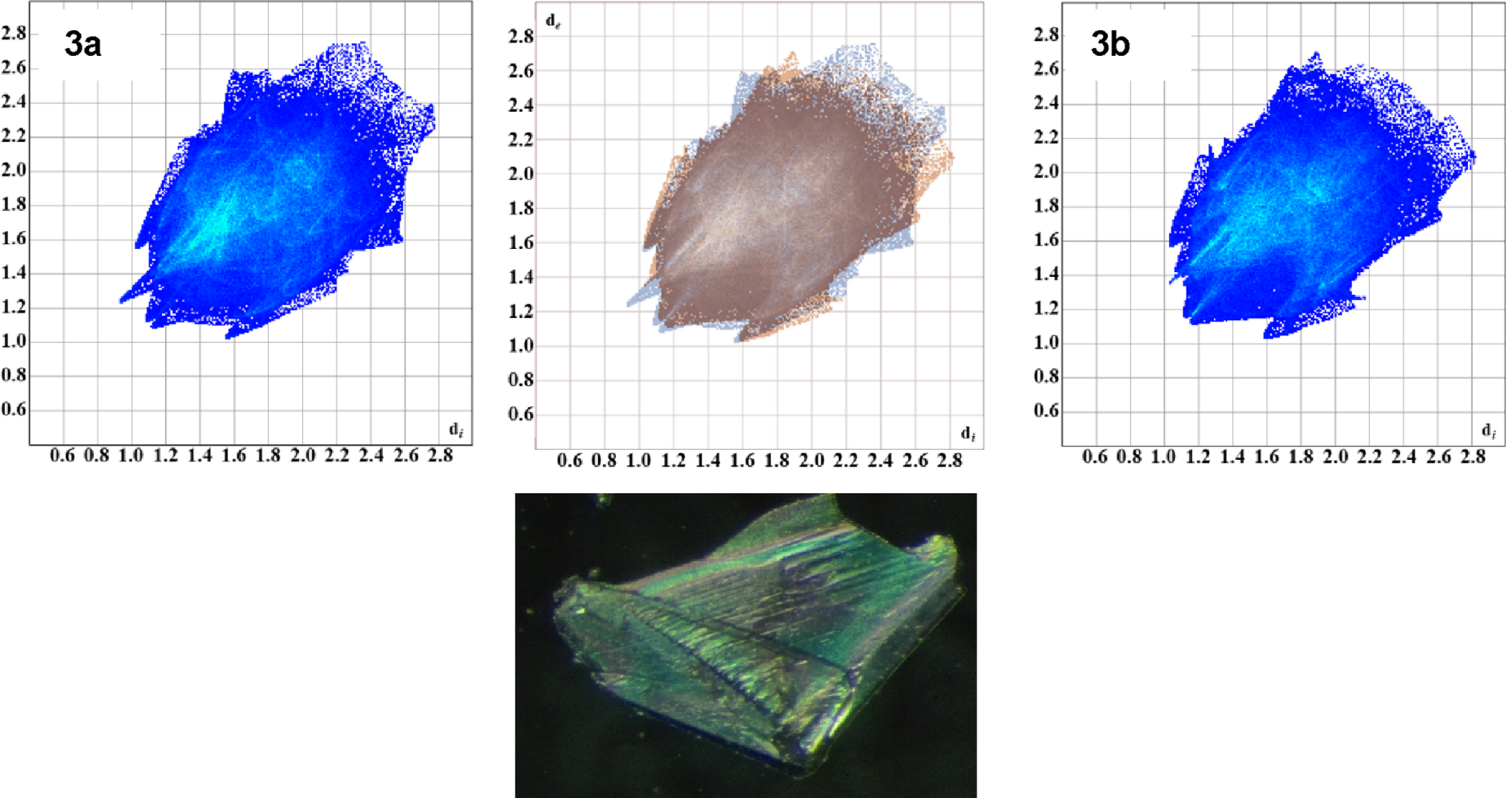
Top: depiction of the interaction fingerprints for **3a** and **3b**. The top middle image is an overlap of the two fingerprints helping to show similarities and differences in the two polymorphs. Bottom: the single-crystal image of the intergrowth crystals of **3** was examined using a polarized microscope.

The X-ray analysis revealed key patterns:

1. **Cation–cation interactions** play a significant role in determining thermal properties. IL **1** (quinoline) has a melting point ∼10 °C lower than [Ph_4_P]­[NTf_2_]. The planar quinoline structure enables molecular interactions absent in [Ph_4_P]­[NTf_2_] (Figure 5). The planar quinoline fragment participates in parallel offset π-stacking interactions along one face with a symmetry adjacent quinoline unit, while the opposite face engages in multiple π···H interactions with aromatic hydrogens from the P-center. The quinoline aromatic hydrogens primarily form interactions with the anions. This creates a set of interactions where the quinoline faces allow for cation–cation interactions through π-stacking and π···H contacts, while the quinoline hydrogens facilitate cation–anion interactions through connections to both fluorine and oxygen atoms of the anion. The increased cation–cation interactions,^48^ along with a more asymmetric cation, explain the lower *T*_m_ despite higher molecular weight.
2. **H-Bonding patterns** significantly influence IL properties.^49^ ILs **2**, **13** (carbazole), and **8** (diphenylamine) (containing N–H groups) form cyclical R_1_^2^(6) H bonds^50^ with the [NTf_2_]^−^ anion’s sulfonyl oxygens (Figure 6). The anion conformation plays a crucial role in enabling these interactions. IL **8** contains rotationally disordered anions in both *trans* and less common TS1 conformations,^51^ with only the TS1 conformation forming the cyclical H-bonding motif. Conversely, in IL **2**, the *cis* conformation readily forms these ringed H bonds. These conformation-dependent interactions likely optimize orbital overlap between anions and cations.^51^ In contrast, IL **9-NTf**_**2**_ lacks H-bond donor capability due to its triphenylamine structure, resulting in weaker cation–anion interactions and the absence of a distinct melting transition. While IL **9-PF**_**6**_ forms a crystalline solid with a defined phase transition, **9-NTf**_**2**_ differs critically by one additional phenyl ring around the nitrogen, eliminating the amine’s H-bonding ability. The sterically demanding, nonplanar geometry of triphenylamine also creates a pocket between Ph_3_P^+^ and Ph_3_N moieties that prevents larger anions from approaching the positively charged P-center^52,53^ —the sulfonyl groups cannot geometrically access this site due to the cation’s phenyl ring arrangement, weakening cation–anion interactions and reducing crystallinity. Only smaller anions (i.e., [PF_6_]^−^) can access this pocket effectively.
3. **Oxygen-containing functional groups** influence IL properties primarily through electronic effects rather than by forming specific interactions. This pattern appears across multiple structural variations: ether moieties in ILs **4**, **6**, and **14** form O···H–C contacts that appear incidental to crystal packing rather than structure-directing based on distance and angles of the interactions. Similarly, IL **10** (diphenyl ether) shows O···H interactions that are likely consequences of packing arrangements rather than directing forces. Furthermore, multiple interactions with the anions (O···O and O···F) are also observed. Carbonyl-containing ILs (**6** and **7**) show selective C–H···O contacts with adjacent Ph_3_P^+^ rings. This selectivity stems from competing π–π stacking arrangements and the preferential direction of carbonyl oxygen’s electron density toward the P-center for stabilizing contacts.
4. **Sulfonyl-containing ILs** (**11** and **15**) display complex interaction networks distinct from other *O*-containing ILs, with their tetrahedral geometry enabling diverse interaction motifs compared to planar carbonyl groups. These compounds display a complex network of H···O_SO_2__ interactions exclusively with aromatic hydrogens on the Ph_3_P^+^ rings (Figure 7). This tetrahedral arrangement contrasts with the geometrically constrained planar carbonyl groups, which offer fewer interaction possibilities. These diverse interaction patterns create extended networks where cations surround anions, forming continuous channels throughout the crystal lattice. The collective analysis of *O*-containing compounds (ether, carbonyl, sulfonyl) reveals two key structural heuristics: (i) oxygen-containing groups influence crystal packing primarily through electronic effects rather than through specific hydrogen interactions,^54^ and (ii) the geometry of the oxygen-containing group (planar vs tetrahedral) significantly affects its interaction potential.
5. **Polymorphism**, observed in IL **3**, demonstrates how subtle differences in anion orientation can create distinct crystalline forms while maintaining similar interaction patterns (Figure 8 and Figure S16). The polymorphs differ primarily in the orientation of their [NTf_2_]^−^ anions—in **3a**, the central imide nitrogen points away from the cation, while in **3b** it points toward the cation. Hirshfeld surface analysis shows remarkably consistent interaction patterns between the two forms, with fluorine interactions dominating in both.

The interaction fingerprints (Figure 8) illuminate both similarities and differences between the polymorphs. In both forms, fluorine and oxygen atoms of the anion form multiple short contacts with aromatic hydrogens across the Ph_3_P, carbazole, and phenyl linker regions. **3a** exhibits its shortest H···O contact with a hydrogen on the phenyl linker, while **3b** features a slightly longer H···O distance but compensates with a shorter H···F interaction. This suggests that the hydrogen interactions are energetically similar, allowing the anion to form numerous nondirectional contacts rather than optimizing a single directional hydrogen bond. This nonspecificity likely provides an entropic advantage by maximizing overall interactions throughout the structure. However, despite these multiple, degenerate close interactions, long-range electrostatics, and repulsions are impacted by the changes in the anion geometry. A discussion of the differences in interaction energies and visualized crystal frameworks of the polymorphs is provided in the Supporting Information in detail (Figures S6, S17, and S18).

To summarize our crystallographic analysis, the inclusion of the distinctive aryl moieties leads to the formation of π interactions with multiple cations. These, in part, account for the lower melting points we observed in several compounds, despite having high molecular weights. Concomitantly, we noted that specific functional groups (e.g., sulfone groups) displayed the preferential formation of bonds with aromatic hydrogens on the cationic PPh_3_ moiety, as opposed to the aromatic hydrogens on the extended π groups. We provide additional commentary in the Supporting Information to expand upon these discussions.

### Photophysical Properties

The triphenylphosphonium (TPP) moiety itself does not exhibit strong intrinsic fluorescence and is therefore not widely used as a core luminophore in mainstream applications. However, it is frequently conjugated to a variety of fluorescent molecules—such as BODIPY, rhodamine, and coumarin derivatives—or employed as a carrier or anchor in fluorescent probes. TPP is well-known for its ability to target and accumulate in mitochondria due to its positive charge and lipophilicity, which facilitate its transport across lipid membranes and accumulation in the negatively charged inner mitochondrial membrane. This property has made TPP-conjugated probes valuable for visualizing mitochondrial (dys)­function and dynamics in live cells.^55,56^ Mitochondrial localization also enables the design of “mitocans”—anticancer agents that target tumor cell mitochondria and induce apoptosis via local release of apoptotic factors^57^ —as well as mitochondria-targeted photodynamic therapy agents.^58^ In addition, the TPP cation provides high stability in biological media and low reactivity with cellular components, helping to minimize off-target effects. TPP-based fluorogens have also been used to monitor protein aggregation by detecting aggregation-induced changes in fluorescence intensity or polarization—useful for monitoring the early stage formation of amyloid fibrils and related pathological processes.^59^

The absorbance spectra, emission maxima, and quantum yields of fluorescence were measured for each of the triphenylphosphonium-based salts shown in Figure 9. The electronic absorption spectra of salts **1**–**15** dissolved in dilute ethanolic solution are summarized in Figure S22. The absorption maxima are collected in Table S4 of the Supporting Information. Three key points emerge from these data. First, for most compounds, the band maxima fall between 227 and 279 nm. Only compounds **3**, **4**, **6**, and **9** show substantial peaks past 330 nm. Second, the UV–vis absorption profiles of the bromide analogs of **2**, **6**, and **9** in ethanol closely match those of their [NTf_2_]^−^ counterparts but exhibit higher molar absorptivity. The degree of hyperchromism is substantial, ranging from 23 to 66%. For instance, as illustrated in Figure 9, the [Br]^−^ and [NTf_2_]^−^ salts of **2** share identical spectral shapes, with a UV–vis band maximum at 279 nm. However, the molar extinction coefficient at this peak is 48% higher for the bromide salt. The molar extinction coefficient typically decreases as the UV band shifts to longer wavelengths.

**Figure 9:**
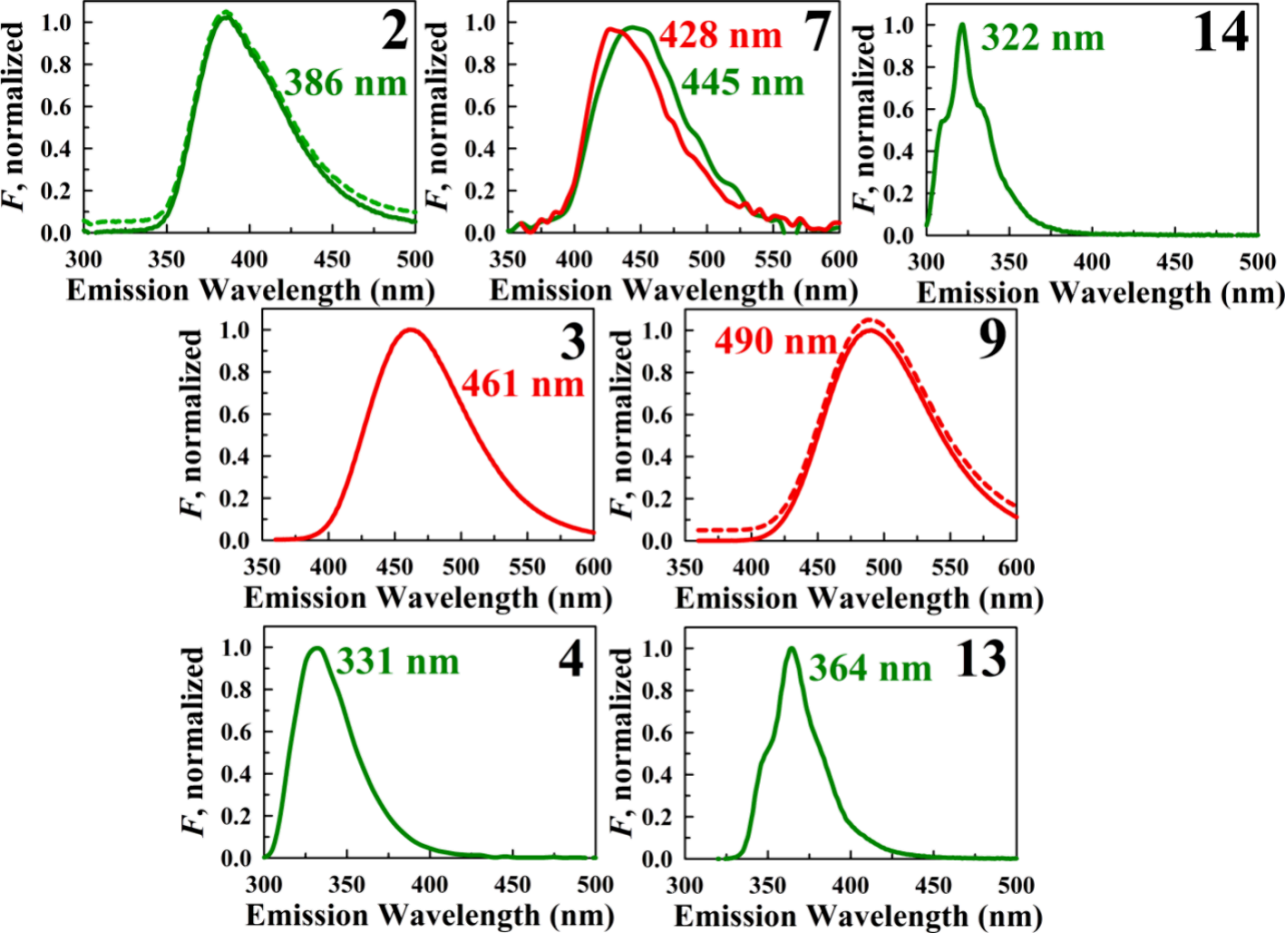
Normalized steady-state fluorescence emission profiles of the subset of fluorescent triphenylphosphonium-bearing salts measured in a dilute ethanolic solution. Unless otherwise specified, the data correspond to the [NTf_2_]^−^ salts. Dashed profiles represent the bromide salts for samples **2** and **9**, shown alongside their [NTf_2_]^−^ counterparts. For the sake of clarity, these spectra are vertically shifted. Fluorescence maxima are indicated by the provided wavelengths. The green profiles represent excitation at 280 nm, while the red spectra correspond to excitation at 350 nm.

The fluorescence characteristics (emission maxima and quantum yields) of phosphonium salts **1**–**15** are summarized in Figure S22 and Table S5. It should be stressed that these measurements were conducted for the salts dissolved in ethanol at “infinite dilution” (low micromolar concentration). Under certain conditions (e.g., ground-state excimer formation), luminescence might be observed in the solid state for specific compounds. Several noteworthy points of these results merit attention: (i) The triphenylphosphonium moiety alone does not facilitate the formation of fluorescent salts. In fact, of the 15 ILs, 9 were essentially nonemissive. The fluorescence properties are primarily governed by the choice of linkage in these salts, as discussed below. (ii) Some linkages, particularly diphenylamine (**8**), diphenyl ether (**10**), and diphenyl sulfonyl (**11**), are not expected to act as competent fluorophores due to structural considerations—specifically, the presence of floppy, poorly conjugated units—and supporting literature precedents. As anticipated, these compounds show negligible emission. However, the cyclized analogs of **8** and **10**, carbazole (**2**) and dibenzofuran (**4**), respectively, do exhibit emission: weak violet fluorescence is observed for **2** (386 nm maximum, QY ≈ 3%) and **4** yields somewhat stronger deep violet emission at 331 nm (8% QY). This underscores the role of rigidification, a well-known strategy for enhancing the fluorescence of organic fluorophores, where restricting the motion of the fluorophore typically reduces nonradiative decay pathways, such as vibrational relaxation and internal conversion. A classic example of this phenomenon is the comparison between fluorescein and phenolphthalein,^60^ which share a similar molecular structure. However, the pH-indicator dye phenolphthalein lacks the oxygen bridge found in fluorescein, a feature that induces molecular rigidity and enhances its photoluminescence.^60^ (iii) A comparison of the bis­(trifluoromethylsulfonyl)­imide and bromide forms of **2** and **9** reveals that, while their spectral profiles are identical, the [NTf_2_]^−^ salts exhibit a slight increase in quantum yield, by 6.4 and 8.0%, respectively. Due to the heavy-atom effect, bromide can act as a weak quencher of fluorescence, participating in both heavy-atom-induced intersystem crossing and collisional (dynamic) quenching. Although bromide is a weaker quencher than iodide, it still exhibits some quenching ability,^61^ which may account for the slight enhancement in quantum yield observed when bromide is replaced by [NTf_2_]^−^, a less interactive counterion. (iv) Replacing the carbazole functionality in **2** with a phenylcarbazole moiety in **3** significantly enhances the emission, shifting from a weak violet emission (386 nm, 3% QY) to strong blue emission (25% QY) with a band maximum at 461 nm. (v) Bridged bis­(phosphonium) salts of the type [Ph_3_P–X–PPh_3_]^2+^ exhibit stronger fluorescence compared to their monocationic counterparts. For instance, the carbazole bridged dicationic salt **13** displays a sharper, blue-shifted (by 22 nm) UV emission with more than double the relative intensity of the moncationic variant **2** (7.4% vs 3.3% QY). Similarly, the dicationic dibenzofuran salt **14** is 3.4 times more emissive than its monocationic counterpart **4** (27.4% vs 8.0% QY), with a 9 nm blue shift and more structured emission profile in the near-UV (322 nm maximum). In both cases, the increased rigidity likely contributes to the enhanced fluorescence properties, suggesting that such bridged dicationic fluorophores are promising candidates for further study.

In contrast, the bridged diphenylsulfonyl dication **15**, as well as its monocationic analog **11**, showed no fluorescence. (vi) *N*-Heterocycles, such as quinolines, are generally weakly fluorescent compared to their isoelectronic hydrocarbon counterparts, and the quinoline salt **1** was indeed found to be nonfluorescent. Similarly, the tetraphenylsilane salt **12** showed no fluorescence, despite this moiety being commonly used as a building block in host materials for phosphorescent organic light-emitting diodes (OLEDs), where it plays a role in confining excited triplet excitons within the emitting molecule. (vii) A comparison of triphenylphosphonium analogs bearing dibenzothiophene (**5**), xanthone (**6**), and thioxanthone (**7**) units reveals that compounds **5** and **6** are nonfluorescent, while compound **7** exhibits weak emission (∼3% QY). Xanthone is known to undergo ultrafast (∼1 ps) intersystem crossing upon photoexcitation, resulting in a vanishingly small QY (∼10^–4^) in most solvents. In contrast, the QY of thioxanthone in hydroxylic solvents has been shown to increase with solvent polarity.^62^ (viii) Finally, the triphenylamine-bearing salt **9** is the most fluorescent compound among those studied, with the [NTf_2_]^−^ form exhibiting a QY of 42% in the blue-green region (490 nm fluorescence maximum). Although one might expect pH-dependent behavior for **9**, the addition of an acid or base to an ethanolic solution of **9** produced no noticeable changes in absorbance or fluorescence. This is attributed to the electron-withdrawing effect of the three aromatic groups attached to the central nitrogen, which delocalize the nitrogen lone pair and impart a partial positive charge to the nitrogen.^63^ As a result, nitrogen protonation is prevented, making the propeller-shaped triphenylamine unit nonbasic, unlike most amines. The promising fluorescence properties of **9**, along with the fact that triphenylamine derivatives are effective hole transporters in OLEDs, suggest a promising avenue for developing next-generation functional salts and ionic liquids based on this chemistry. Overall, the structure–function insights gathered here highlight potential pathways for developing novel and enhanced phosphonium salts as sensory dyes, as well as components in light-emitting diodes and organic photovoltaics.^64^ In addition, the supra-ambient melting points of the parent salts suggest that these π-conjugated phosphoniums may be suitable for preparing nanoscale assemblies of uniform materials based on organic salts (nanoGUMBOS), consistent with prior reports of nanoGUMBOS derived from high-melting salts comprising cationic dyes, including cyanine, pseudoisocyanine, and thiacarbocyanine fluorescent dyes.^65–68^

## Conclusions

The systematic study of organophosphorus salts with extended π-conjugated cations has yielded important insights into the complex relationships among their molecular structures, intermolecular interactions, and thermophysical properties. By employing an integrated approach combining crystallographic analysis and thermal characterization, we have established several key principles for molecular engineering of thermally stable IL materials with tunable fluidity.

Our comprehensive thermal stability assessment examined 15 tetraarylphosphonium salts paired with the [NTf_2_]^−^ anion. Mass loss measurements and detailed NMR spectroscopic analysis confirmed the exceptional thermal stability of these materials. The majority of these materials exhibited negligible mass loss or structural changes even after 96 h of exposure to 300 °C under aerobic conditions. The inherently nonvolatile character of these compounds coupled with their exceptional thermal resilience provides a significant advantage over conventional heat transfer fluids and a meaningful advancement in the development of thermally robust materials.

Comprehensive crystallographic studies directly inform the thermophysical trends observed in our DSC analysis, providing molecular-level explanations for the thermal behaviors of these ILs. A key discovery is that cation–cation interactions exert more significant influence on melting behavior than those previously recognized in phosphonium-based systems. The strategic incorporation of aryl groups into the tetraphenylphosphonium core modifies these interactions through two mechanisms: (1) altered molecular packing and lattice spacing due to increased cation size and (2) modulated interaction strength and directionality through the electronic effects of different linking groups (ether, carbonyl, or sulfonyl) between phenyl rings.

This work provides key insights into how the molecular engineering of these functional ILs determines their properties. Through crystallographic and thermal analyses, we established a systematic approach for rationally designing organic-ion materials with exceptionally high-temperature stability. These findings enable the development of materials for extreme-temperature applications, particularly as heat transfer fluids, phase changing materials, and nuclear reactor coolants. Furthermore, their tailorable photophysical properties make these materials promising candidates for use in sensory dyes, light-emitting diodes, and organic photovoltaics.

## Supplementary Material





## Notes

^1^H, ^13^C, and ^31^P NMR spectra; DSC and TGA thermograms; additional theoretical and crystallographic data and discussion; images of the crystals, photophysical data; and summary of an unsuccessful synthetic attempt (PDF)
Crystallographic data (ZIP)

## Notes

This article is dedicated to the memory of my father, Professor Hossein Mirjafari (1940–2024), a dedicated scholar and compassionate mentor who shaped countless minds, including my own.
