# Supplementary material for: Property-Driven Design of Thermally Robust Organophosphorus Ionic Liquids for High-Temperature Applications
Source: ACS Appl Eng Mater. 2025 May 5;3(5):1468–82. doi: 10.1021/acsaenm.5c00221 (PMC12117501; doi:10.1021/acsaenm.5c00221)
Supplement: Supplementary file 1 [file em5c00221_si_001.pdf]

SUPPORTING INFORMATION

Property-Driven Design of Thermally Robust Organophosphorus Ionic Liquids for High-Temperature Applications

Muhammadiqboli Musozoda,[a] Andrew L. Bishuk,[a] Blake J. Britton,[a] Marija Scheuren,[b] Charles H. Laber,[c,d] Gary A. Baker,[d] Matthew S. Baker,[a] Matthias Zeller,[e] Daniel H. Paull,[f] Patrick C. Hillesheim,*,[b,g] Arsalan Mirjafari*,[a]

[a] Department of Chemistry, State University of New York at Oswego, Oswego, New York 13126, United States

[b] Department of Chemistry and Physics, Ave Maria University, Ave Maria, Florida 34142, United States

[c] Department of Chemistry, University of Missouri, Columbia, Missouri 65211, United States

[d] U.S. Army Engineer Research and Development Center, Vicksburg, Mississippi, 39180, United States

[e] Department of Chemistry, Purdue University, West Lafayette, Indiana 47907, United States

[f] Department of Chemistry and Physics, Florida Gulf Coast University, Fort Myers, Florida 33965, United States

[g] Department of Chemistry, Illinois State University, Normal, Illinois 61761, United States

Corresponding Authors:

Emails: arsalan.mirjafari@oswego.edu (A.M.), patrick.hillesheim@avemaria.edu (P.C.H.)

Table of Contents

Unsuccessful Pd(II)-Catalyzed Approach……….……………………………………………..........S3

Kinetic Studies.……….…………………………………………...S3

Crystallographic Data and Discussion..…………………………………………………………S7

Crystal Structures and Crystal Systems…………………….…………………………………..S7

Polymorphs..…………………………………………………………………......................S8

Supramolecular Interactions……………………………………………………………………S11

General Comments and Trends in Supramolecular Interactions………………………......S11

H-Bonding Rings…………..……………………………………………………..………S13

π Interactions..………………………………………………………………...………......S14

H⋯C | C⋯H interactions………………………………………………………………S15

C⋯C Interactions. …………………………………………………………….….. .S16

$[PF_6]^-$ vs. $[NTf_2]^-$ Anions. ……………………………………………………………......S18

Sterics of Triphenyl Moieties…………………………………………..………….......S19

Polymorphism…..…………………………………………………………..............................S19

Conclusion………………………………………………………………..............................S21

Crystal Data and Structure Refinement for the IL Products………………………………………S23

Additional Refinement Details……….………………………………………………………S28

Crystal Images of the Representative IL products………………….……………….……….....S31

Photophysical Experiments……………………..…………………….…………….………......S38

References………………………………………………………………………………………..S41

^{1}H, ^{13}C and ^{31}P NMR Spectra of the Products…..…..……………………………………..........S43

DSC and TGA Thermograms of the Products..………………………………………….........S73

EXPERIMENTAL PROCEDURES AND CHARACTERIZATION DATA

Unsuccessful Pd(II)-Catalyzed Approach

A dry round-bottomed flask equipped with a magnetic stirrer was charged with the heterocyclic scaffold (**a**, 1.0 equiv.), triphenylphosphine (**b**, 1.1 equiv.), and palladium(II) trifluoroacetate (0.01 equiv.). After evacuating and backfilling with nitrogen degassed xylenes were added. The mixture was heated to reflux overnight under nitrogen. This procedure was adapted from a previous report where product precipitation from non-polar solvents facilitates the formation and isolation of IL products.[1–3] After cooling to room temperature, the bromide salts precipitated. The precipitate was collected by filtration and washed with fresh xylenes (3 × 20 mL) to afford white solids. *Note 1:* Yields were consistently low (< 10%) for π-extended heterocycles containing oxygen and sulfur linkages. *Note 2:* The major product, identified as $[Ph_4P][NTf_2]$ and characterized by single-crystal X-ray diffraction, formed during the coupling reaction between Ph_3P and 4-bromotriphenylamine. This product likely results from oxidative addition of Pd(0) into the phosphonium–phenyl bond, followed by reductive elimination with triphenylphosphine to yield $[Ph_4P][NTf_2]$.[4]

Kinetic Studies

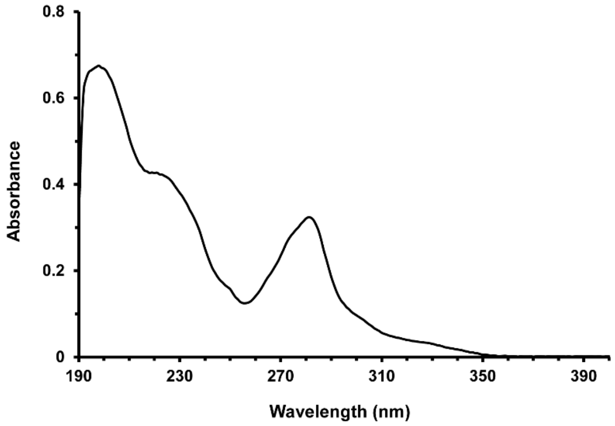

Figure S1. UV-vis spectrum of the bromide salt of IL **2** (carbazole).

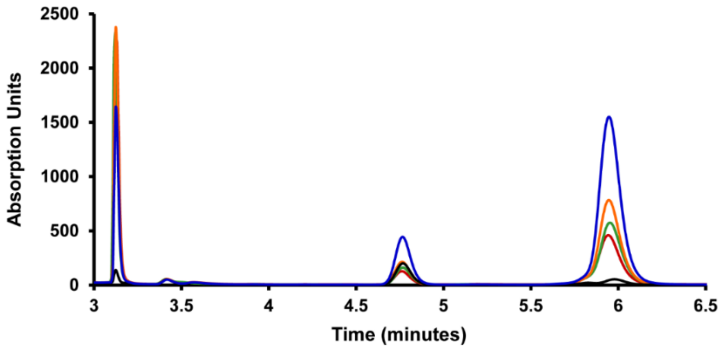

Figure S2. HPLC traces monitoring the production of the bromide salt of IL **2** over a 60-minute window. The signals at 3.2, 4.8, and 5.9 represent the product (IL **2**), 3-bromocarbazole, and Ph_3P, respectively. Traces are provided at 0 minutes (black), 15 minutes (blue), 30 minutes (orange), 45 minutes (green), and 60 minutes (red).

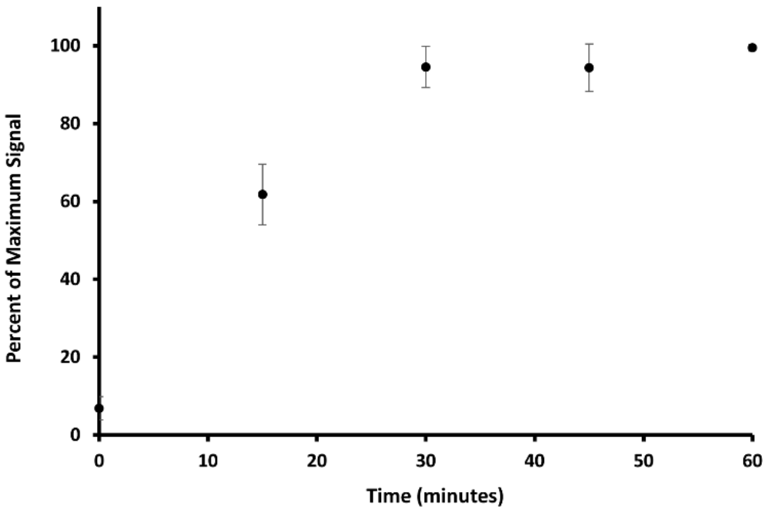

Figure S3. Rate of the bromide salt of IL **2** production resulting from the above HPLC data. Each data point is the average of three data points from individual reactions, and the error bars represent the standard deviations from these averages. *Note:* The final data point lacks visible error bars as the three replicate measurements exhibited virtually identical values.

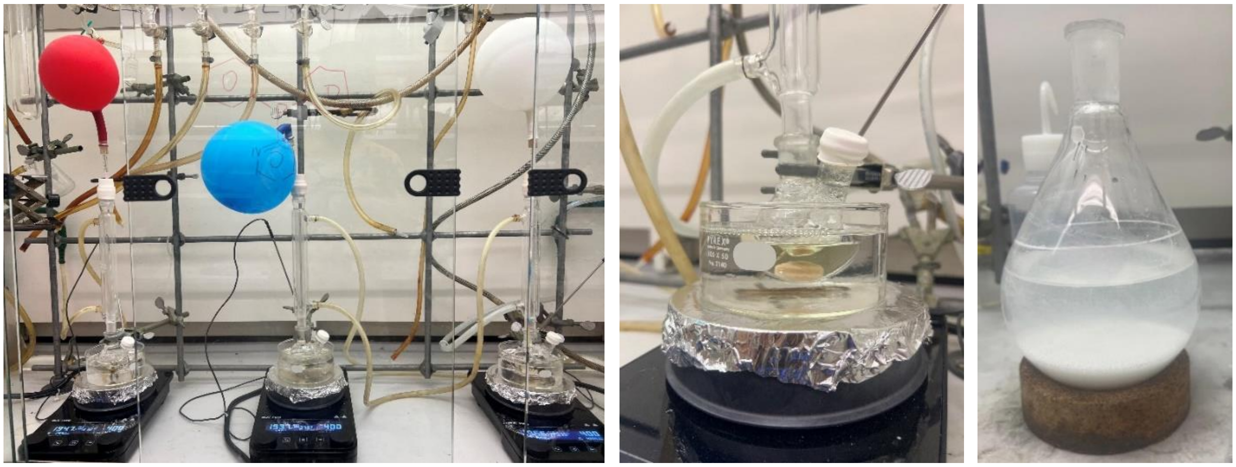

Figure S4. Reaction setup for the Ni(II)-catalyzed coupling reaction showing the kinetic studies and scalability of the process.

Before **After**

2

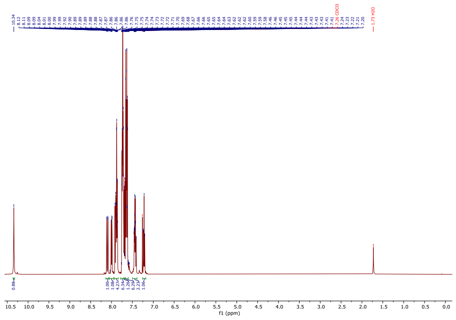

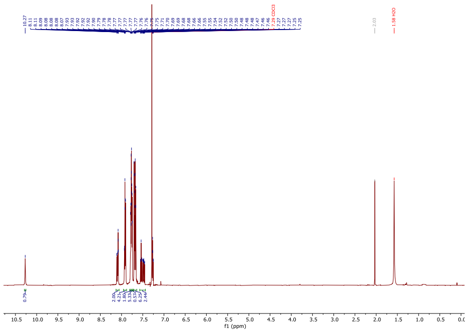

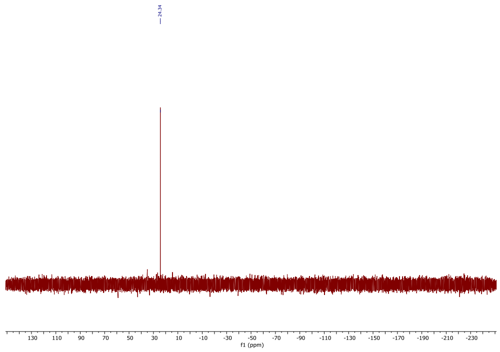

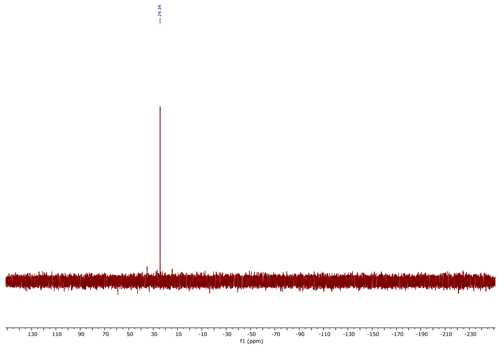

7

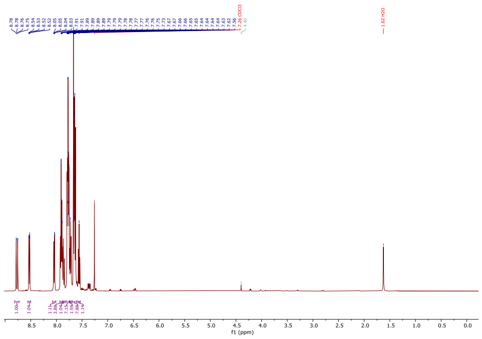

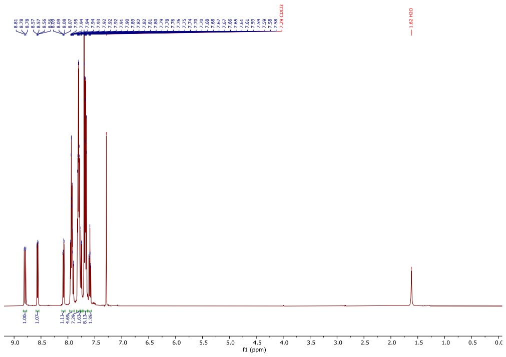

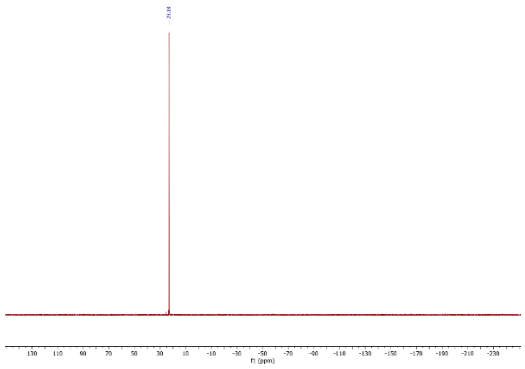

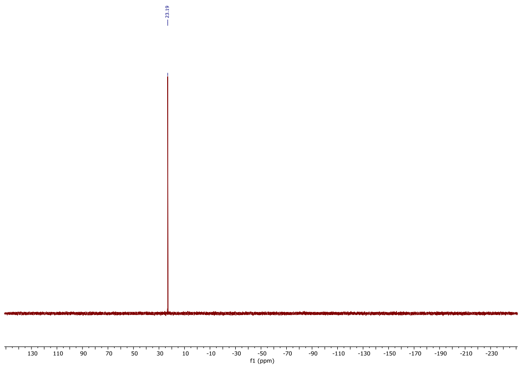

10

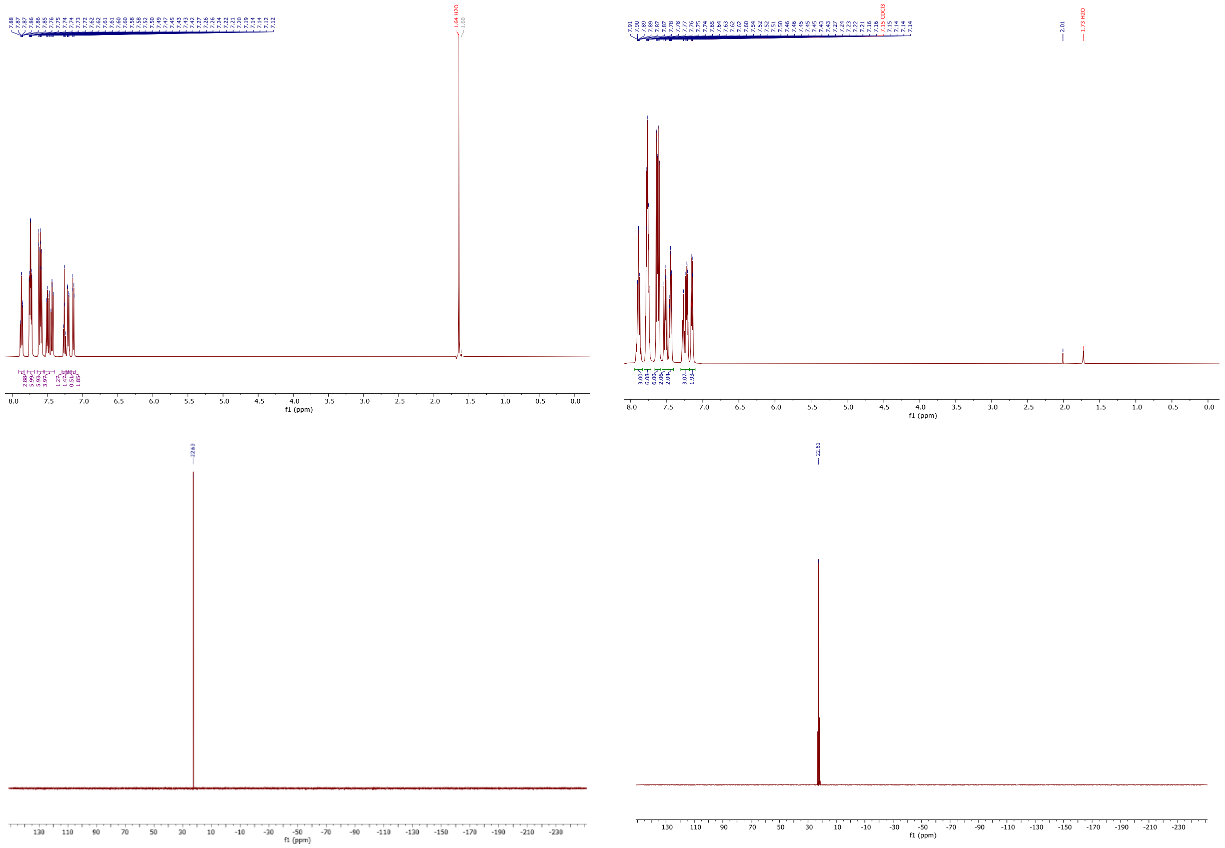

Figure S5. ^{1}H and ^{31}P NMR spectra of representative IL samples before (left) and after (right) long-term thermal exposure at 300°C for 96 hours in air.

Crystallographic Data and Discussion

Single crystal data of samples **1**, **2-NTf$_2$**, **2-PF$_6$**, **3b, 4–8**, **10–12** and **14** were collected on a Bruker Quest diffractometer with fixed chi geometry, a Mo Kα wavelength (λ = 1.54178 Å) sealed tube X-ray source and a curved graphite crystal for monochromatization and a Photon II area detector and an Oxford Cryosystems low temperature device. Data for **3a**, **3b**, **9-PF$_6$** and **13** were collected on a Bruker Quest diffractometer with kappa geometry, a Cu Kα wavelength (λ = 1.54178 Å) I-μ-S microsource X-ray tube, Montel optics for monochromatization, a Photon III area detector and an Oxford Cryosystems low temperature device. Examination and data collection were performed at 150 K. Data was collected, reflections were indexed and processed, and the files scaled and corrected for absorption using SADABS[5] and APEX4 or 5.[6] The space groups were assigned using XPREP within the SHELXTL suite of programs,[7] the structures were solved by dual methods using ShelXT[8] and refined by full matrix least squares against F^2 with all reflections with Shelxl2018 or 2019[9] using the graphical interfaces Shelxle.[10] If not specified otherwise H atoms were positioned geometrically and constrained to ride on their parent atoms. C–H bond distances were constrained to 0.95 Å for alkene C–H moieties, and to 0.99 and 0.98 Å for aliphatic CH_2 and CH_3 moieties, respectively. Methyl H atoms were allowed to rotate but not to tip to best fit the experimental electron density. Absolute structure factors were determined using Parson's method.[11]

Complete crystallographic data, in CIF format, was deposited with the Cambridge Crystallographic Data Centre. CCDC numbers 2426148, 2426157–2426159, 2426176–2426178, 2426381–2426390 containing the supplementary crystallographic data for this paper. These data can be obtained free of charge from The Cambridge Crystallographic Data Centre via www.ccdc.cam.ac.uk/data_request/cif.

Hirshfeld surfaces, their corresponding images, and fingerprint plots were calculated using *CrystalExplorer21*.[12]

1.1. Crystal Structures and Crystal Systems. Understanding the structures of ILs is crucial, especially in the context of novel cation design. Moreover, analyzing the supramolecular interactions within a series of ILs is essential not only for rationalizing their properties but also for guiding the design of future compounds, to draw out heuristic principles. Consequently, we crystallized the compounds to examine their structures and interactions. Compound **2** was crystallized with both $[NTf_2]^-$ and $[PF_6]^-$ counterions (see § 2.4), while **9** could only be crystallized as the $[PF_6]^-$ salt. In the case of **9**, a disordered solvent molecule was found in the unit cell but could not be properly modeled due to significant disorder. The solvent was removed using SQUEEZE,[13] a method for handling disordered moieties in crystallography, leaving a void within the crystal that precludes the calculation of the Hirshfeld surface for this molecule. We speculate that this was a chloroform moiety as the removed electron density, along with the shape of the density, most closely matches this molecule which was used in the growth of the crystal. However, the chloroform molecule could not adequately be modeled due to heavy disorder.

Several key details regarding the crystal structures should be noted. First, the most common crystal system was orthorhombic, with seven of the seventeen crystals displaying this lattice, followed by triclinic with six compounds. ILs **1**, **9**, and the two polymorphs of **3** display a monoclinic crystal system. Second, most compounds exhibit minimal cation disorder; only **9** and

15 show any disorder within the cations. In these cases, the disorder is confined to the extended aromatic moieties and does not involve the benzene rings of the Ph_3P core. In our previous work, we observed a lack of disorder in Ph_3P-based IL systems, especially concerning rotations of the phenyl rings.[14] This trend holds true for the tetra-aryl systems studied herein. However, several examples of anion disorder were observed, as is common with $[NTf_2]^-$ moieties. Notably, several compounds feature the less common TS1 conformation of the $[NTf_2]^-$ anion.[15]

1.2. Polymorphs. IL **3** crystallizes as a set of intergrowth polymorphs. Intergrowth polymorphism refers to the phenomenon in which a single compound crystallizes into distinct polymorphic forms within the same crystal, resulting in intergrown domains with a well-defined interface. To facilitate discussion, we will simply refer to the two distinct structures as **3a** and **3b** (Figure S6). An overlay is provided to help visualize the differences in the polymorphs. A packing diagram is also shown to help show the relationship between the two polymorphs (Figure S6). The main distinction between the two structures arises from the orientation of the anion. While both structures have a *cis* anion, when looking at the two crystal structures the anion is crystallized in what can be best described as either a rotation or inversion isomer. As discussed previously, there exist two energetically identical forms of the *cis* isomer of $[NTf_2]^-$.[16] Therefore, the two polymorphs provide a snapshot of the interactions arising from the two extrema of the conformational energy profile of the *cis* conformations of the anions. To draw a distinction in the crystals, then, **3a** has the central imide nitrogen (N_{NTf_2}) within the asymmetric unit oriented away from the cation, while in **3b** the N_{NTf_2} is pointing towards the cation.

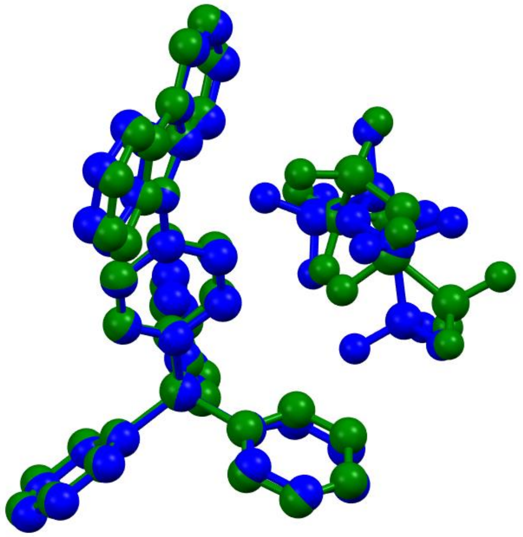

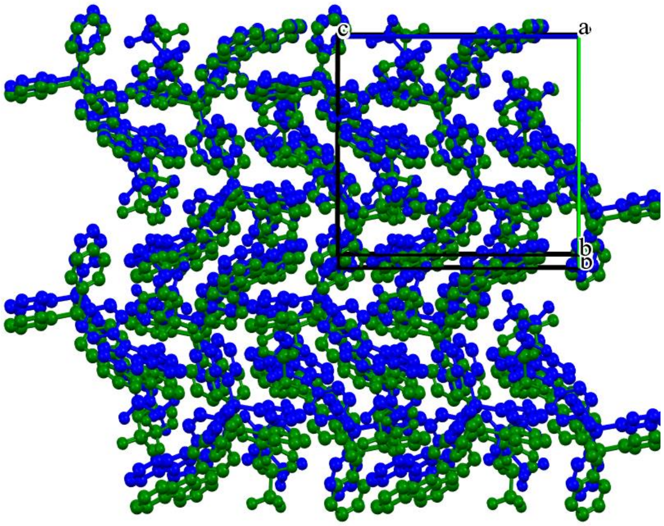

Figure S6. Left: Overlay (left) and Overlaid packing diagram (right) of **3a** (green) and **3b** (blue). Hydrogen atoms omitted for clarity.

To better understand the similarities and differences between **3a** and **3b**, Hirshfeld surface analysis was conducted for both anions in the polymorphs (Figure S7). Both anions display the same relative percentages of interactions, with the fluorine atoms accounting for the highest percentage for both, followed by oxygen, and then nitrogen. The nitrogen interactions, arising from the central N_{NTf_2} imide moiety, account for ~4% of the interactions, the majority of which are

H···N_{NTf_2} interactions with aromatic hydrogens. Despite the structural similarities of the crystals, the two anions are interacting with distinctive hydrogens. In **3a**, the shortest H···N_{NTf_2} interaction is with a hydrogen on the carbazole moiety at a distance of 2.700 Å (*d*(H···N)) while in **3b** the shortest interaction is with the linking phenyl ring at a distance of 2.777 Å (*d*(H···N)). From this simple observation one can conclude that, from an energetic perspective, these H···N interactions are likely similar in energy given the distance and identity of the atoms involved. That is to say, neither of the two aromatic hydrogens are likely too different with respect to charge (i.e., how positive each hydrogen is feeling).

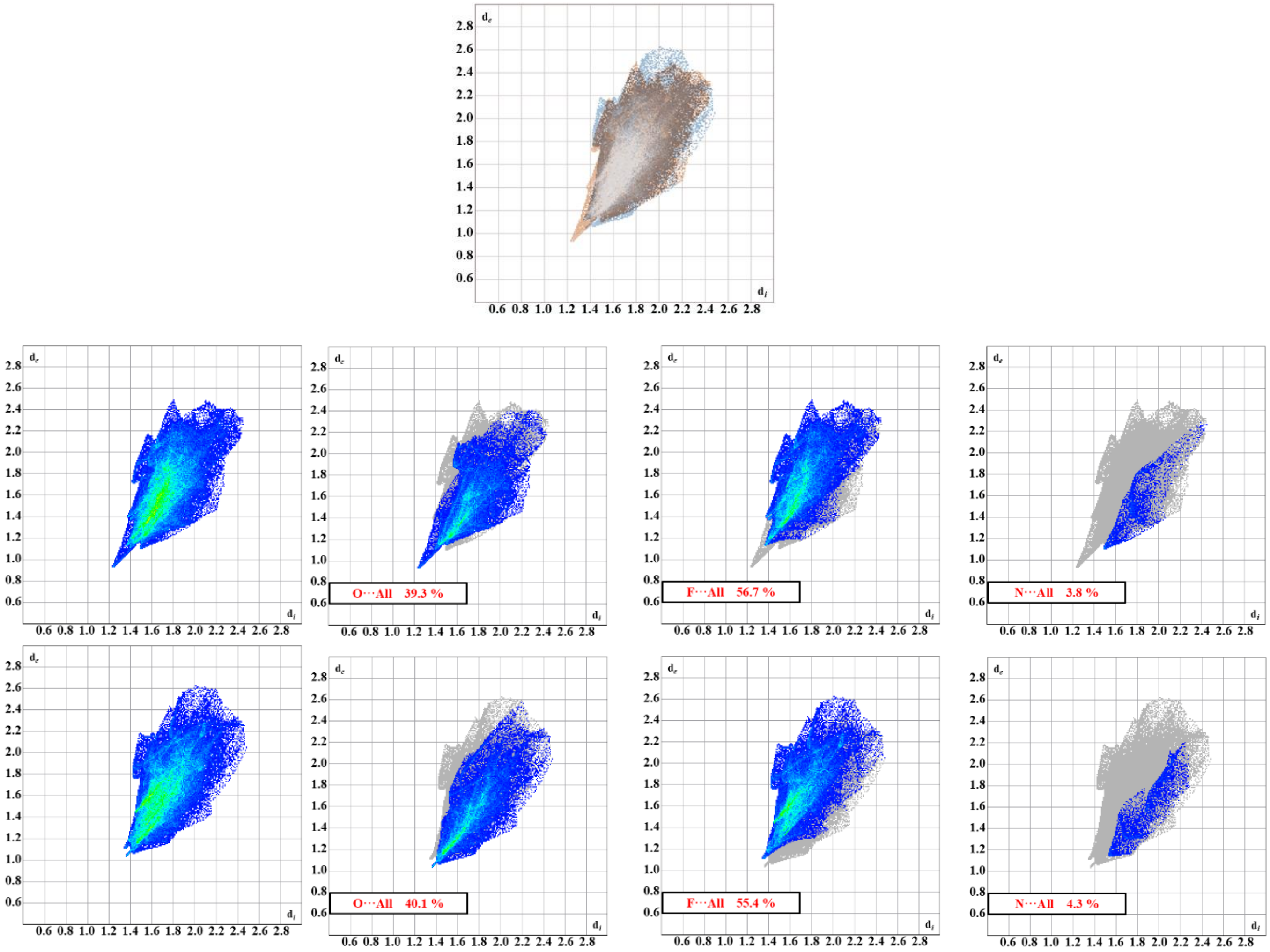

Figure S7. Interaction figure prints for the $[NTf_2]^-$ anions in **3a** and **3b**. The top figure shows an overlap of the individual fingerprints, clarifying the differences in shape and topology of the interactions.

Oxygen interactions vary in complexity, given the increased number of oxygen atoms involved and the geometry of the SO_2 moieties. In both cases, that is both anions in **3a** and **3b**, the sulfonyl oxygens (O_{SO_2}) form multiple interactions, linking multiple cations through complex H···O interactions (Figure S8). However, despite the complex nature of these supramolecular

interactions, careful inspection of the fingerprints allows us to distill useful information. First, the relative percentages of the O_{SO_2} interactions are practically the same for the two orientations (39.3% *vs.* 40.1%). Second, the sulfonyl oxygens interact with a variety of different aromatic hydrogens. Hydrogens on the Ph_3P, carbazole, and linker phenyl rings are all observed to form short contacts with these oxygen atoms. This behavior mirrors the interactions observed with the imide nitrogen, suggesting that the electronic characteristics of these aromatic hydrogens are similar and enable multiple, non-directional interactions between the cation and anion. Finally, as a point of distinction, **3a** has the shortest H⋯O interaction with an adjacent aromatic hydrogen on the phenyl linker (2.251 Å (*d*(H⋯O))). In **3b**, the shortest H⋯O interaction is with a carbazole hydrogen at a distance of 2.528 Å (*d*(H⋯O).

The fluorine atoms represent the largest percentage of interactions in both polymorphs with 56.7% for **3a** and 55.4% for **3b**. As with the oxygen atoms, interactions with hydrogens account for the bulk of these percentages. Likewise, the interactions are with varying aromatic hydrogens located on the various moieties within the cation (i.e., carbazole, linking phenyl ring, *etc.*). In contrast with the oxygen interactions, **3b** has the shortest H⋯F interaction with a linking phenyl hydrogen at a distance of 2.551 Å (*d*(H⋯F)). The shortest interaction in **3a** is with a hydrogen on the Ph_3P moiety at a distance of 2.599 Å (*d*(H⋯F)).

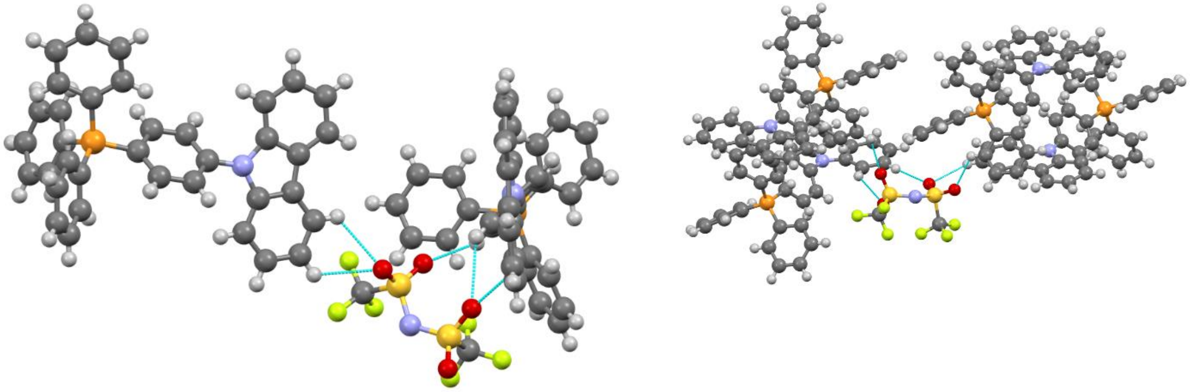

Figure S8. Depiction of H⋯O interactions in **3a** (left) and **3b** (right). Interactions are shown at less than or equal to the sum of the van der Waal radius of the atoms.

To draw some broad conclusions with the present data, changes in the orientation of the anions (e.g., the two *cis* conformations) reveal several important facts regarding the supramolecular interactions. First, the relative percentages of interactions remain constant: fluorine interactions are the most prevalent, while nitrogen interactions are the least common. Second, one orientation (**3a**) has marginally shorter H⋯O interactions and slightly longer H⋯F interactions than the other. Finally, the interactions overall appear indifferent with respect to the donor hydrogens. Simply, the anion is observed to make close contacts with nearly all distinct types of hydrogens on different portions of the cation. This would point towards an increase in entropy in the molten state given the multiple potential interactions the anion can make while being energetically similar. Since none of the hydrogens are significantly more ‘positive’ than others, the anion can find a way to maximize the total number of interactions, rather than optimization with a single directional interaction as with classical hydrogen bonding.

Supramolecular Interactions

<u>*2.1. General Comments and Trends in Supramolecular Interactions.*</u> To better understand the interactions of the cations, we conducted Hirshfeld surface analysis on all the cations from the crystal structures. The interaction fingerprints are shown in Figure S9. Examining these fingerprints reveals several common topological features. First, the cations share similar interactions, reflected in the overall similarity of the fingerprint shapes. Second, evidence of inefficient crystalline packing is observed in several cations, revealed by the dispersed set of points, particularly in the top right quadrant of the fingerprints. Inefficient packing arising from asymmetric cations and anions is a known contributing factor to the low melting points of ILs. Finally, most of the cations display prominent hydrogen bonding and π interactions, which appear as spikes (for H-bonds) and wings (for π interactions) in the data. Considering the importance of these interactions in relation to the properties of ILs, the following sections examine these specific interactions in detail.[17,18]

Aside from the interaction fingerprints, which provide a facile image of similarities and differences in the supramolecular interactions, Hirshfeld surface analysis allows for quantitative analysis of interaction percentages. This information is provided in graphical form in Figure S10.

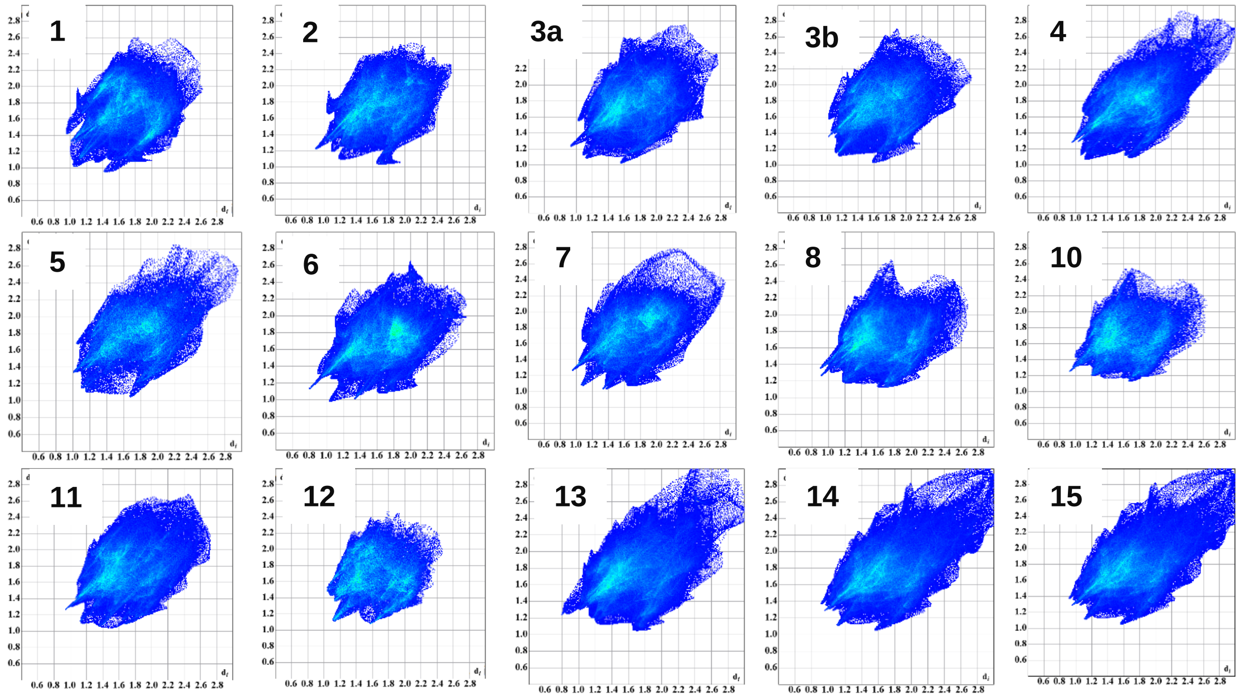

Figure S9. Interaction fingerprints for the compounds. Similarities in shape and size point towards a shared set of interactions based on similar cation structure.

Several general trends emerge when examining the hydrogen interactions within these compounds. First, contacts involving hydrogen atoms (i.e., H···All) account for roughly 75% of the total interactions observed in every crystal—a result that is unsurprising given the organic nature of the cations. The remaining interactions, predominantly involving carbon atoms, make up about 21% of the total. These C···All contacts encompass interactions with the cation's π system (e.g., C···H, C···C, C···F, and C···O), which are recognized as key components in the behavior of ILs.[19]

This interplay of interactions, both the numerically dominant hydrogen contacts and the π-interactions, not only stabilizes the crystalline structure but also reinforces the idea that the anion's capacity for multiple, non-directional contacts is central to the observed entropic effects. Because the anion can engage in multiple, energetically similar interactions with a variety of hydrogen and carbon donors, multiple geometric arrangements of the two moieties are possible upon melting. This allows the anion to move around the cation, maximizing its interactions and contributing to an overall increase in entropy, much like the trends observed in the different polymorphs.

Second, the dications (**13–15**) all display a higher than average percentage of H⋯O | O⋯H and H⋯F | F⋯H interactions compared to the monocationic species. This logically follows from the necessity for charge balance (i.e., dications need two anions). It should be noted, however, that there are some noteworthy anomalies to this observation. For example, the monocationic IL **2-NTf$_2$** displays an H⋯O | O⋯H percentage of 23.8%, which is the second highest overall and nearly as high as the IL **15**. This deviation is rationalized by the presence of favorable H-bonding ring motifs interactions, explained in § 2.2 and within the main narrative.

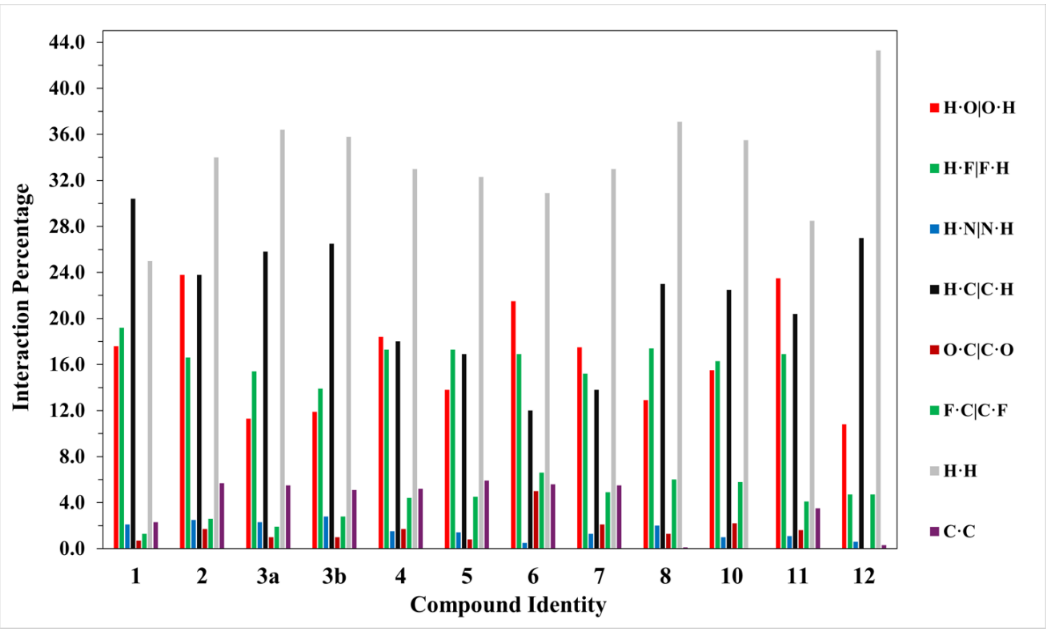

Figure S10. Graphed interaction percentages for each monocationic salts.

The dications also display lower than average percentages of cation⋯cation interactions (i.e., H⋯H, H⋯C | C⋯H, C⋯C). This is also due to charge balancing, which necessitates that the immediate molecular shell of the cations consists of more anions than cations. For example, compound **2-NTf$_2$** is surrounded by seven anions, while compound **13** is surrounded by eleven anions. The presence of more anions in the immediate shell of the dications rationalizes the lower percentage of cation⋯cation interactions in these compounds. Specifically, the dications all have notably lower percentages of C⋯C interactions compared to the monocationic species. In summary, increasing the charge on the cation of an IL increases the cation–anion interactions while decreasing the cation–cation interactions. An exception to this is compound **10**, a monocation, which displays no notable π-stacking (e.g., C⋯C).

Finally, regarding general trends, H···N | N···H interactions represent the lowest percentage of interactions on average. Most of these interactions arise from the anionic imide nitrogen, though certain compounds contain nitrogen in the cation, contributing a small percentage of interactions. Nevertheless, it is well understood at this point that the majority interactions arising from the $[NTf_2]^-$ anion involve the sulfonyl oxygens and the fluorine atoms. This is due to increased charge density on the O atoms arising from the resonance delocalization of the negative charge on the species.[15]

2.2. H-Bonding Rings. Both the *cis* and TS1 conformations[15] are higher in energy than the trans conformation. Thus, in **2**, **8**, and **13**, these higher energy conformations form multiple H-bonding $R_1^2(6)$ ring motifs with the N—H moiety, likely helping to stabilize these conformations through the formation of these stronger hydrogen interactions. In **8**, the non-planar geometry of the two benzene rings in the diphenylamine moiety prevents the formation of this poly-cyclic hydrogen bonding motif observed in **2** and **13** (Figure S11). However, the TS1 anion does still form an additional ring motif with the aromatic C—H moiety ortho to the amine, forming an $R_2^1(6)$ motif where a single sulfonyl oxygen is accepting a H-bond from both the amine and the C—H moiety.

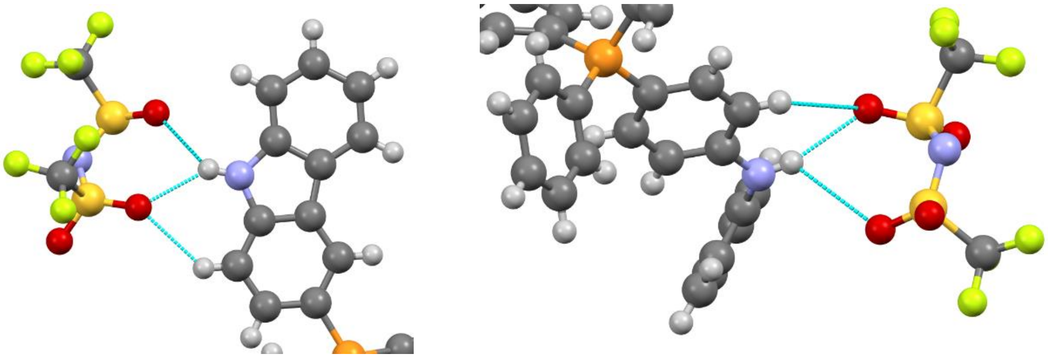

Figure S11. Depiction of the hydrogen bonding rings in **2** (left) and **8** (right). Both systems show cyclical hydrogen bonding with both the amine N—H moiety and the adjacent aromatic C—H. Only parts of the cation are shown for clarity.

IL **13** further allows for additional ring hydrogen bonding motifs, facilitated by the planar geometry of the carbazole moiety and the *cis* conformation of the $[NTf_2]^-$ anion (Figure S12). The carbazole moiety has three hydrogen atoms in a planar arrangement: the amine N—H and the two adjacent C—H aromatic hydrogens. The anion in **13** is disordered with the two conformations having similar geometries. Both are *cis* conformations and allow for multiple cyclical hydrogen bonds to form sulfonyl oxygens and the central imide nitrogen of the anion. As can be seen in the figure, both conformations of the anion allow for multiple hydrogen bonds to form, though different atoms and ring sizes are formed. Thus, speculatively, we can again point towards the potential that given the similar nature of these interactions (i.e., distances, angles, atoms involved, number of interactions) is helping to facilitate multiple conformations of the anion in the molten state, providing more accessible energetic states and thus increasing entropy.

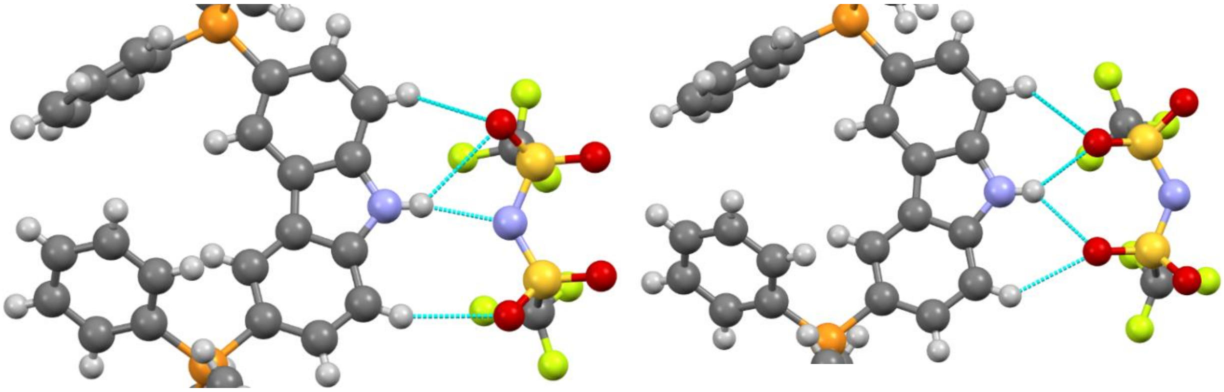

Figure S12. Multiple H-bonding ring motifs are observed with both conformations of the *cis* anion in IL **13**. The pictures show only parts of the cation for clarity. The anion is disordered over two positions and are shown separately in each of the images.

2.3. π Interactions. The cation design of these compounds includes extended π systems, which play a crucial role in determining their thermophysical properties through π interactions. Broadly, interactions with the π system of the ILs account for approximately 22% of the total interactions on average. The majority of these interactions arise from aryl hydrogen interactions with the π system of adjacent aromatic moieties. These H···C | C···H interactions account for approximately 20% of the π interactions, with the remaining percentage mostly consisting of stacking-type interactions (C···C). In the discussion herein, the focus will be on the π interactions involving the varied aryl moieties rather than the benzene moieties on the phosphonium core. Our previous papers have discussed the π interactions of the Ph_3P moieties in detail.[20,21] The H···C | C···H and C···C interaction fingerprints for the compounds are shown in Figures S13 and S14, respectively.

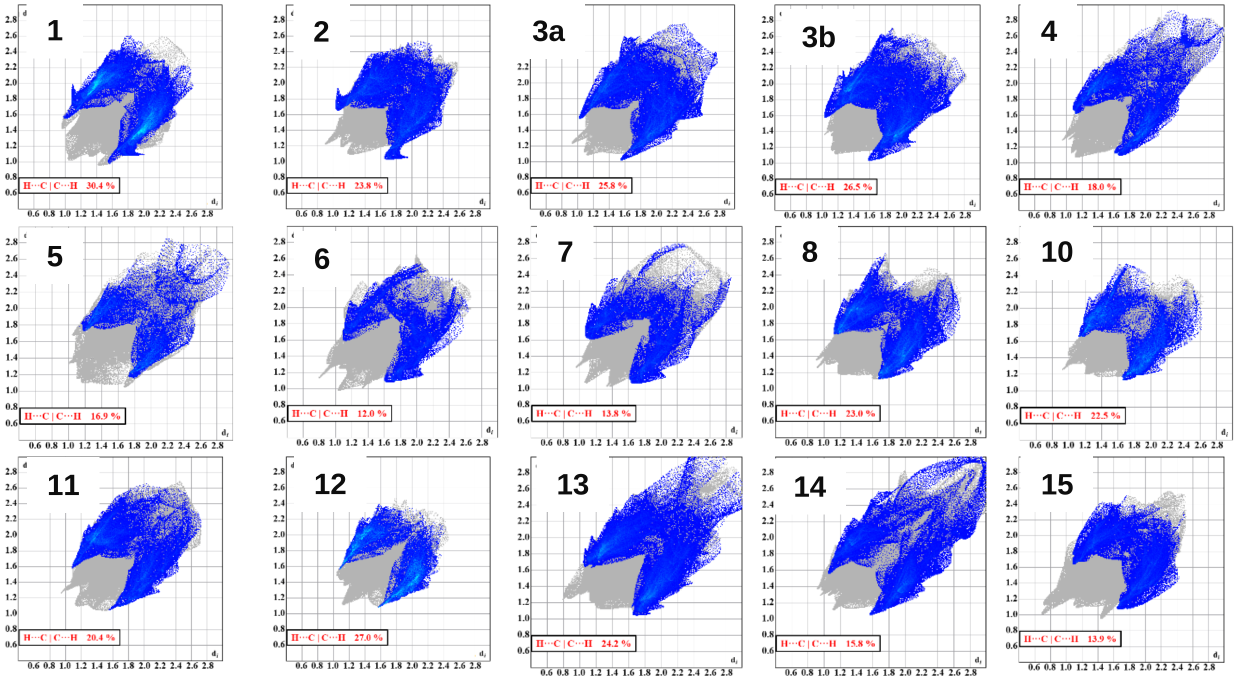

Figure S13. H···C | C···H interaction fingerprints for the compounds. Similarities in structure and interactions are seen in the fingerprints for related compounds, e.g., **8** and **10**.

2.3.1. H···C | C···H interactions. Some general trends emerge from examining the fingerprints of the compounds. First, Compound **1** exhibits the highest percentage of interactions. As discussed in the main narrative, the quinoline moiety forms parallel offset stacking interactions. This arrangement of cations, thus, positions the aromatic moieties to form additional interactions. Furthermore, careful inspection of the fingerprints shows that the interactions in **1** are some of the shortest within this entire set of compounds. Specifically, these short interactions arise between hydrogens on the quinoline moiety and π system of benzene rings on the Ph_3P core. Aromatic hydrogens on the Ph_3P core also form short interactions with the π system of the quinoline. These two interactions then form infinite chains of cations, helping to stabilize the solid state of this IL.

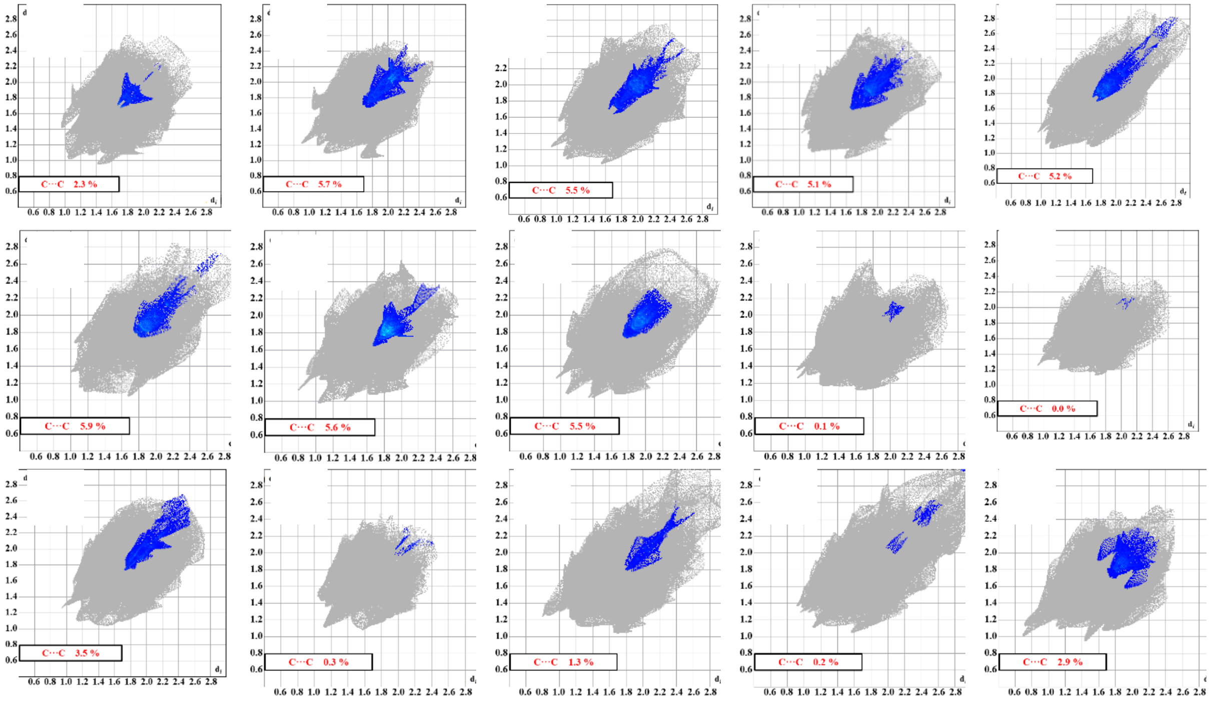

Figure S14. C···C interaction fingerprints for the compounds.

Second, the polymorphs **3a** and **3b** exhibit notably high percentages of H···C | C···H interactions among the compounds examined. This is likely due to the more accessible carbazole moiety in compound **3**. Specifically, the carbazole moiety is positioned further away from the bulky Ph_3P group, distanced by a linking phenyl ring, which can block or compete with interactions due to the sterics of the triphenyl moieties.

Third, compound **6** has the lowest H···C | C···H percentage among the compounds examined, with compound **7** having the second-lowest percentage. Both of these compounds contain a carbonyl moiety as part of the extended polycyclic system. The carbonyl group in compounds **6** and **7** form a notable percentage of O···H interactions of 4.3% and 3.1% respectively. Thus, it could be that these interactions are more energetically preferred over the formation of H···C | C···H interactions. Additionally, the sulfur moiety within the π system influences the electronic structure of **7**, affecting the charge distribution in the aromatic system.

Additionally, the sulfur forms several S⋯H interactions with adjacent cations. Thus, a complex set of competing interactions arise as more functional groups are introduced into the π moieties of the cations.

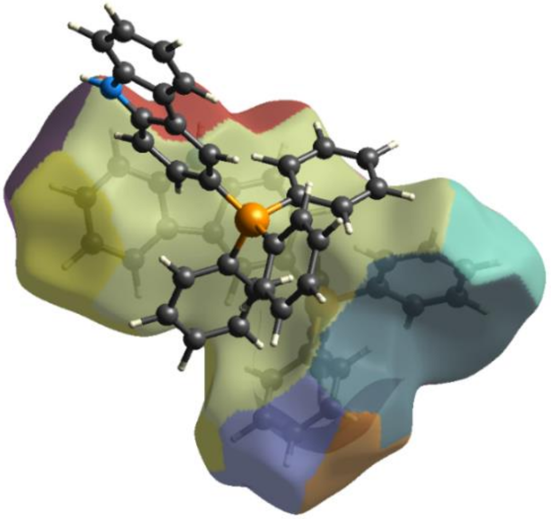

Figure S15. Depiction of the calculated Hirshfeld Surface of **2** with the fragment patches shown. Each unique color represents a distinctive neighboring molecule, which interacts with the central compound. The large beige fragment patch shown represents the largest area of interaction in this molecule.

Finally, IL **2** stands out due to the presence of distinct H⋯C | C⋯H wing features. These wings arise from perpendicular T-shaped C—H⋯π interactions between Ph_3P aromatic rings. These interactions form chains of cations, leading to the symmetric nature of the fingerprint and are among some of the shortest H⋯C | C⋯H interactions in the entire series. Other compounds in the series also exhibit similar interactions leading to wing features, such as compound **11**, which shows perpendicular Y-shaped C—H⋯π interactions. The difference in interaction geometry (T-shape vs. Y-shape)[22] is manifested in the different shapes of the wings.

<u>2.3.2. C⋯C Interactions.</u> One of the main utilities of Hirshfeld surface analysis lies in its ability to facilitate the comparison of interaction motifs via the shapes of fingerprint plots. Examining the fingerprints for the compounds reveals a diverse set of interactions with multiple unique patterns emerging. However, closer inspection begins to reveal some recurring interaction motifs. For example, compounds **8**, **10**, and **12** display an insignificant amount of C⋯C interactions, well below the average of 3.7 % for the entire set of compounds. Interestingly, these compounds exhibit an above-average amount of H⋯C | C⋯H interactions, pointing towards a subtle balance between C⋯C and H⋯C | C⋯H interactions within these ILs. Notably, the geometric requirements to form C⋯C are more rigorous than H⋯C | C⋯H interactions since the π systems are required to align in parallel manner to form the stacking interactions which lead to C⋯C.

Broadly speaking, notable patterns emerge concerning cation geometry and interactions. Compounds **1–7** all contain conjugated polycyclic π systems. ILs **2–7** display higher-than-average percentages of C⋯C stacking arising from these moieties. In contrast, **8**, **10**, and **12** display lower percentages of C⋯C stacking due, in part, to the aromatic moieties in these systems not forming

extended planar systems. The central linking atom, oxygen in the case of **10**, breaks the planar geometry of the aromatic system thus decreasing π stacking interactions.

Compound **1** shows a lower than average percentage of C⋯C interactions, however this can be rationalized as the aromatic system in **1** is comprised of only two conjugated rings rather than the three rings found in, for example, **2**. However, despite this lower percentage, **1** does display parallel offset stacking interactions as discussed in the main narrative. This draws another important point that percentages are a means to identify patterns rather than indicative of which interactions are most important within a compound.

Likewise, the dicationic ILs show lower percentages of π stacking. There are two possible explanations for this:

1. Charge Balance Requirements: The increased charge on the cation requires more anions in the immediate molecular shell, thus replacing cation–cation interactions with cation–anion interactions.
2. Steric Hindrance: The dications have Ph_3P moieties on both ends of the extended planar π systems, which restricts the geometries capable of forming stacking interactions.

Another pattern emerges when examining compounds which all display a distinctive fish-tail pattern in the C⋯C fingerprint plots as exemplified in **3** and **13**. The "tail" portion of these interactions arises from canted, offset π interactions between the extended aromatic moieties on the cations (e.g., carbazole and dibenzothiophene groups) and adjacent phenyl rings. Furthermore, a distinction between the polymorphs **3a** and **3b** emerges when examining the C⋯C interactions. While both display a similar percentage of interactions, the nature of these non-covalent interactions is quite different – **3a** shows the fish-tail motif, whereas **3b** displays an arrowhead-like fingerprint with two noticeable wing features.

This arrowhead-like fingerprint is also found in compounds **2** and partially in compound **11**. ILs **3b** and **2** both contain the carbazole moiety, following similar interactions would appear in both. The arrowhead-like interaction fingerprint arises from reciprocal π-stacking interactions between the carbazole rings and the Ph_3P cations, forming extended chains. The reciprocal nature of the interaction accounts for the formation of the two wing features in the fingerprint plots. Variations in local geometry surrounding the carbazole moieties in both molecules account for subtle differences in interaction distances and angles, reflected in the fingerprints. Compound **11** also displays these chain-like π interactions despite the lack of a carbazole group.

Finally, the overall geometry of the aromatic moieties in compounds **2** and **8**, as well as in compounds **4** and **10**, allows for a broad comparison of the influence of geometry. Specifically, compounds **2** and **8** differ only in the planar versus tetrahedral geometry of the extended aromatic system. The planar geometry of compound **2** allows for a marked increase in C⋯C interactions. The same trend is observed with compounds **4** and **10**, wherein the planar arrangement in compound **4** results in a higher percentage of C⋯C interactions compared to its tetrahedral congener, compound **10**. Notably, however, an increase in C⋯C interactions does not necessarily mean a decrease in H⋯C | C⋯H interactions for these contrasting pairs of compounds.

These observations are reflected in the calculated fragment patches of the compounds. Briefly, fragment patches are a visualization of the number of molecules and contact areas between individual molecules in the crystal. For example, compound **2** makes contact with 18 adjacent molecules (fragment count of 18) with the largest surface area contact of approximately 79 $Å^2$. This beige-colored contact area happens to be directly above the planar carbazole moiety,

corresponding to a series of H···C | C···H interactions with the π system of the ring (Figure S11). The comparable interactions in IL **8** cover an area of about 47 Å², which is not the largest interaction based on contact surface area. A similar trend is observed in ILs **4** and **10**, where IL **4** displays a larger fragment area compared to IL **10**, also involving the planar aromatic moiety. These observations point to changes in cation interactions based on the local geometry of the aromatic moieties on the cations, with the more accessible planar geometries allowing for increased cation–cation interactions. As a final point, we also observe this when comparing compound **1** with $[PPh_4][NTf_2]$ which is discussed in the manuscript.

To summarize the discussion of the H···C | C···H and C···C interactions:

- The C···C interaction motifs are quite varied, yet two notable patterns emerge: the fish-tail and arrowhead motifs. Both arise from distinct stacking interactions between the PPh_3 rings and the unique aromatic systems of the respective cations.
- The addition of a phenyl linker in IL **3** increases the amount of π interactions by distancing the bulky PPh_3 group from the extended aromatic system. Thus, IL **3** has a higher-than-average percentage of cation–cation interactions.
- The geometry of the extended aromatic systems allows for a measure of control over C···C interactions. A planar arrangement of these moieties permits greater C···C interactions. Notably, the dications have lower-than-average C···C interactions due, in part, to the steric influence of the two bulky PPh_3 moieties.

2.4. $[PF_6]^-$ vs. $[NTf_2]^-$ Anions. Compound **2** was crystallized with both $[PF_6]^-$ and $[NTf_2]^-$ anions, enabling us to examine the effects of variations in anion geometry and composition. The fingerprints for both compounds are shown in Figure S16. Several key details emerge from contrasting the supramolecular interactions of the two compounds.

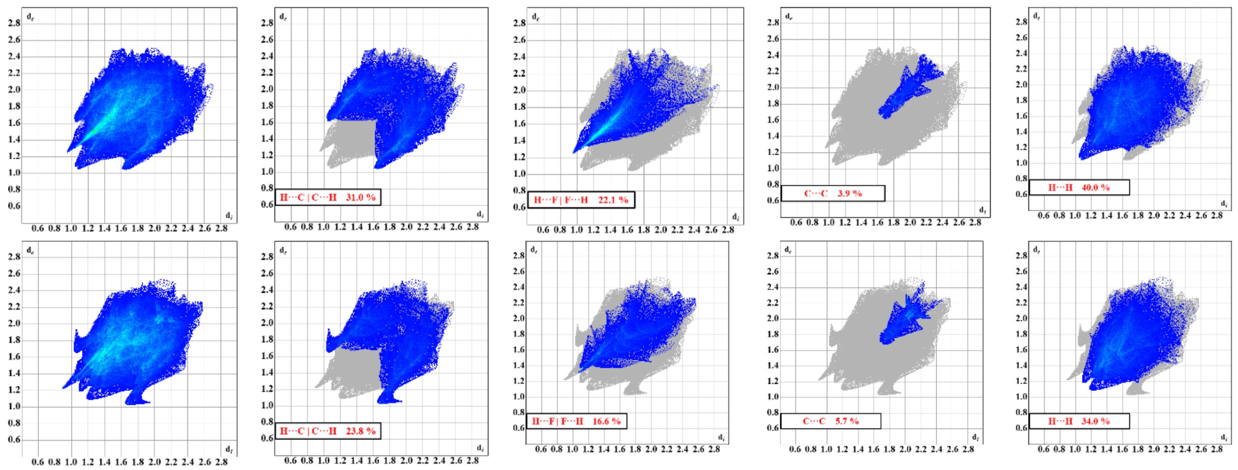

Figure S16. The interaction fingerprints for **2-PF$_6$** (top) and **2-NTf$_2$** (bottom). Change in anion geometry and composition noticeably change the interactions formed in each compound.

First, as expected, **2-PF$_6$** displays the highest overall percentage of H···F | F···H interactions among all the compounds examined. This is due to two reasons: (i) the composition of the PF_6^- anion, and (ii) the sulfonyl oxygen moieties in the $[NTf_2]^-$ anion compete with the CF_3 groups for hydrogen interactions, thus lowering the H···F | F···H interaction percentage in $[NTf_2]^-$

-based compounds. Notably, while the H···F | F···H interactions are high, **2-PF$_6$** also has the lowest percentage of F···C | C···F interactions. We speculate that the H···F | F···H interactions are energetically preferred over the F···C | C···F interactions, so an increase in hydrogen interactions comes at the cost of decreased F···C | C···F interactions. It should be noted that both F···C | C···F and H···F | F···H interactions represent cation–anion interactions; thus, a higher percentage of these interactions typically corresponds to higher melting points.

Second, **2-PF$_6$** has a higher percentage of H···H interactions than **2-NTf$_2$**. Furthermore, the 40% of H···H interactions in **2-PF$_6$** is higher than the average for the compounds (approximately 35%). Close examination of the H···H fingerprint plots for both compounds also reveals that the H···H interactions in **2-PF$_6$** are shorter than those in **2-NTf$_2$**. These two observations – the higher percentage and shorter distances – are likely related to the smaller volume of the $[PF_6]^-$ anion, which allows cations to be closer to each other, thus increasing the H···H contacts while concomitantly shortening the interactions.

Finally, both **2-PF$_6$** and **2-NTf$_2$** display the arrowhead-like C···C interaction fingerprint, corresponding to reciprocal interactions between the aromatic moieties on the Ph_3P core and the carbazole moiety. However, while both compounds display these reciprocal interactions, the geometries of the interactions are quite different, as revealed by the different shapes and percentages in the respective fingerprints.

2.5. Sterics of Triphenyl Moieties. The impact of sterics becomes evident when contrasting **9-NTf$_2$** with its PF_6 analog. The smaller PF_6^- anion can more readily access the pocket between N and P atoms, enabling stronger cation-anion interactions and promoting crystallinity. This change in pocket size is illustrated by comparing the P···N distance in **9-PF$_6$** (5.980 Å) with the P···Si distance in the silicon analog (6.499 Å, **12**). The larger silicon atom creates more space between the phenyl groups, reducing steric hindrance, increasing the size of the pocket in the cation, and allowing for additional interactions that are absent in **9-NTf$_2$**. As discussed above, a solvent molecule is seen to occupy this space between the triphenyl moieties. This points towards the theory that a small molecule could access this pocket within the cation, allowing for the formation of the crystal structure. This idea of steric blocking using triphenyl moieties is observed in the dicationic systems. The bulky Ph_3P moieties hinder the formation of stacking interactions due to the bulky nature of these groups. This is observed in the lower C···C percentage for these ILs.

Polymorphism

To better understand the formation of the polymorphs **3a** and **3b** we employed the computational tools available in *CrystalExplorer25*. As has been discussed, crystal polymorphism arises from the fact that the potential energy landscape for crystallization is fairly flat, with multiple possible local minima leading to the inevitable formation of stable or metastable polymorphs.[23,25] Herein we approached analysis of the polymorphs by means of examining energetic changes in the interactions and crystalline structure of the two crystals as a supplement to the other data collected on the samples (i.e., melting points, thermal stability).

Before discussing our results, a few crucial caveats must be stated. First, we present energy framework[25] calculations using *CrystalExplorer25*.[26] The exact energy values (Table S1 and S2) are more than likely inaccurate due to calculation of the wavefunctions of the ions as

described in the literature.[27,28] This is not a fault of the *CrystalExplorer* itself. Its methods have been validated in numerous studies. Rather it highlights the challenges that organic crystals pose for the computational routines currently packaged with the program. Access to alternative computational tools (see below) might overcome this limitation. Even so, any systematic error would affect both **3a** and **3b** equally, so comparing the two in isolation remains valid. Importantly, the calculated values still make logical sense: differing anion conformations produce distinct interaction energies with the cations (vide infra). Further, it is essential to keep in mind that coulombic interactions will (and do) dominate the intermolecular interactions of ILs.[29] This is borne out in the energy values we present. The discussion with respect to other interactions contribute as supplementary interactions, however, they can account for 20 – 30% of the total lattice energy for IL systems, as the results also demonstrate.[30,31]

Second, we used the computational tools we have available. There are more sophisticated, and extremely costly, both with respect to licensing and computational power, computational programs available (e.g., Gaussian, Materials Studio, CRYSTAL23, Quantum ESPRESSO) which would further resolve the lingering issues of the two polymorphs. We simply do not have access to these programs.

Third, for our calculations, we generated two differently-sized clusters: a smaller one containing all molecules within 5 Å of a central cation, and a larger one extending to 10 Å. Both datasets were consistent; however, these clusters are smaller than the 30–40 Å range typically recommended for lattice-energy calculations.[28,32] Attempts to run the calculations at larger clusters failed.

Finally, all energies were calculated at the HF 3-21G level of theory implemented in CrystalExplorer. Attempts to run calculations on larger clusters or at a higher level of theory also failed, though a calculation on a single cation-anion pair at the CE-1p-B3LYP level of theory was completed (Table S3).

With these considerations in mind, Tables S1 and S2 present the calculated interaction energies for the 10 Å clusters of **3a** and **3b**. Summing the total energies to approximate the lattice energy suggests that **3b** is lower in energy than **3a**. This contrasts with the single ion-pair interaction energy calculation, which indicates **3a** is lower. The discrepancy likely arises from increased $OSO_2\cdots H$ interactions in the asymmetric unit of **3a** due to the anion's orientation, implying that the distinguishing factors for these polymorphs lie in long-range interactions.

These long-range interactions are depicted in the energy frameworks shown in Figure S17. Several notable observations emerge from visual inspection of these frameworks. First, the Coulombic framework appears quite messy and difficult to interpret, which is expected given the isotropic nature of Coulombic interactions. Second, the dispersion (green) and exchange (magenta) frameworks look quite similar, and dispersion is the second-largest contributor to the total system energy.

Third, the primary differences appear in the polarization (rose) and repulsion (yellow) frameworks, where the tube diameters are proportional to each interaction's strength. The conformational changes in the cis anion logically alter molecular positions, thereby logically influencing repulsion. Interestingly, although repulsion increases in **3b**, its electrostatic contribution also increases, resulting in a lower overall lattice energy for this polymorph.

In short, our examination of the polymorphs' energy reveals several key observations. First, different anion conformations indeed influence the overall lattice energy, which might seem

self-evident since crystals of both samples physically exist and can be reproducibly isolated, albeit as a mixture of intergrown crystals. At first glance, this is surprising because both cis-anion conformations also appear in the molten state and in solution, where they often manifest as disorder in crystals. Nevertheless, there must be a specific property of this compound that enables the consistent formation of isolable crystals containing both conformations.

Second, there are subtle variations in the individual energetic contributions arising from changes in the anion conformations. Again, this observation may seem self-evident, given the physical differences in the asymmetric unit (e.g., atom-atom interactions). However, focusing solely on overlapping radii is a rather simplistic approach to crystal analysis. When examined from a broader perspective, these variations become evident as noteworthy shifts in long-range interactions. Consequently, these preliminary findings suggest the value of a more extensive investigation into these polymorphs.

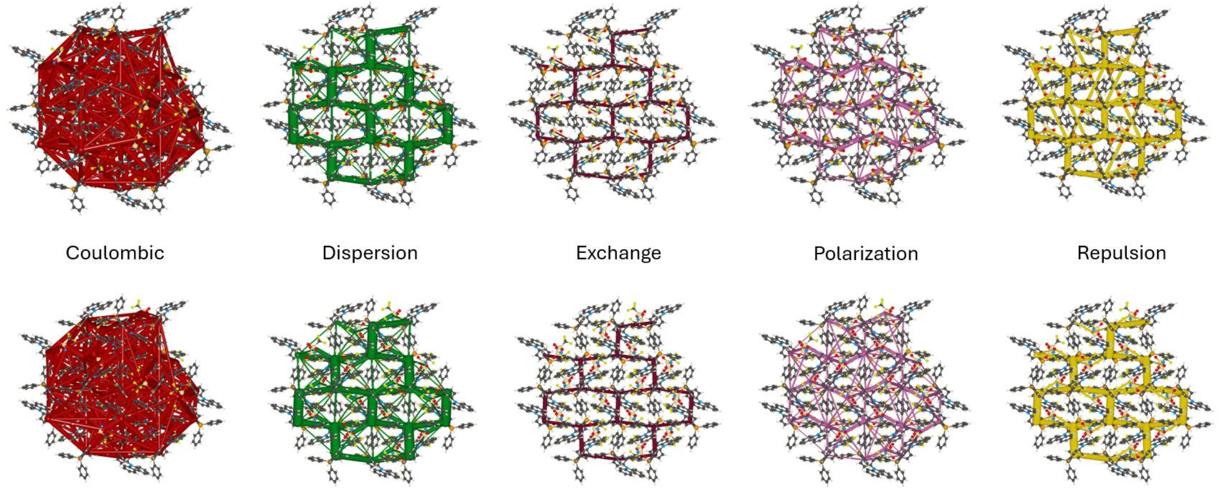

Figure S17. Energy frameworks for **3a** (top) and **3b** (bottom). The tube size is proportional to the calculated energy value (bigger value, larger radius tube). All images are viewed down on the crystallographic A axis. Interactions less than 10 kJ/mol are omitted from the images.

Conclusion

The highly crystalline nature of these Ph_3P-based ILs allows for rigorous examination of the supramolecular interactions, allowing for correlation between structure and properties. Several heuristic principles are gleaned from this analysis:

i. The carbazole moiety enables the formation of distinctive hydrogen-bonding rings with the $[NTf_2]^-$ anion. In all three cations containing this group a $R_1^2(6)$ ring forms between the sulfonyl oxygen atoms of the $[NTf_2]^-$ anion and the amine hydrogen of the carbazole group. This hydrogen-bonding ring appears to stabilize the higher energy *cis* and TS1 conformations of the $[NTf_2]^-$ anion, with all three crystals displaying these uncommon conformations in the solid state.
ii. The dicationic species exhibit a higher number of interactions with surrounding anions, as expected due to charge balance requirements. This is evidenced by a reduction in the

percentage of cation–cation interactions (such as H···C | C···H) and an increase in cation–anion interactions (such as O···C | C···O). Notably, both sets of interactions involve the π system of the cation, emphasizing the importance of these non-covalent interactions in determining the thermophysical properties of ILs. This also suggests potential applications of these interactions in areas such as separations.

iii. The accessibility of the extended π systems is influenced by the geometry of the bond to the phosphonium core. For example, compound **10** contains a diphenyl ether moiety with a tetrahedral geometry, whereas compound **4** contains a completely planar dibenzofuran group. The planar arrangement in **4** allows for increased C···C interactions compared to the tetrahedral arrangement in **10**. Similarly, compounds **2** and **8** display this trend, with the planar moiety in compound **2** leading to increasing C···C interactions. This demonstrates that planar geometries facilitate greater π–π stacking interactions, which can influence the properties of the ILs.

iv. Examining polymorphs **3a** and **3b** reveals subtle variations in cation–anion interactions. Despite these differences, the key metrics (distances, angles, and overall geometry) are nearly identical, suggesting that multiple anion orientations can form energetically similar interactions. This multiplicity of interactions likely contributes to increased entropy observed upon melting. Furthermore, the extended π-systems in these compounds play a significant role in lattice stabilization. Recent studies indicate that hydrogen bonding and π-stacking interactions (e.g., C···C and H···C|C···H contacts) are nearly equally important in co-crystal systems,[23] and enhanced π interactions in ILs facilitate additional cation–cation contacts, leading to lower melting points. Thus, a combination of energetically similar hydrogen interactions and competitive π interactions appear as a reasonable hypothesis for rationalizing the thermodynamic contributions to the melting points as discussed in the main narrative.

Crystal Data and Structure Refinement for the Products

For general procedures common to all structures, see Crystallographic Data and Discussion section (*vide supra*).

	IL **1**	IL **2-NTf$_2$**	IL **2-PF$_6$**	IL **3a**
**Crystal data**				
Chemical formula	$C_{27}H_{21}NP·C_2F_6NO_4S_2$	$C_{30}H_{23}NP·C_2F_6NO_4S_2$	$C_{30}H_{23}F_6NP_2$	$C_{36}H_{27}NP·C_2F_6NO_4S_2$
M_r	670.57	708.61	573.43	784.70
Crystal system, space group	Monoclinic, $P2_1/c$	Orthorhombic, *Pbca*	Orthorhombic, $Pca2_1$	Monoclinic, $P2_1/n$
Temperature (K)	150	150	150	150
a, *b*, *c* (Å)	11.3019(4), 16.9497(6), 15.5372(6)	18.990(5), 13.1895(19), 24.341(5)	18.855(3), 8.7778(11), 15.697(2)	13.6611(7), 15.6623(8), 16.3646(9)
β (°)	99.324(1)	90	90	93.655(3)
V (Å^3)	2937.04(19)	6097(2)	2597.9(6)	3494.3(3)
Z	4	8	4	4
Radiation type	Mo *K*α	Mo *K*α	Mo *K*α	Cu *K*α
μ (mm^{-1})	0.31	0.31	0.23	2.50
Crystal size (mm)	0.18 × 0.15 × 0.08	0.43 × 0.32 × 0.12	0.31 × 0.28 × 0.06	0.23 × 0.10 × 0.09
**Data collection**				
Diffractometer	Bruker AXS D8 Quest			
Absorption correction	Multi-scan, *SADABS* 2016/2			
T_{min}, T_{max}	0.692, 0.747	0.682, 0.747	0.413, 0.746	0.588, 0.754
No. of measured, independent and observed [$I > 2σ(I)$] reflections	52903, 11213, 6931	160447, 11641, 8917	25218, 5541, 3968	60086, 7594, 6794
R_{int}	0.059	0.048	0.116	0.068
$(\sin θ/λ)_{max}$ (Å^{-1})	0.770	0.770	0.666	0.639
**Refinement**				
$R[F^2 > 2σ(F^2)]$, $wR(F^2)$, S	0.047, 0.117, 1.01	0.042, 0.121, 1.05	0.051, 0.129, 1.04	0.074, 0.210, 1.08
No. of reflections	11213	11641	5541	7594
No. of parameters	533	424	356	478
No. of restraints	519	-	2	-
H-atom treatment	Constrained	Constrained	Mixed	Constrained
$Δρ_{max}$, $Δρ_{min}$ (e Å^{-3})	0.37, −0.46	0.42, −0.67	0.28, −0.44	0.73, −0.53
Absolute structure	-	-	Flack x determined using 1225 quotients [(I+)-(I-)]/[(I+)+(I-)]	-
Absolute structure parameter	-	-	0.02 (8)	-

	IL **3b**	IL **4**	IL **5**	IL **6**

**Crystal data**				
Chemical formula	$C_{36}H_{27}NP·C_2F_6NO_4S_2$	$C_{30}H_{22}OP·C_2F_6NO_4S_2$	$C_{30}H_{22}PS·C_2F_6NO_4S_2$	$C_{31}H_{22}O_2P·C_2F_6NO_4S_2$
M_r	784.70	709.59	725.65	737.60
Crystal system, space group	Monoclinic, $P2_1/n$	Orthorhombic, *Pbca*	Orthorhombic, *Pbca*	Triclinic, $P\bar{1}$
Temperature (K)	150	150	150	150
a, *b*, *c* (Å)	14.1879(11), 14.8621(10), 16.4244(11)	18.8537(7), 17.3295(6), 19.4300(9)	18.9755(9), 17.3055(9), 19.5136(10)	12.2156(9), 15.5662(13), 26.979(2)
α, β, γ (°)	90, 92.592(5), 90	90, 90, 90	90, 90, 90	79.356(3), 81.001(2), 76.006(2)
V ($Å^3$)	3459.7(4)	6348.3(4)	6407.9(6)	4858.4(7)
Z	4	8	8	6
Radiation type	Cu *K*α	Mo *K*α	Mo *K*α	Mo *K*α
μ (mm^{-1})	2.52	0.30	0.36	0.30
Crystal size (mm)	0.19 × 0.17 × 0.16	0.53 × 0.48 × 0.42	0.43 × 0.41 × 0.29	0.32 × 0.19 × 0.16
**Data collection**				
Diffractometer	Bruker AXS D8 Quest			
Absorption correction	Multi-scan, *SADABS* 2016/2			
T_{min}, T_{max}	0.601, 0.754	0.670, 0.747	0.671, 0.747	0.657, 0.746
No. of measured, independent and observed [$I > 2\sigma(I)$] reflections	29107, 7416, 6439	193688, 12710, 9327	148513, 12233, 8691	113710, 24102, 19043
R_{int}	0.051	0.065	0.082	0.036
$(\sin \theta/\lambda)_{max}$ ($Å^{-1}$)	0.639	0.782	0.770	0.669
**Refinement**				
$R[F^2 > 2\sigma(F^2)]$, $wR(F^2)$, S	0.047, 0.134, 1.07	0.040, 0.123, 1.03	0.037, 0.102, 1.02	0.065, 0.184, 1.06
No. of reflections	7416	12710	12233	24102
No. of parameters	478	424	424	2005
No. of restraints	-	-	-	4915
H-atom treatment	Constrained	Constrained	Constrained	Constrained
$\Delta\rho_{max}$, $\Delta\rho_{min}$ (e $Å^{-3}$)	1.02, −0.52	0.46, −0.41	0.43, −0.43	1.09, −0.58

	IL **7**	IL **8**	IL **9-PF$_6$**	IL **10**
**Crystal data**				
Chemical formula	$C_{31}H_{22}OPS·C_2F_6NO_4S_2$	$C_{30}H_{25}NP·C_2F_6NO_4S_2$	$C_{36}H_{29}NP·F_6P$[+solvent]	$C_{30}H_{24}OP·C_2F_6NO_4S_2$
M_r	753.66	710.63	651.54	711.61
Crystal system, space group	Triclinic, $P\bar{1}$	Orthorhombic, $Pca2_1$	Monoclinic, $P2_1/c$	Orthorhombic, $Pca2_1$
Temperature (K)	150	150	150	150
a, *b*, *c* (Å)	10.1124(3), 11.8054(3), 14.7226(5)	18.3030(7), 9.0442(3), 19.0341(9)	12.1445(18), 7.2917(15), 41.313(8)	18.1938(8), 9.1950(5), 18.6910(8)
α, β, γ (°)	90.233(1), 105.459(1), 107.041(1)	90, 90, 90	90, 96.366(13), 90	90, 90, 90
V (Å^3)	1613.18(8)	3150.8(2)	3635.9(11)	3126.9(3)
Z	2	4	4	4
Radiation type	Mo $K\alpha$	Mo $K\alpha$	Cu $K\alpha$	Mo $K\alpha$
μ (mm^{-1})	0.36	0.30	1.56	0.30
Crystal size (mm)	0.32 × 0.29 × 0.23	0.45 × 0.41 × 0.05	0.55 × 0.03 × 0.03	0.32 × 0.29 × 0.05
Data collection				
Diffractometer	Bruker AXS D8 Quest			
Absorption correction	Multi-scan, *SADABS* 2016/2\			
T_{min}, T_{max}	0.687, 0.747	0.679, 0.747	0.515, 0.754	0.692, 0.747
No. of measured, independent and observed [$I > 2\sigma(I)$] reflections	73340, 12309, 10051	49976, 11525, 8763	27038, 7516, 5550	180614, 11972, 10087
R_{int}	0.037	0.062	0.132	0.061
$(\sin \theta/\lambda)_{max}$ (Å^{-1})	0.770	0.769	0.638	0.772
Refinement				
$R[F^2 > 2\sigma(F^2)]$, $wR(F^2)$, S	0.039, 0.115, 1.04	0.041, 0.110, 1.02	0.118, 0.326, 1.06	0.032, 0.084, 1.03
No. of reflections	12309	11525	7516	11972
No. of parameters	442	563	492	560
No. of restraints	-	548	251	592
H-atom treatment	Constrained	Mixed	Constrained	Constrained
$\Delta\rho_{max}$, $\Delta\rho_{min}$ (e Å^{-3})	0.59, −0.50	0.40, −0.33	0.46, −0.70	0.29, −0.22
Absolute structure	-	Flack x determined using 3444 quotients [(I+)-(I-)]/[(I+)+(I-)]	-	Flack x determined using 4331 quotients [(I+)-(I-)]/[(I+)+(I-)]
Absolute structure parameter	-	−0.004(19)	-	−0.006(10)

	IL **11**	IL **12**	IL **13**	IL **14**
**Crystal data**				
Chemical formula	$C_{30}H_{24}O_2PS·C_2F_6NO_4S_2$	$C_{42}H_{34}PSi·C_2F_6NO_4S_2$	$C_{48}H_{37}NP_2·2(C_2F_6NO_4S_2)$	$C_{48}H_{36}OP_2·2(C_2F_6NO_4S_2)$
M_r	759.67	877.90	1250.02	1251.01
Crystal system, space group	Triclinic, $P\bar{1}$	Triclinic, $P\bar{1}$	Orthorhombic, $Pna2_1$	Triclinic, $P\bar{1}$
Temperature (K)	150	150	150	150
a, *b*, *c* (Å)	17.6427(6), 20.1507(9), 20.3250(7)	7.2784(3), 12.0888(5), 12.7434(6)	16.3785(6), 20.4679(7), 16.0134(6)	14.2651(4), 14.3862(5), 14.6944(6)
α, β, γ (°)	73.175(1), 72.225(1), 80.448(1)	67.730(2), 75.505(2), 82.688(2)	90, 90, 90	66.877(1), 83.015(1), 89.463(1)
V (Å^3)	6562.4(4)	1003.93(8)	5368.2(3)	2750.31(17)
Z	8	1	4	2
Radiation type	Mo $K\alpha$	Mo $K\alpha$	Cu $K\alpha$	Mo $K\alpha$
μ (mm^{-1})	0.36	0.28	3.08	0.33
Crystal size (mm)	0.38 × 0.32 × 0.12	0.43 × 0.16 × 0.14	0.45 × 0.21 × 0.18	0.27 × 0.24 × 0.17
**Data collection**				
Diffractometer	Bruker AXS D8 Quest			
Absorption correction	Multi-scan, *SADABS* 2016/2			
T_{min}, T_{max}	0.683, 0.747	0.698, 0.747	0.666, 0.747	0.690, 0.747
No. of measured, independent and observed [$I > 2\sigma(I)$] reflections	274962, 50137, 33256	40777, 7615, 5950	99067, 11957, 10838	97814, 20980, 15332
R_{int}	0.073	0.041	0.057	0.045
$(\sin \theta/\lambda)_{max}$ (Å^{-1})	0.770	0.771	0.649	0.770
**Refinement**				
$R[F^2 > 2\sigma(F^2)]$, $wR(F^2)$, S	0.046, 0.132, 1.04	0.045, 0.111, 1.06	0.031, 0.083, 1.04	0.042, 0.115, 1.03
No. of reflections	50137	7615	11957	20980
No. of parameters	1765	335	870	860
No. of restraints	-	75	604	573
H-atom treatment	Constrained	Constrained	Mixed	Constrained
$\Delta\rho_{max}$, $\Delta\rho_{min}$ (e Å^{-3})	1.22, −1.01	0.39, −0.44	0.41, −0.31	0.51, −0.58
Absolute structure	-	-	inversion twin.	-
Absolute structure parameter	-	-	0.385(12)	-

	IL **15**
**Crystal data**	
Chemical formula	$C_{48}H_{38}O_2P_2S\cdot 2(C_2F_6NO_4S_2)$
M_r	1301.08
Crystal system, space group	Triclinic, $P\bar{1}$
Temperature (K)	150
a, *b*, *c* (Å)	11.9087(6), 12.4092(3), 19.8049(6)
α, β, γ (°)	100.474(2), 95.149(2), 105.326(2)
V (Å^3)	2746.15(18)
Z	2
Radiation type	Cu *K*α
μ (mm^{-1})	3.40
Crystal size (mm)	0.21 × 0.20 × 0.09
**Data collection**	
Diffractometer	Bruker AXS D8 Quest
Absorption correction	Multi-scan, *SADABS* 2016/2
T_{min}, T_{max}	0.548, 0.754
No. of measured, independent and observed [$I > 2\sigma(I)$] reflections	135338, 11896, 10730
R_{int}	0.060
$(\sin \theta/\lambda)_{max}$ (Å^{-1})	0.639
**Refinement**	
$R[F^2 > 2\sigma(F^2)]$, $wR(F^2)$, S	0.049, 0.148, 1.04
No. of reflections	11896
No. of parameters	1803
No. of restraints	6516
H-atom treatment	Constrained
$\Delta\rho_{max}$, $\Delta\rho_{min}$ (e Å^{-3})	0.50, −0.58

Additional Refinement Details

1: The anion is disordered by pseudo-inversion. The two disordered moieties were restrained to have similar geometries. U_{ij} components of ADPs for disordered atoms closer to each other than 2.0 Å were restrained to be similar. Subject to these conditions the occupancy ratio refined to 0.9554(12) to 0.0446(12).

3a and **3b**: Macroscopic crystals had plate like shape (Figure S18). Inspection using polarized light microscopy suggested some twinning, indicated by striations and color variation when viewed with polarized light. Attempts to fit first macroscopic crystals and then small fragment to one or more domains that matched the same unit cell were unsuccessful. The patterns were also tested for presence of a supercell (commensurate modulation) or incommensurate modulation. None of these could be found, indicating that the crystals were not twinned (at least not "only" twinned) but consisted of an intergrowth of compounds with different unit cells. Closer inspection of the patterns (using the program Cell Now) revealed for small fragments the presence of two closely related but different monoclinic primitive unit cells. Successive screening of various fragments with uniform color in polarized light allowed to find sufficiently large crystallites dominated by one unit cell and domain with negligible overlap with other domains and full data for both polymorphs were collected from these.

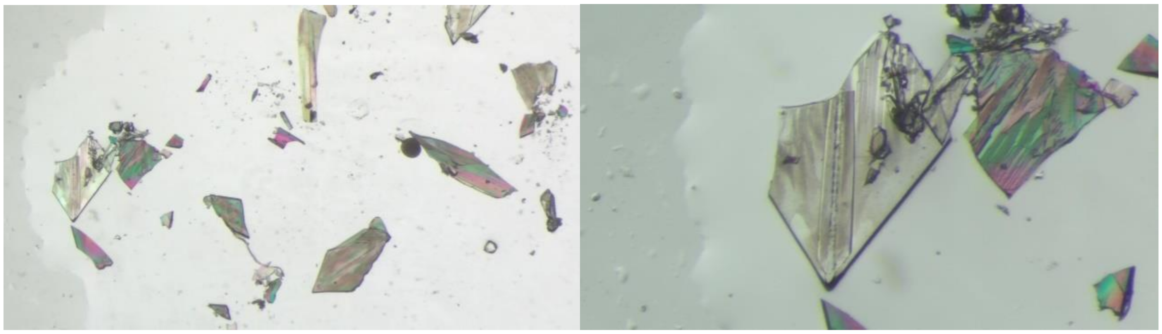

Figure S18. Images of the crystals of **3** viewed under a microscope.

6: Major disorder is observed for one of the three triflimide anions. Five-fold whole anion disorder was refined. Two-fold disorder was refined for a second triflimide (apparent very minor additional disorder that would have required to refine unresolved disorder for atoms of the neighboring cations was ignored). All disordered moieties were restrained to have similar geometries as the one not disordered triflimide. U_{ij} components of ADPs for disordered atoms closer to each other than 2.0 Å were restrained to be similar. Subject to these conditions the occupancy ratio refined to 0.9535(18) to 0.0465(18) for the two-fold disorder, and 0.297(2) to 0.312(2) to 0.215(2) to 0.133(2) to 0.0430(14).

8: The anion was refined as disordered over two orientations. The two disordered moieties were restrained to have similar geometries. U_{ij} components of ADPs for disordered atoms closer to each other than 2.0 Å were restrained to be similar. Subject to these conditions the occupancy ratio refined to 0.646(3) to 0.354(3).

9-PF_6: A phenyl ring was refined as disordered. The two disordered moieties were restrained to have similar geometries. U_{ij} components of ADPs for disordered atoms closer to each other than 2.0 Å were restrained to be similar. Subject to these conditions, the occupancy ratio refined to 0.66(2) to 0.34(2).

Four fluorine atoms of a $[PF_6]^-$ anion were refined as disordered. The two disordered moieties were restrained to have similar geometries (under omission of not disordered F atoms and the P atom). U_{ij} components of ADPs for disordered atoms closer to each other than 2.0 Å were restrained to be similar. Subject to these conditions the occupancy ratio refined to 0.810(10) to 0.190(10).

The structure contains additional 683 $Å^3$ of solvent accessible pores. No substantial electron density peaks were found in the solvent accessible pores (less than three electrons per cubic Å) and the residual electron density peaks could not be completely modeled (part of the disordered electron density matches highly disordered chloroform; disorder modelling up to five-fold gave however unsatisfying results). The structure factors were instead augmented via reverse Fourier transform methods using the SQUEEZE routine[13,33] as implemented in the program Platon. The resultant FAB file containing the structure factor contribution from the electron content of the pore space was used together with the original hkl file in the further refinement. (The FAB file with details of the Squeeze results is appended to the deposited cif file). The Squeeze procedure corrected for 236 electrons within the solvent accessible pores.

10: The triflimide anion was refined as disordered over two orientations. The two disordered moieties were restrained to have similar geometries. U_{ij} components of ADPs for disordered atoms closer to each other than 2.0 Å were restrained to be similar. Subject to these conditions the occupancy ratio refined to 0.799(2) to 0.201(2).

11: The structure has four independent units and exhibits pseudo-translations (pseudo-lattice centering and a pseudo-translation along b).

12: The P and Si atoms share a common position. ADPs and position were constrained to be the same. The triflimide ion is disordered around an inversion center. The two equivalent half moieties were restrained to have similar geometries. U_{ij} components of ADPs for C and S atoms were restrained to be similar.

13: One of the two triflimide anions is disordered by pseudo-inversion. The two disordered moieties were restrained to have similar geometries as the other, not disordered triflimide. U_{ij} components of ADPs for disordered atoms closer to each other than 2.0 Å were restrained to be similar. Subject to these conditions the occupancy ratio refined to 0.780(4) to 0.220(4).

14: One triflimide anion was refined as disordered. The two disordered moieties were restrained to have similar geometries as the other not disordered triflimide. U_{ij} components of ADPs for disordered atoms closer to each other than 2.0 Å were restrained to be similar. Subject to these conditions the occupancy ratio refined to 0.8474(15) to 0.1526(15).

15: The structure is extensively disordered. The two $[NTf_2]^-$ anions were refined as five- and three-fold disordered, respectively. All disordered moieties were restrained to have similar geometries. U_{ij} components of ADPs for disordered atoms closer to each other than 2.0 Å were restrained to be similar. Subject to these conditions the occupancy ratio refined to 0.658(3) to 0.153(2) to 0.0498(12) to 0.0908(18) to 0.048(2) for the triflimide of S2/S3, and to 0.8137(15) to 0.1285(16) to 0.0577(15) for the triflimide of S4/S5.

The middle section of the cation (the sulfonyl group and the two adjacent phenylene rings) are disordered by a wiggle motion. Three moieties were refined, which were restrained to have similar geometries. U_{ij} components of ADPs for disordered atoms closer to each other than 2.0 Å were restrained to be similar. Subject to these conditions the occupancy ratio refined to 0.411(3) to 0.485(3) to 0.104(3).

Crystal Images of Representative IL products

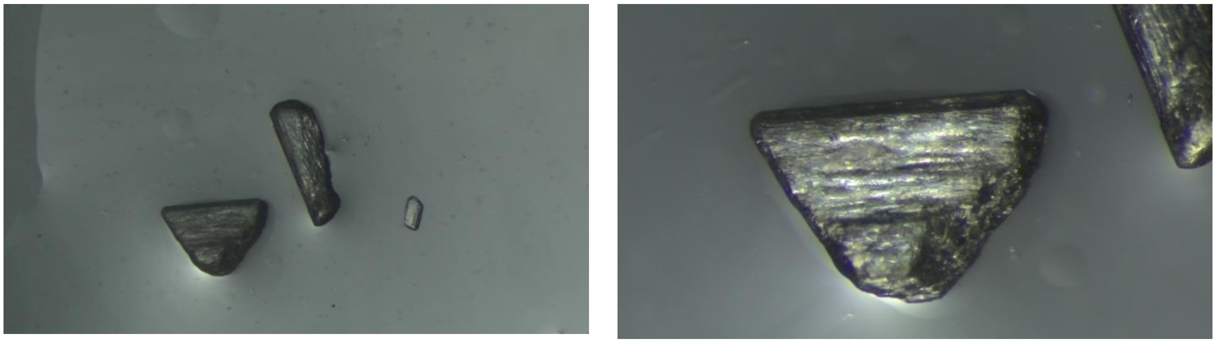

Figure S19. Images of the crystals of IL **6** viewed under a microscope.

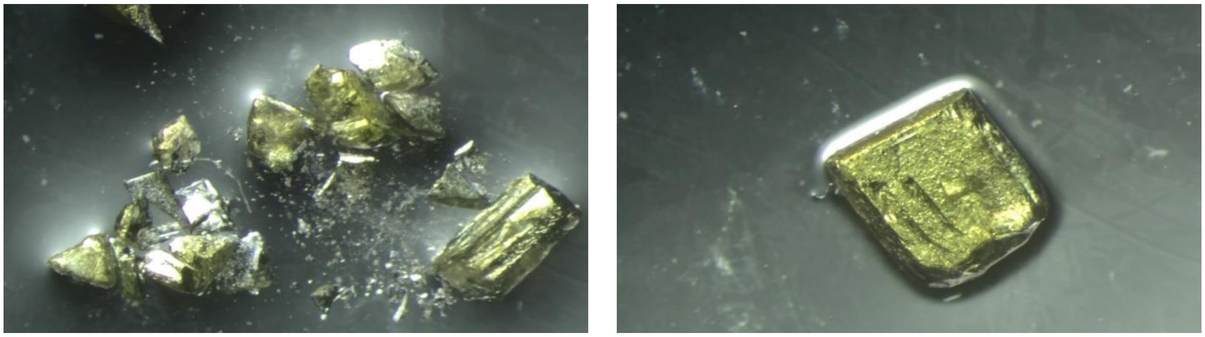

Figure S20. Images of the crystals of IL **7** viewed under a microscope.

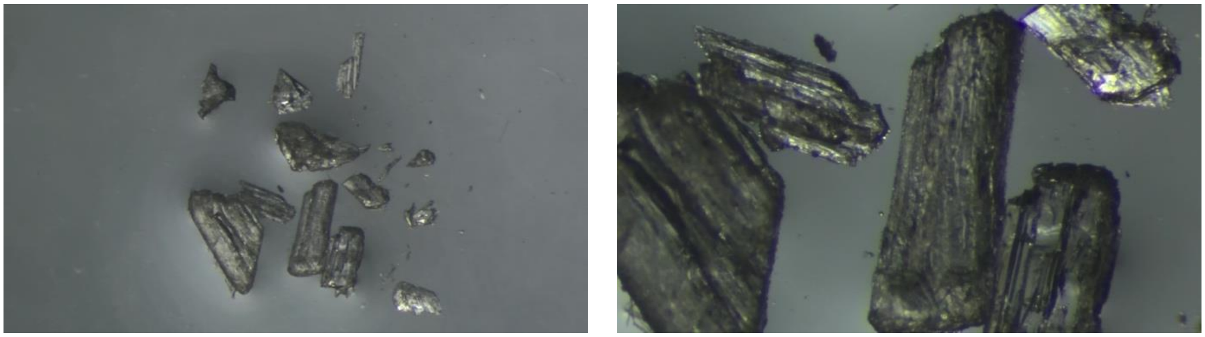

Figure S21. Images of the crystals of IL **13** viewed under a microscope.

Table S1. The calculated interaction energies at the HF 321-G level for the distinct ion pairs in a 10Å cluster for **3a**. The colors represent specific symmetry related molecules to allow for distinction in the calculations. An image with the color-coded molecules is provided in Figure S17.

Color	Label	Count	Distance	Description	Coulomb	Dispersion	Exchange	Polarization	Repulsion	Total
	4	1	5.44	1A : 2A	-209.7	-35.4	-15.2	-51.7	31.2	-266.3
	3	1	6.82	1A : 2A1/2+x,1/2-y,-1/2+z	-169.9	-29.8	-7.3	-27.8	15.6	-211.3
	2	1	7.13	1A : 2A3/2-x,1/2+y,5/2-z	-143.9	-22.5	-4.1	-14.3	10.1	-171.4
	15	1	7.22	2A : 2A1-x,2-y,1-z	97.1	-108	-39.4	-21.1	75.7	17.4
	6	1	8.8	1A : 2A -1/2+x,1/2-y,-1/2+z	-209.4	-23.8	-8.7	-31.9	17.4	-248.5
	5	1	9.25	1A : 2A 1-x,1-y,1-z	-163.6	-11.6	-1.6	-15.4	3.6	-185.6
	18	1	9.49	2A : 2A 1-x,1-y,1-z	136.5	-23.9	-2.2	-13.8	4.8	110.7
	8	2	10.39	2A : 2A -1/2+x,1/2-y,-1/2+z	115.1	-67	-31.1	-15.7	58.1	68.5
	7	2	11.05	2A : 2A-1/2+x,1/2-y,1/2+z	131.3	-12.2	-4.8	-11.5	9.4	119
	23	1	11.05	1A : 2A1-x,2-y,1-z	-114.7	-0.9	0	-5.4	0	-121.2
	9	1	11.2	1A : 2A3/2-x,-1/2+y,5/2-z	-155.6	-5.8	-1	-14.5	2.3	-172.3
	12	2	11.78	2A : 2A 3/2-x,1/2+y,3/2-z	134.7	-12.5	-3.1	-10	6.1	122
	10	1	11.98	1A : 2Ax,y,-1+z	-147.8	-2.6	-0.1	-9.9	0.2	-159.3
	13	1	12.18	1A : 2A-x,1-y,1-z	-121.1	-1.2	0	-3.9	0	-127
	14	2	12.21	2A : 2A3/2-x,1/2+y,5/2-z	105.9	-7.7	-0.4	-6.2	0.9	97.3
	21	1	12.66	2A : 2A-x,2-y,1-z	108.1	-1.7	0	-4.9	0	105.4
	19	1	13.08	1A : 2A1+x,y,z	-87.3	-0.8	0	-2.4	0	-91.3
	30	1	13.61	1A : 2A-x,2-y,1-z	-103.5	-0.3	0	-2.1	0	-107.2
	26	1	13.64	1A : 2A1/2+x,1/2-y,1/2+z	-85.4	-0.5	0	-2.2	0	-88.9
	11	2	13.66	2A : 2A-1+x,y,z	101.5	-9.2	-2.2	-6.8	4.4	92.4

	24	1	13.94	1A : 2A 3/2-x,1/2+y,3/2-z	-99.2	-0.3	0	-1.9	0	-102.5
	39	1	13.98	1A : 2A1-x,1-y,2-z	-91.3	-0.2	0	-1.6	0	-94.2
	16	1	14.08	2A : 2A-x,1-y,1-z	132.6	-6.1	-0.1	-8.9	0.2	124
	28	1	14.15	1A : 2Ax,1+y,z	-81.7	-0.4	0	-1.6	0	-84.6
	25	2	14.6	2A : 2A5/2-x,1/2+y,5/2-z	77.5	-2.1	0	-2.2	0	75.7
	27	2	15.22	2A : 2A5/2-x,1/2+y,3/2-z	85.9	-0.6	0	-2.3	0	85.5
	20	1	15.24	1A : 2A1-x,2-y,2-z	-90	-0.5	0	-3.4	0	-94.3
	46	1	15.33	1A : 2A5/2-x,-1/2+y,5/2-z	-90.7	-0.1	0	-1.5	0	-93.5
	33	2	15.66	2A : 2Ax,-1+y,z	84.4	-0.6	0	-2	0	84.2
	45	1	15.67	1A : 2A-1/2+x,1/2-y,1/2+z	-87.5	-0.1	0	-1.3	0	-90.1
	32	1	15.97	1A : 2A1/2+x,-1/2-y,-1/2+z	-84.7	-0.1	0	-1	0	-87
	31	1	16.17	1A : 2A-1+x,y,z	-98.4	-0.1	0	-1.6	0	-101.4
	29	2	16.36	2A : 2Ax,y,-1+z	85.5	-0.5	0	-2	0	85.3
	44	1	16.4	1A : 2A3/2-x,-1/2+y,3/2-z	-95.7	-0.1	0	-1.4	0	-98.5
	34	1	16.91	1A : 2A-1/2+x,-1/2-y,-1/2+z	-90.1	-0.1	0	-1.2	0	-92.7
	47	1	17.74	1A : 2A1/2-x,1/2+y,5/2-z	-77.1	0	0	-0.7	0	-79.1
	48	2	17.76	2A : 2A1/2+x,3/2-y,1/2+z	68.8	-0.3	0	-1.1	0	69.2
	22	1	17.77	2A : 2A1-x,2-y,2-z	66.7	-1.1	0	-1.3	0	66.2
	17	1	17.81	2A : 2A2-x,2-y,1-z	68.2	-2	0	-1.7	0	66.6
	50	1	18	2A : 2A1-x,2-y,-z	77.8	-0.2	0	-1.2	0	78.4
	35	2	18.15	2A : 2A-1/2+x,3/2-y,1/2+z	67.3	-0.2	0	-0.8	0	67.8
	42	1	18.48	1A : 2A1+x,1+y,z	-64.4	-0.1	0	-0.6	0	-66.1
	37	1	19.02	2A : 2A1-x,1-y,-z	84.3	-0.2	0	-1.4	0	84.8
	43	1	20.09	2A : 2A-x,2-y,-z	77.3	-0.1	0	-1	0	78

	40	2	20.14	2A : 2A1/2+x,-1/2-y,-1/2+z	76.6	-0.1	0	-1	-35.4	48.6
	49	2	20.48	2A : 2A1/2-x,-1/2+y,3/2-z	80.8	-0.1	0	-1.2	0	81.5
	38	2	20.64	2A : 2A-1+x,y,-1+z	67	-0.1	0	-0.9	0	67.6
	36	2	20.78	2A : 2A-1+x,-1+y,z	67	-0.2	0	-0.9	0	67.6
	41	2	21.61	2A : 2A3/2+x,1/2-y,1/2+z	64.7	-0.1	0	-0.7	0	65.3
				**Sum**	**-1207.1**	**-337.35**	**-100.5**	**-287.25**	**182.85**	**-1654.05**
				**Percent of Total**	**73.0%**	**20.4%**	**6.1%**	**17.4%**	**11.1%**	

Table S2. The calculated interaction energies at the HF 321-G level for the distinct ion pairs in a 10Å cluster for **3b**. The colors represent specific symmetry related molecules to allow for distinction in the calculations. An image with the color-coded molecules is provided in Figure S17.

Color	Label	Count	Distance	Description	Coulomb	Dispersion	Exchange	Polarization	Repulsion	Total
	1	1	7.58	1A : 2A1/2+x,1/2-y,-1/2+z	-168.4	-21.3	-7.5	-27.6	15.4	-202.3
	2	1	4.92	1A : 2A	-189.1	-48.5	-9.9	-35	23	-248.5
	3	1	9.03	1A : 2A -1/2+x,1/2-y,-1/2+z	-211.2	-22.3	-8.7	-32	17.8	-248.7
	4	2	11.14	2A : 2A-1/2+x,1/2-y,1/2+z	129.6	-10.9	-3.2	-10.7	6.3	117.8
	5	1	13.89	2A : 2A-x,1-y,1-z	139.2	-9	-1	-11	2.1	127.5
	6	2	11.63	2A : 2A3/2-x,1/2+y,5/2-z	109.8	-12.1	-1.9	-7.7	3.9	97.5
	7	1	12.79	1A : 2A5/2-x,1/2+y,5/2-z	-115.2	-10.3	-4.8	-14.2	9.6	-132
	8	1	10.95	1A : 2A3/2-x,-1/2+y,5/2-z	-158.8	-7.1	-2.4	-16.9	5	-177.2
	9	1	7.13	2A : 2A1-x,2-y,1-z	101.1	-112.8	-47.7	-21	91.8	23.5
	10	1	8.76	1A : 2A 1-x,1-y,1-z	-165.8	-16	-2.2	-16.4	5.2	-191.6
	11	2	14.19	2A : 2A-1+x,y,z	98.7	-7.2	-1.7	-6	3.5	91.6
	12	1	12.53	1A : 2Ax,y,-1+z	-142.4	-1.7	0	-7.9	0	-151.8
	13	1	6.97	1A : 2A3/2-x,1/2+y,5/2-z	-165.2	-19.7	-2.3	-25.3	5.8	-199.8
	14	2	11.86	2A : 2A3/2-x,-1/2+y,3/2-z	134.8	-13.7	-4.3	-10.1	8.4	121.8
	15	1	17.45	2A : 2A1-x,2-y,2-z	67.8	-1.7	0	-1.5	0	66.7
	16	1	10.77	1A : 2A1-x,2-y,1-z	-111.9	-1.1	0	-4.4	0	-117.8
	17	2	10.66	2A : 2A -1/2+x,1/2-y,-1/2+z	111	-67.5	-33.9	-15.3	62.2	65.3
	18	1	9.1	2A : 2A 1-x,1-y,1-z	136	-22.7	-1.1	-15.9	2.6	109
	19	1	12.35	1A : 2A-x,1-y,1-z	-122.9	-1.1	0	-4.1	0	-128.9
	20	1	12.69	2A : 2A-x,2-y,1-z	111.3	-2.3	0	-5.5	0	107.7

	21	1	18.52	2A : 2A2-x,2-y,1-z	65.7	-0.9	0	-1.2	0	65.3
	22	1	15.26	1A : 2A1-x,2-y,2-z	-90	-0.5	0	-3.3	0	-94.3
	23	1	13.82	1A : 2A1+x,y,z	-83.5	-0.5	0	-1.9	0	-86.8
	24	1	13.56	1A : 2A1/2+x,1/2-y,1/2+z	-83.4	-0.6	0	-2	0	-86.8
	25	2	14.68	2A : 2A5/2-x,-1/2+y,5/2-z	77	-2.1	0	-2.1	0	75.2
	26	1	13.18	1A : 2Ax,1+y,z	-87.9	-0.5	0	-2.3	0	-91.6
	27	2	16.42	2A : 2Ax,y,-1+z	84.9	-0.5	0	-2	0	84.7
	28	1	13.82	1A : 2A 3/2-x,1/2+y,3/2-z	-98.4	-0.3	0	-1.8	0	-101.8
	29	1	13.85	1A : 2A-x,2-y,1-z	-99	-0.3	0	-1.8	0	-102.3
	30	2	15.56	2A : 2A5/2-x,1/2+y,3/2-z	84.3	-0.5	0	-2.1	0	84
	31	1	16.12	1A : 2A-1+x,y,z	-96.8	-0.1	0	-1.6	0	-99.7
	32	1	16.22	1A : 2A-1/2+x,-1/2-y,-1/2+z	-98.5	-0.2	0	-1.6	0	-101.6
	33	2	14.86	2A : 2Ax,-1+y,z	88.8	-0.8	0	-2.5	0	88.2
	34	1	16.19	1A : 2A3/2-x,-1/2+y,3/2-z	-91.3	-0.1	0	-1.2	0	-93.9
	35	2	17.7	2A : 2A-1/2+x,3/2-y,1/2+z	69.4	-0.2	0	-0.9	0	69.9
	36	1	15.46	1A : 2A1/2+x,-1/2-y,-1/2+z	-91	-0.1	0	-1.3	0	-93.7
	37	2	20.55	2A : 2A-1+x,-1+y,z	68.2	-0.2	0	-0.9	0	68.7
	38	1	15.13	1A : 2A-1/2+x,1/2-y,1/2+z	-86.5	-0.1	0	-1.3	0	-89.1
	39	1	17.78	1A : 2Ax,-1+y,z	-81.3	-0.1	0	-0.8	0	-83.5
	40	1	18.46	1A : 2A1+x,1+y,z	-66.2	-0.1	0	-0.7	0	-68
	41	1	13.91	1A : 2A1-x,1-y,2-z	-94.6	-0.2	0	-2.1	0	-97.9
	42	2	17.4	2A : 2A1/2+x,3/2-y,1/2+z	70.8	-0.3	0	-1.2	0	71
	43	1	19.19	2A : 2A1-x,1-y,-z	82.8	-0.2	0	-1.4	0	83.3
	44	2	19.42	2A : 2A-1/2+x,-1/2-y,1/2+z	79	-0.1	0	-1.1	0	79.6

	45	1	20.63	2A : 2A-x,2-y,-z	75.7	-0.1	0	-0.9	0	76.5
	46	2	21.21	2A : 2A-1+x,y,-1+z	65.4	-0.1	0	-0.8	0	66
	47	2	22.49	2A : 2A-3/2+x,1/2-y,-1/2+z	62.3	-0.1	0	-0.6	0	63
	48	1	18.34	2A : 2A1-x,2-y,-z	77	-0.1	0	-1.2	0	77.6

**Sum**	**-1275.7**	**-360.75**	**-110.1**	**-299.1**	**220.45**	**-1730.35**
**Percent of Total**	**73.7%**	**20.8%**	**6.4%**	**17.3%**	**12.7%**	

IL	Distance	Description	Coulomb	Dispersion	Exchange	Polarization	Repulsion	Total	Total (HF)
**3a**	5.44	1A : 2A	-211.8	-25.8	-33	-39.1	59.4	-254.8	-266.3
**3b**	4.92	1A : 2A	-195.8	-32.5	-27.2	-28.3	49.9	-238.7	-248.5

Table S3. Energy contributions for a single cation-anion pair in **3a** and **3b** calculated at the CE-1p-B3LYP level of theory. A comparison of the same interaction calculated at the HF level of theory is provided at the end.

Photophysical Experiments

Table S4: Molar extinction coefficients (*ε*) at UV-Vis band maxima (λ_{abs}) for salts **1–15.**

IL	ε/L mol^{-1} cm^{-1} (λ_{abs}/nm)
**1**	63,518 (245 nm)
**2*** [a]	14,747 (279 nm)
**2**	9,937(279 nm)
**3**	71,552 (233 nm); 16,008 (340 nm)
**4**	54,658 (233 nm); 18,125 (268 nm)
**5**	51,329 (233 nm); 27,111 (268 nm)
**6*** [a]	14,363 (251 nm); 1,797 (332 nm)
**6**	8,638 (251 nm); 1,418 (332 nm)
**7**	39,644 (228 nm); 14,320 (319 nm)
**8**	32,974 (227 nm); 29,563 (328 nm)
**9*** [a]	16,519 (335 nm)
**9**	13,481 (335 nm)
**10**	2,106 (336 nm)
**11**	26,154 (252 nm)
**12**	** [b]
**13**	** [b]
**14**	57,833 (248 nm), 10,218 (297 nm)
**15**	78,952 (228 nm), 26,324 (257 nm)

[a] The * indicates a bromide salt; all other salts are $[NTf_2]^-$. [b] These UV-Vis spectra exhibit no distinct peaks or defined shoulders.

Table S5. Summary of fluorescence quantum yields and fluorescence emission maxima for salts **1–15.**

IL	QY (280 nm)	QY (350 nm)	$\lambda_{f,max}$ (nm)[a]
**1**	NF [b]	NF	NF
**2*** [c]	0.031 ± 0.002	—	386 nm (280 nm)
**2**	0.033 ± 0.001	—	386 nm (280 nm)
**3**	—	0. 25 ± 0.02	461 nm (350 nm)
**4**	0.080 ± 0.006	NF	331 nm (280 nm)
**5**	NF	NF	NF
**6*** [c]	NF	NF	NF
**6**	NF	NF	NF
**7**	0.028 ± 0.002	0.031 ± 0.001	445 nm (280 nm); 428 nm (350 nm)
**8**	NF	NF	NF
**9*** [c]	—	0.386 ± 0.006	490 nm (350 nm)
**9**	—	0.417 ± 0.001	490 nm (350 nm)
**10**	—	NF	NF
**11**	—	NF	NF
**12**	—	NF	NF
**13**	0.074 ± 0.011	NF	364 nm (280 nm)
**14**	0.274 ± 0.010	NF	322 nm (280 nm)
**15**	NF	NF	NF

[a] The parenthetical value indicates the excitation wavelength, λ_{ex}. [b] NF denotes essentially non-fluorescent (QY < 0.005) in dilute solution. [c] The * indicates a bromide salt; all other salts are $[NTf_2]^-$.

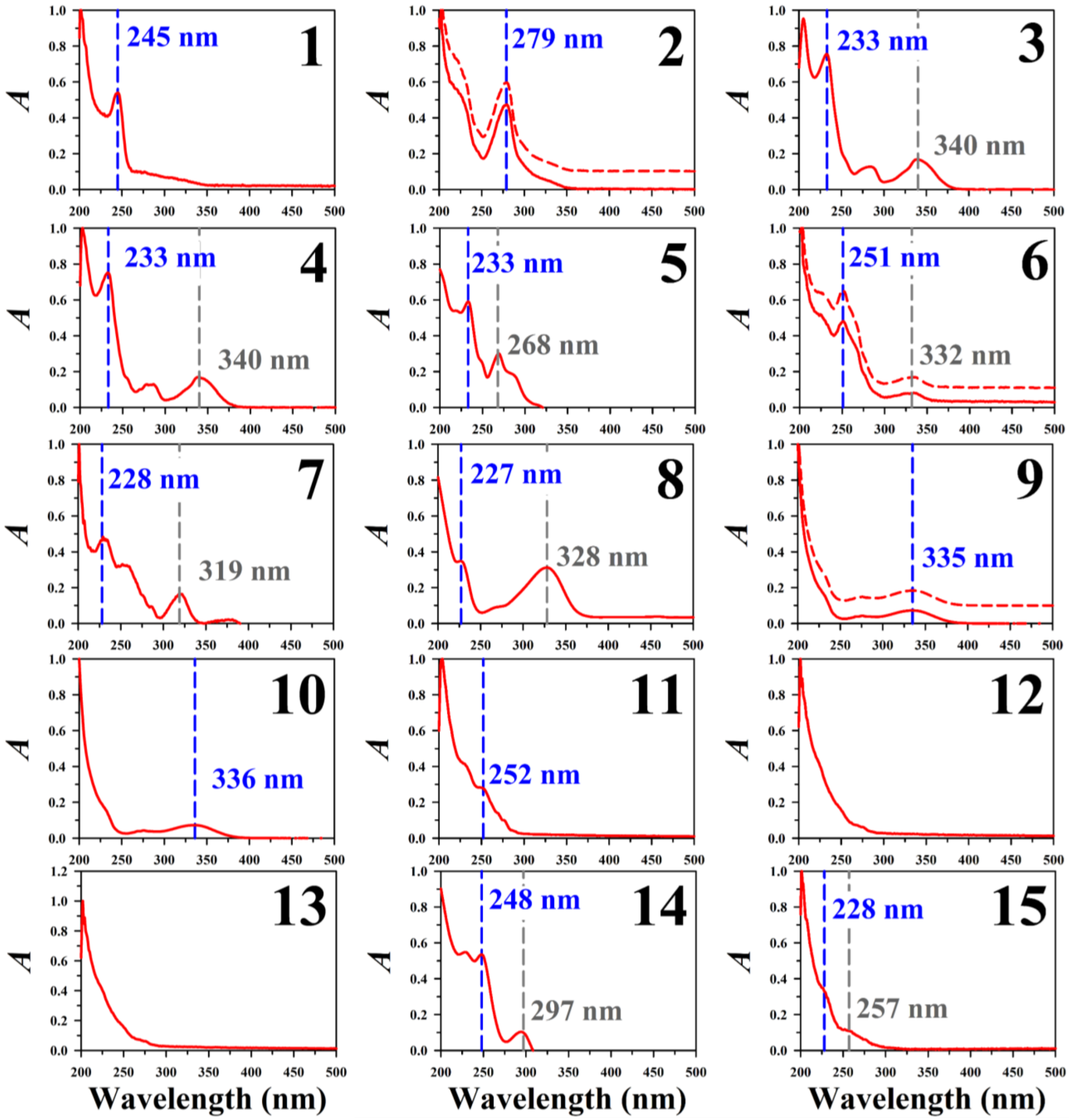

Figure S22. Summary of UV-Vis absorption profiles of triphenylphosphonium salts **1** to **15**, recorded in dilute ethanolic solution. Unless otherwise specified, the data correspond to the $[NTf_2]^-$ salts. Dashed profiles represent the bromide salts for **2**, **6**, and **9**, allowing direct comparison with their $[NTf_2]^-$ counterparts. The wavelengths provided indicate the peak or shoulder maxima.

References

(1) Marcoux, D.; Charette, A. B. Nickel-Catalyzed Synthesis of Phosphonium Salts from Aryl Halides and Triphenylphosphine. *Adv. Synth. Catal.* **2008**, *350*, 2967–2974.

(2) Cassity, C. A.; Siu, B.; Soltani, M.; McGeehee, J. L.; Strickland, K. J.; Vo, M.; Salter, E. A.; Stenson, A. C.; Wierzbicki, A.; West, K. N.; Rabideau, B. D.; Davis, J. H. The Effect of Structural Modifications on the Thermal Stability, Melting Points and Ion Interactions for a Series of TetraarylPhosphonium-Based Mesothermal Ionic Liquids. *Phys. Chem. Chem. Phys.* **2017**, *19*, 31560−31571.

(3) Marcoux, D.; Charette, A. B. Palladium-Catalyzed Synthesis of Functionalized Tetraarylphosphonium Salts. *J. Org. Chem.* **2008**, *73*, 590–593.

(4) Goodson, F. E.; Wallow, T. I.; Novak, B. M. Mechanistic Studies on the Aryl−Aryl Interchange Reaction of ArPdL2I (L = Triarylphosphine) Complexes. *J. Am. Chem. Soc.* **1997**, *119*, 12441−12453.

(5) Krause, L.; Herbst-Irmer, R.; Sheldrick, G. M.; Stalke, D. Comparison of Silver and Molybdenum Microfocus X-Ray Sources for Single-Crystal Structure Determination. *J. Appl. Cryst.* **2015**, *48*, 3−10.

(6) Bruker. Apex5 v2023.9-2, SAINT V8.40B, **2023**.

(7) Sheldrick, G. M. A Short History of SHELX. *Acta Cryst. A* **2008**, *64*, 112−122.

(8) Sheldrick, G. M., SHELXT – Integrated Space-Group and Crystal-Structure Determination. *Acta Cryst. A* **2015**, *71*, 3–8.

(9) Sheldrick, G. M. Crystal Structure Refinement with SHELXL. *Acta Cryst. C* **2015**, *71*, 3−8.

(10) Hübschle, C. B.; Sheldrick, G. M.; Dittrich, B. ShelXle: A Qt Graphical User Interface for SHELXL. *J. Appl. Cryst.* **2011**, *44*, 1281–1284.

(11) Parsons, S., Flack, H., Wagner, T. Use of Intensity Quotients and Differences in Absolute Structure Refinement. *Acta Cryst. B* **2013**, *69*, 249–259.

(12) Spackman, P. R.; Turner, M. J.; McKinnon, J. J.; Wolff, S. K.; Grimwood, D. J.; Jayatilaka, D.; Spackman, M. A. *CrystalExplorer*: A Program for Hirshfeld Surface Analysis, Visualization and Quantitative Analysis of Molecular Crystals. *J. Appl. Crystallogr.* **2021**, *54*, 1006−1011.

(13) Spek, A. L. PLATON SQUEEZE: A Tool for the Calculation of the Disordered Solvent Contribution to the Calculated Structure Factors. *Acta Cryst. C* **2015**, *71*, 9–18

(14) Scheuren, M.; Teodoro, L.; Witters, A.; Musozoda, M.; Adu, C.; Guillet, G.; Freeze, R.; Zeller, M.; Mirjafari, A.; Hillesheim, P. C. Planting the Seeds of a Decision Tree for Ionic Liquids: Steric and Electronic Impacts on Melting Points of Triarylphosponium Ionic Liquids. *J. Phys. Chem. B* **2024**, *128*, 5895−5907.

(15) Philippi, F.; Pugh, D.; Rauber, D.; Welton, T.; Hunt, P. A. Conformational Design Concepts for Anions in Ionic Liquids. *Chem. Sci.* **2020**, *11*, 6405–6422.

(16) Canongia Lopes, J. N.; Shimizu, K.; Pádua, A. A. H.; Umebayashi, Y.; Fukuda, S.; Fujii, K.; Ishiguro, S. A Tale of Two Ions: The Conformational Landscapes of Bis(Trifluoromethanesulfonyl)Amide and *N*,*N*-Dialkylpyrrolidinium. *J. Phys. Chem. B* **2008**, *112*, 1465−1472.

(17) Hunt, P. A.; Ashworth, C. R.; Matthews, R. P. Hydrogen Bonding in Ionic Liquids. *Chem. Soc. Rev.* **2015**, *44*, 1257−1288.

(18) Matthews, R. P.; Welton, T.; Hunt, P. A. Hydrogen Bonding and π–π Interactions in Imidazolium-Chloride Ionic Liquid Clusters. *Phys. Chem. Chem. Phys.* **2015**, *17*, 14437–14453.
(19) Matthews, R. P.; Welton, T.; Hunt, P. A. Competitive π Interactions and Hydrogen Bonding within Imidazolium Ionic Liquids. *Phys. Chem. Chem. Phys.* **2014**, *16*, 3238–3253.
(20) Teodoro, L. I.; Bellia, S. A.; Zeller, M.; Hillesheim, P. C. Examining the Interactions of a Thermally Robust Task-Specific Phosphonium-Based Ionic Compound. *Chem. Data Collect.* **2021**, *35*, 100760.
(21) O'Rourke, B.; Lauderback, C.; Teodoro, L. I.; Grimm, M.; Zeller, M.; Mirjafari, A.; Guillet, G. L.; Hillesheim, P. C. Developing Structural First Principles for Alkylated Triphenylphosphonium-Based Ionic Liquids. *ACS Omega* **2021**, *6*, 32285–32296.
(22) Martinez, C. R.; Iverson, B. L. Rethinking the Term "Pi-Stacking." *Chem. Sci.* **2012**, *3*, 2191.
(23) Cruz-Cabeza, A. J.; Spackman, P. R.; Hall, A. V. The Interplay between Hydrogen Bonds and Stacking/T-Type Interactions in Molecular Cocrystals. *Commun. Chem.* **2024**, *7*, 1–9.
(24) Gavezzotti, A. Crystal Polymorphism: Conventional and Real Wisdom. In *Theoretical and Computational Chemistry*; Gavezzotti, A., Ed.; Elsevier: Amsterdam, 2021; Vol. 20, pp 143−168.
(25) Mackenzie, C. F.; Spackman, P. R.; Jayatilaka, D.; Spackman, M. A. CrystalExplorer Model Energies and Energy Frameworks: Extension to Metal Coordination Compounds, Organic Salts, Solvates and Open-Shell Systems. *IUCrJ* **2017**, *4*, 575−587.
(26) Spackman, P. R.; Spackman, M. A.; Gale, J. D. A Transferable Quantum Mechanical Energy Model for Intermolecular Interactions Using a Single Empirical Parameter. *IUCrJ* **2023**, *10*, 754−765.
(27) Spackman, M. A. Towards the Use of Experimental Electron Densities to Estimate Reliable Lattice Energies. *CrystEngComm* **2018**, *20*, 5340−5347.
(28) Spackman, M. A.; Spackman, P. R.; Thomas, S. P. Beyond Hirshfeld Surface Analysis: Interaction Energies, Energy Frameworks and Lattice Energies with CrystalExplorer. In *Complementary Bonding Analysis*; Grabowsky, S., Ed.; De Gruyter: Berlin, 2021; pp 329−352.
(29) Gavezzotti, A. The "Sceptical Chymist": Intermolecular Doubts and Paradoxes. *CrystEngComm* **2013**, *15*, 4027−4035.
(30) Dong, K.; Song, Y.; Liu, X.; Cheng, W.; Yao, X.; Zhang, S. Understanding Structures and Hydrogen Bonds of Ionic Liquids at the Electronic Level. *J. Phys. Chem. B* **2012**, *116*, 1007−1017.
(31) Saher, S.; Piper, S. L.; Forsyth, C. M.; Kar, M.; MacFarlane, D. R.; Pringle, J. M.; Matuszek, K. Investigation of the Intermolecular Origins of High and Low Heats of Fusion in Azolium Salt Phase Change Materials for Thermal Energy Storage. *Mater. Adv.* **2024**, *5*, 2991−3000.
(32) Macrae, C. F.; Sovago, I.; Cottrell, S. J.; Galek, P. T. A.; McCabe, P.; Pidcock, E.; Platings, M.; Shields, G. P.; Stevens, J. S.; Towler, M.; Wood, P. A. Mercury 4.0: From Visualization to Analysis, Design and Prediction. *J. Appl. Crystallogr.* **2020**, *53*, 226−235.
(33) van der Sluis, P.; Spek, A. L. BYPASS: An Effective Method for the Refinement of Crystal Structures Containing Disordered Solvent Regions. *Acta Crystallogr. A* **1990**, *46*, 194−201.

^{1}H, ^{13}C and ^{31}P NMR Spectra of the Product

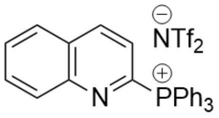

1

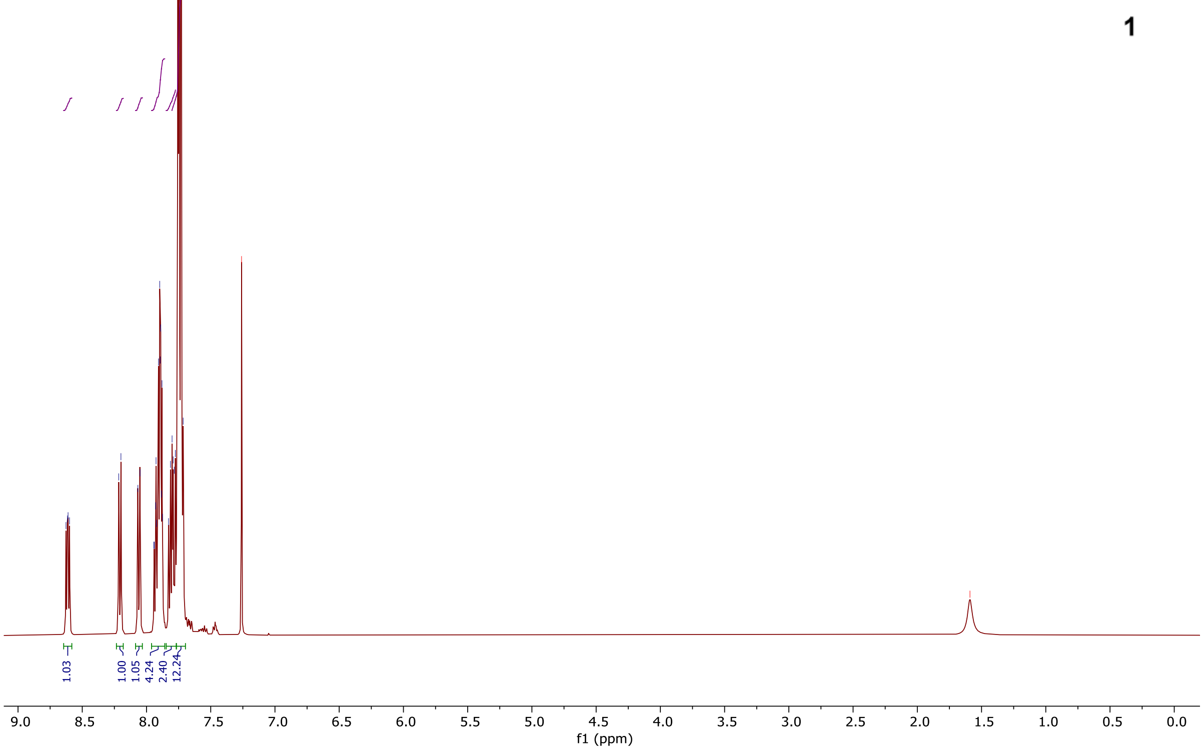

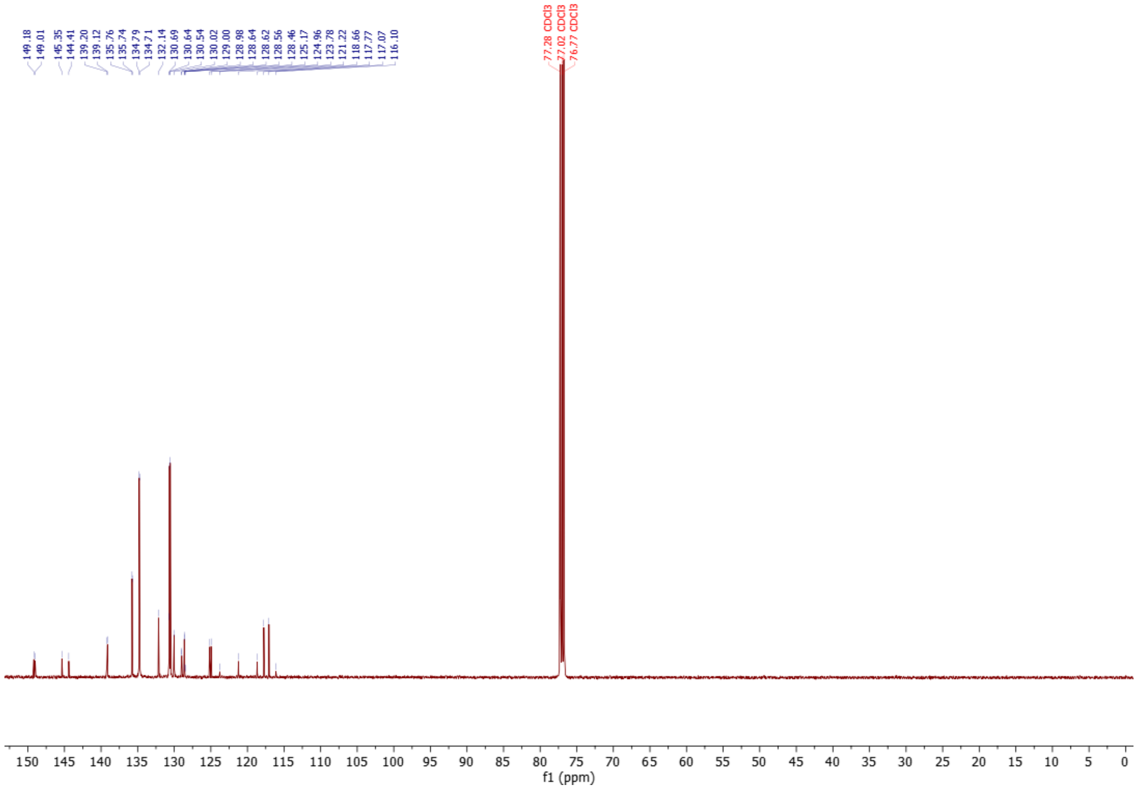

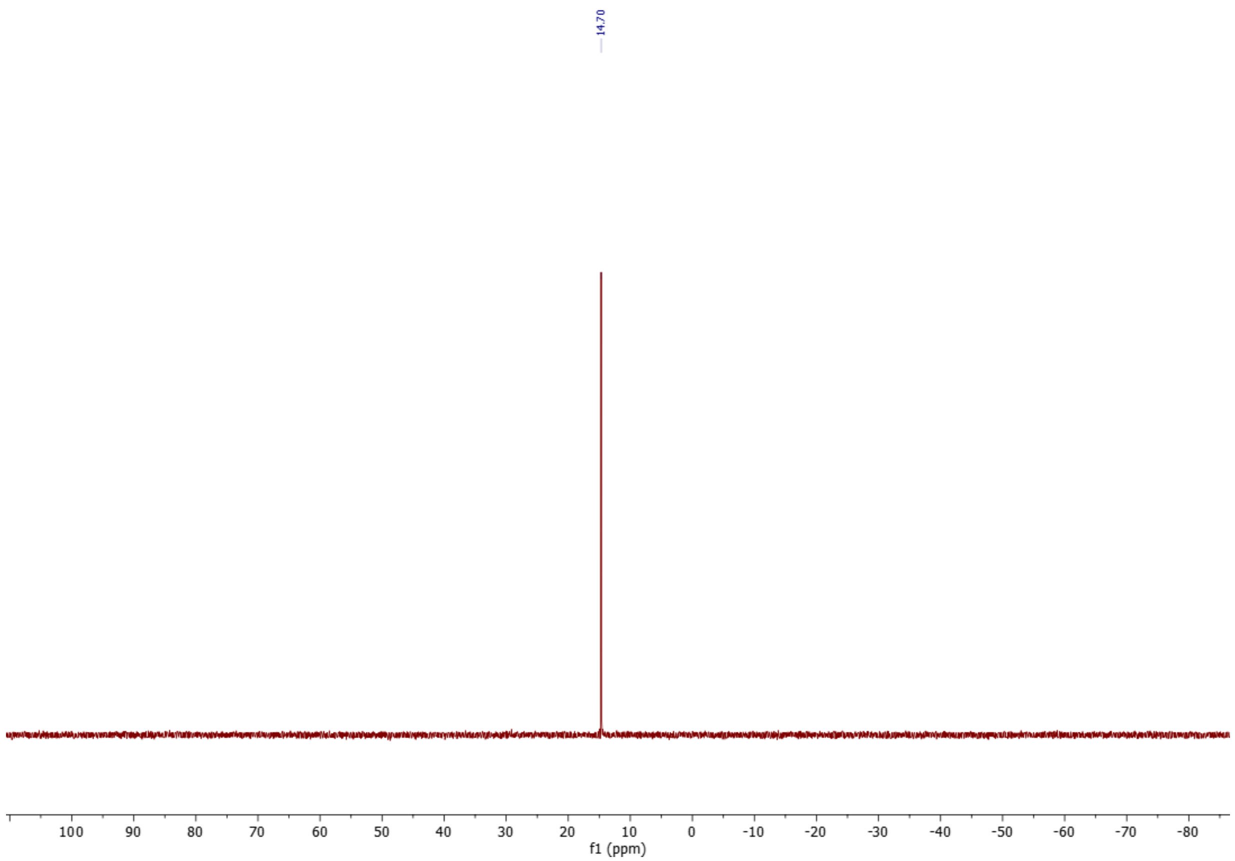
14.70
100 90 80 70 60 50 40 30 20 10 0 -10 -20 -30 -40 -50 -60 -70 -80
f1 (ppm)

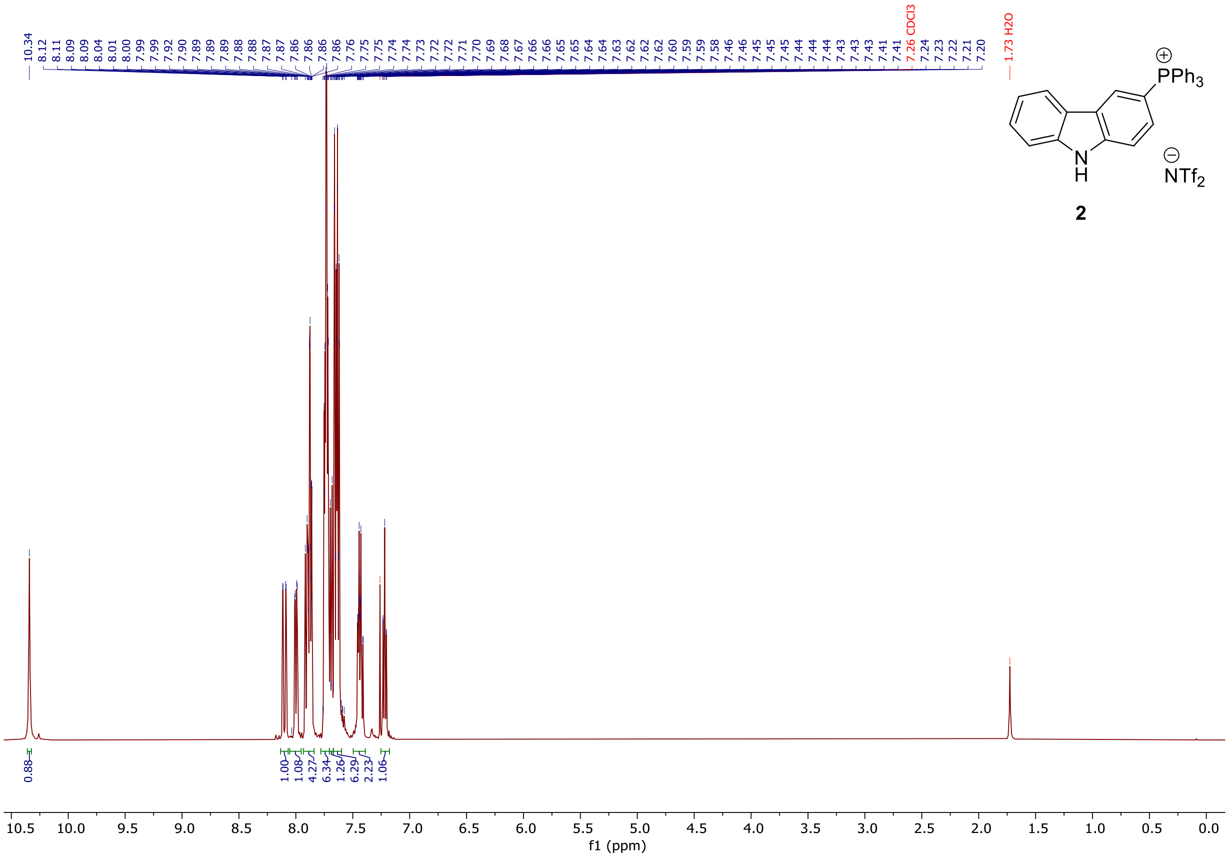
10.34 8.12 8.11 8.09 8.09 8.04 8.01 8.00 7.99 7.99 7.92 7.90 7.89 7.89 7.89 7.88 7.87 7.87 7.86 7.86 7.86 7.76 7.75 7.75 7.74 7.74 7.73 7.72 7.72 7.71 7.70 7.69 7.68 7.67 7.66 7.66 7.65 7.65 7.64 7.63 7.62 7.62 7.62 7.60 7.59 7.59 7.58 7.46 7.46 7.45 7.45 7.44 7.44 7.44 7.43 7.43 7.43 7.41 7.26 CDCl3 7.24 7.23 7.22 7.21 7.20
1.73 H2O
0.88 1.00 1.08 4.27 6.34 1.26 6.29 2.23 1.06
10.5 10.0 9.5 9.0 8.5 8.0 7.5 7.0 6.5 6.0 5.5 5.0 4.5 4.0 3.5 3.0 2.5 2.0 1.5 1.0 0.5 0.0
f1 (ppm)
PPh3
N
H
NTf2

2

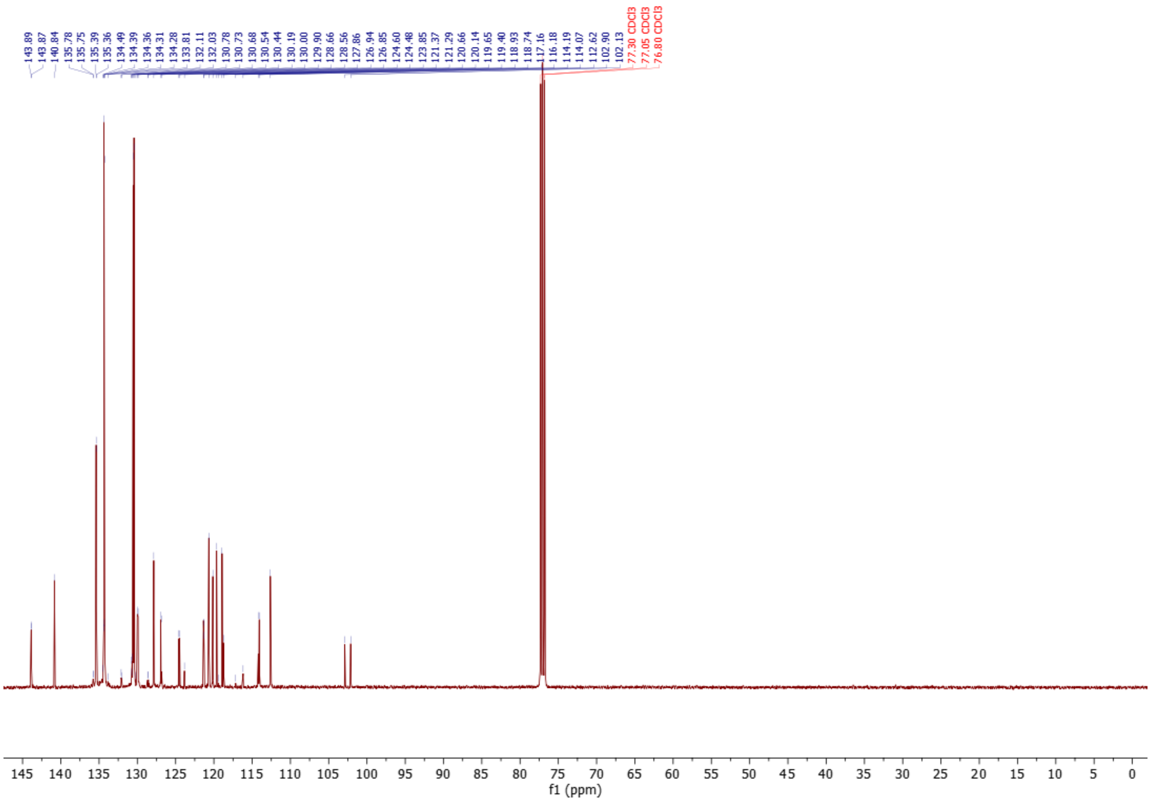
143.89 143.87 140.84 135.78 135.75 135.39 135.36 134.49 134.39 134.36 134.31 134.28 133.81 132.11 132.03 130.78 130.73 130.68 130.54 130.44 130.19 130.00 129.90 128.66 128.56 127.86 126.94 126.85 124.60 124.48 123.85 121.37 121.29 120.66 120.14 119.65 119.40 118.93 118.74 117.16 116.18 114.19 114.07 112.62 102.90 102.13
77.30 CDCl3 77.05 CDCl3 76.80 CDCl3
145 140 135 130 125 120 115 110 105 100 95 90 85 80 75 70 65 60 55 50 45 40 35 30 25 20 15 10 5 0
f1 (ppm)

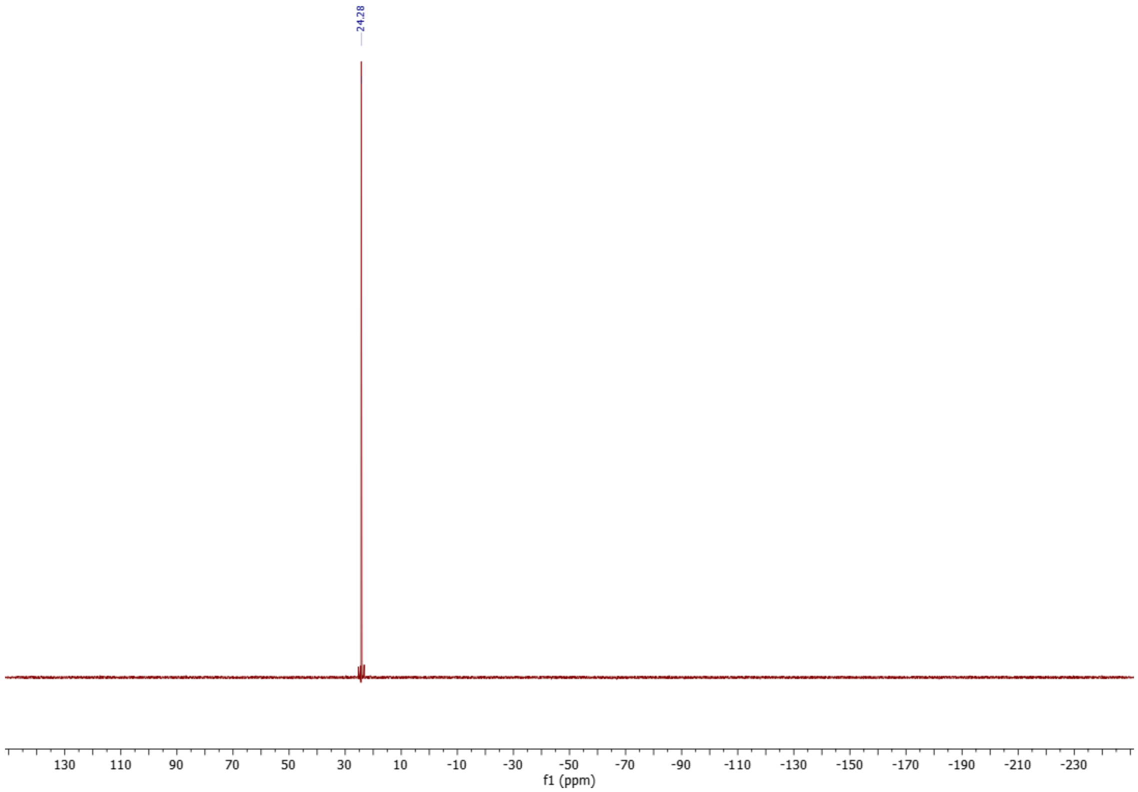
24.28
130 110 90 70 50 30 10 -10 -30 -50 -70 -90 -110 -130 -150 -170 -190 -210 -230
f1 (ppm)

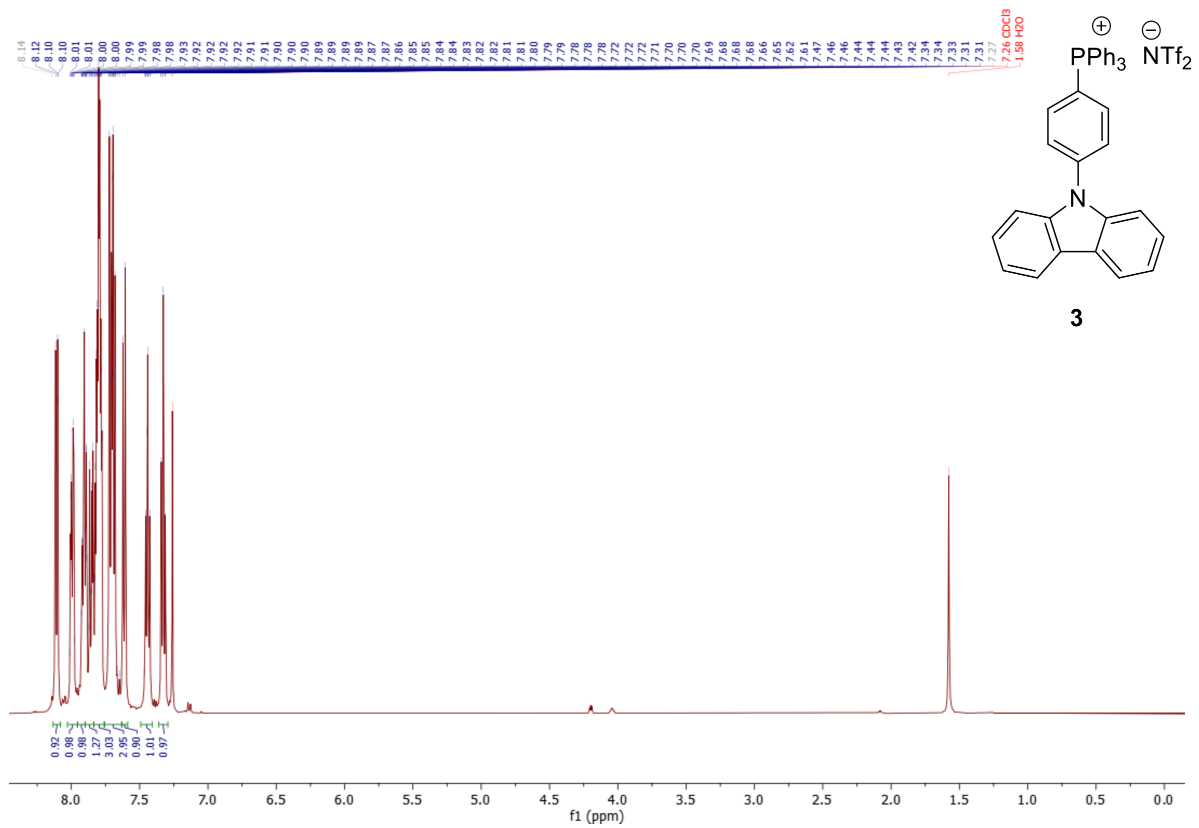

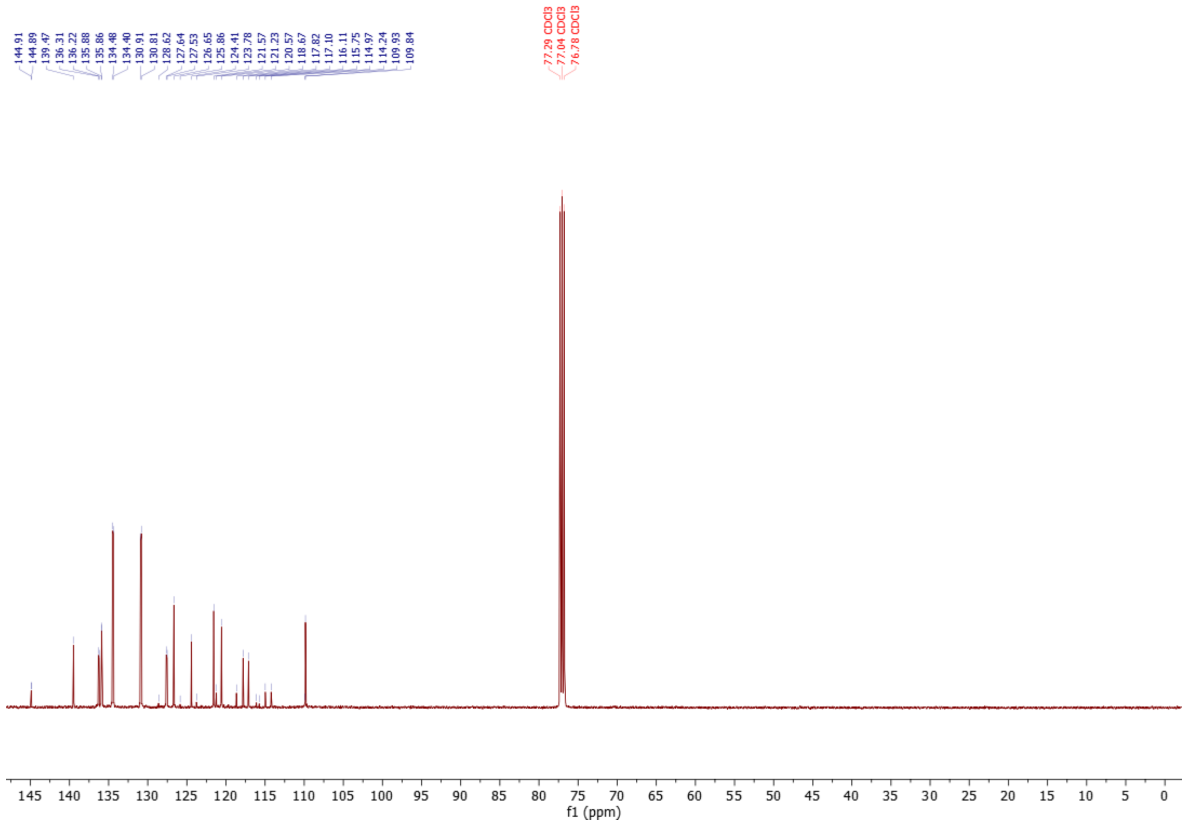

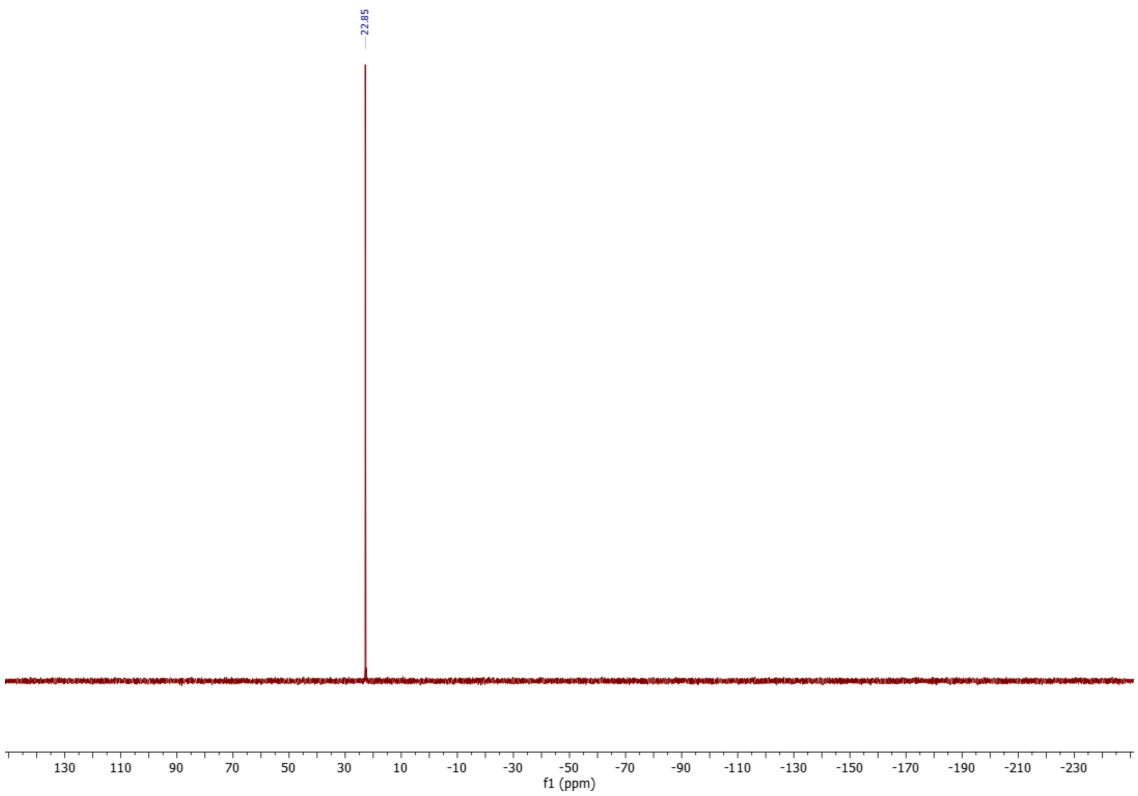
22.85
130 110 90 70 50 30 10 -10 -30 -50 -70 -90 -110 -130 -150 -170 -190 -210 -230
f1 (ppm)

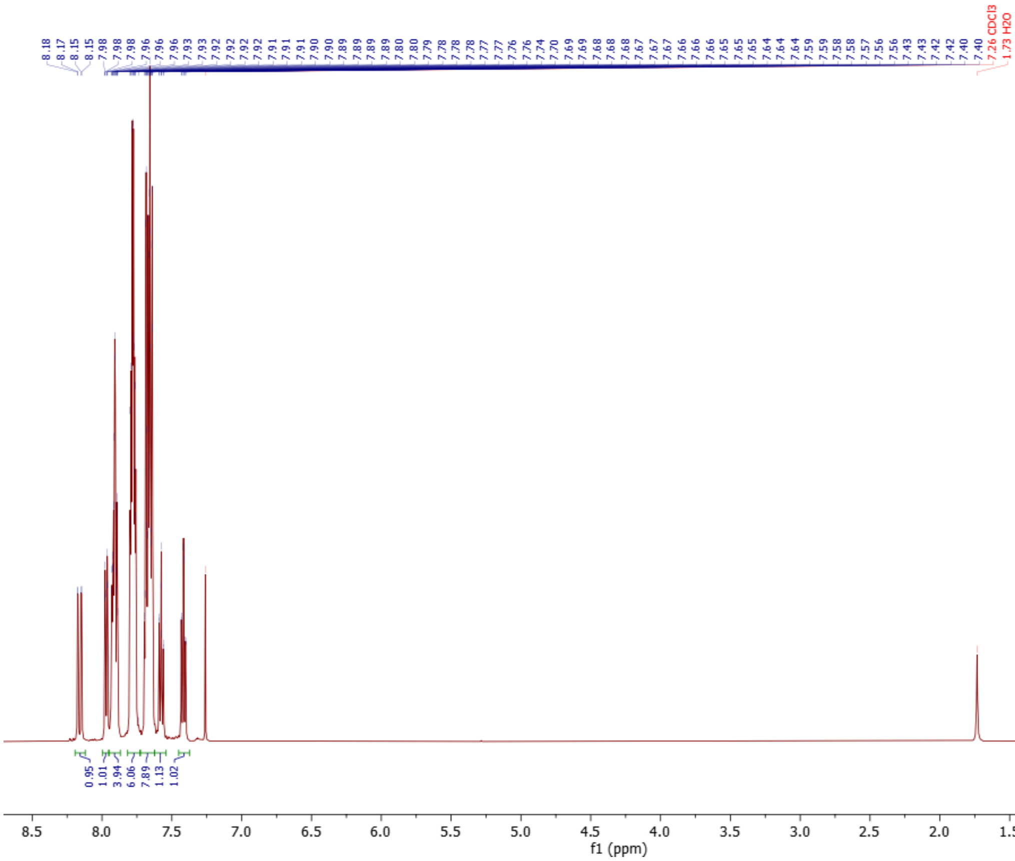
8.18
8.17
8.15
8.15
7.98
7.98
7.98
7.96
7.96
7.96
7.93
7.93
7.92
7.92
7.92
7.92
7.91
7.91
7.91
7.90
7.90
7.89
7.89
7.89
7.89
7.80
7.80
7.79
7.78
7.78
7.78
7.77
7.76
7.76
7.74
7.70
7.69
7.69
7.68
7.68
7.67
7.67
7.67
7.66
7.66
7.66
7.65
7.65
7.65
7.64
7.64
7.59
7.59
7.58
7.58
7.57
7.56
7.56
7.43
7.43
7.42
7.42
7.40
7.40
7.26 CDCl3
1.73 H2O
0.95
1.01
3.94
6.06
7.89
1.13
1.02
8.5 8.0 7.5 7.0 6.5 6.0 5.5 5.0 4.5 4.0 3.5 3.0 2.5 2.0 1.5 1.0 0.5 0.0
f1 (ppm)

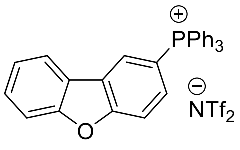
PPh3
NTf2
O

4

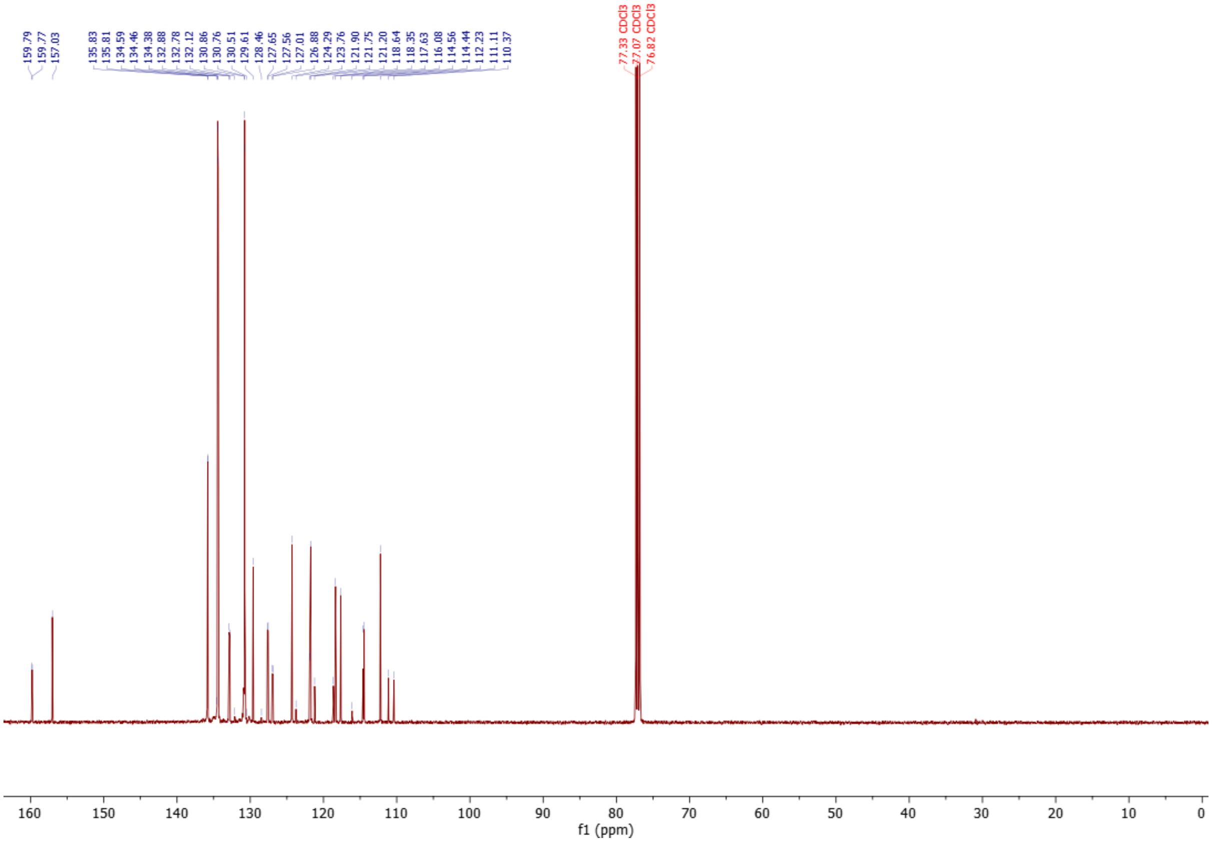
159.79
159.77
157.03
135.83
135.81
134.59
134.46
134.38
132.88
132.78
132.12
130.86
130.76
130.51
129.61
128.46
127.65
127.56
127.01
126.88
124.29
123.76
121.90
121.75
121.20
118.64
118.35
117.63
116.08
114.56
114.44
112.23
111.11
110.37
77.33 CDCl3
77.07 CDCl3
76.82 CDCl3
160 150 140 130 120 110 100 90 80 70 60 50 40 30 20 10 0
f1 (ppm)

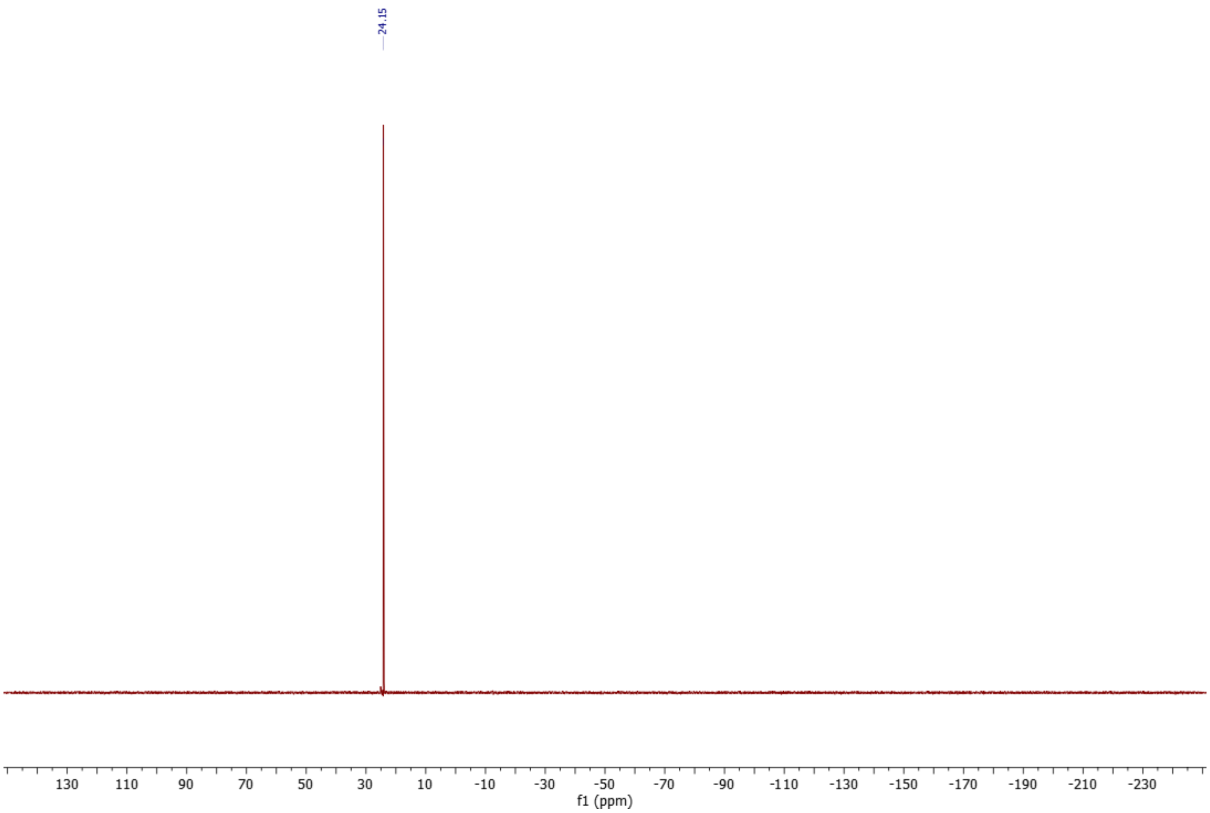
24.15
130
110
90
70
50
30
10
-10
-30
-50
-70
-90
-110
-130
-150
-170
-190
-210
-230
f1 (ppm)

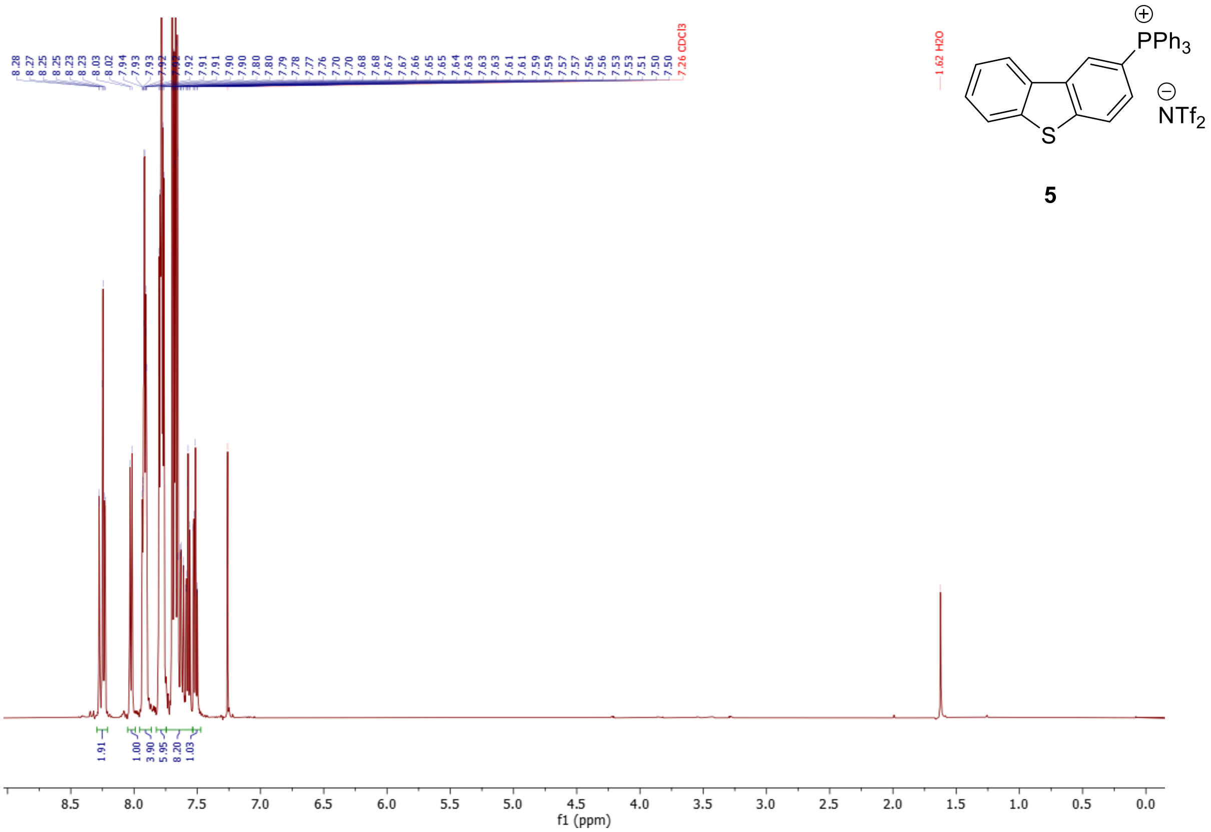
8.28 8.27 8.25 8.25 8.23 8.23 8.23 8.03 8.02 7.94 7.93 7.93 7.92 7.92 7.92 7.91 7.91 7.90 7.90 7.80 7.80 7.79 7.78 7.77 7.76 7.70 7.68 7.68 7.67 7.67 7.66 7.65 7.65 7.64 7.63 7.63 7.63 7.61 7.61 7.59 7.59 7.57 7.57 7.56 7.56 7.53 7.53 7.51 7.50 7.50
7.26 CDCl3
1.62 H2O
1.91
1.00
3.90
5.95
8.20
1.03
f1 (ppm)
PPh3
NTf2
S
5

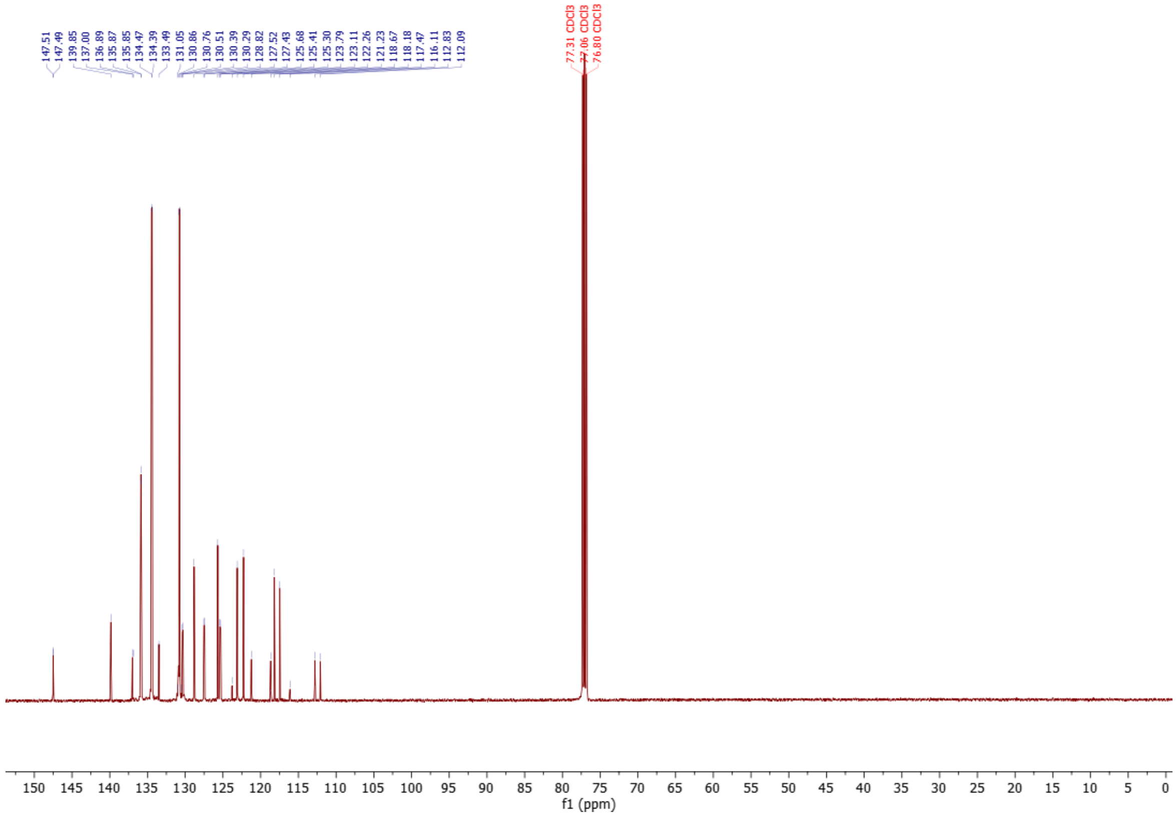
147.51 147.49 139.85 137.00 136.89 135.87 135.85 134.47 134.39 133.49 131.05 130.86 130.76 130.51 130.39 130.29 128.82 127.52 127.43 125.68 125.41 125.30 123.79 123.11 122.26 121.23 118.67 118.18 117.47 116.11 112.83 112.09
77.31 CDCl3
77.06 CDCl3
76.80 CDCl3
f1 (ppm)

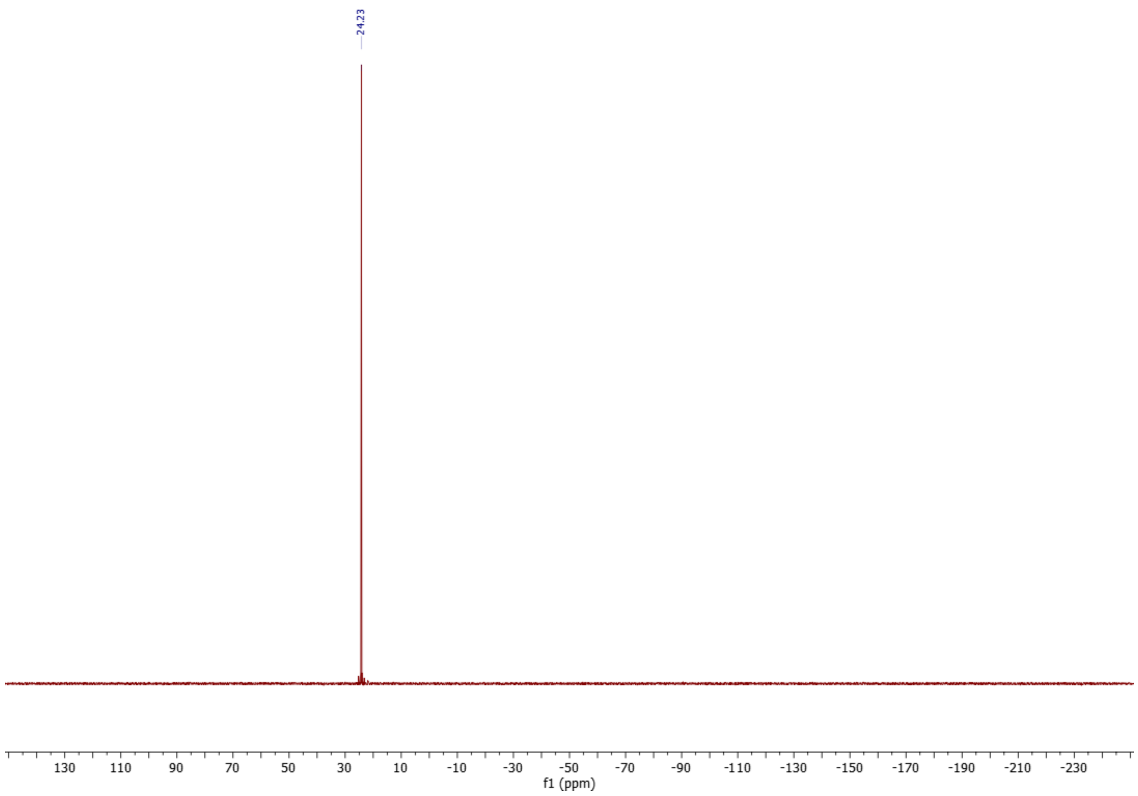
24.23
130 110 90 70 50 30 10 -10 -30 -50 -70 -90 -110 -130 -150 -170 -190 -210 -230
f1 (ppm)

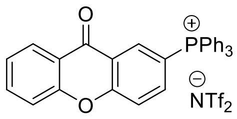
O
PPh3
NTf2

6

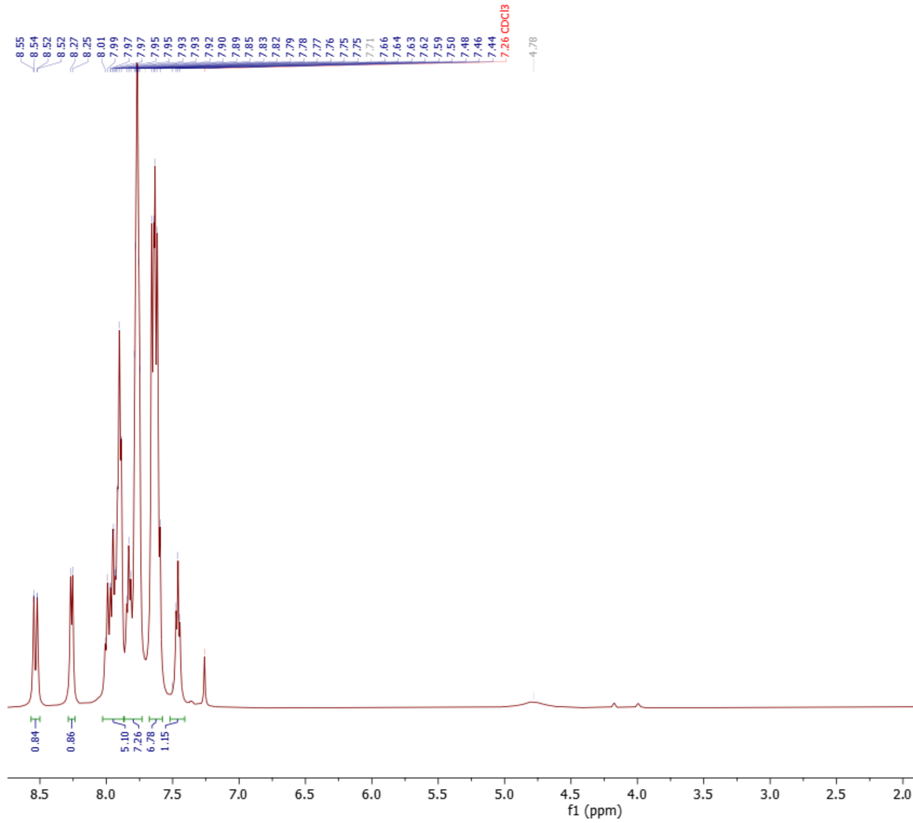
8.55 8.54 8.52 8.52 8.27 8.25 8.01 7.99 7.97 7.97 7.95 7.95 7.93 7.93 7.92 7.90 7.89 7.85 7.83 7.82 7.79 7.78 7.77 7.76 7.75 7.75 7.71 7.66 7.64 7.63 7.62 7.59 7.50 7.48 7.46 7.44
7.26 CDCl3
4.78
1.75 H2O
0.84
0.86
5.10
7.26
6.78
1.15
f1 (ppm)

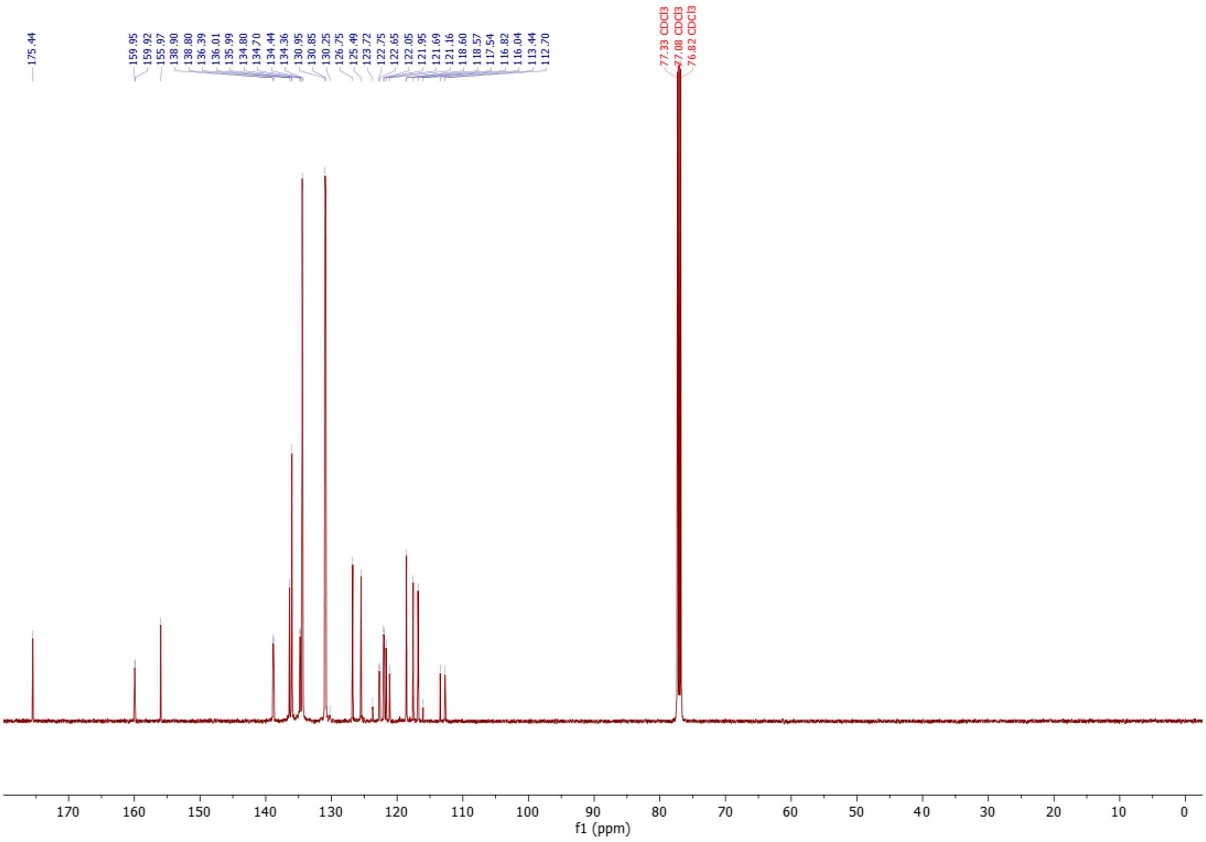
175.44
159.95 159.92 155.97 138.90 138.80 136.39 136.01 135.99 134.80 134.70 134.44 134.36 130.95 130.85 130.25 126.75 125.49 123.72 122.75 122.65 122.05 121.95 121.69 121.16 118.60 118.57 117.54 116.82 116.04 113.44 112.70
77.33 CDCl3
77.08 CDCl3
76.82 CDCl3
f1 (ppm)

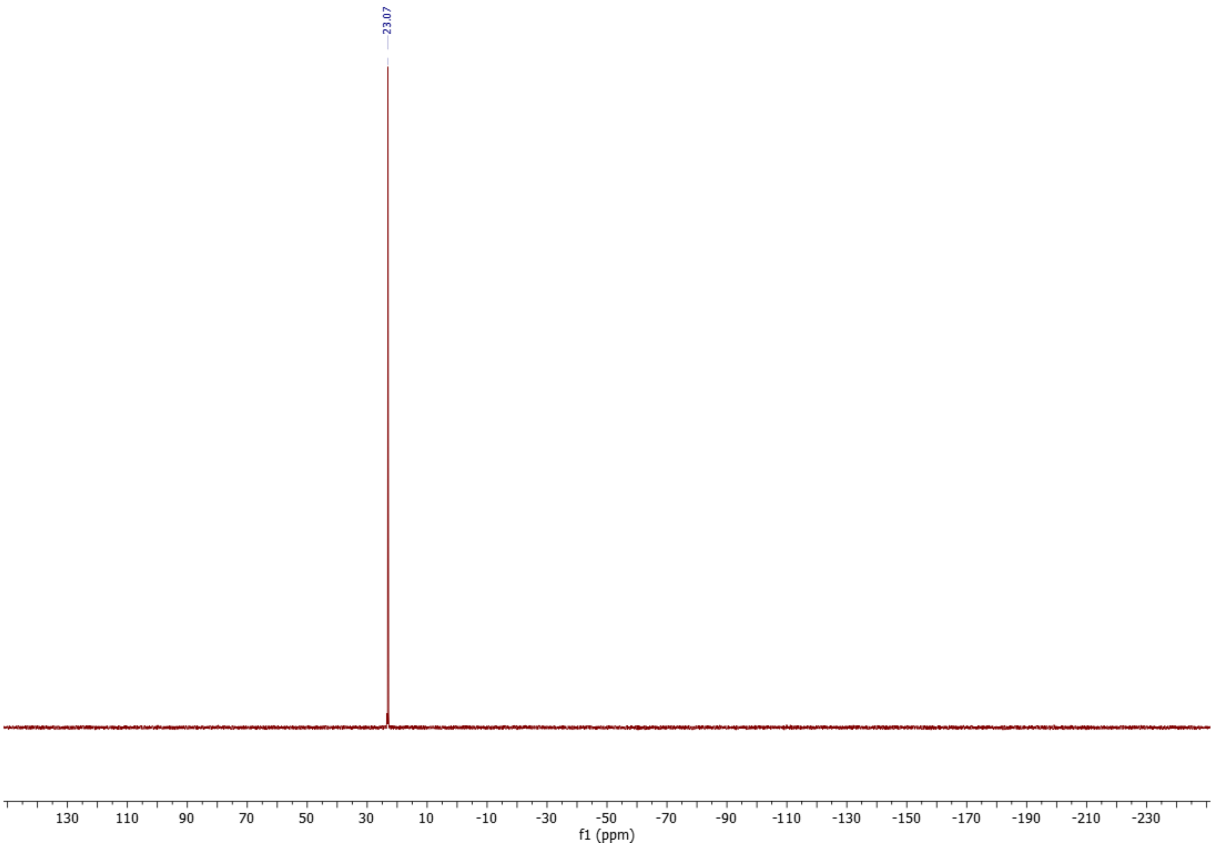
23.07
130
110
90
70
50
30
10
-10
-30
-50
-70
-90
-110
-130
-150
-170
-190
-210
-230
f1 (ppm)

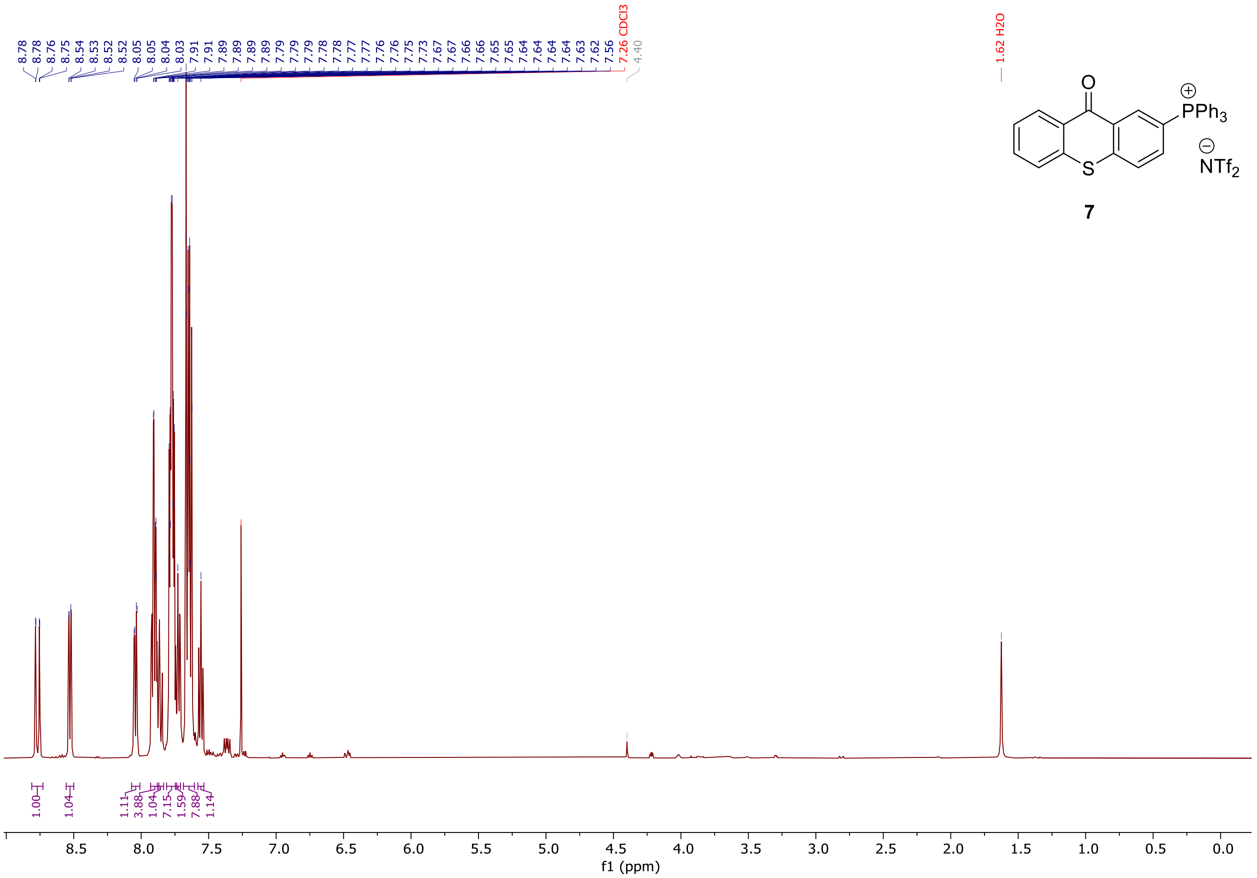
8.78 8.78 8.76 8.75 8.54 8.53 8.52 8.52 8.05 8.05 8.04 8.03 7.91 7.91 7.89 7.89 7.89 7.79 7.79 7.79 7.78 7.78 7.77 7.77 7.76 7.76 7.75 7.73 7.67 7.67 7.66 7.65 7.65 7.64 7.64 7.64 7.63 7.62 7.56
7.26 CDCl3
4.40
1.62 H2O
1.00 1.04 1.11 3.88 1.04 7.15 1.59 7.88 1.14
f1 (ppm)

7

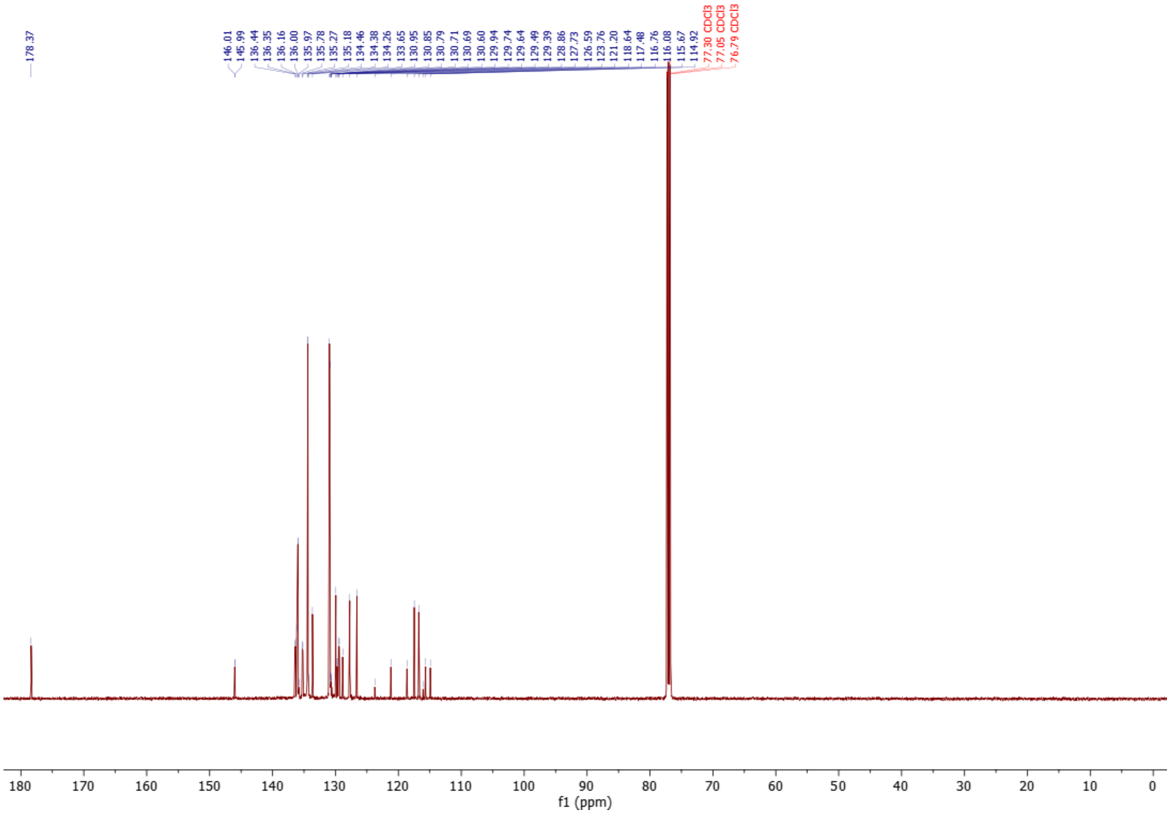
178.37
146.01 145.99 136.44 136.35 136.16 136.00 135.97 135.78 135.27 135.18 134.46 134.38 134.26 133.65 130.95 130.85 130.79 130.71 130.69 130.60 129.94 129.74 129.64 129.49 129.39 128.86 127.73 126.59 123.76 121.20 118.64 117.48 116.76 116.08 115.67 114.92
77.30 CDCl3
77.05 CDCl3
76.79 CDCl3
f1 (ppm)

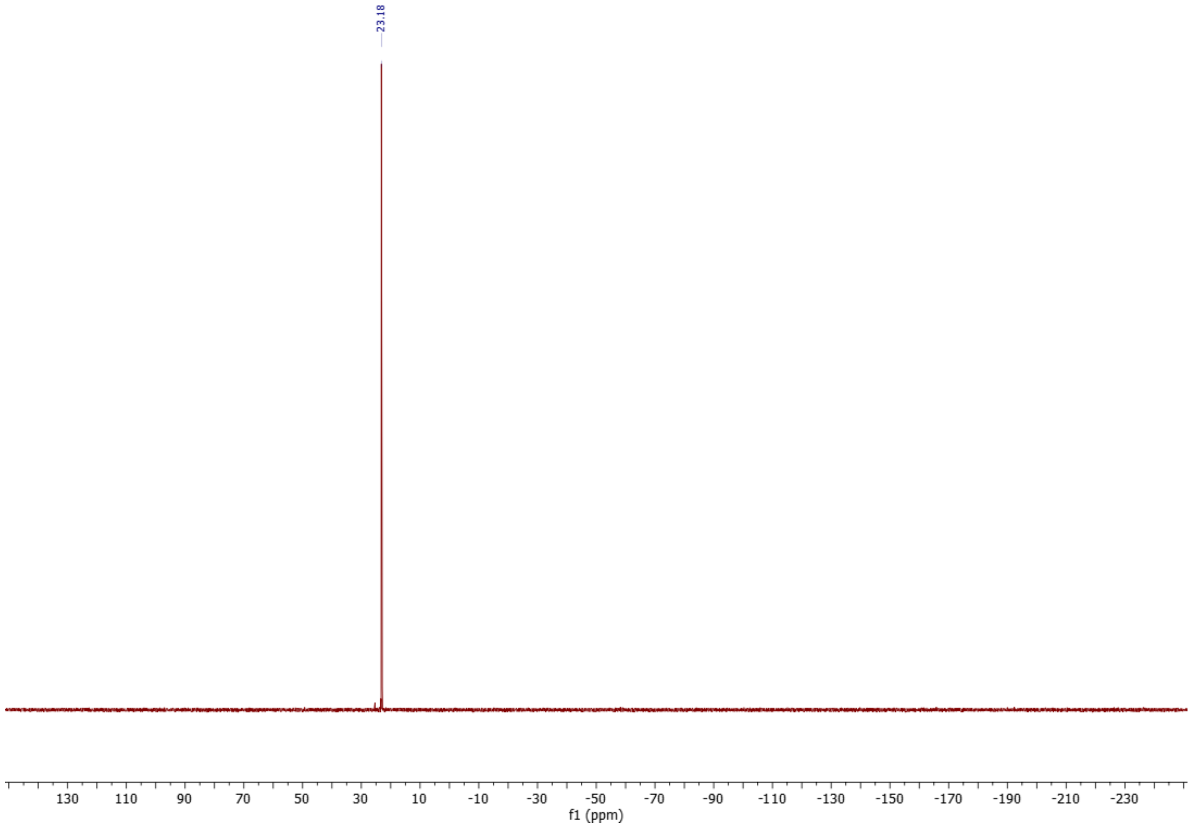
23.18
130
110
90
70
50
30
10
-10
-30
-50
-70
-90
-110
-130
-150
-170
-190
-210
-230
f1 (ppm)

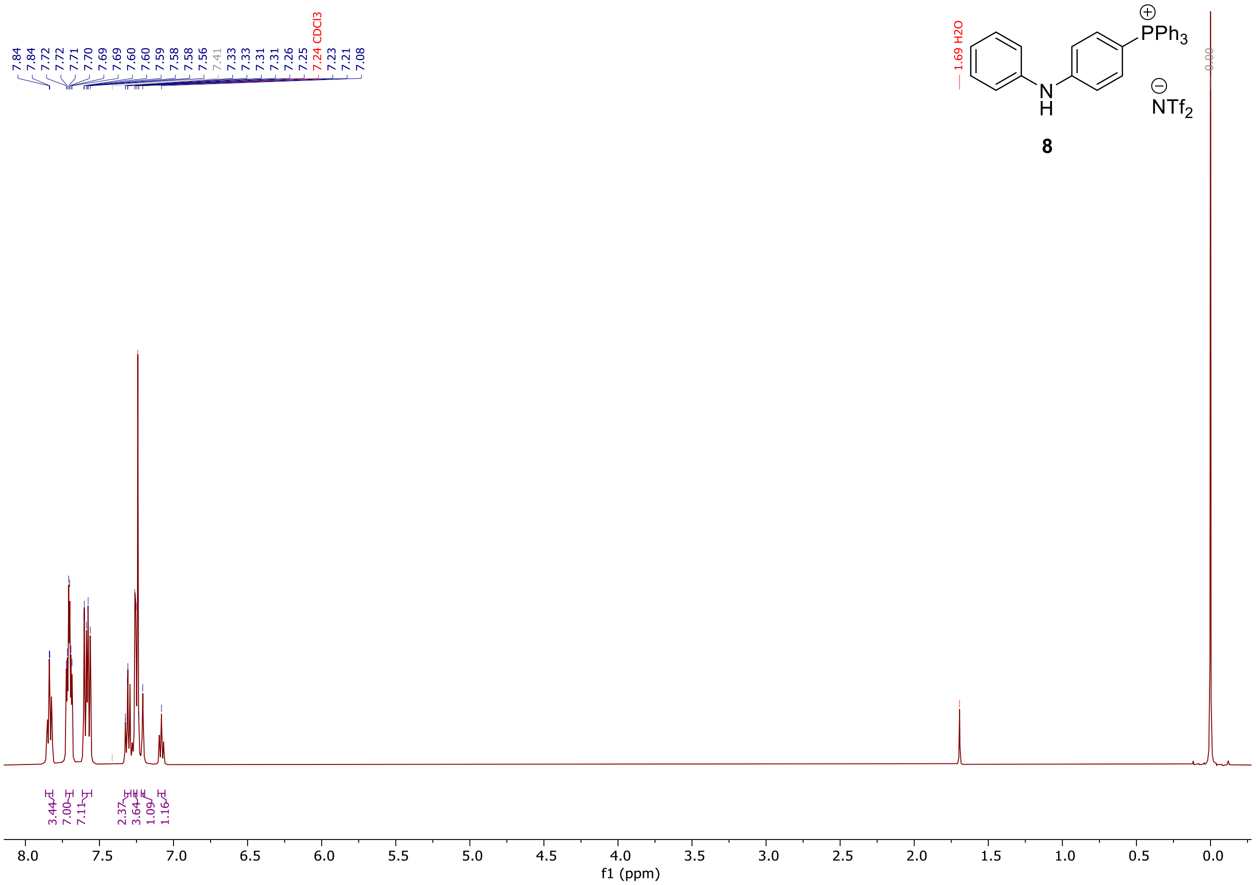
7.84 7.84 7.72 7.72 7.71 7.70 7.69 7.69 7.60 7.60 7.59 7.58 7.58 7.56 7.41 7.33 7.33 7.31 7.31 7.26 7.25 7.24 CDCl3 7.23 7.21 7.08
1.69 H2O
0.00
PPh3
N
H
NTf2
8
3.44 7.00 7.11 2.37 3.64 1.09 1.16
8.0 7.5 7.0 6.5 6.0 5.5 5.0 4.5 4.0 3.5 3.0 2.5 2.0 1.5 1.0 0.5 0.0
f1 (ppm)

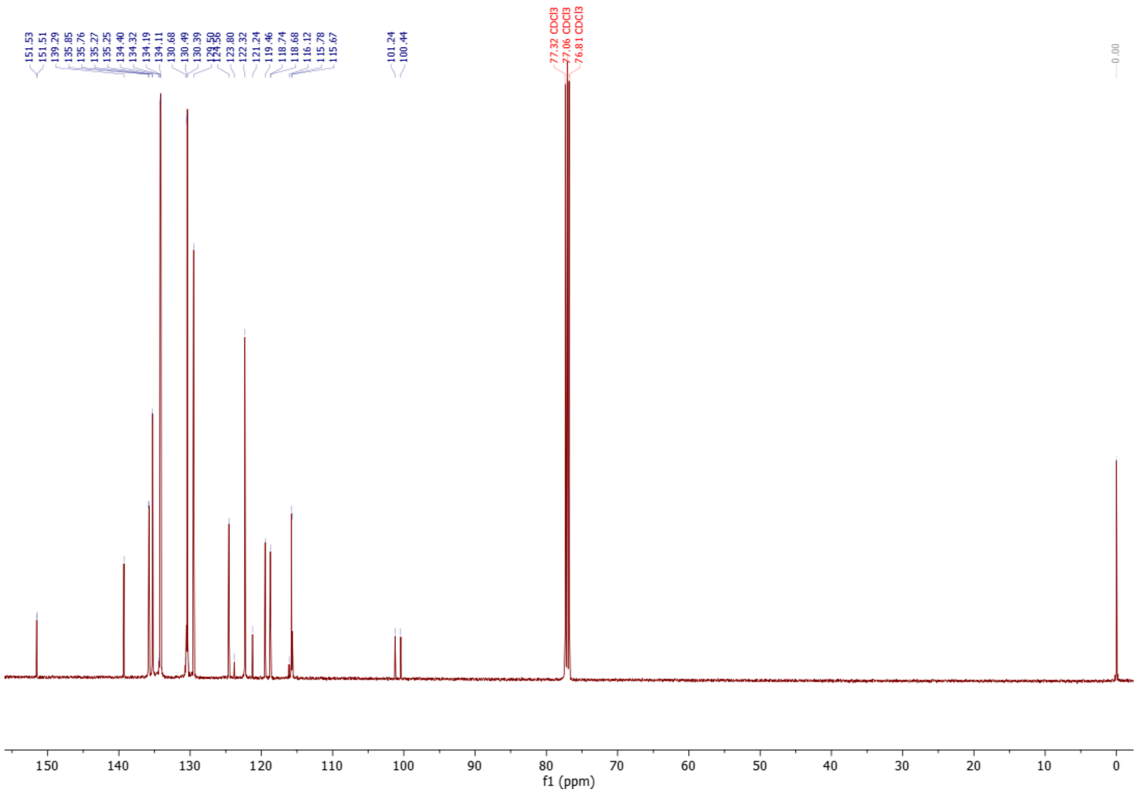
151.53 151.51 139.29 135.85 135.76 135.27 135.25 134.40 134.32 134.19 134.11 130.68 130.49 130.39 129.58 129.50 123.80 122.32 121.24 119.46 118.74 118.68 116.12 115.78 115.67
101.24 100.44
77.32 CDCl3 77.06 CDCl3 76.81 CDCl3
0.00
150 140 130 120 110 100 90 80 70 60 50 40 30 20 10 0
f1 (ppm)

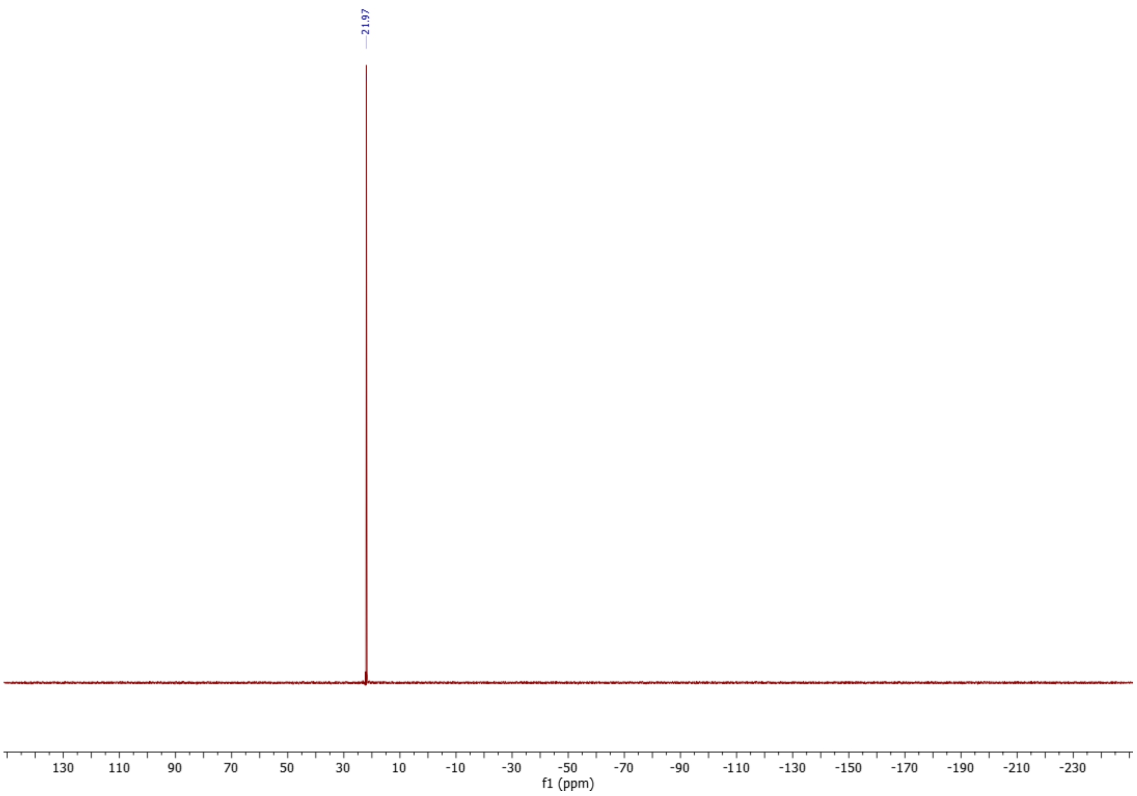
21.97
130 110 90 70 50 30 10 -10 -30 -50 -70 -90 -110 -130 -150 -170 -190 -210 -230
f1 (ppm)

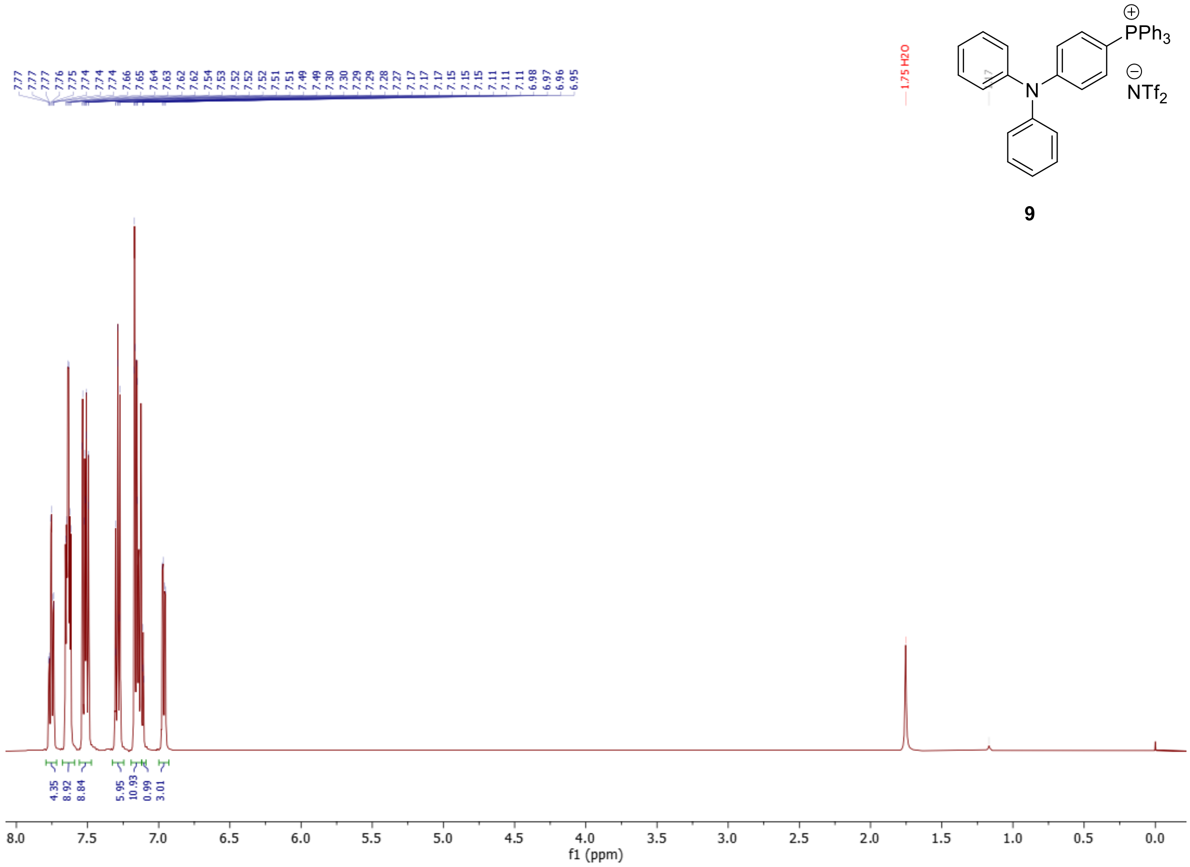

7.77
7.77
7.77
7.76
7.75
7.74
7.74
7.74
7.66
7.65
7.64
7.63
7.62
7.62
7.54
7.53
7.52
7.52
7.52
7.51
7.51
7.49
7.49
7.30
7.29
7.29
7.28
7.27
7.17
7.17
7.17
7.15
7.15
7.15
7.11
7.11
7.11
6.98
6.97
6.96
6.95
1.75 H2O
4.35
8.92
8.84
5.95
10.93
0.99
3.01
8.0
7.5
7.0
6.5
6.0
5.5
5.0
4.5
4.0
3.5
3.0
2.5
2.0
1.5
1.0
0.5
0.0
f1 (ppm)
PPh3
NTf2
N

9

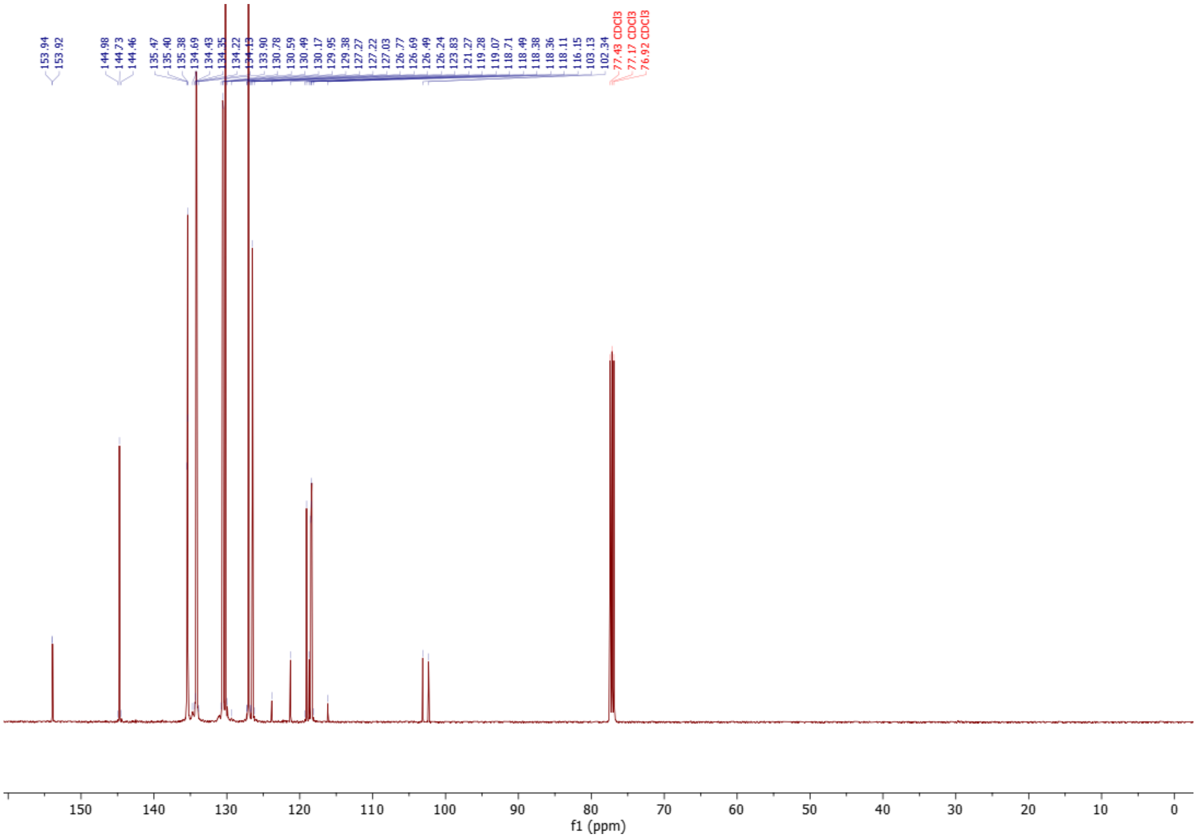

153.94
153.92
144.98
144.73
144.46
135.47
135.40
135.38
134.69
134.43
134.35
134.22
134.13
133.90
130.78
130.59
130.49
130.17
129.95
129.38
127.27
127.22
127.03
126.77
126.69
126.49
126.24
123.83
121.27
119.28
119.07
118.71
118.49
118.38
118.36
118.11
116.15
103.13
102.34
77.43 CDCl3
77.17 CDCl3
76.92 CDCl3
150
140
130
120
110
100
90
80
70
60
50
40
30
20
10
0
f1 (ppm)

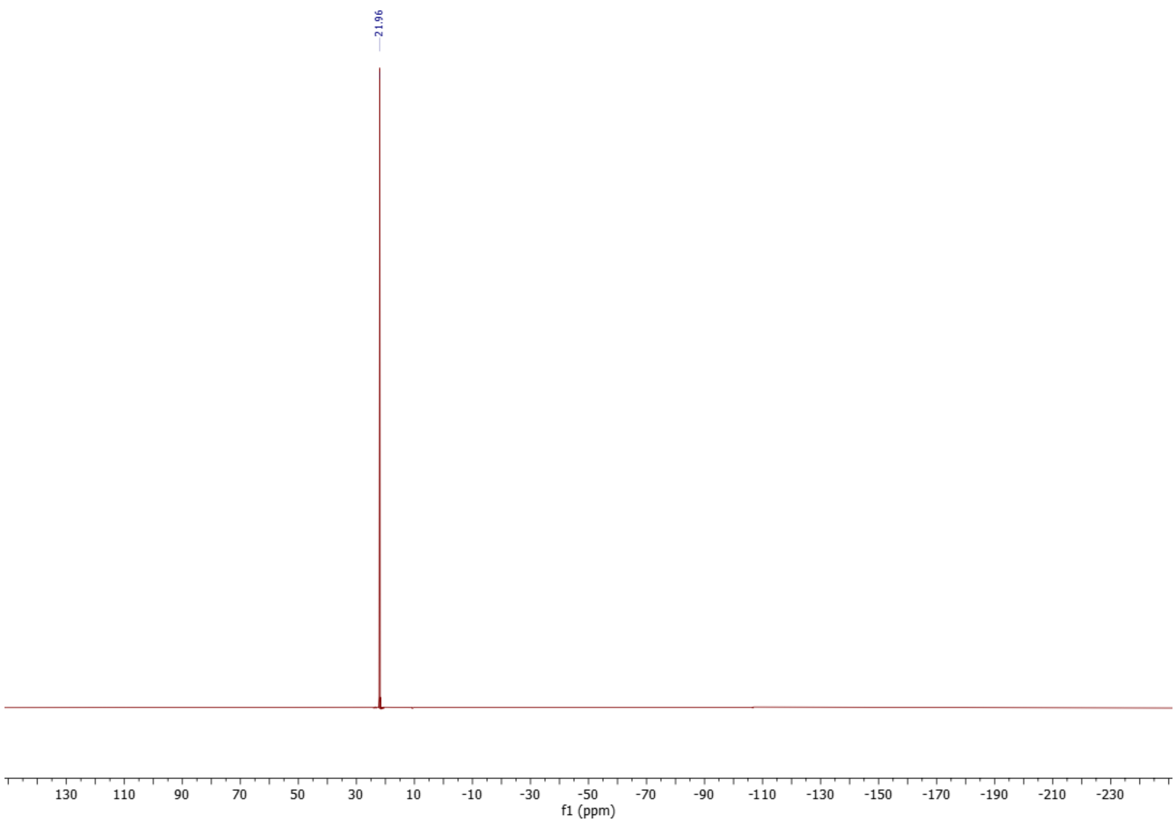
21.96
130
110
90
70
50
30
10
-10
-30
-50
-70
-90
-110
-130
-150
-170
-190
-210
-230
f1 (ppm)

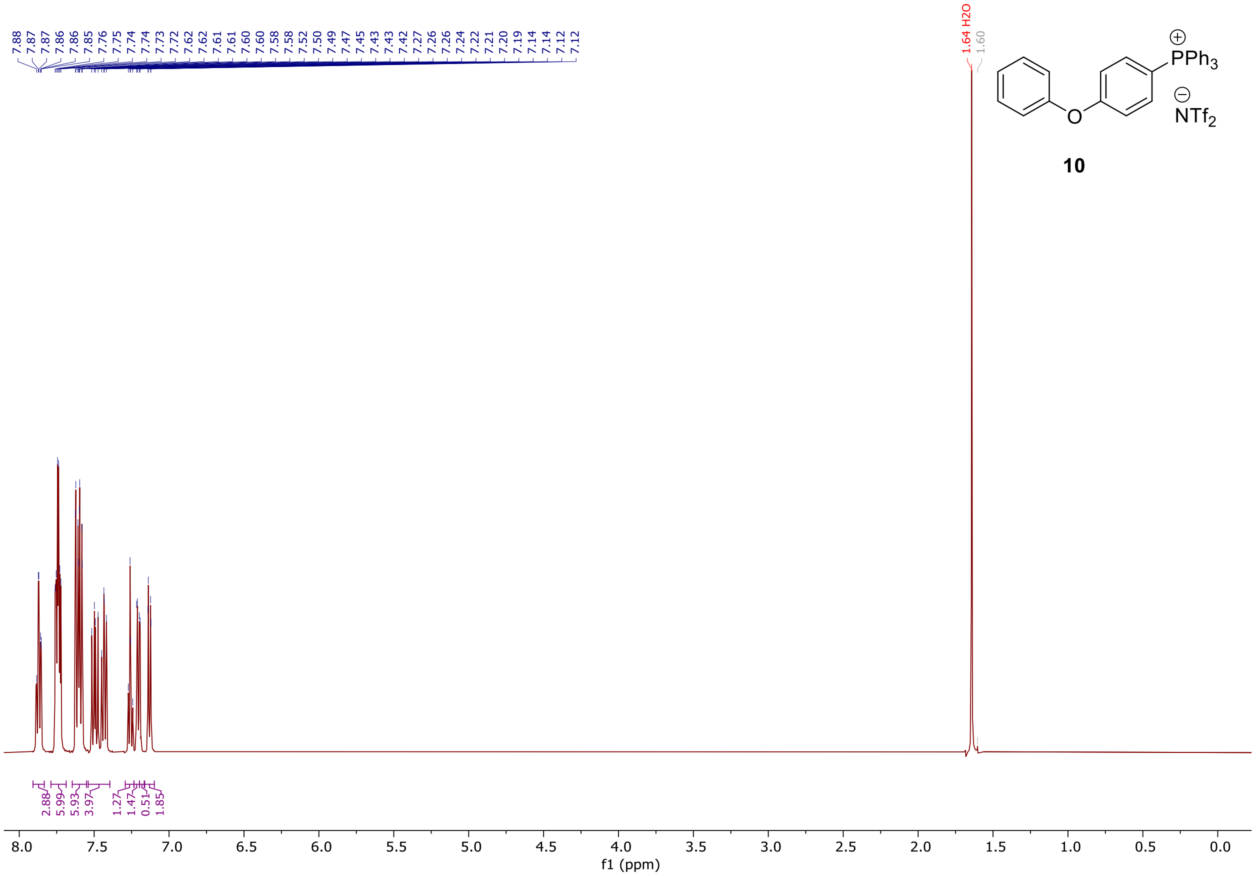
7.88 7.87 7.87 7.86 7.86 7.85 7.76 7.75 7.74 7.74 7.73 7.72 7.62 7.62 7.61 7.61 7.60 7.60 7.58 7.58 7.52 7.50 7.49 7.47 7.45 7.43 7.43 7.42 7.27 7.26 7.26 7.24 7.22 7.21 7.20 7.19 7.14 7.12 7.12
1.64 H2O
1.60
PPh3
NTf2
O
2.88 5.99 5.93 3.97 1.27 1.47 0.51 1.85
f1 (ppm)

10

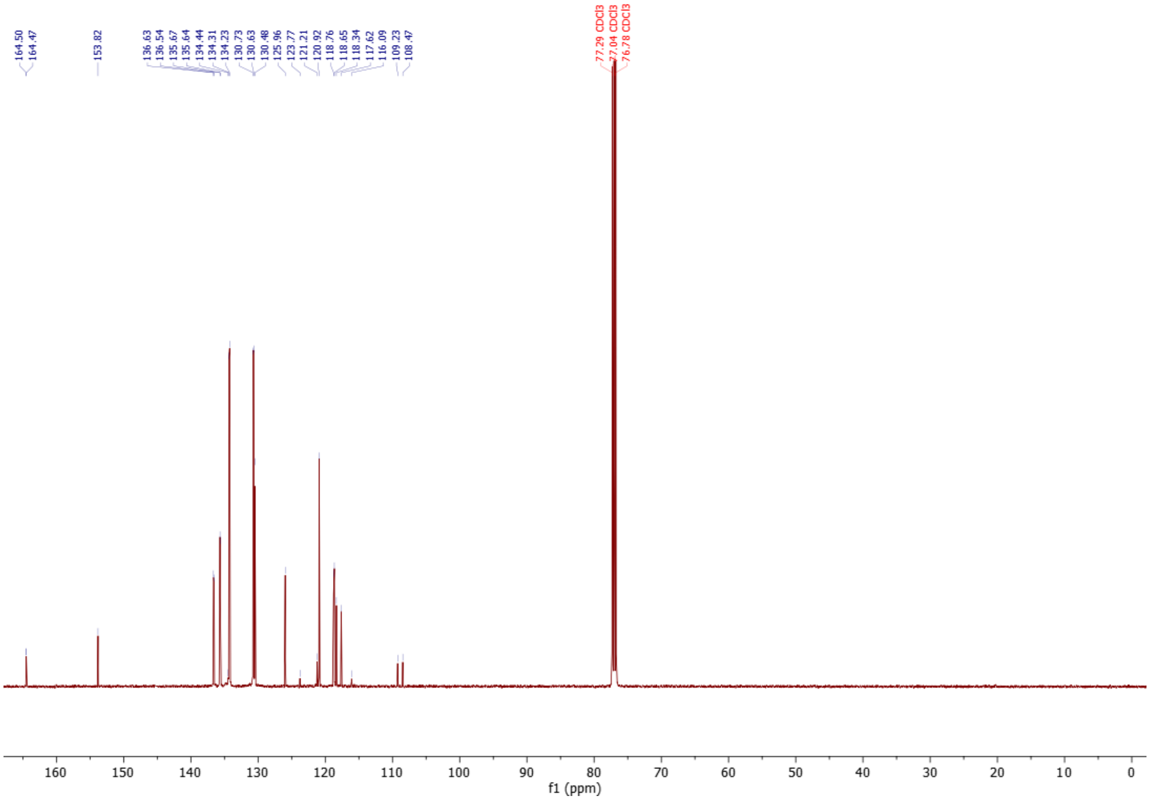
164.50 164.47
153.82
136.63 136.54 135.67 135.64 134.44 134.31 134.23 130.73 130.63 130.48 125.96 123.77 121.21 120.92 118.76 118.65 118.34 117.62 116.09 109.23 108.47
77.29 CDCl3 77.04 CDCl3 76.78 CDCl3
f1 (ppm)

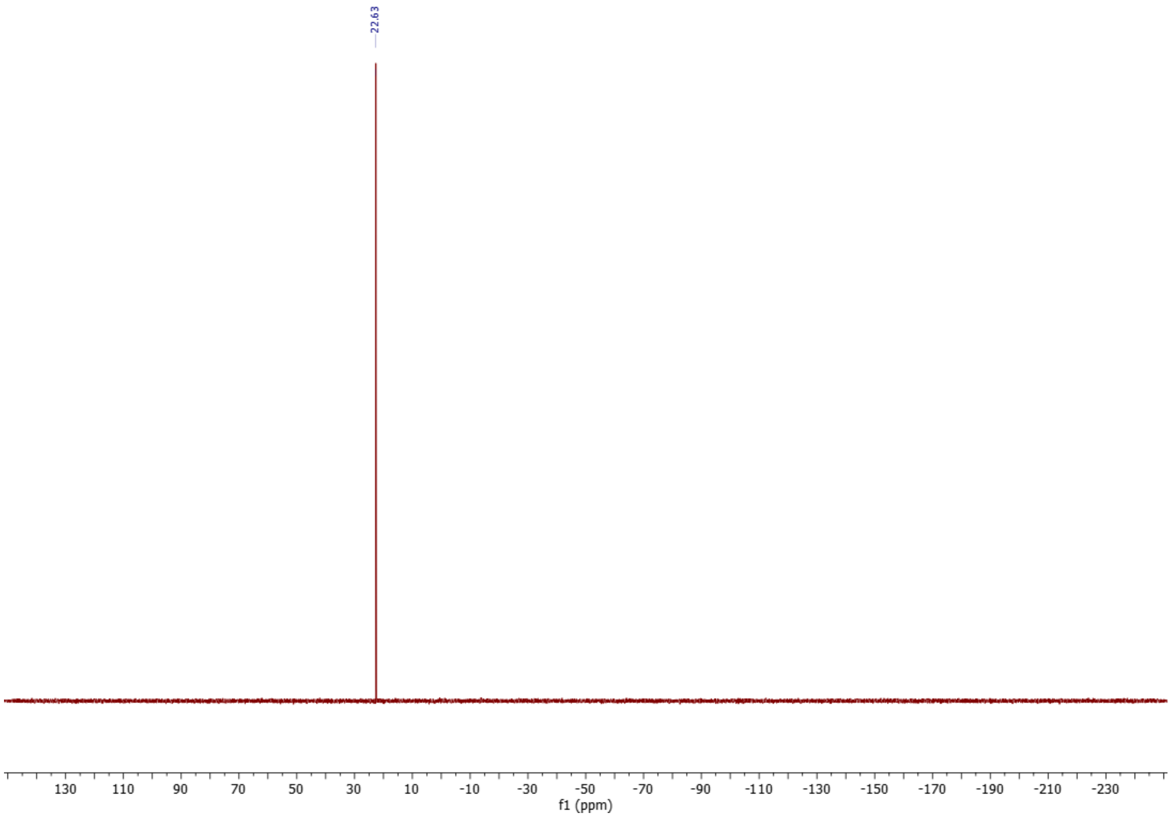
22.63
130
110
90
70
50
30
10
-10
-30
-50
-70
-90
-110
-130
-150
-170
-190
-210
-230
f1 (ppm)

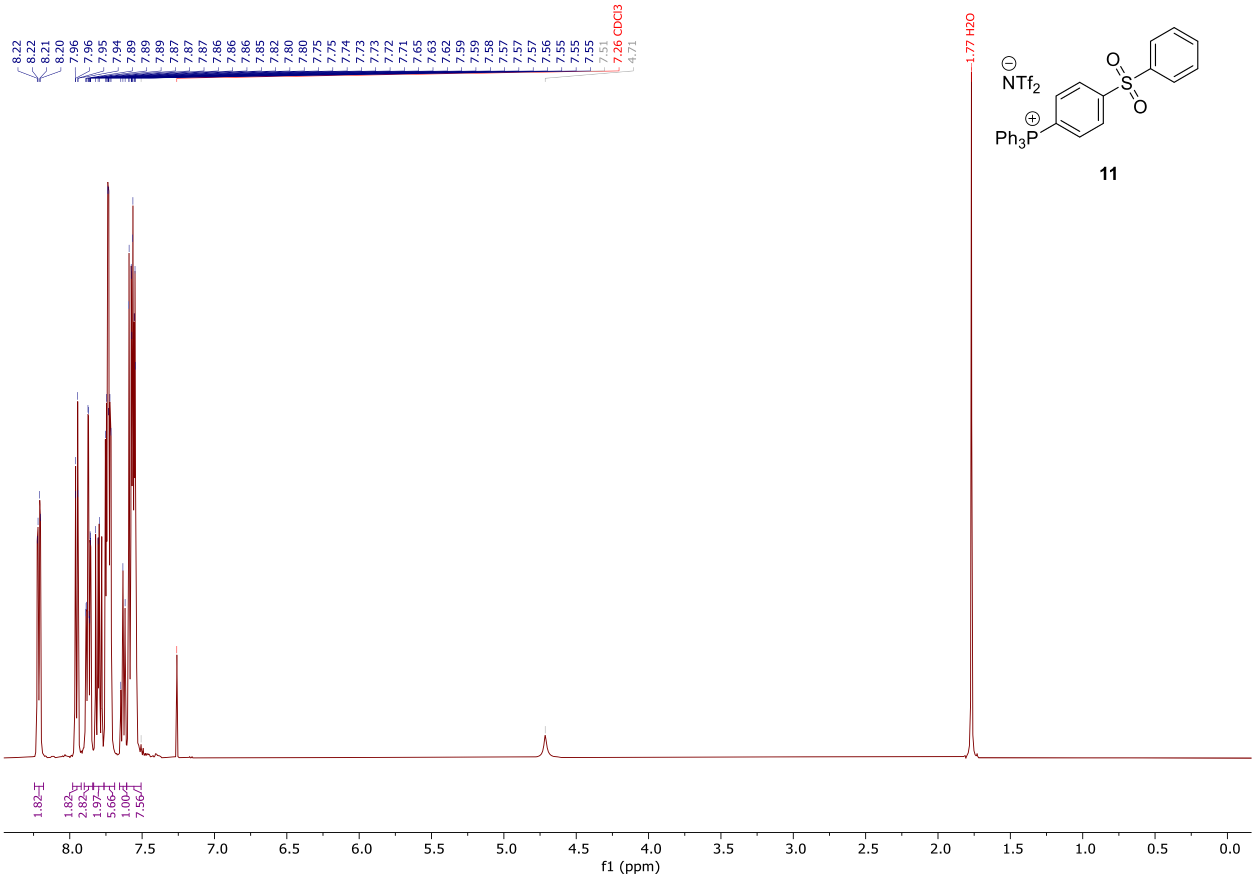

7.26 CDCl3
1.77 H2O
NTf2
Ph3P
11
f1 (ppm)

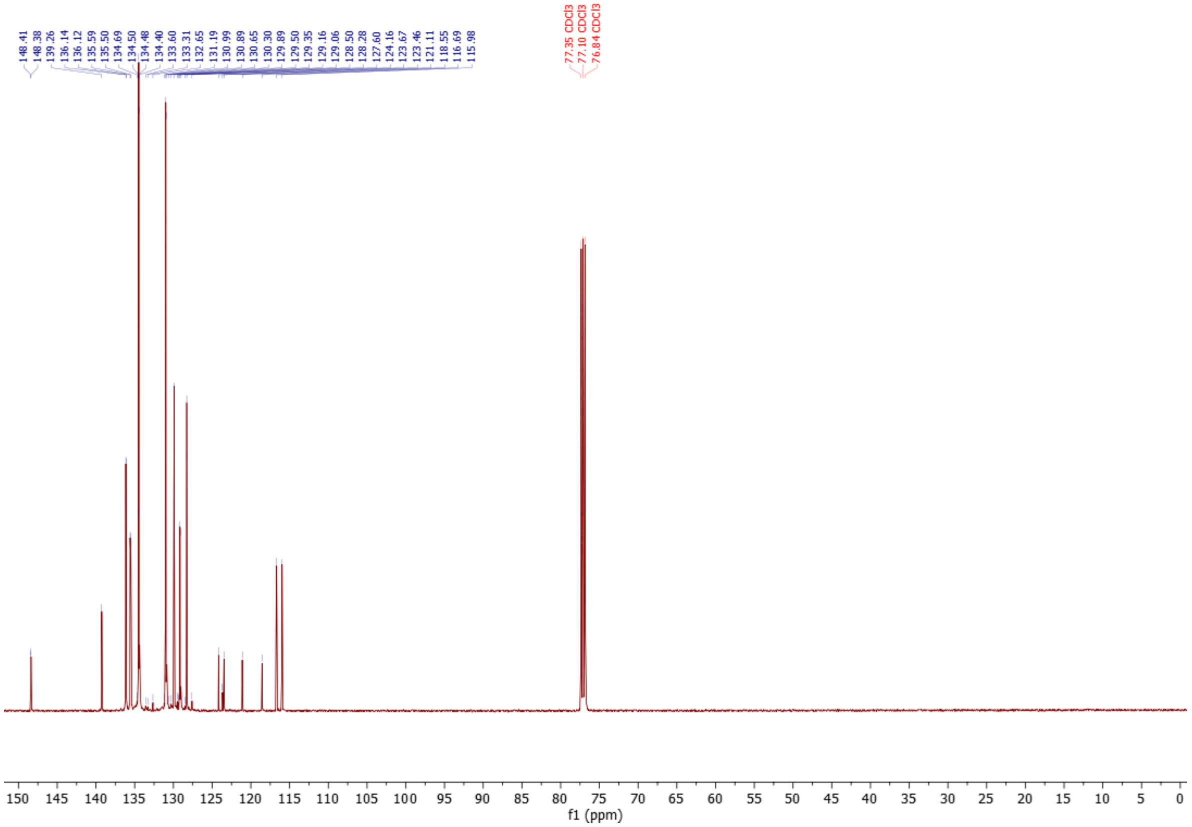

77.35 CDCl3
77.10 CDCl3
76.84 CDCl3
f1 (ppm)

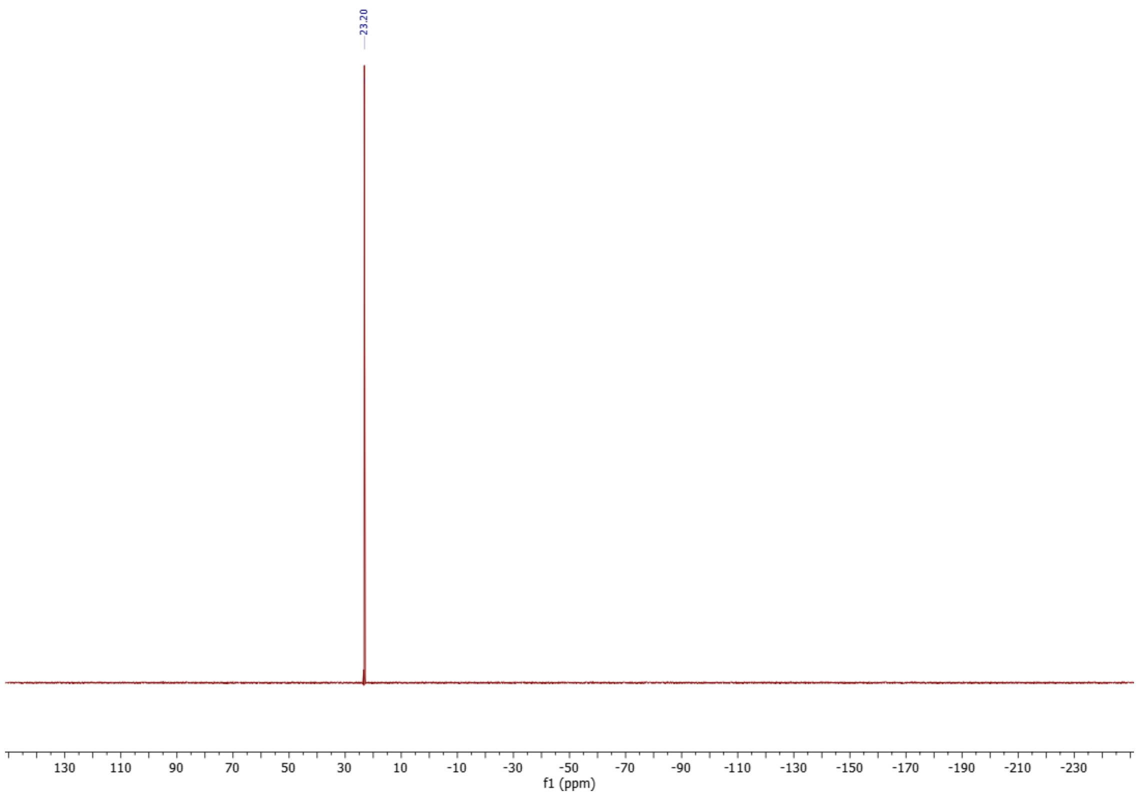
23.20
130
110
90
70
50
30
10
-10
-30
-50
-70
-90
-110
-130
-150
-170
-190
-210
-230
f1 (ppm)

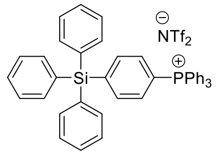
$\ominus$
NTf_2
$\oplus$
PPh_3
Si

12

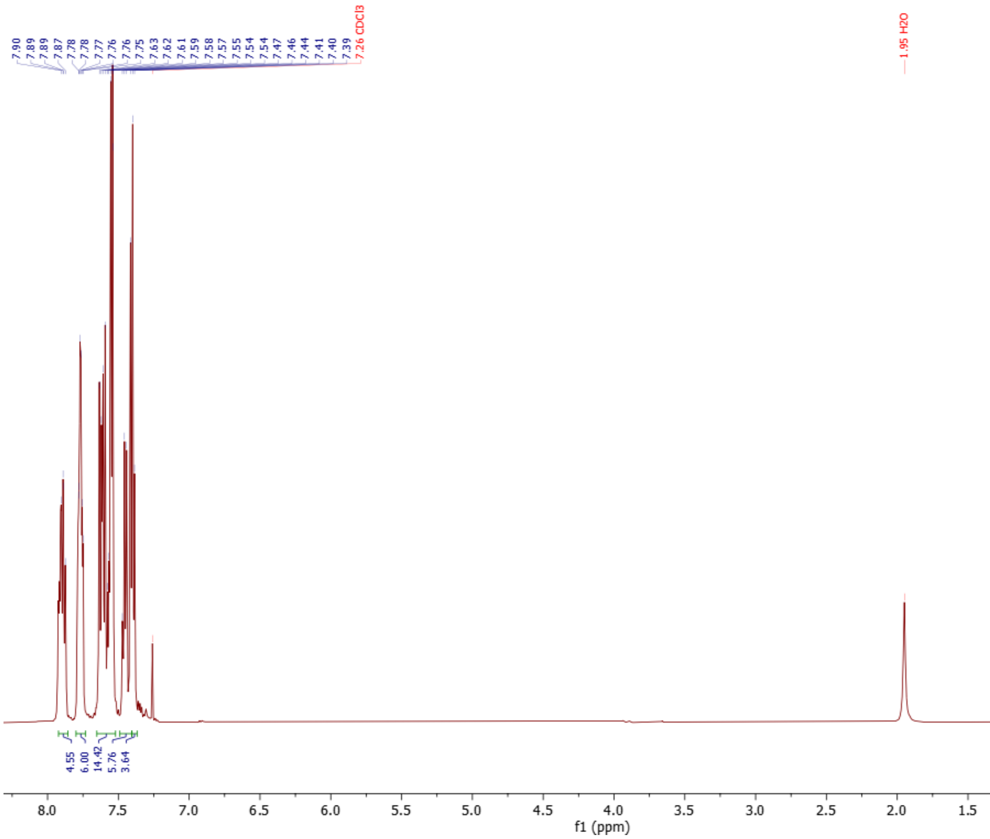
7.90
7.89
7.89
7.87
7.78
7.78
7.77
7.76
7.76
7.75
7.63
7.62
7.61
7.59
7.58
7.57
7.55
7.54
7.54
7.54
7.47
7.46
7.44
7.41
7.40
7.39
7.26 CDCl3
1.95 H2O
4.55
6.00
14.42
5.76
3.64
8.0
7.5
7.0
6.5
6.0
5.5
5.0
4.5
4.0
3.5
3.0
2.5
2.0
1.5
1.0
0.5
0.0
f1 (ppm)

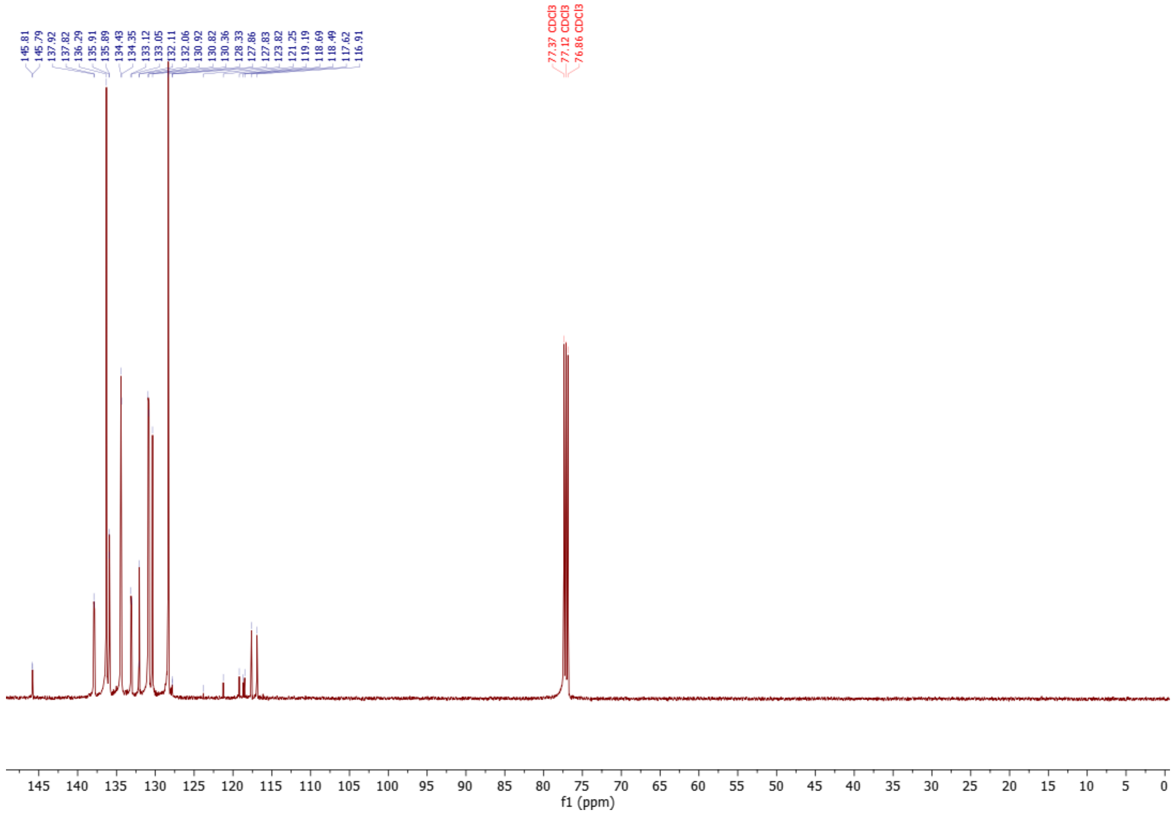
145.81
145.79
137.92
137.82
136.29
135.91
135.89
134.43
134.35
133.12
133.05
132.11
132.06
130.92
130.82
130.36
128.33
127.86
127.83
123.82
121.25
119.19
118.69
118.49
117.62
116.91
77.37 CDCl3
77.12 CDCl3
76.86 CDCl3
145
140
135
130
125
120
115
110
105
100
95
90
85
80
75
70
65
60
55
50
45
40
35
30
25
20
15
10
5
0
f1 (ppm)

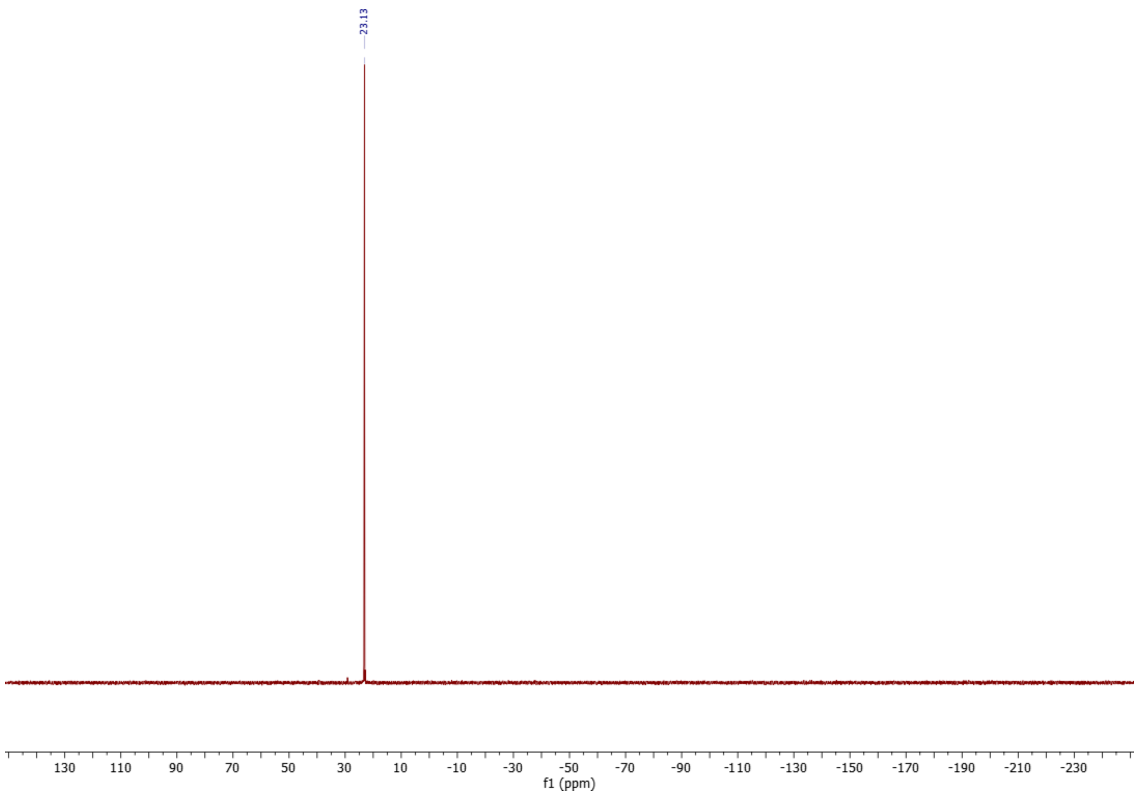

23.13
130 110 90 70 50 30 10 -10 -30 -50 -70 -90 -110 -130 -150 -170 -190 -210 -230
f1 (ppm)

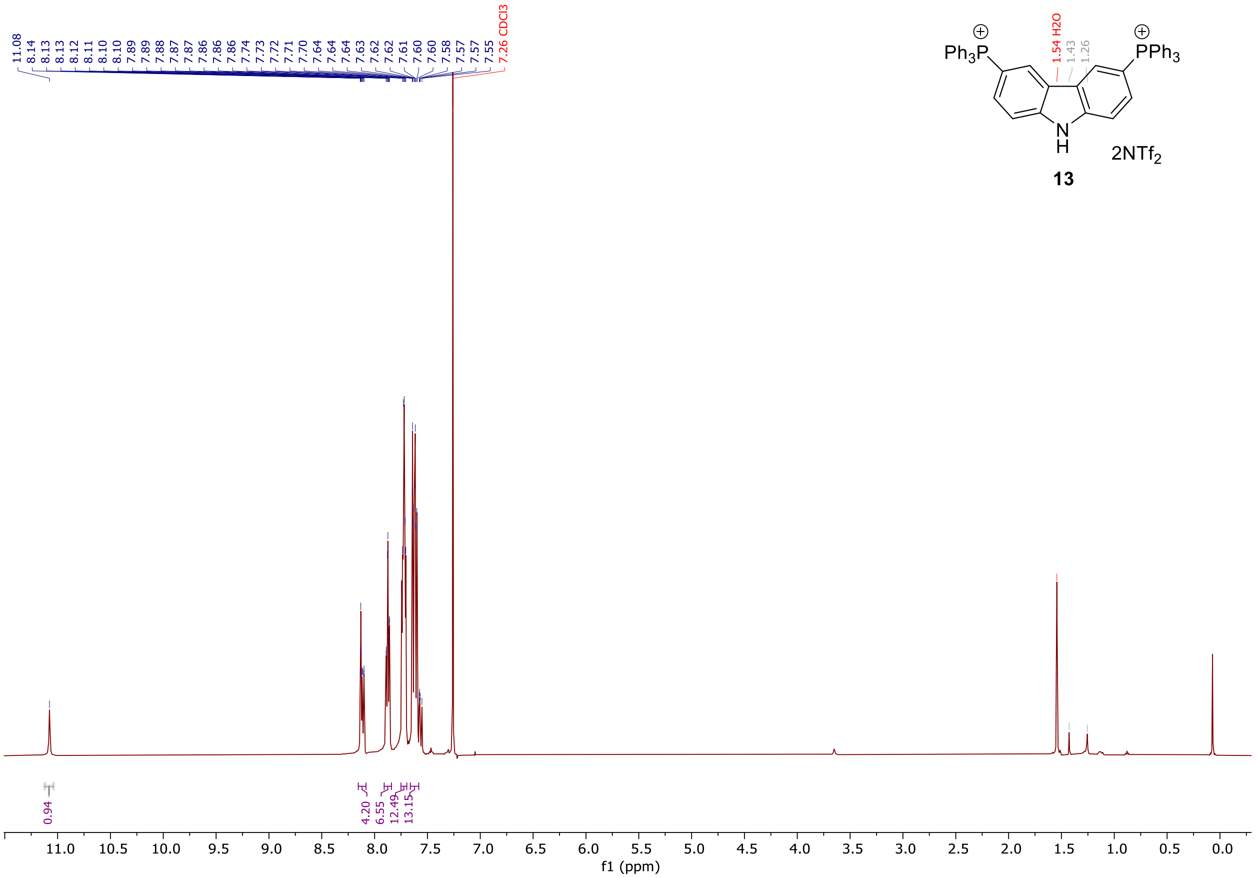
11.08
8.14
8.13
8.13
8.12
8.11
8.10
8.10
7.89
7.89
7.88
7.87
7.87
7.86
7.86
7.86
7.74
7.73
7.72
7.71
7.70
7.64
7.64
7.64
7.63
7.62
7.62
7.61
7.60
7.60
7.58
7.57
7.57
7.55
7.26 CDCl3
1.54 H2O
1.43
1.26
Ph3P
PPh3
N
H
2NTf2
13
0.94
4.20
6.55
12.49
13.15
f1 (ppm)

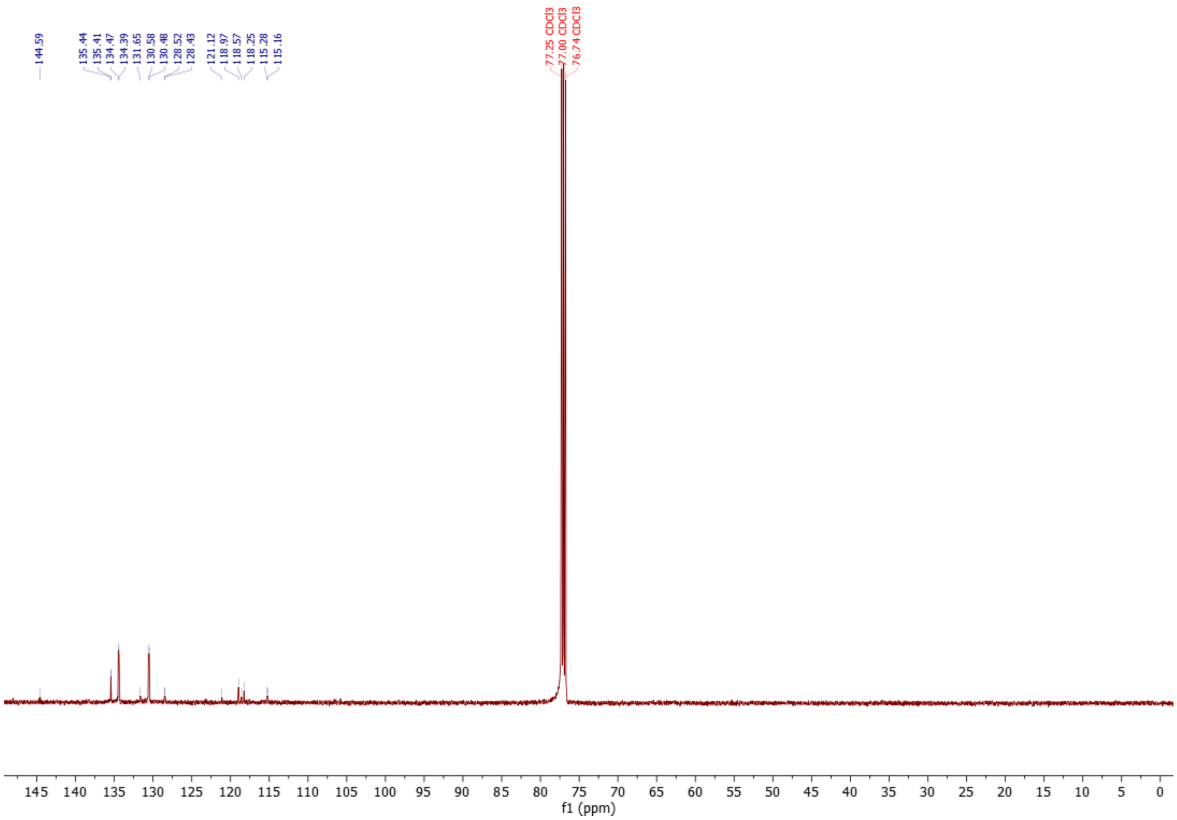
144.59
135.44
135.41
134.47
134.39
131.65
130.58
130.48
128.52
128.43
121.12
118.97
118.57
118.25
115.28
115.16
77.25 CDCl3
77.00 CDCl3
76.74 CDCl3
f1 (ppm)

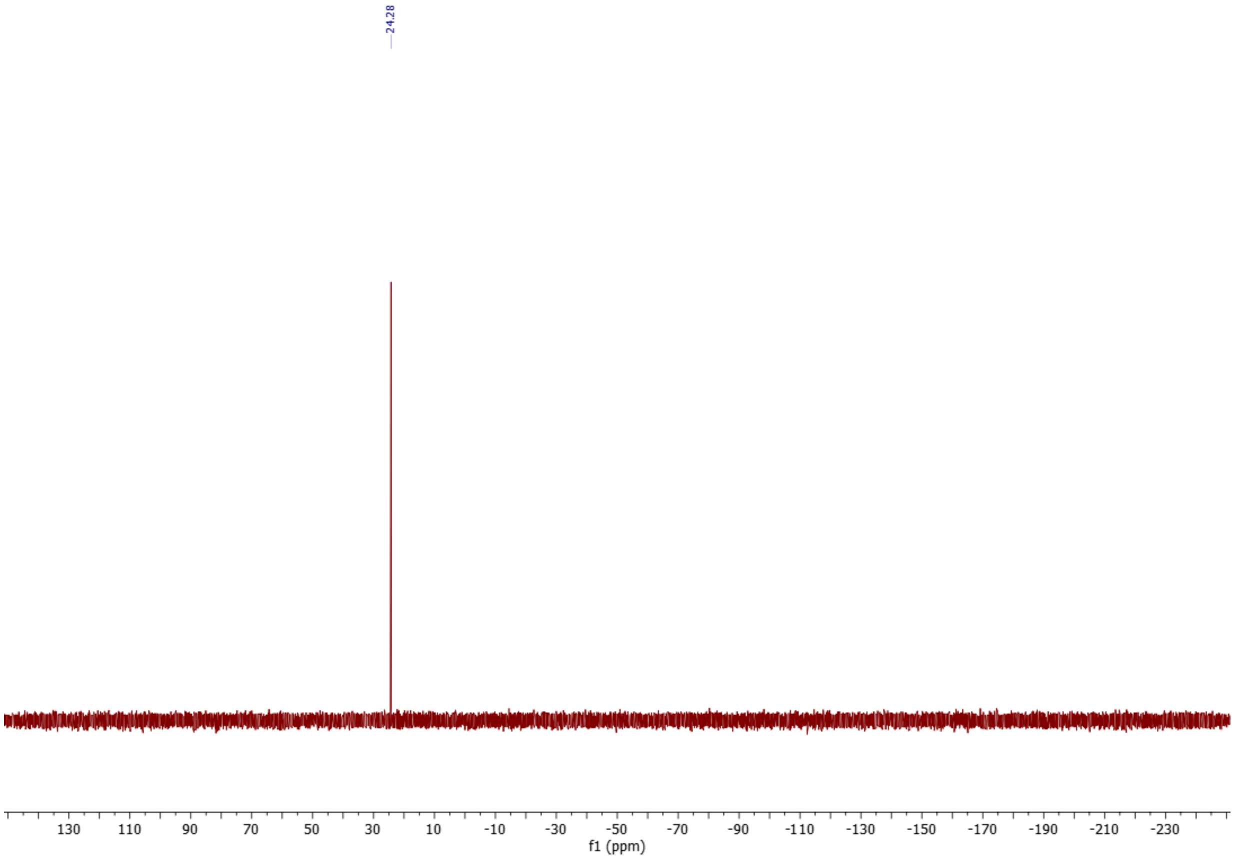

24.28
130
110
90
70
50
30
10
-10
-30
-50
-70
-90
-110
-130
-150
-170
-190
-210
-230
f1 (ppm)

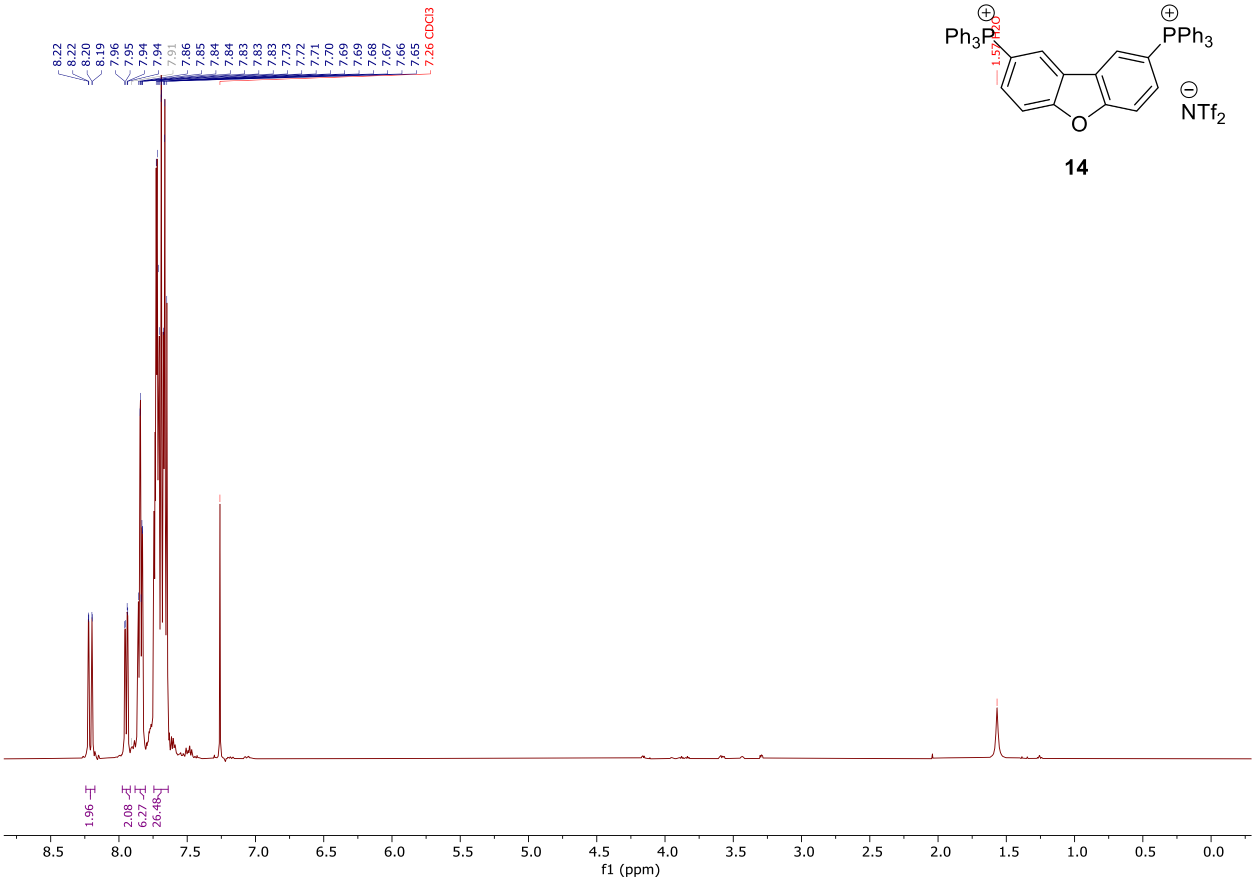

14

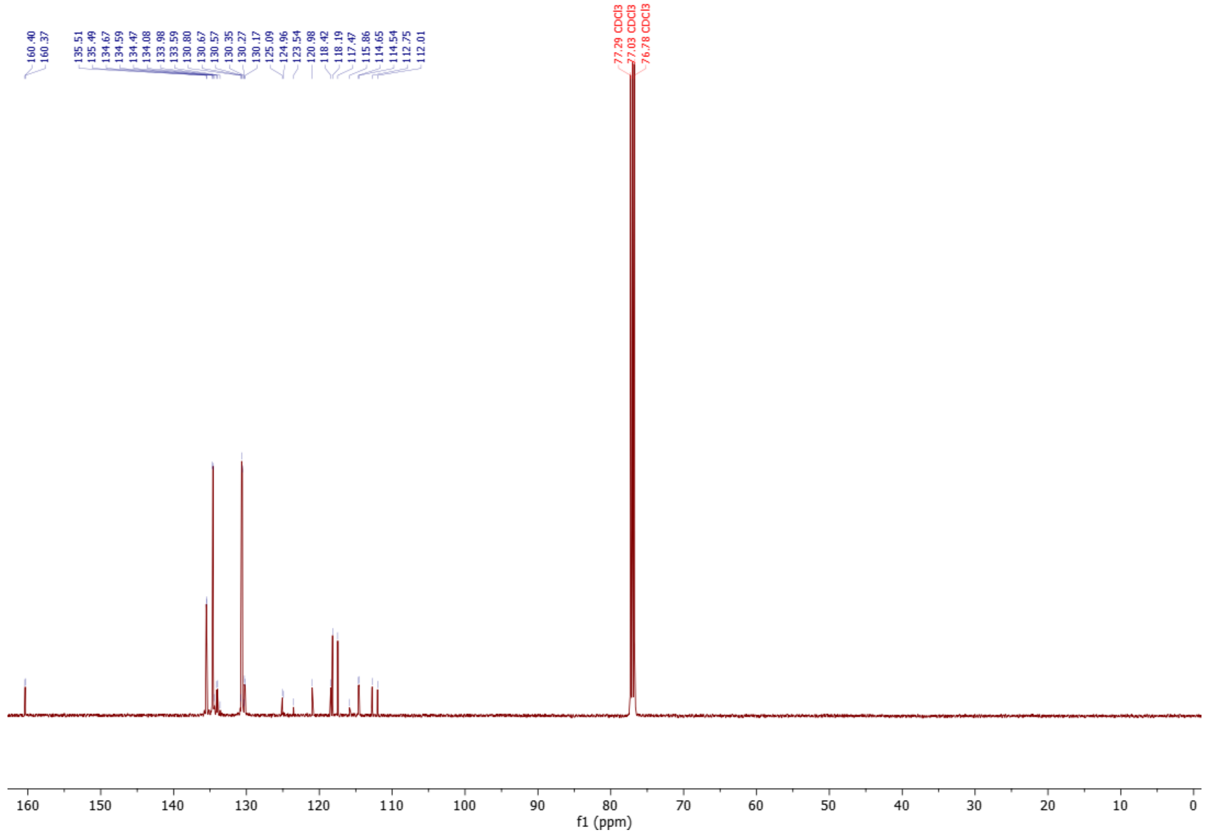

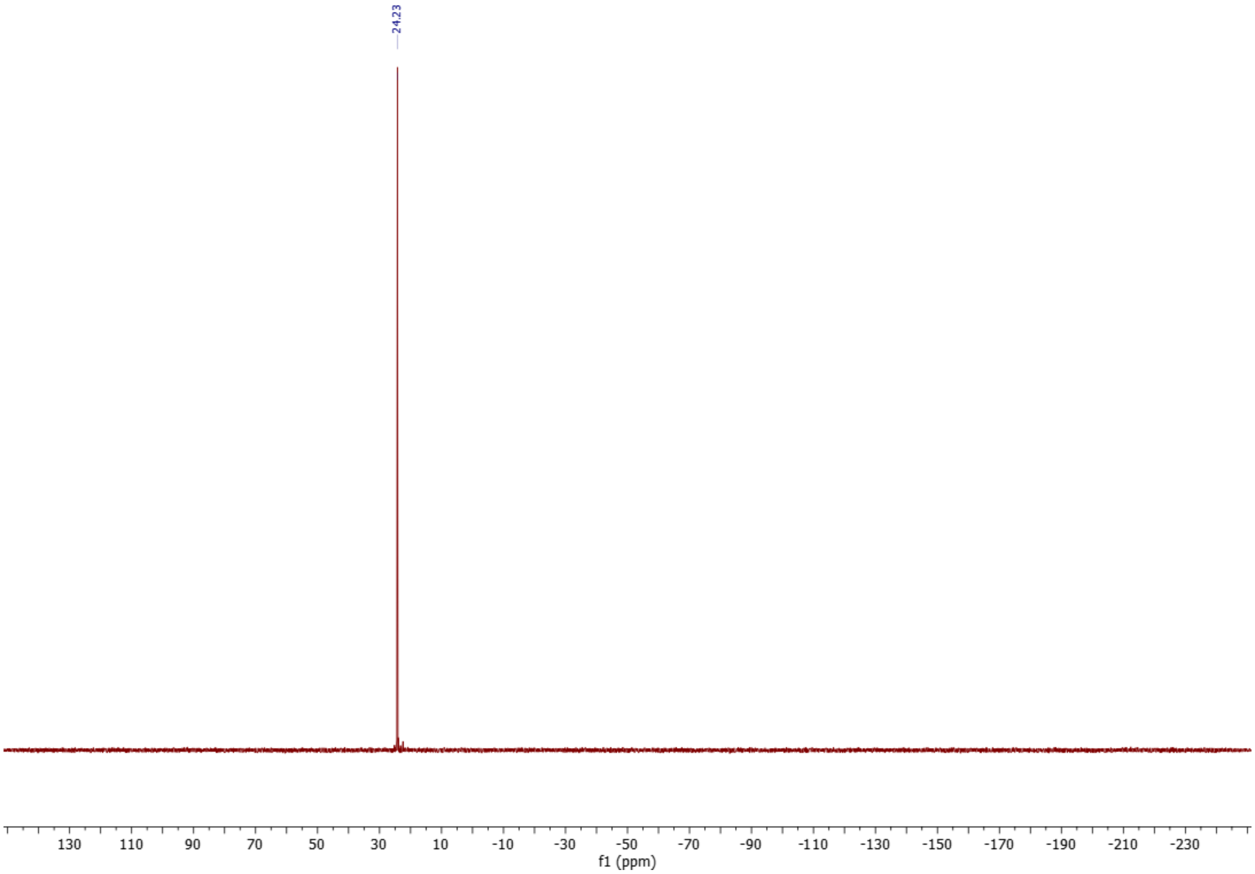
24.23
130
110
90
70
50
30
10
-10
-30
-50
-70
-90
-110
-130
-150
-170
-190
-210
-230
f1 (ppm)

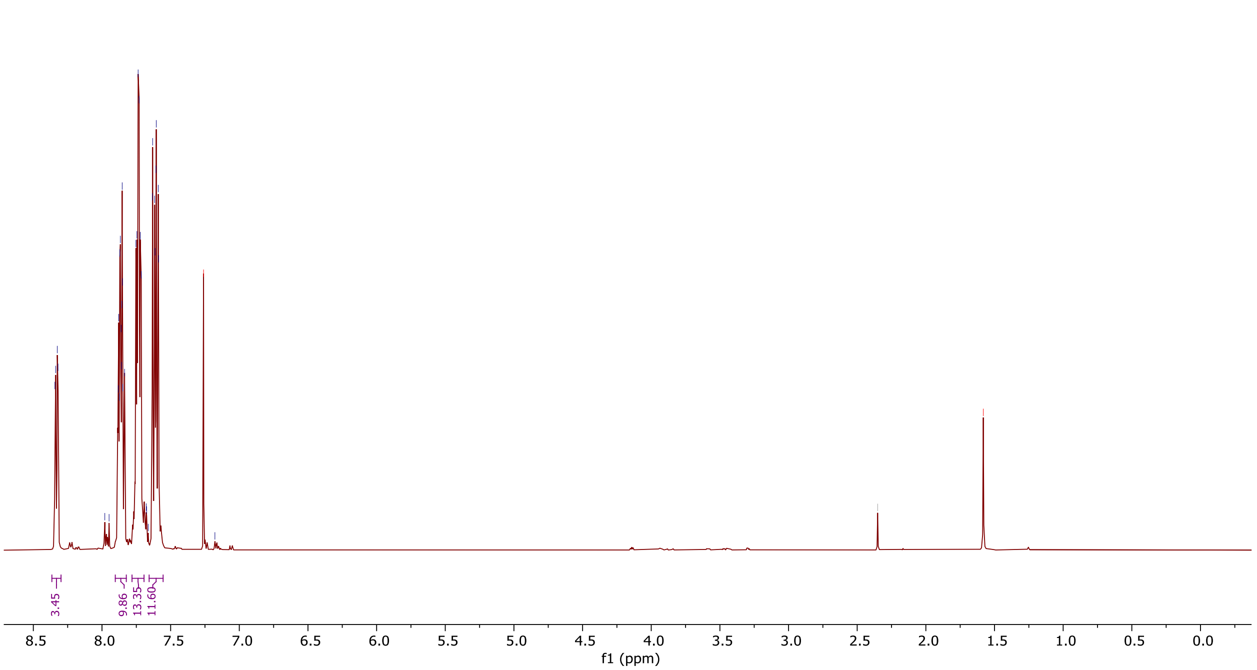
8.34
8.34
8.33
8.32
7.98
7.95
7.88
7.88
7.87
7.87
7.87
7.86
7.86
7.85
7.85
7.85
7.84
7.75
7.75
7.74
7.73
7.72
7.71
7.68
7.67
7.66
7.63
7.63
7.62
7.61
7.61
7.60
7.59
7.59
7.26 CDCl3
7.18
2.35
1.58 H2O
3.45
9.86
13.35
11.60
f1 (ppm)

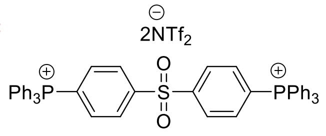
2NTf2
Ph3P
PPh3
S
O
O

15

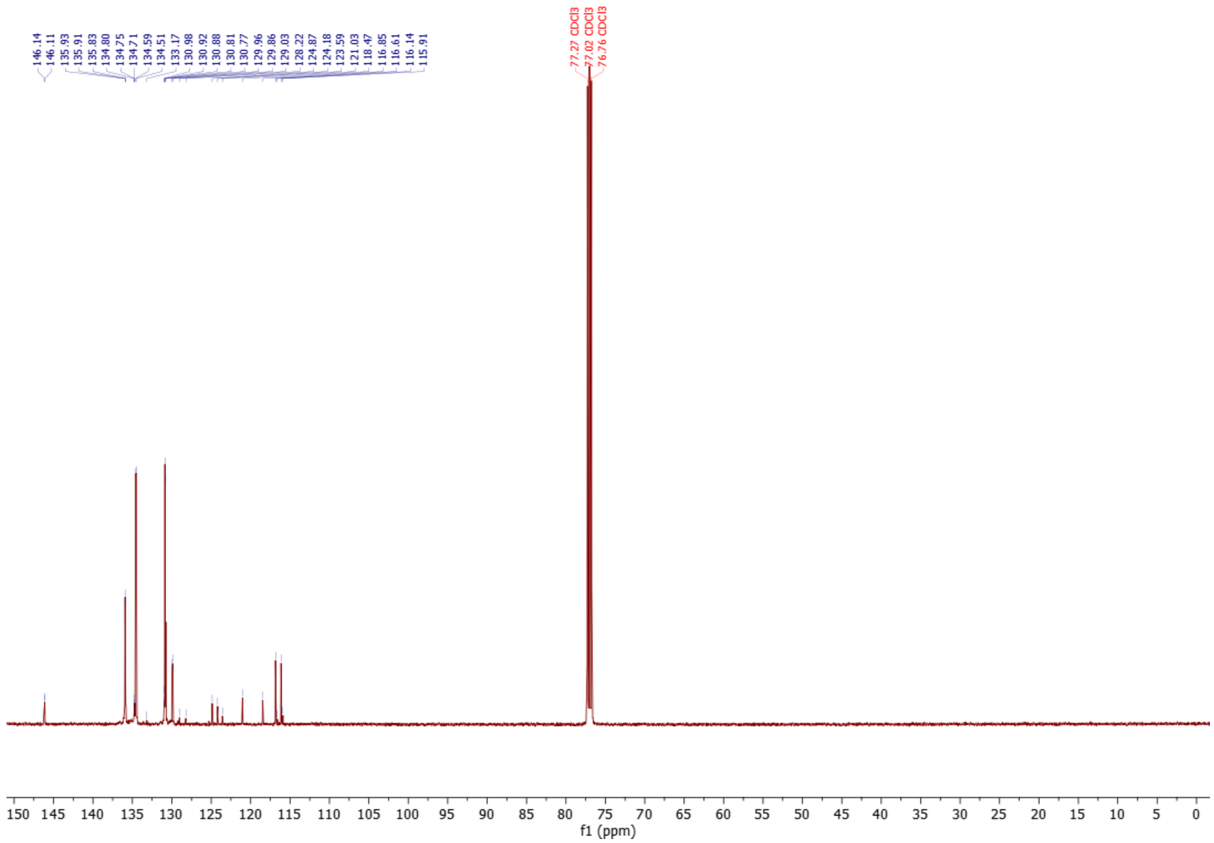
146.14
146.11
135.93
135.91
135.83
134.80
134.75
134.71
134.59
134.51
133.17
130.98
130.92
130.88
130.81
130.77
129.96
129.86
129.03
128.22
124.87
124.18
123.59
121.03
118.47
116.85
116.61
116.14
115.91
77.27 CDCl3
77.02 CDCl3
76.76 CDCl3
f1 (ppm)

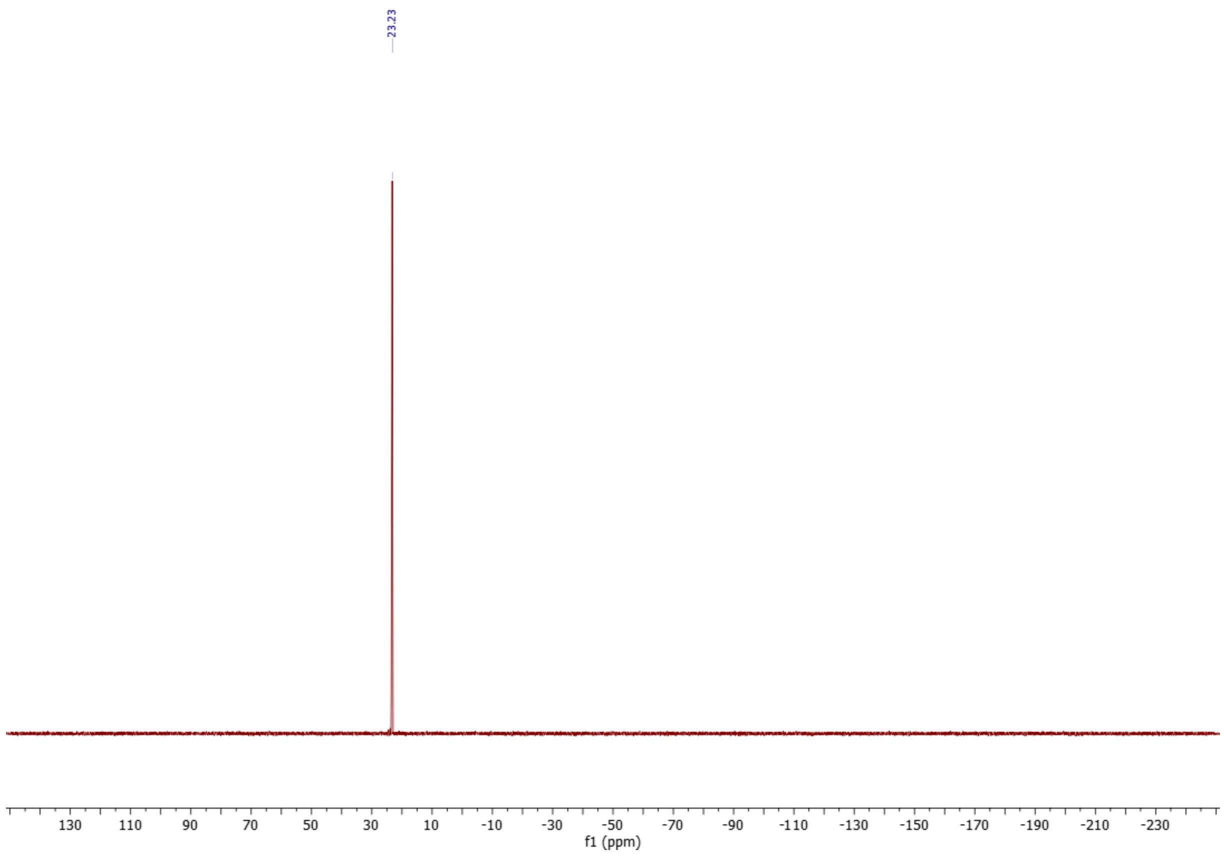

23.23
130 110 90 70 50 30 10 -10 -30 -50 -70 -90 -110 -130 -150 -170 -190 -210 -230
f1 (ppm)

DSC and TGA Thermograms of the Products

1

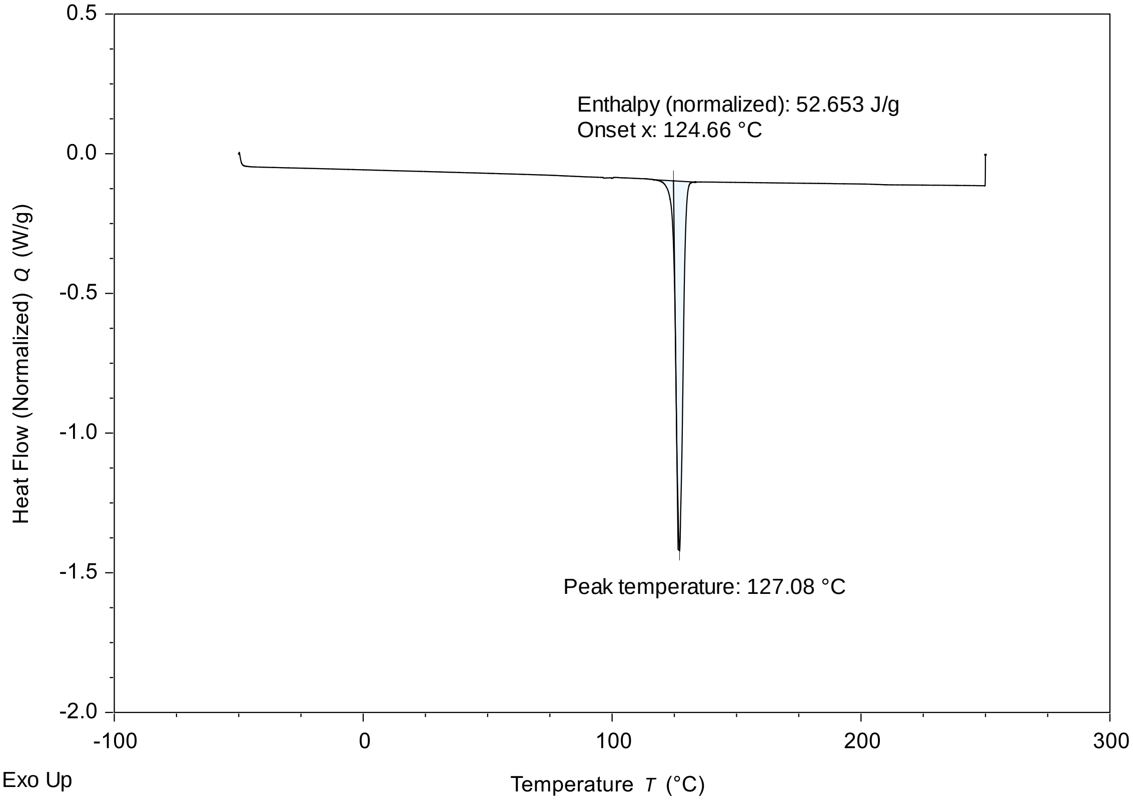

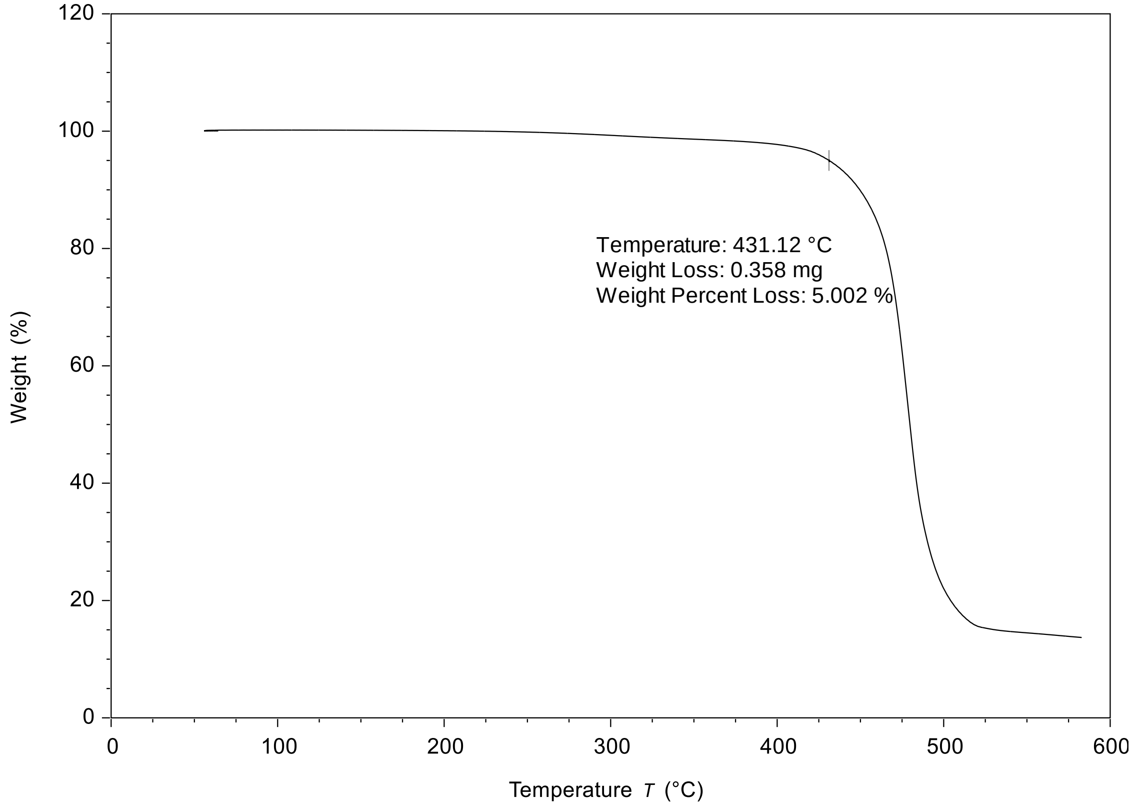

2

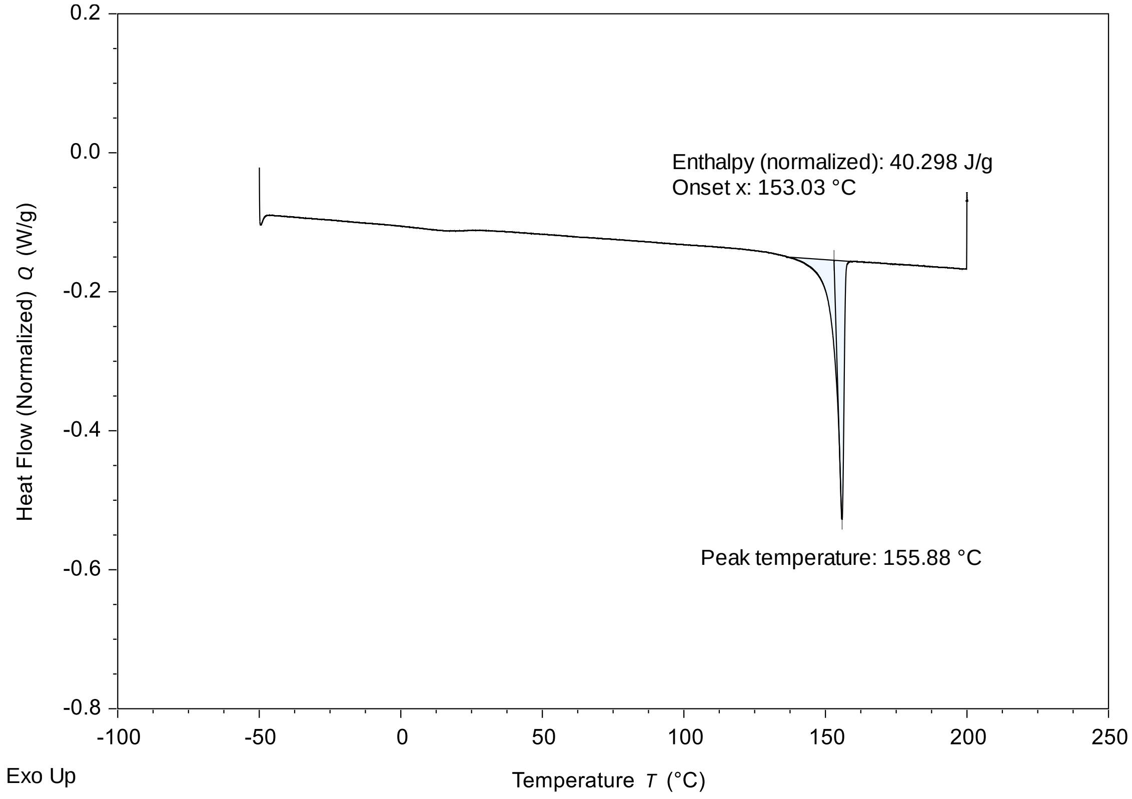
Enthalpy (normalized): 40.298 J/g
Onset x: 153.03 °C
Peak temperature: 155.88 °C
Heat Flow (Normalized) Q (W/g)
Temperature T (°C)
Exo Up
0.2
0.0
-0.2
-0.4
-0.6
-0.8
-100
-50
0
50
100
150
200
250

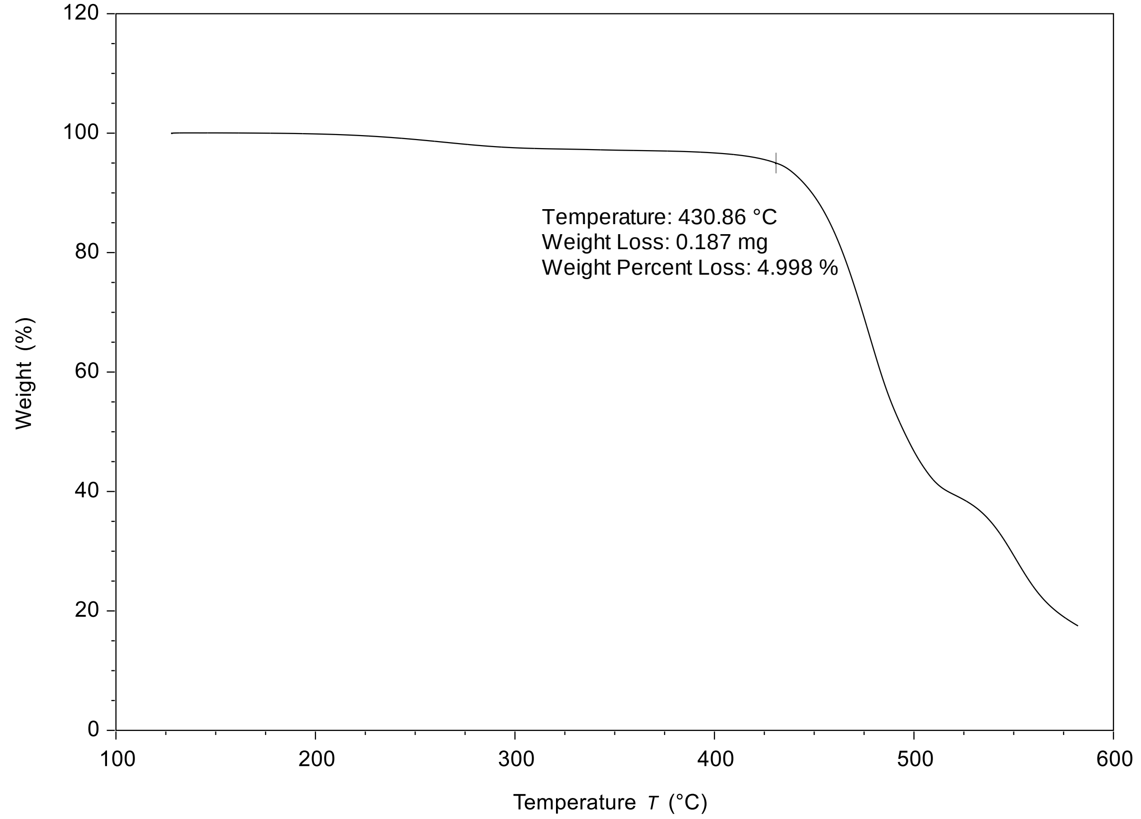
Temperature: 430.86 °C
Weight Loss: 0.187 mg
Weight Percent Loss: 4.998 %
Weight (%)
Temperature T (°C)
120
100
80
60
40
20
0
100
200
300
400
500
600

3

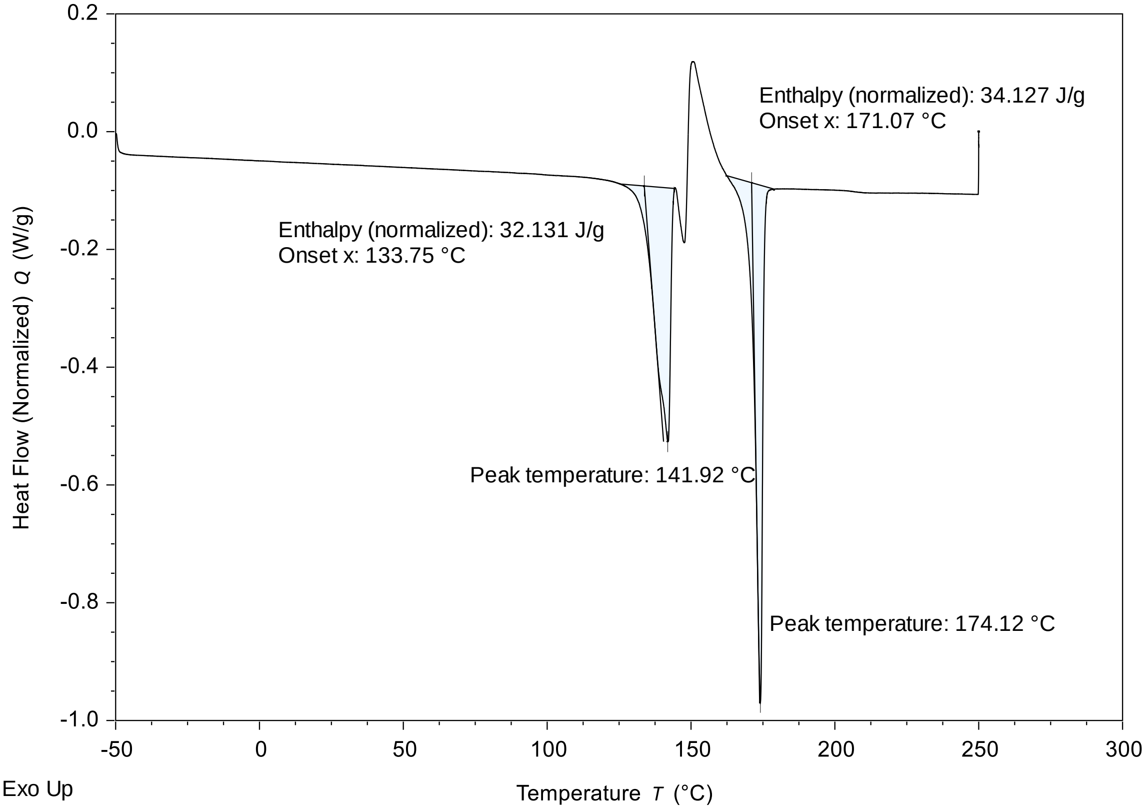

Enthalpy (normalized): 34.127 J/g
Onset x: 171.07 °C
Enthalpy (normalized): 32.131 J/g
Onset x: 133.75 °C
Peak temperature: 141.92 °C
Peak temperature: 174.12 °C
Heat Flow (Normalized) Q (W/g)
Temperature T (°C)
Exo Up
0.2
0.0
-0.2
-0.4
-0.6
-0.8
-1.0
-50
0
50
100
150
200
250
300

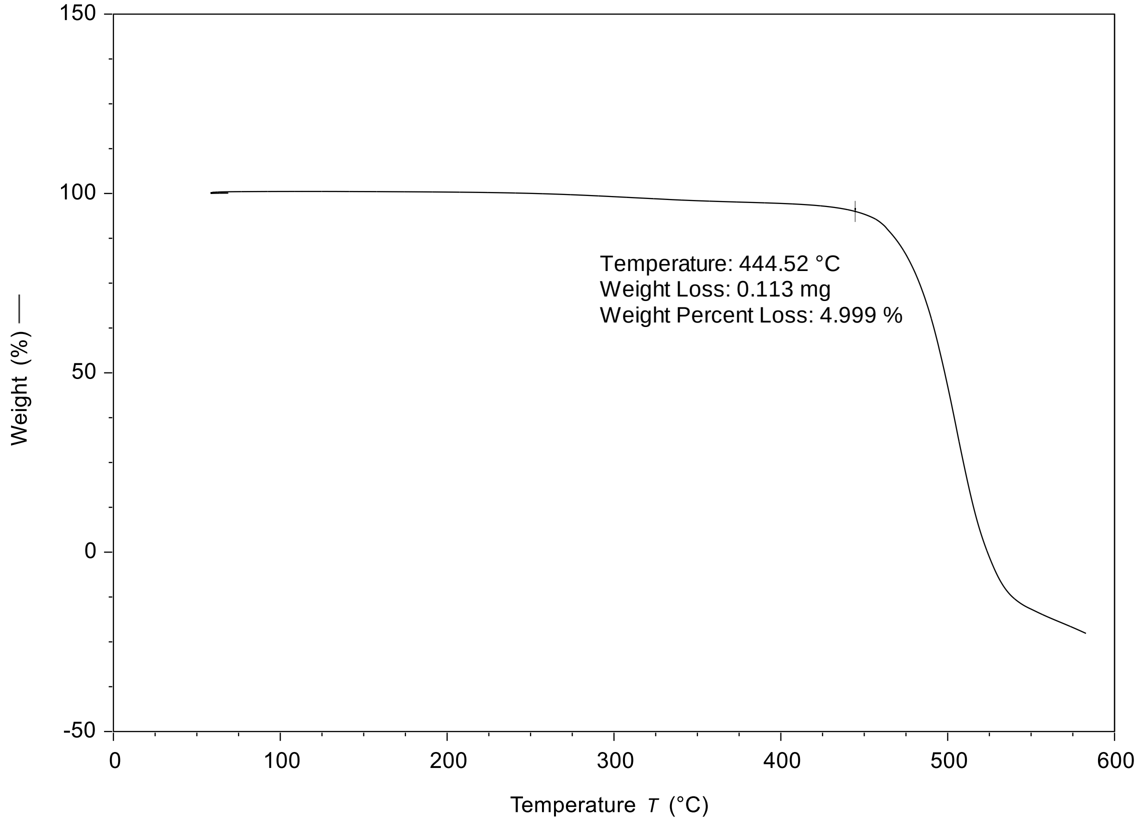

Temperature: 444.52 °C
Weight Loss: 0.113 mg
Weight Percent Loss: 4.999 %
Weight (%)
Temperature T (°C)
150
100
50
0
-50
0
100
200
300
400
500
600

4

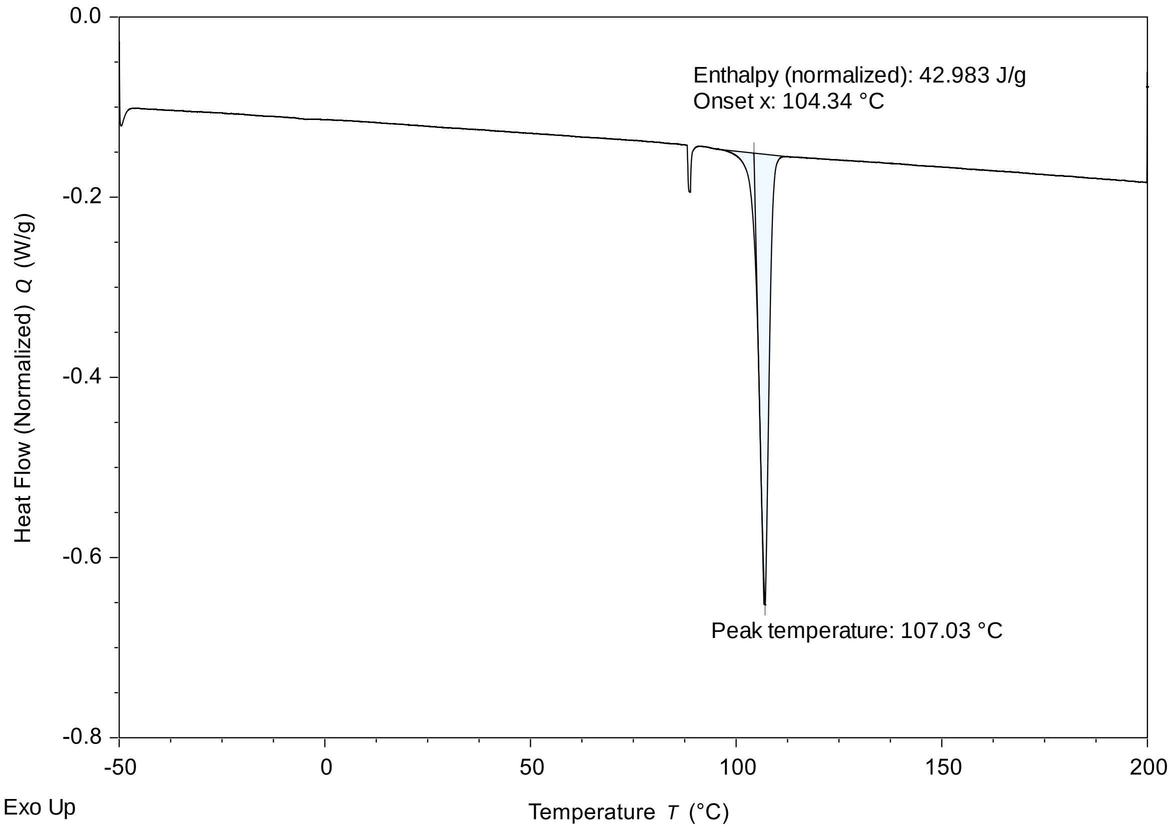

Enthalpy (normalized): 42.983 J/g
Onset x: 104.34 °C
Peak temperature: 107.03 °C
0.0
-0.2
-0.4
-0.6
-0.8
Heat Flow (Normalized) Q (W/g)
-50
0
50
100
150
200
Temperature T (°C)
Exo Up

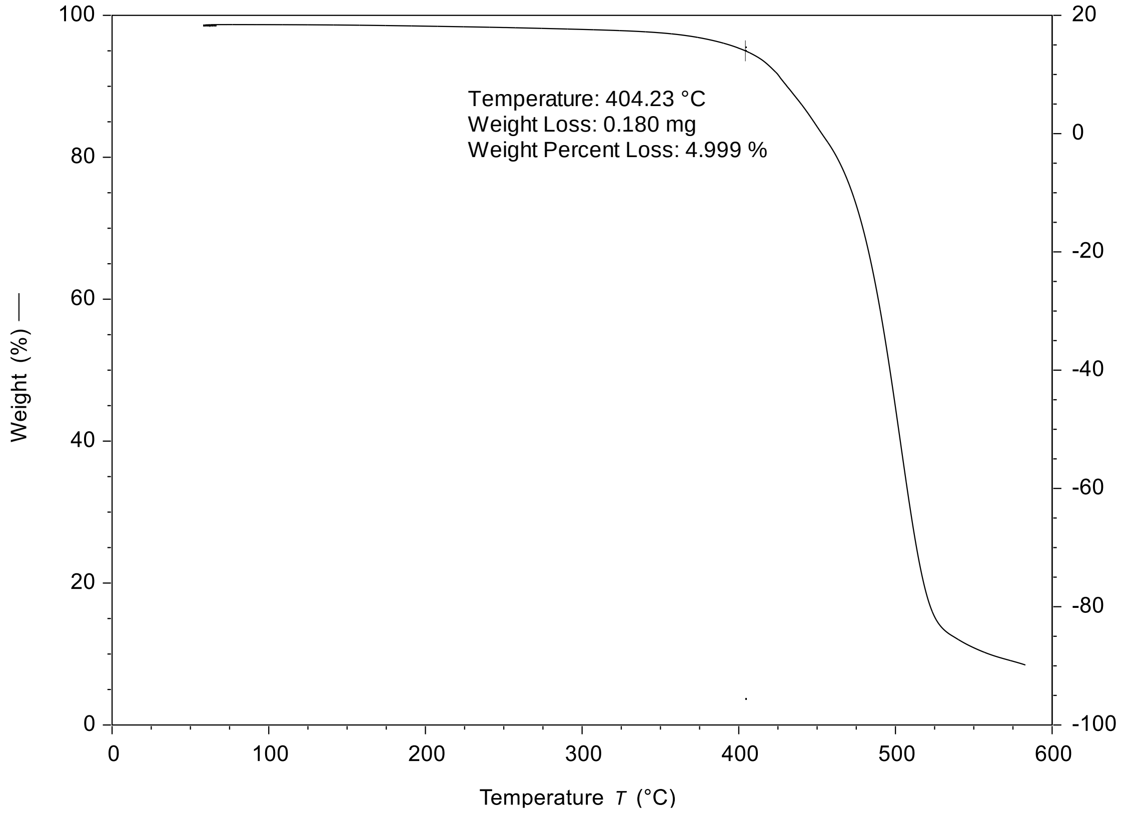

Temperature: 404.23 °C
Weight Loss: 0.180 mg
Weight Percent Loss: 4.999 %
100
80
60
40
20
0
Weight (%)
20
0
-20
-40
-60
-80
-100
0
100
200
300
400
500
600
Temperature T (°C)

5

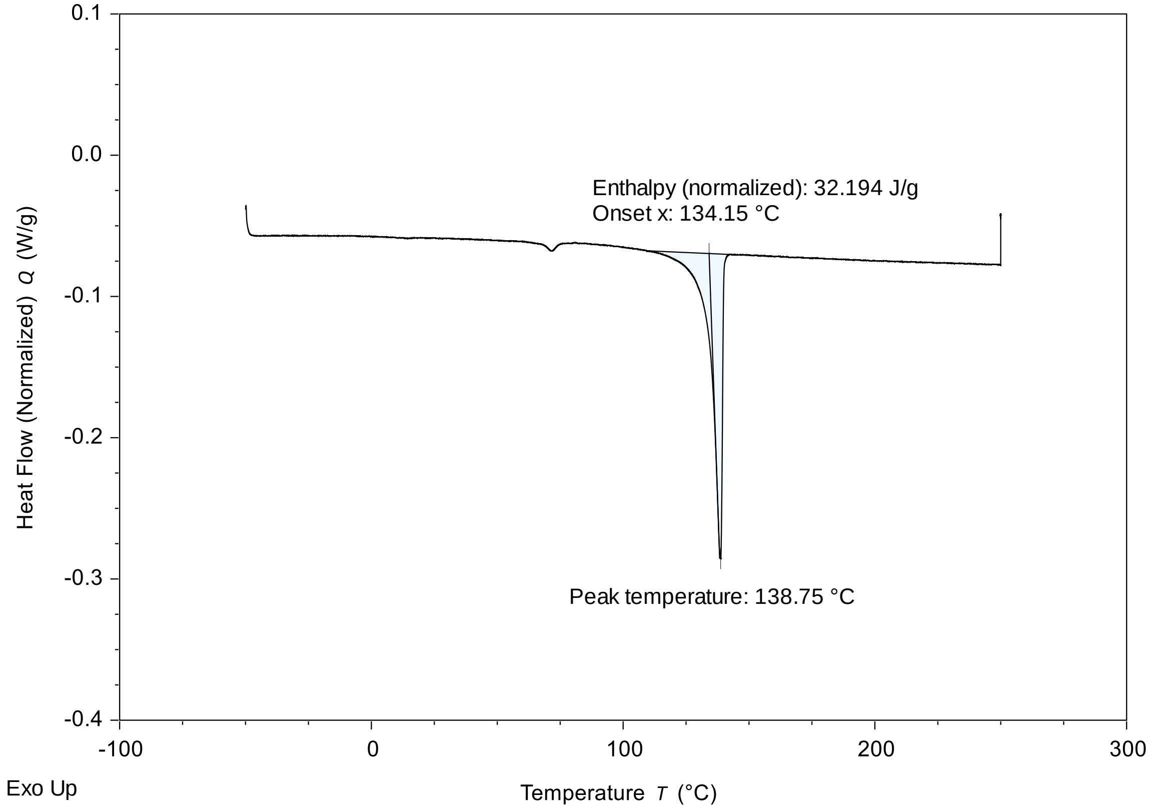

Enthalpy (normalized): 32.194 J/g
Onset x: 134.15 °C
Peak temperature: 138.75 °C
Heat Flow (Normalized) Q (W/g)
0.1
0.0
-0.1
-0.2
-0.3
-0.4
-100
0
100
200
300
Exo Up
Temperature T (°C)

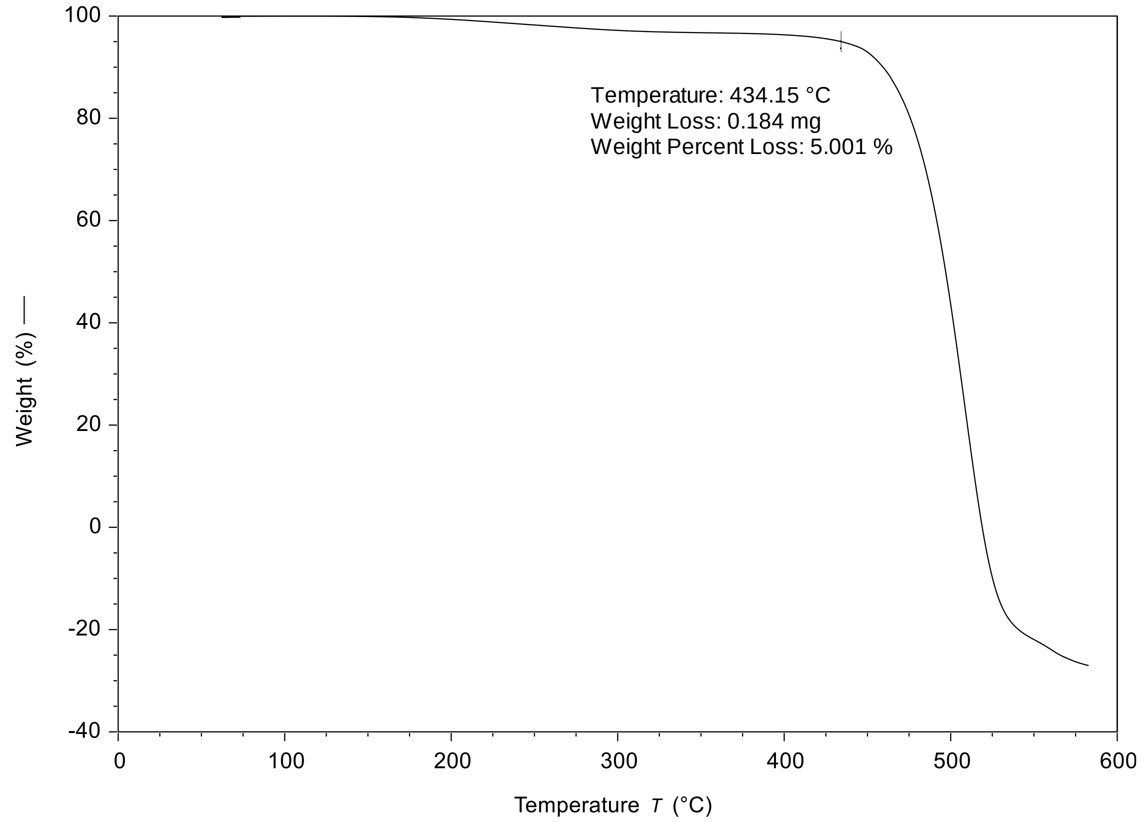

Temperature: 434.15 °C
Weight Loss: 0.184 mg
Weight Percent Loss: 5.001 %
Weight (%)
100
80
60
40
20
0
-20
-40
0
100
200
300
400
500
600
Temperature T (°C)

6

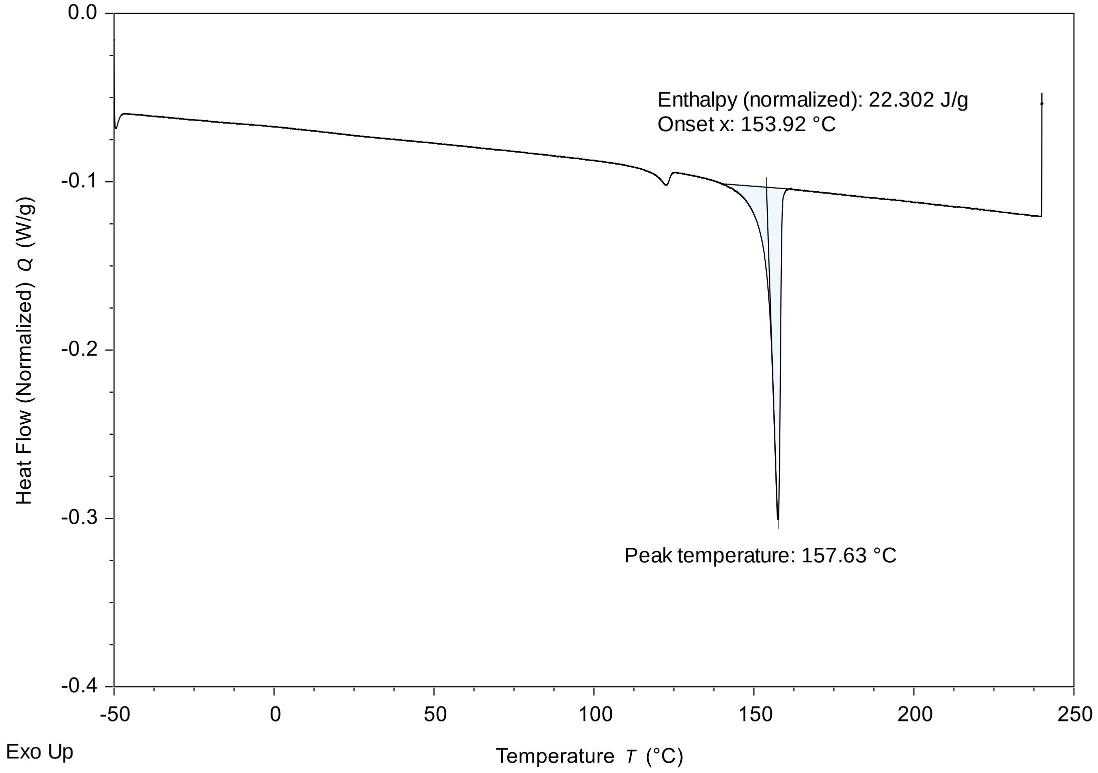

Enthalpy (normalized): 22.302 J/g
Onset x: 153.92 °C
Peak temperature: 157.63 °C
Heat Flow (Normalized) Q (W/g)
Temperature T (°C)
Exo Up
0.0
-0.1
-0.2
-0.3
-0.4
-50
0
50
100
150
200
250

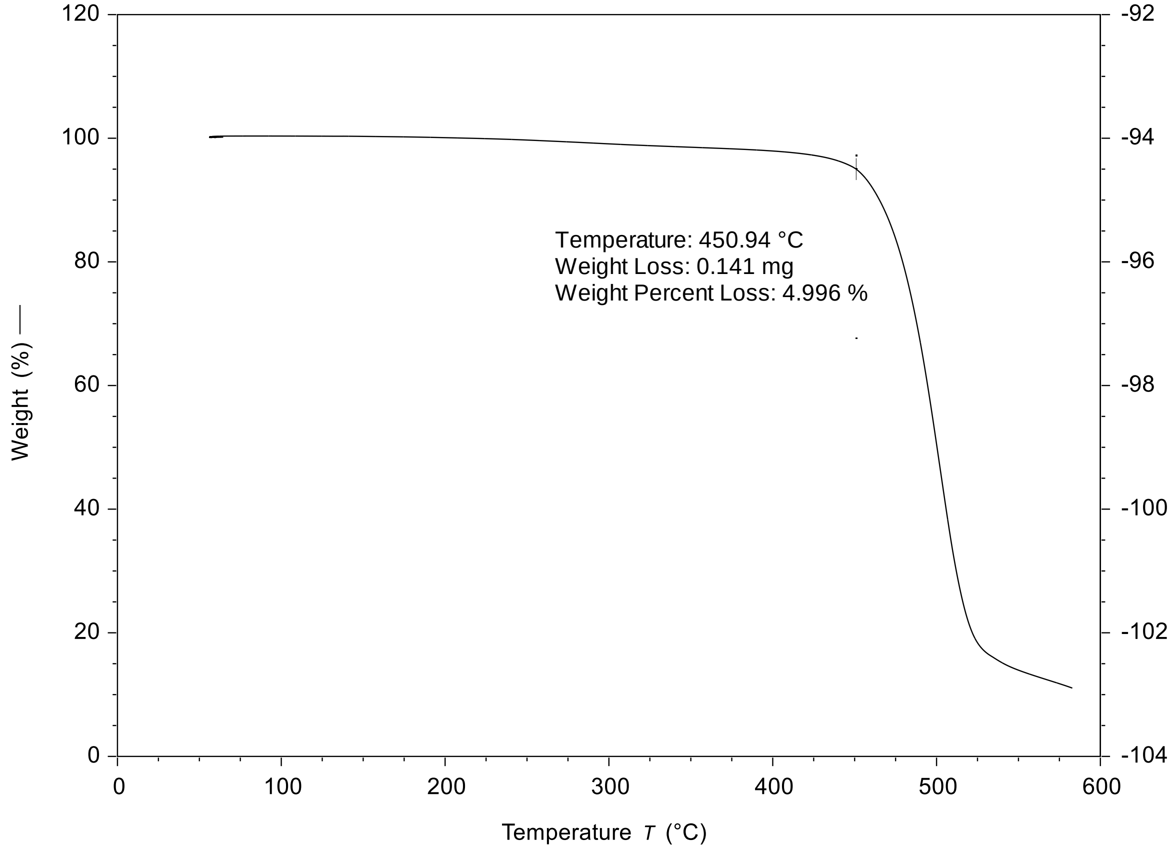

Temperature: 450.94 °C
Weight Loss: 0.141 mg
Weight Percent Loss: 4.996 %
Weight (%)
Temperature T (°C)
120
100
80
60
40
20
0
-92
-94
-96
-98
-100
-102
-104
0
100
200
300
400
500
600

7

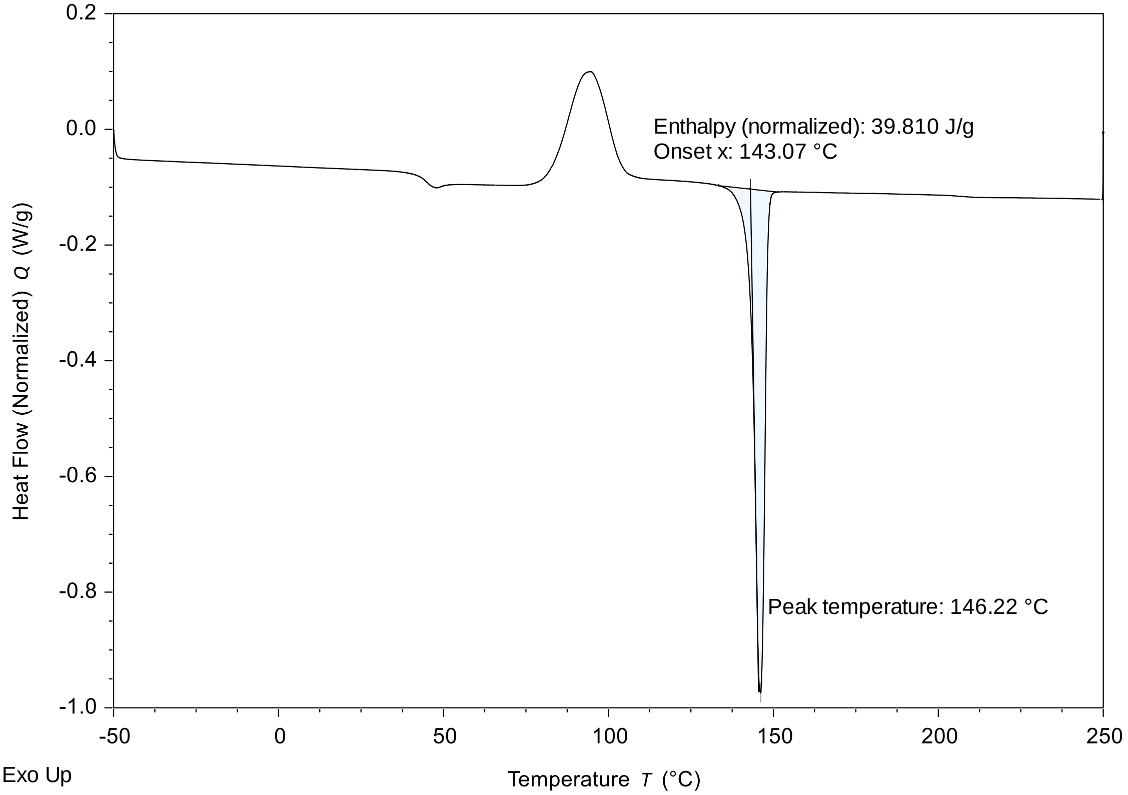
Enthalpy (normalized): 39.810 J/g
Onset x: 143.07 °C
Peak temperature: 146.22 °C
Heat Flow (Normalized) Q (W/g)
Temperature T (°C)
Exo Up

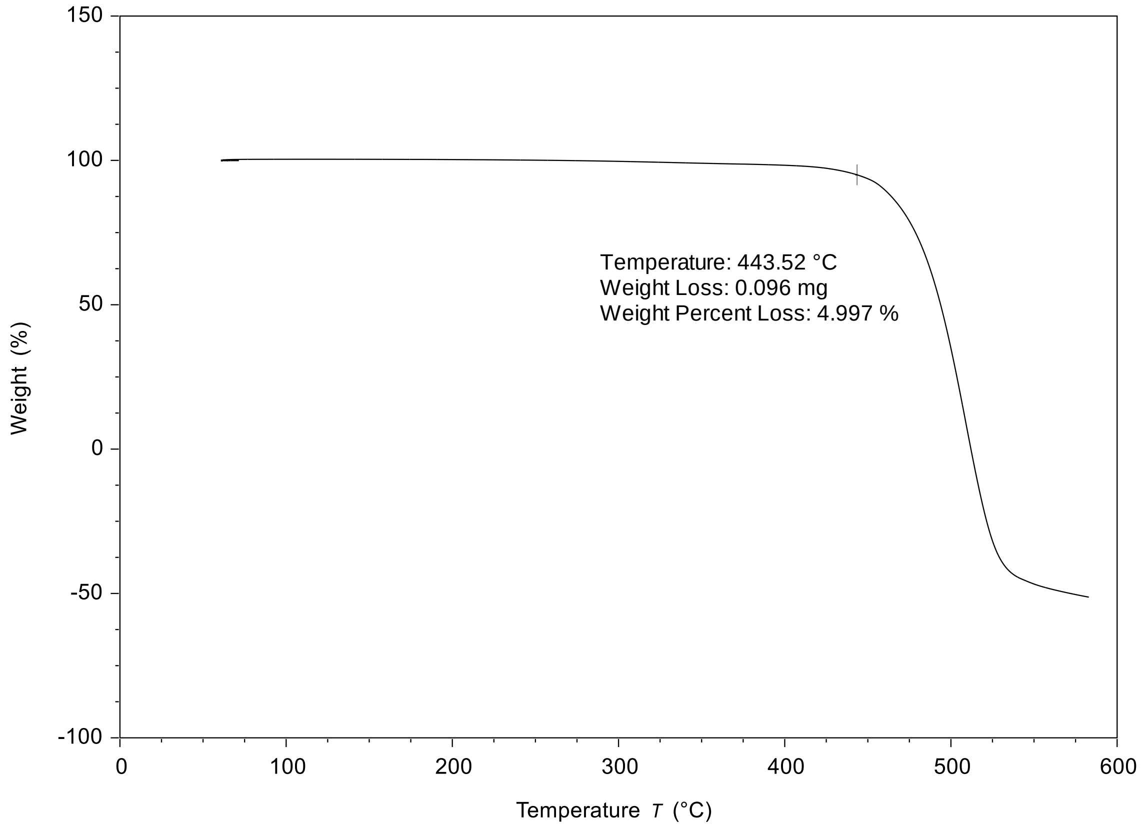
Temperature: 443.52 °C
Weight Loss: 0.096 mg
Weight Percent Loss: 4.997 %
Weight (%)
Temperature T (°C)

8

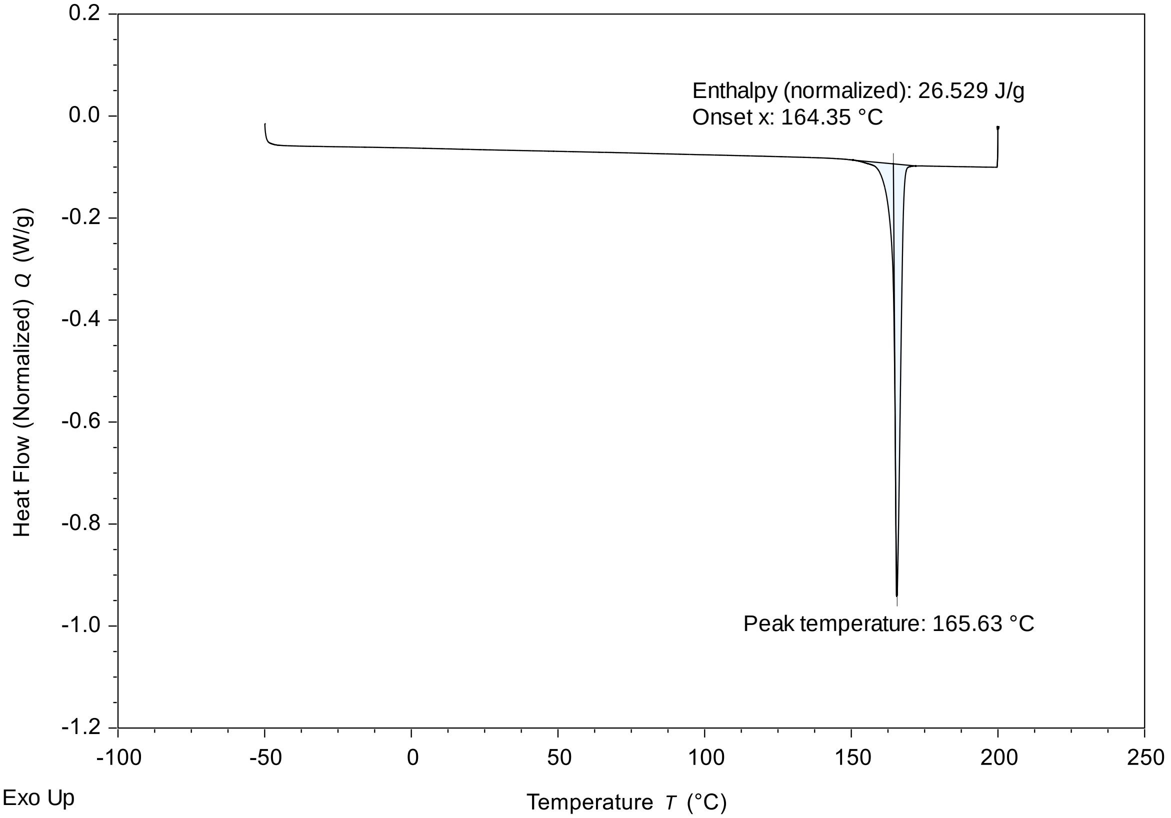
Enthalpy (normalized): 26.529 J/g
Onset x: 164.35 °C
Peak temperature: 165.63 °C
Heat Flow (Normalized) Q (W/g)
Temperature T (°C)
Exo Up
0.2
0.0
-0.2
-0.4
-0.6
-0.8
-1.0
-1.2
-100
-50
0
50
100
150
200
250

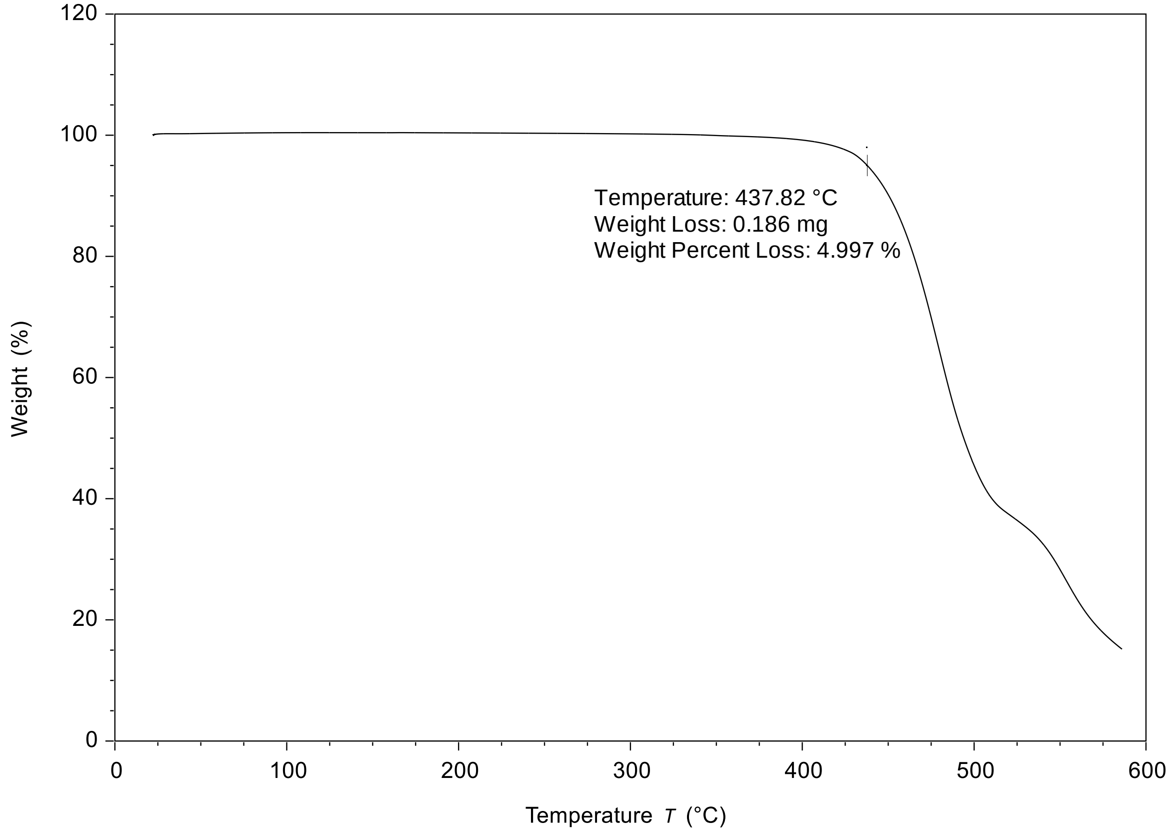
Temperature: 437.82 °C
Weight Loss: 0.186 mg
Weight Percent Loss: 4.997 %
Weight (%)
Temperature T (°C)
120
100
80
60
40
20
0
0
100
200
300
400
500
600

9

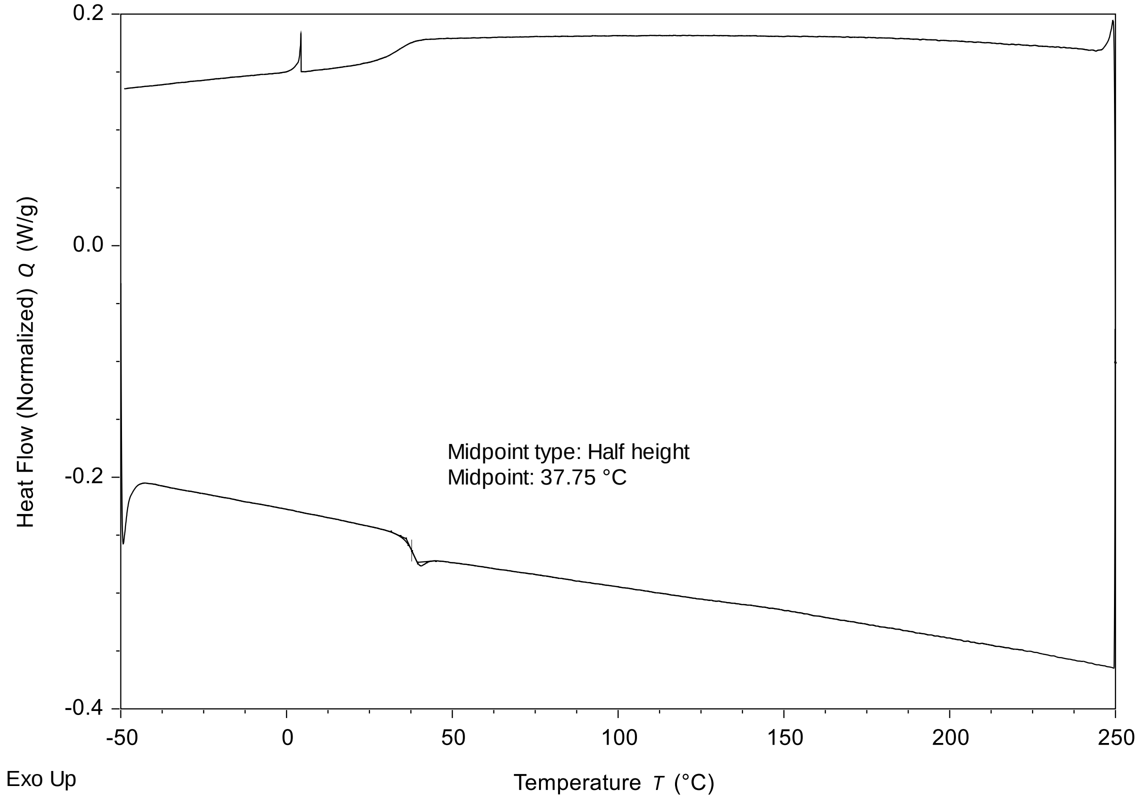
0.2
0.0
-0.2
-0.4
Heat Flow (Normalized) Q (W/g)
Midpoint type: Half height
Midpoint: 37.75 °C
-50
0
50
100
150
200
250
Exo Up
Temperature T (°C)

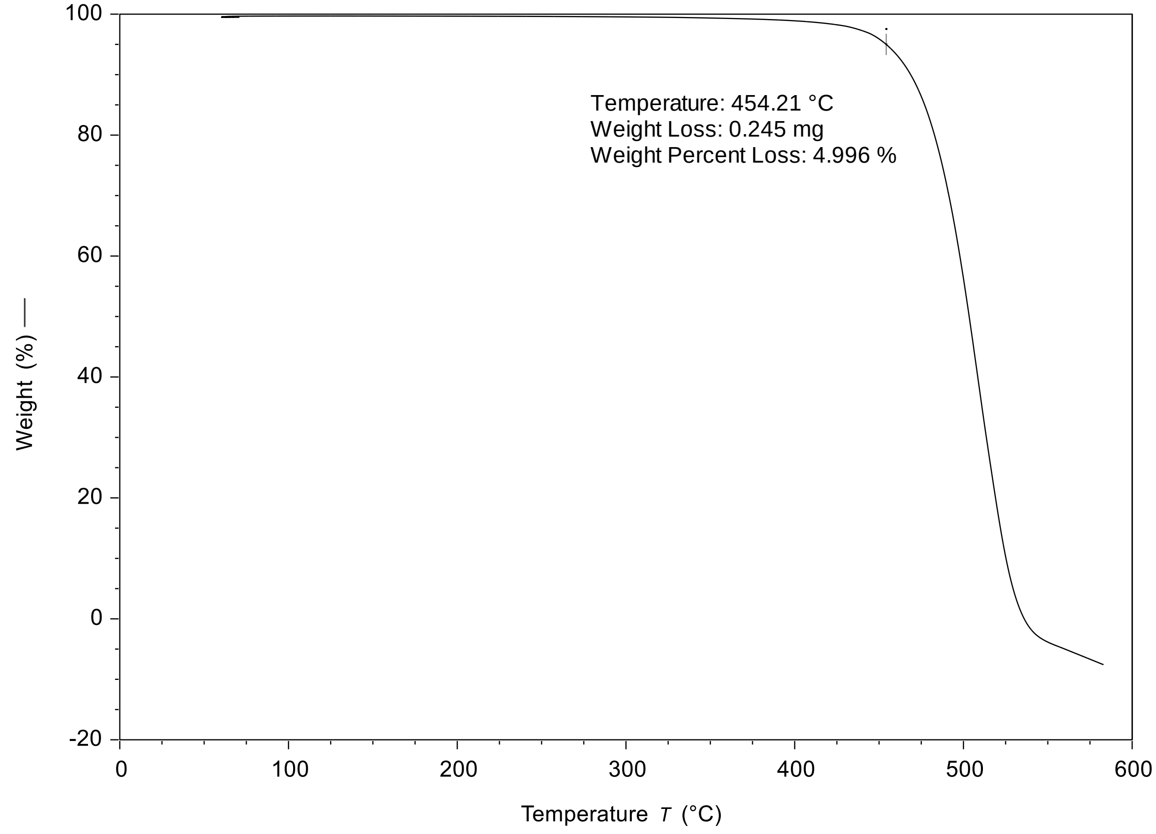
100
80
60
40
20
0
-20
Weight (%)
Temperature: 454.21 °C
Weight Loss: 0.245 mg
Weight Percent Loss: 4.996 %
0
100
200
300
400
500
600
Temperature T (°C)

10

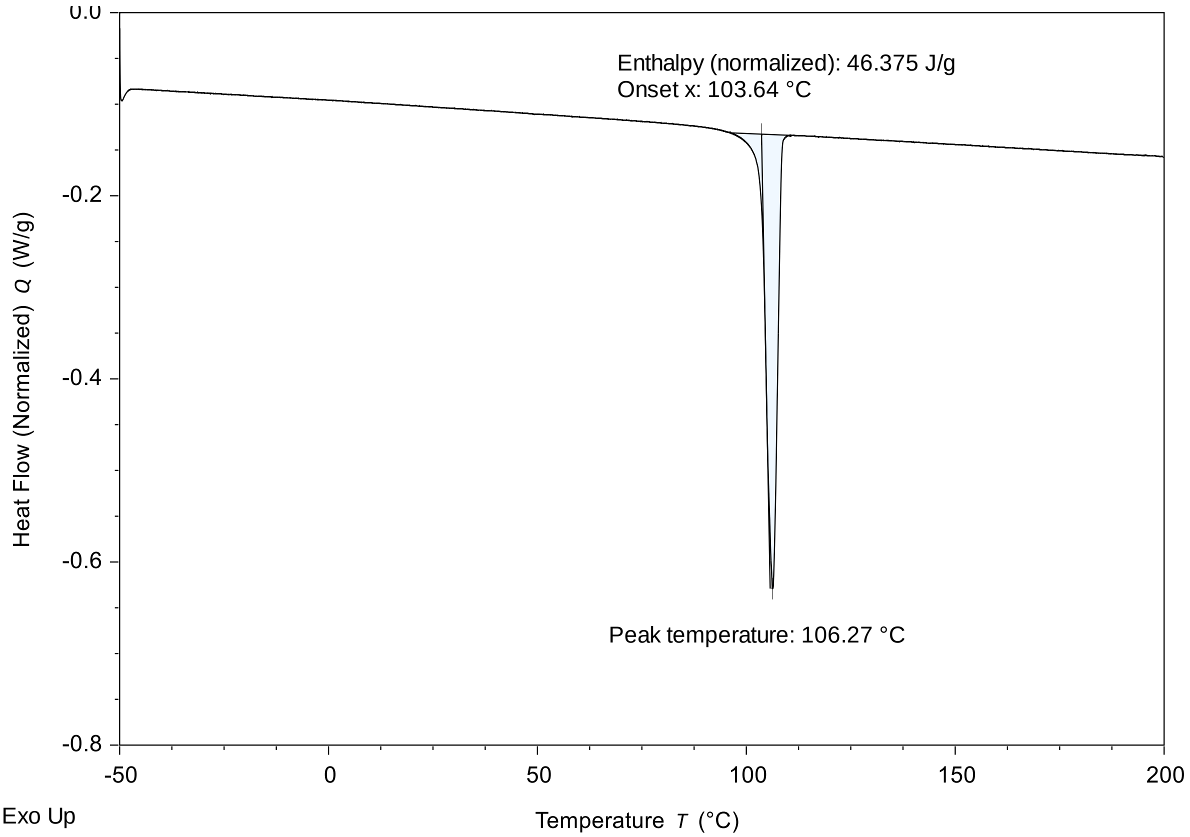
Enthalpy (normalized): 46.375 J/g
Onset x: 103.64 °C
Peak temperature: 106.27 °C
Heat Flow (Normalized) Q (W/g)
0.0
-0.2
-0.4
-0.6
-0.8
-50
0
50
100
150
200
Exo Up
Temperature T (°C)

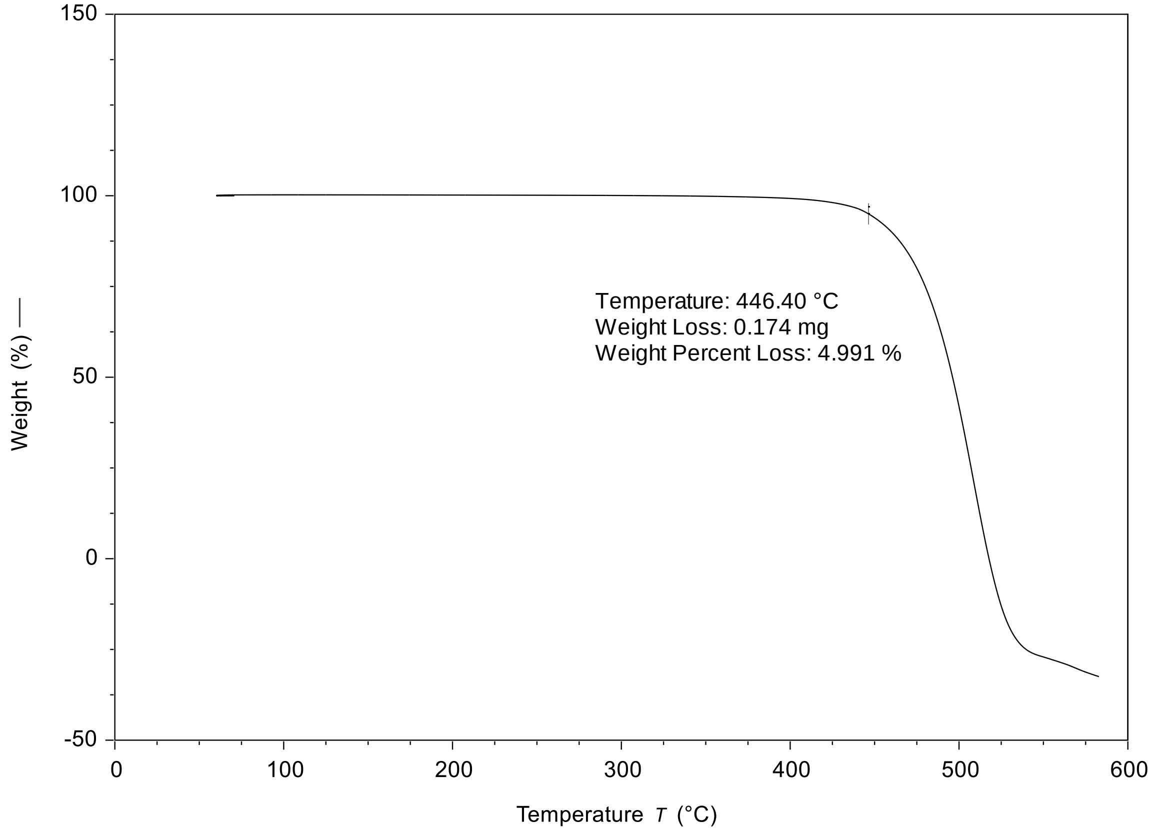
Temperature: 446.40 °C
Weight Loss: 0.174 mg
Weight Percent Loss: 4.991 %
Weight (%)
150
100
50
0
-50
0
100
200
300
400
500
600
Temperature T (°C)

11

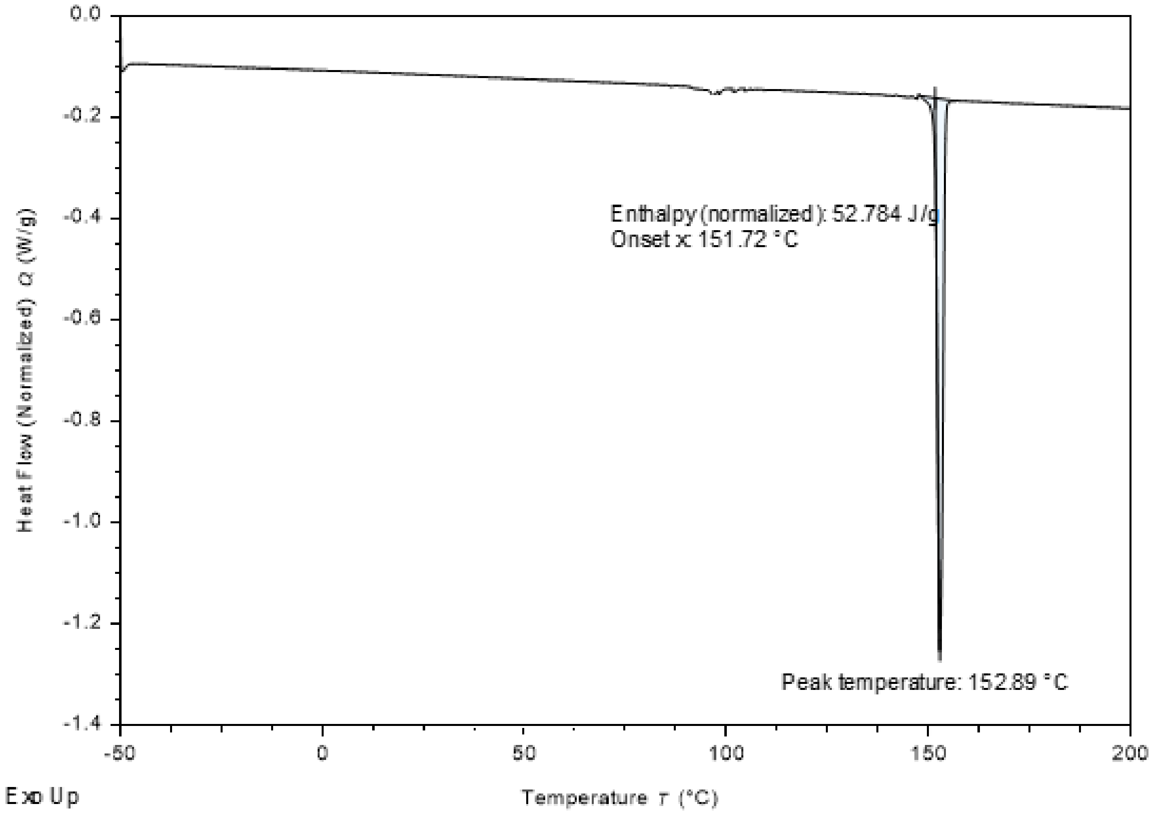
Heat Flow (Normalized) Q (W/g)
Enthalpy (normalized): 52.784 J/g
Onset x: 151.72 °C
Peak temperature: 152.89 °C
Temperature T (°C)
Exo Up

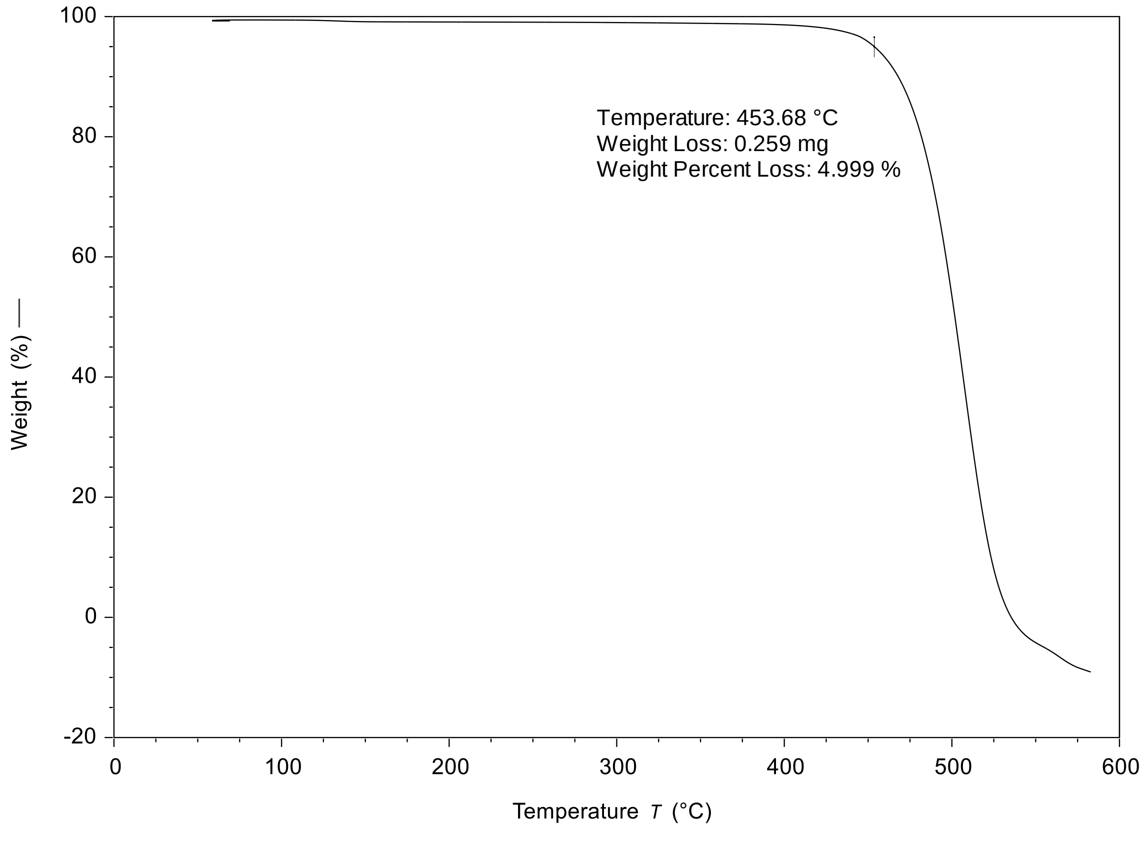
Weight (%)
Temperature: 453.68 °C
Weight Loss: 0.259 mg
Weight Percent Loss: 4.999 %
Temperature T (°C)

12

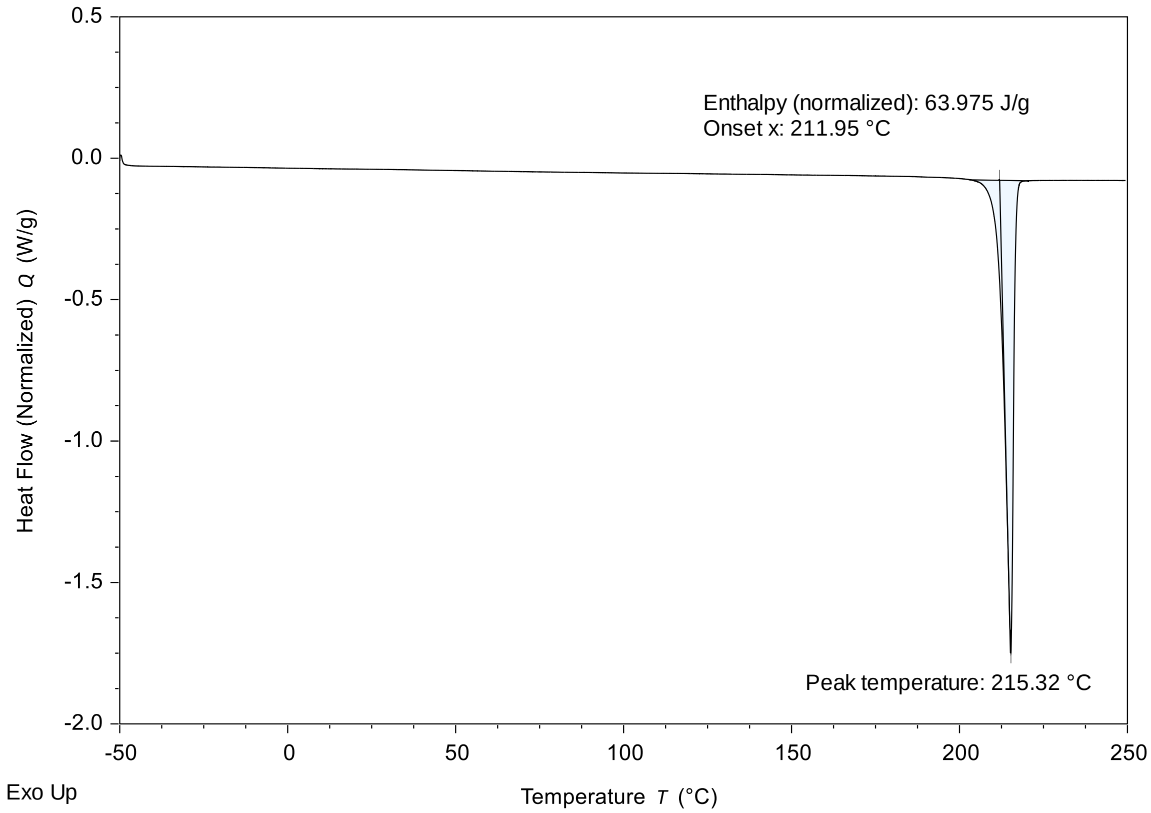
Enthalpy (normalized): 63.975 J/g
Onset x: 211.95 °C
Peak temperature: 215.32 °C
Heat Flow (Normalized) Q (W/g)
Temperature T (°C)
Exo Up
0.5
0.0
-0.5
-1.0
-1.5
-2.0
-50
0
50
100
150
200
250

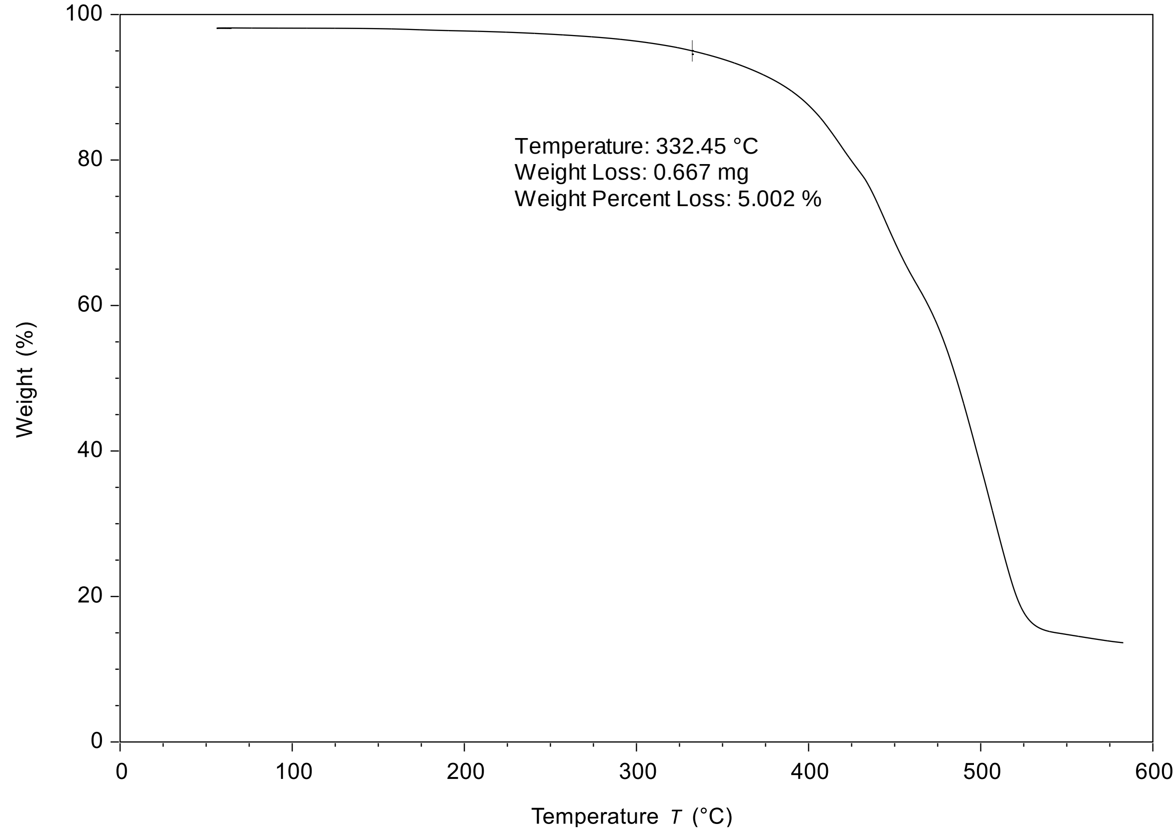
Temperature: 332.45 °C
Weight Loss: 0.667 mg
Weight Percent Loss: 5.002 %
Weight (%)
Temperature T (°C)
100
80
60
40
20
0
0
100
200
300
400
500
600

13

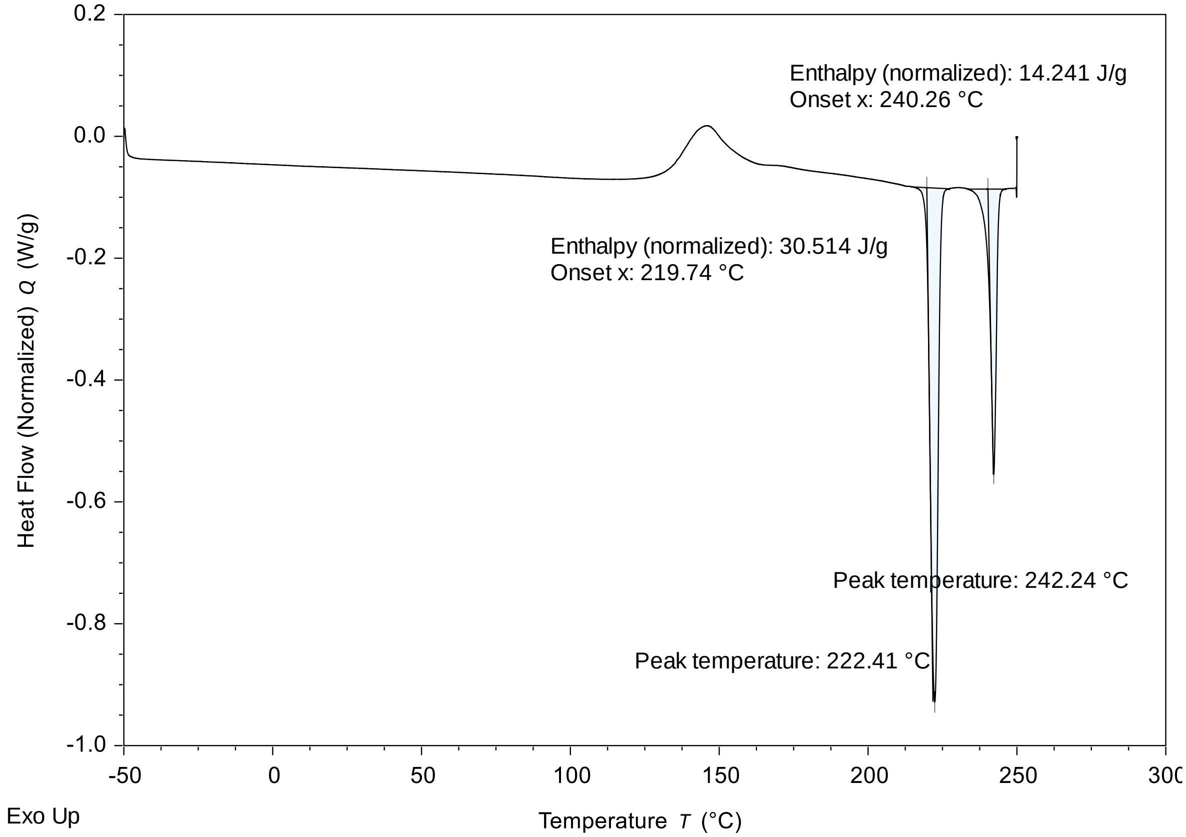
Enthalpy (normalized): 14.241 J/g
Onset x: 240.26 °C
Enthalpy (normalized): 30.514 J/g
Onset x: 219.74 °C
Peak temperature: 242.24 °C
Peak temperature: 222.41 °C
Heat Flow (Normalized) Q (W/g)
Temperature T (°C)
Exo Up
0.2
0.0
-0.2
-0.4
-0.6
-0.8
-1.0
-50
0
50
100
150
200
250
300

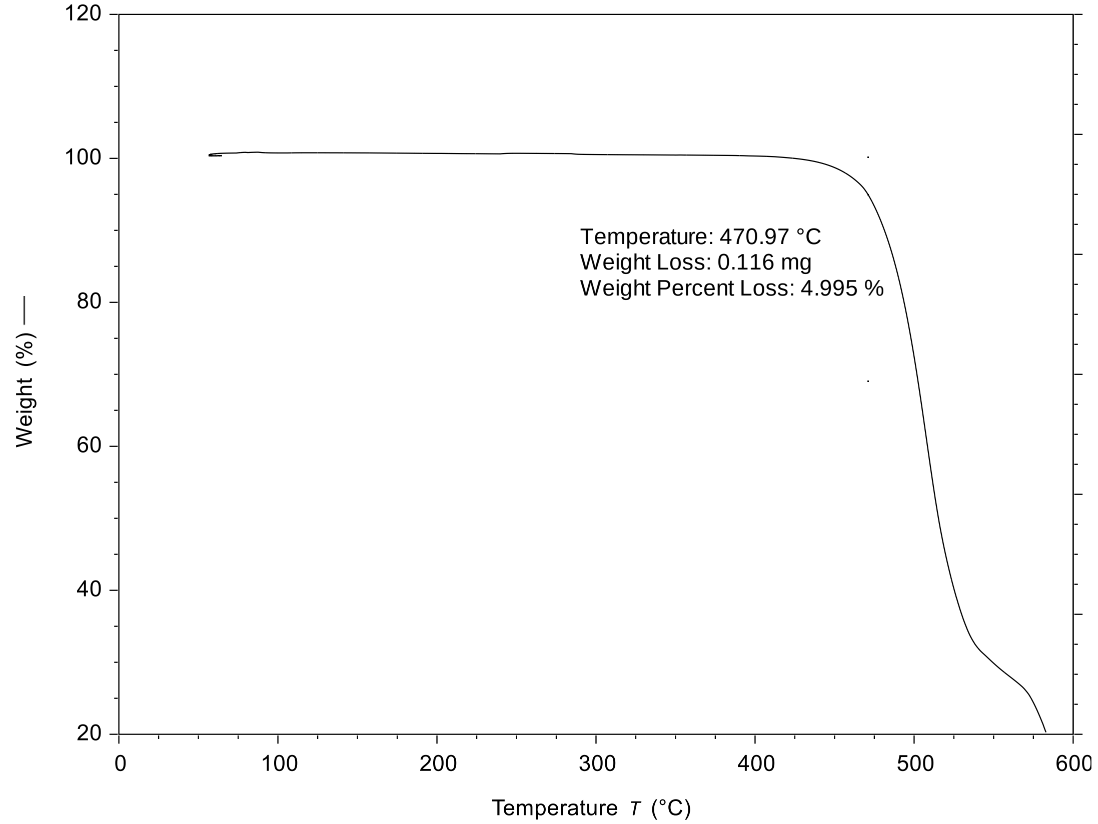
Temperature: 470.97 °C
Weight Loss: 0.116 mg
Weight Percent Loss: 4.995 %
Weight (%)
Temperature T (°C)
120
100
80
60
40
20
0
100
200
300
400
500
600

14

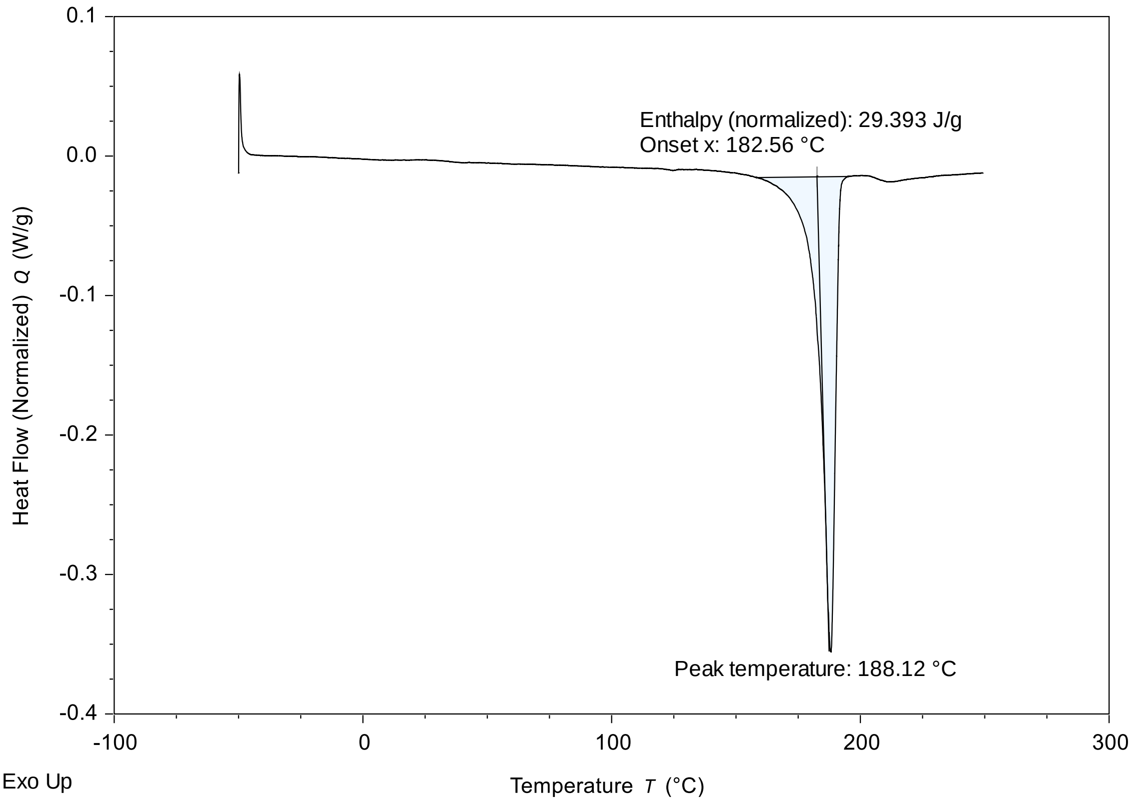
Enthalpy (normalized): 29.393 J/g
Onset x: 182.56 °C
Peak temperature: 188.12 °C
Heat Flow (Normalized) Q (W/g)
Temperature T (°C)
Exo Up
0.1
0.0
-0.1
-0.2
-0.3
-0.4
-100
0
100
200
300

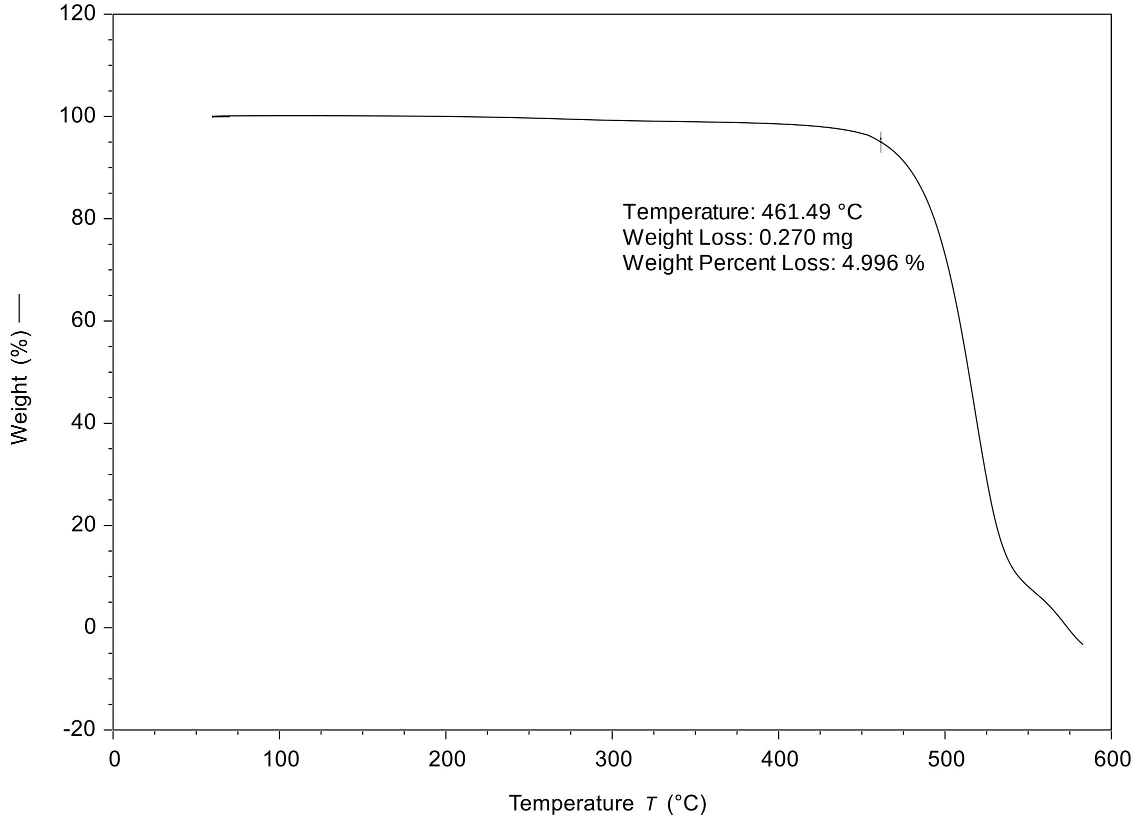
Temperature: 461.49 °C
Weight Loss: 0.270 mg
Weight Percent Loss: 4.996 %
Weight (%)
Temperature T (°C)
120
100
80
60
40
20
0
-20
0
100
200
300
400
500
600

15

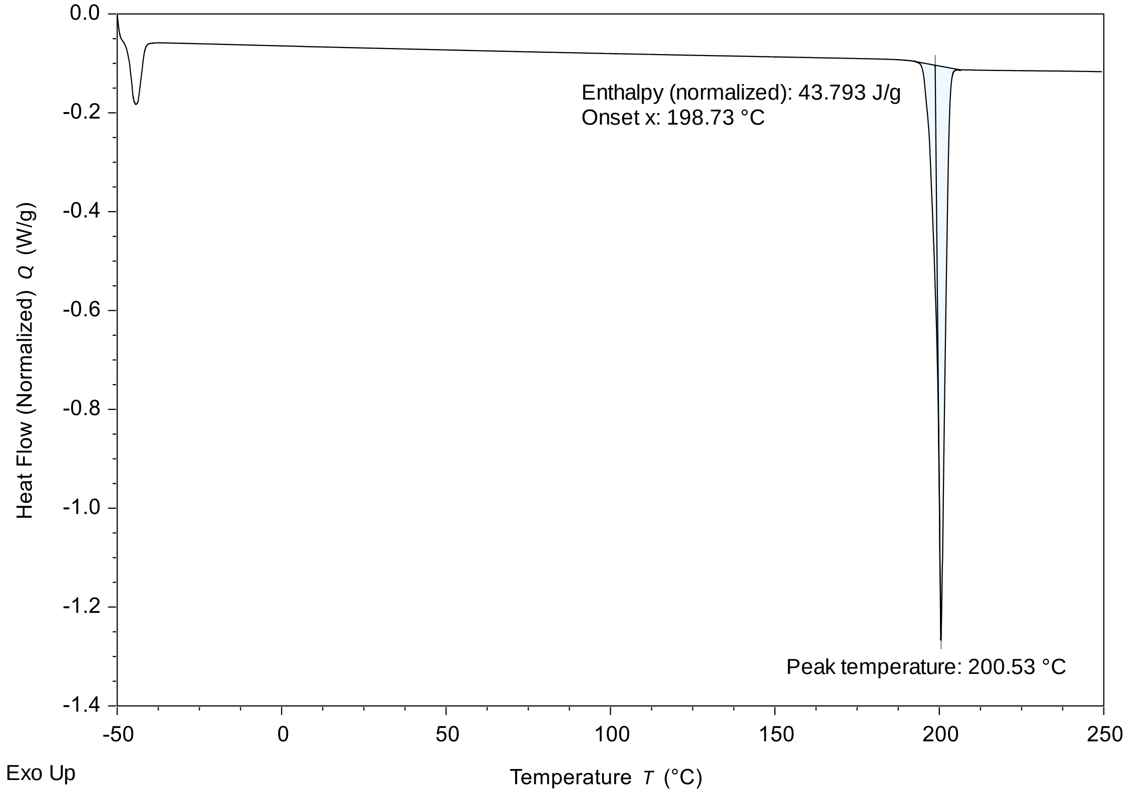

0.0
-0.2
-0.4
-0.6
-0.8
-1.0
-1.2
-1.4
Heat Flow (Normalized) Q (W/g)
Enthalpy (normalized): 43.793 J/g
Onset x: 198.73 °C
Peak temperature: 200.53 °C
-50
0
50
100
150
200
250
Exo Up
Temperature T (°C)

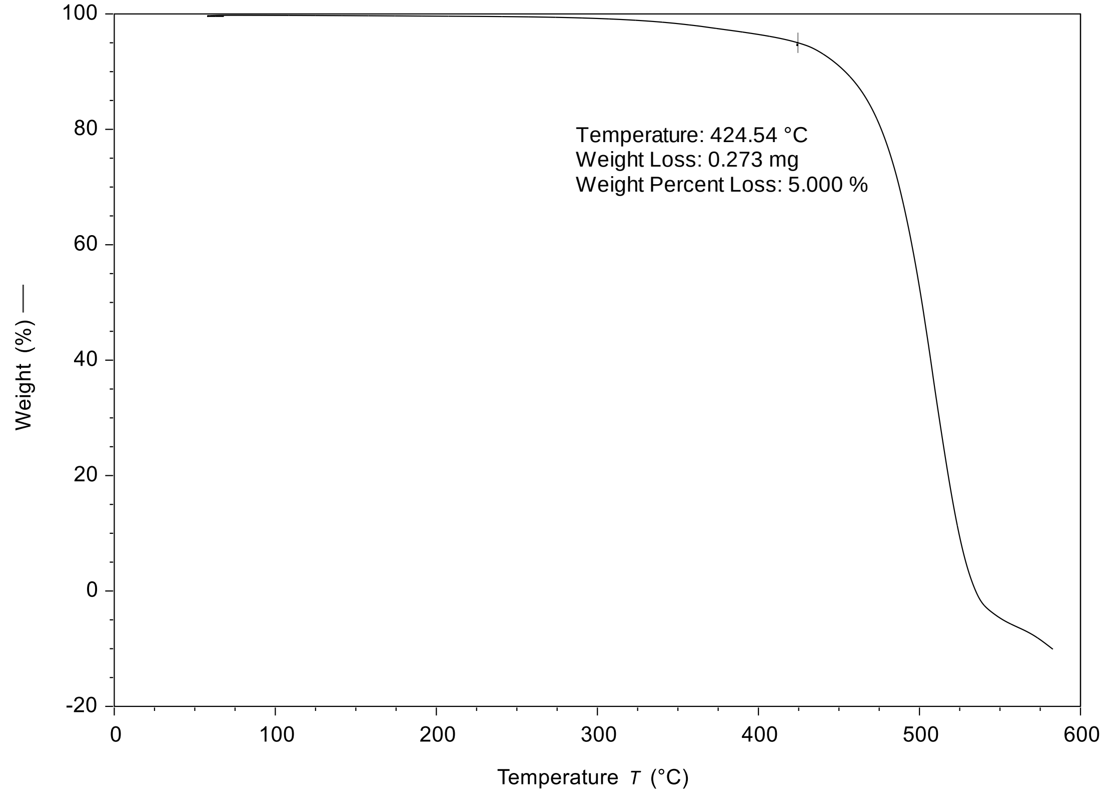

100
80
60
40
20
0
-20
Weight (%)
Temperature: 424.54 °C
Weight Loss: 0.273 mg
Weight Percent Loss: 5.000 %
0
100
200
300
400
500
600
Temperature T (°C)
